# Synthesis of Δ^1^-Pyrrolines via Formal (3 + 2)-Cycloaddition of 2*H*-Azirines with Enones Promoted by Visible Light under Continuous Flow

**DOI:** 10.1021/acsomega.5c01416

**Published:** 2025-04-23

**Authors:** Lorena S. R. Martelli, Lucas G. Furniel, Pedro H. O. Santiago, Javier Ellena, Arlene G. Corrêa

**Affiliations:** † Centre of Excellence for Research in Sustainable Chemistry, Department of Chemistry, Federal University of São Carlos, São Carlos, São Paulo 13565-905, Brazil; ‡ São Carlos Institute of Physics, University of São Paulo, São Carlos, São Paulo 13563-120, Brazil

## Abstract

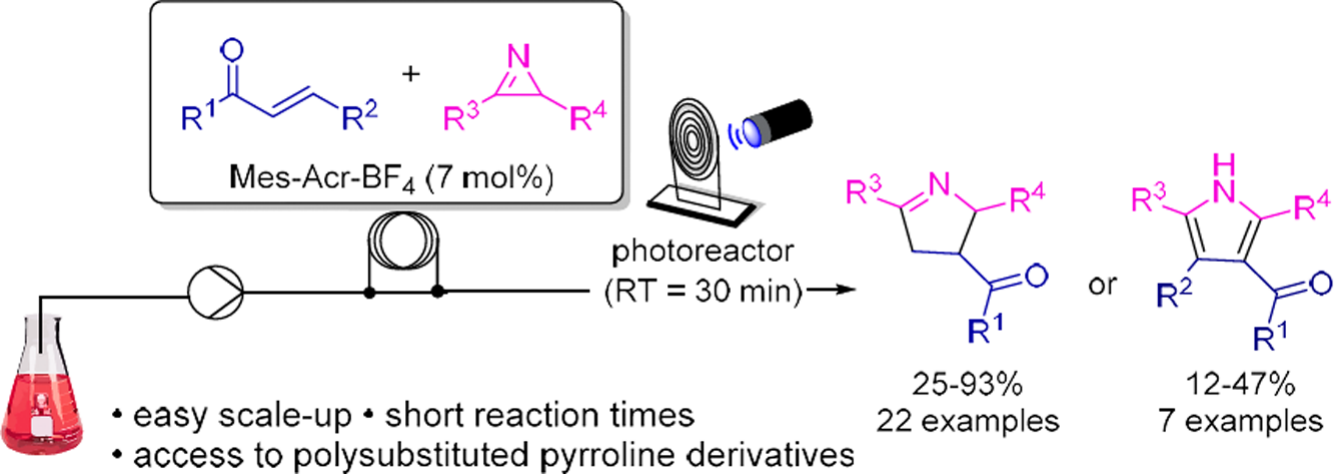

This work reports the first synthesis of Δ^1^-pyrrolines promoted by visible light under continuous flow, achieved through the formal (3 + 2)-cycloaddition of 2*H*-azirines to enones. A total of 22 examples of trisubstituted Δ^1^-pyrrolines were prepared in only 30 min of residence time, with 41–93% yield and diastereomeric ratios up to 7:3. Furthermore, continuous flow conditions were also effective when chalcones were used as starting materials, leading to the formation of 7 tetrasubstituted pyrroles with an overall yield ranging from 12 to 47%, via a photocatalyzed cycloaddition-oxidation sequence.

## Introduction

**Figure 1:**
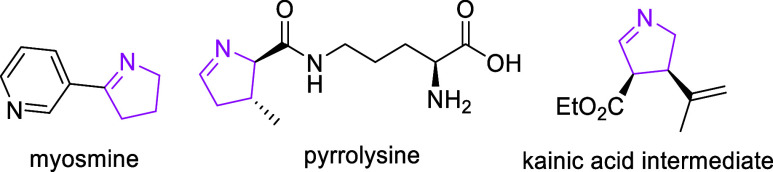
Examples of natural and synthetic Δ^1^-pyrrolines.

Five-membered *N*-heterocycles are widely found in various drugs and natural products, exhibiting significant biological activities.^1^ Among the azaarenes, Δ^1^-pyrroline is highlighted, being present, for example, in myosmine, an alkaloid found in tobacco, that releases nucleus accumbens dopamine in rats,^2^ and in pyrrolysine, an α-amino acid involved in the biosynthesis of proteins in some methanogenic archaea and bacteria.^3^ Moreover, Δ^1^-pyrrolines are key intermediates in the synthesis of natural products, such as the (−)-α-kainic acid (Figure 1).^4^

The importance of these compounds has driven the search for new synthetic methods to achieve this core. A commonly used strategy is the cyclization reaction of various starting materials such as amines,^5^ oximes,^6^ and imines.^7^ Liang et al. described the Michael addition of nitroalkanes to chalcones followed by reductive cyclization to afford the Δ^1^-pyrrolines,^8^ whereas Kempe and co-workers disclosed a nickel-catalyzed hydrogenation-cyclization sequence using γ-nitroketones as starting materials.^9^ Furthermore, a hypervalent iodine promoted (2 + 2 + 1) cycloaddition of aromatic ketones and alkylamines was reported to prepare this core.^10^

**Scheme 1:**
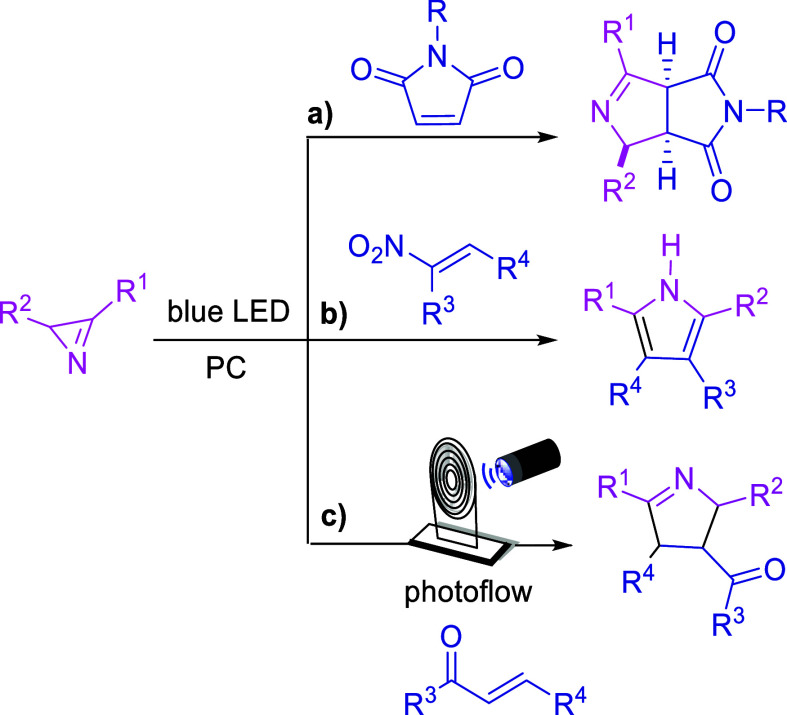
Different Approaches to Achieve Δ^1^-Pyrrolines via Photocatalysis

A different approach to achieve pyrrolines is based on the ring expansion of cyclobutanes^11^ and cyclopropanes.^12^ In this sense, formal (3 + 2) between 2*H*-azirines with various alkenes have been employed to prepare Δ^1^-pyrrolines,^13^ including metal-catalyzed protocols.^14^ Interestingly, this reaction can also be promoted photochemically under UV^15^ or visible-light irradiation as described by Zhang and coll. using maleimides (Scheme 1a).^16^ A limitation of this method is that the pyrroline is usually not isolated, been directly converted to the corresponding pyrrole,^18^ as reported, for example, by Rastogi for the formal (3 + 2)-cycloaddition of 2*H*-azirines and nitroalkenes using an organophotocatalyst followed by denitration (Scheme 1b).^17^

Photochemical/photocatalytic synthesis in continuous flow regime offers greater efficiency, ensuring uniform energy distribution and maximizing process efficiency, due to high-intensity irradiation and small solution quantities.^19^ Hence, photoflow has been extensively investigated in the past decade, overcoming challenges related to reaction scaling,^20^ accelerating reaction times, and reducing byproduct formation.^21^ In this respect, herein, we report the first synthesis of Δ^1^-pyrrolines promoted by visible light under continuous flow through the formal (3 + 2)-cycloaddition of 2*H*-azirines to enones (Scheme 1c).

## Results and Discussion

Based on precedents from literature,^22^ we began our optimization study using acrylophenone (**1**) and 2,3-diphenyl-2*H*-azirine (**2**) as starting materials in 1,2-dichloroethane and 9-mesityl-10-methylacridinium tetrafluoroborate as the photocatalyst (*E*_red_(*P*^+^*/*P*^•^) = +2.08 V), with 30 min of residence time (RT). To our delight, product **3a** was formed in 70% yield and 61:39 diastereoisomeric ratio (d.r.) (entry 1, Table 1). We then performed several tests in order to optimize the reaction conditions, and selected examples are shown in Table 1. As control experiments, without the photocatalyst or the light source, no reaction was observed (entries 2 and 3). Looking for greener solvents, 2-MeTHF and MeCN were tested but did not provide an increase in the yield and showed incomplete consumption of **1** (entries 4 and 5). Increased RT did not translate to a higher yield using MeCN as solvent (entry 6). Unfortunately, other solvents could not be employed due to low solubility of the reactants, which could lead to precipitates and obstruction of the flow device.

**Table 1:**
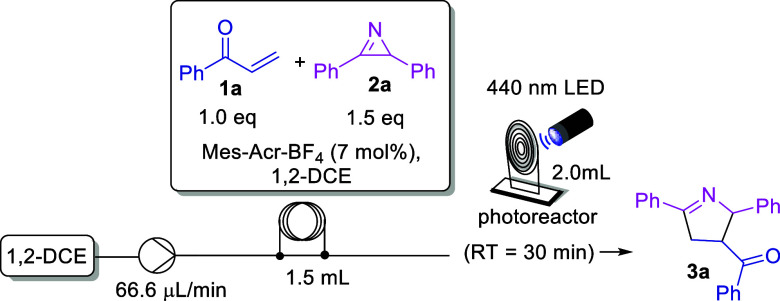
Optimization of the Photocatalyzed Reaction between Acrylophenone (**1a**) and 2*H*-Azirine (**2a**)

entry	deviation from the standard condition	**3a**, yield (%)^b^	d.r.^c^
1^a^	none	70	61:39
2	without photocatalyst	trace	
3	without light	NR^d^	
4	2-MeTHF	45	69:31
5	MeCN	65	47:53
6	MeCN, RT = 40 min	51	48:52
7	TPT (7 mol %)	traces	
8	4-CzlPN (7 mol %)	NR	
9	Mes-Acr-BF_4_ (5 mol %)	53	63:37
10	1.2 equiv of **2a**	54	59:41

a Conditions: **1a** (0.15 mmol), **2a** (0.225 mmol), photocatalyst (7 mol %), solvent (1.5 mL) under 440 nm (40 W) blue LED irradiation.

b After purification by column chromatography.

c d.r. is represented by the *trans*/*cis* ratio.

d NR = no reaction.

Alternative organophotocatalysts, such as 2,4,6-tris(4-methoxyphenyl)pyrylium tetrafluoroborate (TPT) (*E*_red_(*P*^+^*/*P*^•^) = +2.55 V),^22^ and 1,2,3,5-tetrakis(carbazol-9-yl)-4,6-dicyanobenzene (4-CzIPN) (*E*_red_(P*/*P*^–•^) = +1.35 V)^23^ were not able to promote this transformation (entries 7 and 8). Furthermore, lower catalyst or 2*H*-azirine loadings resulted in a decrease in the yield of **3a** (entries 9 and 10). Several attempts to promote the interconversion of the diastereomers using both pure *cis*- and *trans-***3a** and the crude reaction mixture, under acid and base catalysis in different solvents, resulted in incomplete epimerization, together with a significant amount of degradation (see Supporting Information Table S1).

**Scheme 2:**
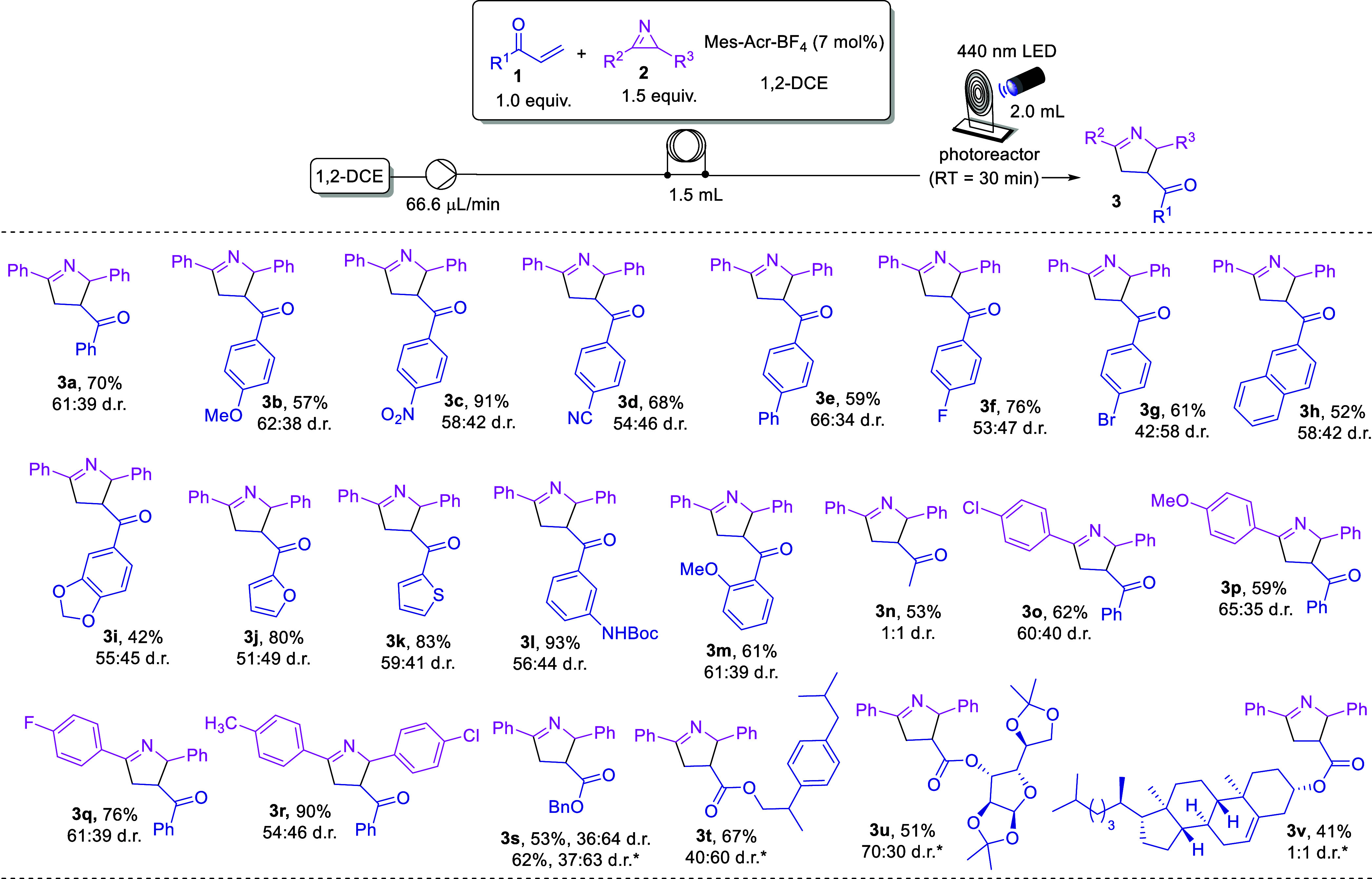
Scope and Limitations of the Photocatalyzed Cycloaddition between 2*H*-Azirines and Acrylophenones or Acrylates Conditions: **1a** (0.15 mmol), **2a** (0.225 mmol), photocatalyst (7 mol %) in 1,2-DCE (1.5 mL) under 440 nm (40 W) blue LED irradiation. *40 min of residence time. d.r. is represented by the *trans*/*cis* ratio.

With the best conditions in hand, we then studied the scope and limitations of this method, as depicted in Scheme 2. In all cases, the *cis*- and *trans*-isomers were isolated by column chromatography and fully characterized. Initially, acrylophenones bearing electron-donating and -withdrawing groups at the *para* position were screened (**3b**–**3g**). The higher yield was achieved with the electron-withdrawing group –NO_2_ (**3c**, 91%, 58:42 d.r.) and the best, although still low, diastereoselectivity observed for the *para*-substituted phenyl group (**3e**, 66:34 d.r.). 2-Naphthyl and 3,4-methylenedioxy substituted starting materials resulted in Δ^1^-pyrrolines with lower yields (**3h**–**3i**, 42–52%). Acrylophenones bearing heterocycles furnished the products in excellent yields (**3j** and **3k**, 80–83% yield). *Meta*-substituted acrylophenone containing the NHBoc group provided product **3l** in 93% and 56:44 d.r. whereas the 1-(2-methoxyphenyl)prop-2-en-1-one resulted in **3m** (61% yield), demonstrating no stereoelectronic effect. Interestingly, methyl vinyl ketone was also a suitable substrate for this reaction, providing product **3n** in 53% yield, although with no diastereoselectivity.

We then turned our attention to the effect of different substituents in the 2*H*-azirine. In this sense, 2*H*-azirines possessing methyl, halogen, and methoxy groups in the *para*-position were tested and, in all cases, the corresponding Δ^1^-pyrrolines **3o**–**r** were obtained in good to excellent yields (59–90%).

Gratifyingly, acrylates are also compatible substrates for this transformation. Using simple benzyl acrylate, product **3s** was formed in 53% and 36:64 d.r., under the standard conditions. However, in this case, it was observed by TLC that the acrylate was not totally consumed. Therefore, using 40 min of RT, we were able to increase the yield to 62% while maintaining the diastereoselectivity. This result prompted us to evaluate more complex acrylates under the reaction conditions. Thus, racemic ibuprofen, d-glucofuranose, and cholesterol derivatives **3t**–**v** were successfully obtained, in 41–67% yield.

One of the major advantages of the use of continuous flow conditions in organic synthesis is the easy reaction scale-up. By switching the sample loop from 1.5 to 10 mL, and using an operation time of 4 h (while maintaining a 30 min RT), we were able to obtain compound **3s** in 66% yield and 40:60 d.r.

**Figure 2:**
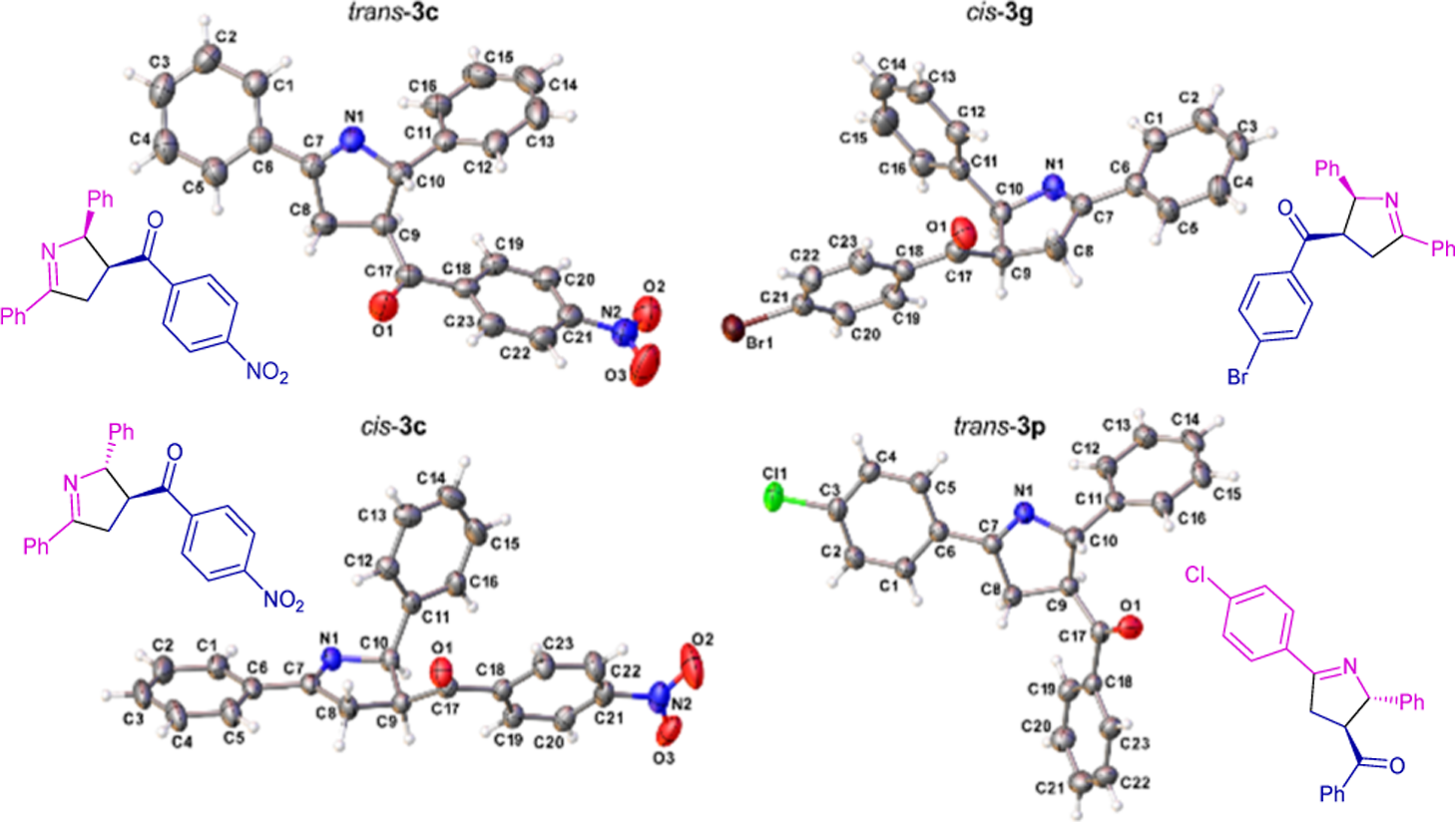
Crystal structures of *trans*- and *cis-***3c**, *cis*-**3g**, and *trans*-**3p**.

To unambiguously assign the relative stereochemistry, we performed single-crystal X-ray crystallography analysis of four compounds, as shown in Figure 2. For product **3c**, both diastereomers had their structures determined, with the major diastereomer assigned as *trans* and the minor as *cis*. Using the elucidated structures and comparing with the ^1^H NMR coupling constants, for each compound described in Scheme 2, the diastereomeric ratios are displayed as *trans*-isomer (less polar): *cis*-isomer (more polar).

We then envisioned using chalcones instead of acrylophenones since it would allow access to tetrasubstituted Δ^1^-pyrrolines. To our delight, using chalcone **4a** and 2*H*-azirine **2a**, under the standard conditions, we were able to obtain the corresponding Δ^1^-pyrroline **3aa** in 82% yield (73:27 d.r.).^24^ However, the major *anti/anti*-Δ^1^-pyrroline isomer showed instability in solution, proving the characterization of the product to be challenging. Therefore, we decided to perform one-pot oxidation to obtain the corresponding pyrrole **5a**. For this purpose, we used two blue LED lamps to generate the Δ^1^-pyrroline **3aa** and then added two equiv of 2,3-dichloro-5,6-dicyano-1,4-benzoquinone (DDQ) as oxidant, under reflux in toluene for 30 min, affording the pyrrole **5a** in 44% overall yield.

**Scheme 3:**
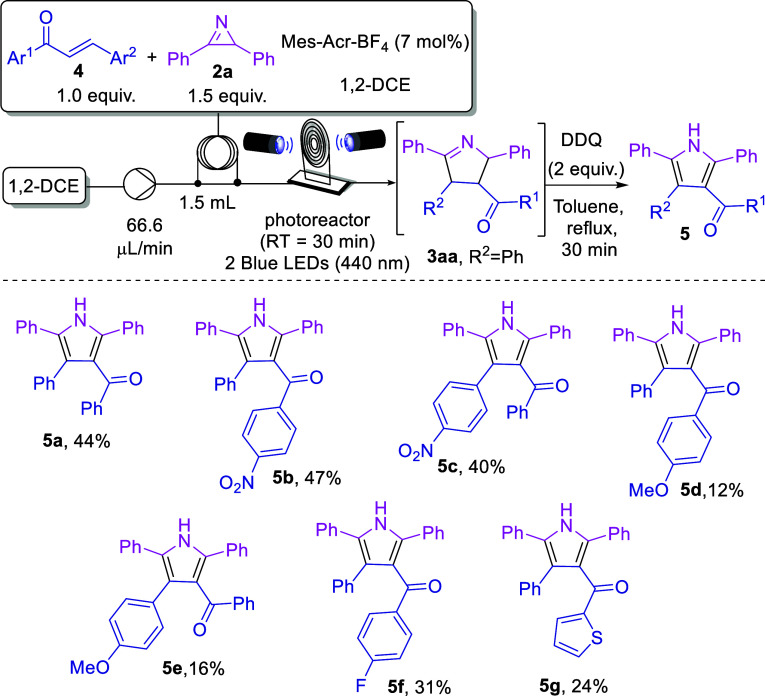
Scope and Limitations of the Photocatalyzed Cycloaddition between *2H*-Azirines and Chalcones Followed by Aromatization Conditions: **4** (0.18 mmol), **2a** (0.27 mmol), photocatalyst (7 mol %) in 1,2-DCE (1.5 mL) under 440 nm (40 W) blue LED irradiation. Then, DDQ (0.3 mmol), 2 mL of toluene, sealed tube, reflux.

Next, we assessed the effect of different substituents on chalcone (Scheme 3). Thus, the electron-withdrawing group nitro was effective, producing **5b**–**c** in 47 and 40% yield, respectively. In contrast, an electron-donating group such as methoxy afforded **5d**–**e** in lower yields (12 and 16%, respectively), probably due to the reduced formation of the pyrroline intermediary. Moreover, fluorine- and thiophen-substituted chalcones provided **5f** and **5g** in moderate yields (31 and 24%, respectively).

**Scheme 4:**
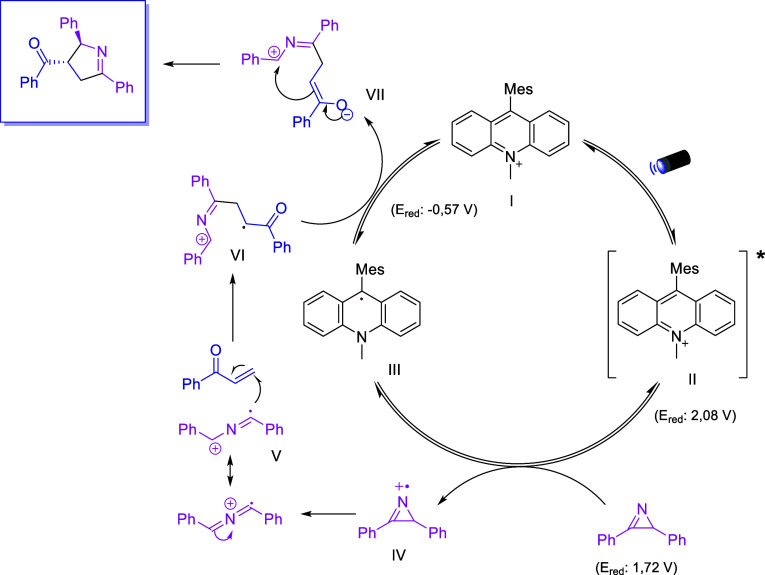
Proposed Reaction Mechanism for the Photocatalytic Formal (3 + 2)-Cycloaddition of *2H*-Azirines with Enones

The mechanism of the photocatalytic formal (3 + 2)-cycloaddition using *2H*-azirines has been widely studied.^25^ Therefore, to confirm the presence of radicals in the reaction, we performed the reaction in the presence of the radical scavenger TEMPO. Although the reaction was not completely inhibited, the resulting decrease in the yield (from 70 to 46%) of **3a** is indicative of the presence of radical species (see Supporting Information). Based on this result and literature reports for similar reaction systems, the proposed mechanism is depicted in Scheme 4.^25^ Upon light irradiation, the photoexcited catalyst oxidizes the 2*H*-azirine to its radical cation, which suffers a ring-opening rearrangement, generating species V. This nucleophilic iminyl radical then attacks the enone (Giese addition), furnishing intermediate VI, which after reduction and intramolecular cyclization yields the Δ^1^-pyrroline as the reaction product.

## Conclusion

In conclusion, the synthesis of Δ^1^-pyrrolines via 2*H*-azirines ring-opening/cycloaddition was developed, using a continuous photoflow platform, resulting in a robust and versatile procedure. Under optimal conditions, 22 examples of trisubstituted pyrrolines were prepared in only 30 min of RT, with yields ranging from 41 to 93% and diastereomeric ratios up to 7:3. When chalcones were employed as starting materials, after a photocatalytic cycloaddition-oxidation sequence, 7 tetrasubstituted pyrroles were obtained with yields in the range of 12–47%. These photoflow conditions provided an easy and reliable protocol for scaling up this methodology.

## Experimental Section

### General Information

All reagents used were commercially available from Sigma-Aldrich, Synth, Exodus, and Merck. The solvents used are from commercial sources and when necessary, dry solvents were treated as recommended in the literature.^26^ Purification of the products was performed by flash column chromatography with silica gel 60, 230–400 mesh ASTM Merck, silica gel 60 A, 70–230 mesh Aldrich Co. TLC analysis was performed on silica gel chromatoplates 60 F_254_ Merck KGaA. Nuclear magnetic resonance spectra were recorded on Bruker ARX 400 MHz spectrometers. Chemical shifts (δ) are expressed in ppm referenced by the residual solvent signal or TMS and coupling constants (*J*) in hertz (Hz). To indicate the multiplicity of signs, the following abbreviation was used: s (singlet), br (broad singlet), d (doublet), t (triplet), q (quadruplet), and m (multiplet). HRMS-ESI analyses were performed on an Agilent 6545 qTOF MS system (Agilent Technologies, Santa Clara, CA, USA) with a jet electrospray interface (ESI) in positive mode. IR spectra were generated on a Shimadzu spectrophotometer, IR Spirit-X Series. The samples were diluted in dichloromethane and applied in a diamond ATR module. GC–MS analyses were performed on a Shimadzu GCMS-QP2010S with electron impact (EI) ionization using a Zebron-ZB-5MS Column. Melting points were obtained using Büchi equipment, model M-560, and reported in degrees Celsius (°C). For the photocatalyzed reactions, Kessil lamps, model PR160L, 440 nm blue LED (40 W) were used. The continuous flow reactions were carried out on Syrris ASIA Flow Chemistry Systems model 2200292 equipment. The photochemical reactor was made on a 3D printer and coupled with a 3.98 m × 0.8 mm PTFE tube. The reduction potential was determined on the IKA ElectraSyn 2.0 equipment against the Ag/Ag^+^ pseudoreference electrode. Single-crystal X-ray diffraction analyses were executed at 210 K using a Rigaku XtaLAB Synergy-S Dualflex diffractometer, equipped with a HyPix-6000HE detector system, and Cu Kα (1.54184 Å) radiation.

### General Procedure for Obtaining Δ:^1^-Pyrrolines

In a Schlenk tube were added the corresponding azirine **2** (0.27 mmol), acrylophenone **1** (0.18 mmol), mesityl acridinium tetrafluoroborate (5.03 mg, 0.0126 mmol, 7 mol %), and anhydrous DCE (1.8 mL). This mixture was degassed (freeze–pump–thaw) three times. The reaction was then submitted to continuous flow in a Syrris ASIA equipment. The solvent (DCE) in the solvent reservoir flow was previously degassed for 5 min in an ultrasound bath with an N_2_ balloon. The reaction setup is shown in Figure S2. The mixture was then transferred with the aid of a syringe to a 1.5 mL loop coupled to an injection pump and pumped at a flow rate of 66.6 μL/min into a 2 mL reactor under irradiation from a lamp of 440 nm blue LED (40 W) at a 10 cm distance from the reactor with a RT of 30 min. The photochemical reactor was made on a 3D printer and coupled with a 3.98 m × 0.8 mm PTFE tube. The reaction crude was collected and concentrated under vacuum and purified with flash column chromatography (silica gel) using hexane-EtOAc 90:10 to 80:20 as eluent.

#### (±)-(*trans*-2,5-Diphenyl-3,4-dihydro-2*H*-pyrrol-3-yl)(phenyl)methanone (**3a**)

The product was obtained as an off-white solid in 46% yield (22.5 mg, 0.069 mmol). ^1^H NMR (400 MHz, CDCl_3_): δ 7.91–7.84 (m, 2H), 7.77–7.70 (m, 2H), 7.49 (t, *J* = 7.4 Hz, 1H), 7.44–7.32 (m, 5H), 7.29–7.18 (m, 3H), 7.18–7.12 (m, 2H), 5.55 (d, *J* = 6.0 Hz, 1H), 4.11 (dt, *J* = 9.6, 6.6 Hz, 1H), 3.62–3.35 (m, 2H). ^13^C{^1^H} NMR (100 MHz, CDCl_3_): δ 199.6, 171.5, 142.9, 136.2, 133.7, 133.5, 131.0, 128.9, 128.7, 128.7, 128.6, 128.1, 127.5, 126.9, 79.6, 53.7, 40.0. mp 86.3–87.8 °C. HRMS (ESI-TOF) *m*/*z*: [M + H]^+^ calcd for C_23_H_20_NO, 326.1539; found, 326.1555. IR (ν_max_): 1680, 1622, 1597, 1577, 1494, 1448, 1338, 1240, 1024, 759, 694 cm^–1^.

#### (±)-(*cis*-2,5-Diphenyl-3,4-dihydro-2*H*-pyrrol-3-yl)(phenyl)methanone (**3a**)

The product was obtained as an off-white solid in 25% yield (12.1 mg, 0.037 mmol). ^1^H NMR (400 MHz, CDCl_3_): δ 8.06–7.98 (m, 2H), 7.70–7.65 (m, 2H), 7.56–7.44 (m, 4H), 7.36 (t, *J* = 7.7 Hz, 2H), 7.06–6.95 (m, 3H), 6.79–6.74 (m, 2H), 5.91 (dd, *J* = 9.6, 2.1 Hz, 1H), 4.75 (dt, *J* = 9.5, 8.2 Hz, 1H), 4.02 (ddd, *J* = 17.5, 8.0, 2.3 Hz, 1H), 3.24 (dd, *J* = 17.5, 9.3 Hz, 1H). ^13^C{^1^H} NMR (100 MHz, CDCl_3_): δ 198.1, 173.6, 137.6, 134.0, 133.0, 131.2, 128.7, 128.5, 128.2, 128.2, 128.2, 128.1, 128.0, 127.6, 79.4, 50.6, 37.2. mp 82.2–83.8 °C. HRMS (ESI-TOF) *m*/*z*: [M + H]^+^ calcd for C_23_H_20_NO, 326.1539; found, 326.1556. IR (ν_max_): 1680, 1614, 1598, 1448, 1342, 1230, 1026, 759, 725, 692 cm^–1^.

#### (±)-(*trans*-2,5-Diphenyl-3,4-dihydro-2*H*-pyrrol-3-yl)(4-methoxyphenyl)methanone (**3b**)

The product was obtained as a white solid in 36% yield (19.0 mg, 0.054 mmol). ^1^H NMR (400 MHz, CDCl_3_): δ 7.95 (d, *J* = 6.6 Hz, 2H), 7.80 (d, *J* = 8.9 Hz, 2H), 7.44 (m, 3H), 7.37–7.18 (m, 5H), 6.88 (d, *J* = 8.9 Hz, 2H), 5.58 (d, *J* = 5.9 Hz, 1H), 4.13 (m, 1H), 3.86 (s, 3H), 3.58 (ddd, *J* = 16.9, 6.9, 1.3 Hz, 1H), 3.49 (ddd, *J* = 17.1, 9.7, 2.0 Hz, 1H). ^13^C{^1^H} NMR (100 MHz, CDCl_3_): δ 198.3, 171.6, 163.9, 143.3, 134.0, 131.3, 131.0, 129.4, 128.8, 128.6, 128.2, 127.6, 127.0, 114.0, 80.0, 55.6, 53.6, 40.3. mp 123–124 °C. HRMS (ESI-TOF) *m*/*z*: [M + H]^+^ calcd for C_24_H_22_NO_2_, 356.1645; found, 356.1650.

#### (±)-(*cis*-2,5-Diphenyl-3,4-dihydro-2*H*-pyrrol-3-yl)(4-methoxyphenyl)methanone (**3b**)

The product was obtained as a white solid in 22% yield (11.7 mg, 0.033 mmol).^1^H NMR (400 MHz, CDCl_3_): δ 7.94 (m, 2H), 7.63 (d, *J* = 8.8 Hz, 2H), 7.48–7.34 (m, 3H), 7.02–6.85 (m, 3H), 6.78 (d, *J* = 8.8 Hz, 2H), 6.72 (m, 2H), 5.81 (dd, *J* = 9.6, 2.0 Hz, 1H), 4.61 (dd, *J* = 17.8, 9.3 Hz, 1H), 3.91 (ddd, *J* = 17.4, 8.2, 2.3 Hz, 1H), 3.78 (s, 3H), 3.12 (dd, *J* = 17.4, 9.3 Hz, 1H). ^13^C{^1^H} NMR (100 MHz, CDCl_3_): δ 196.3, 173.8, 163.4, 137.7, 134.0, 131.1, 130.7, 130.5, 128.7, 128.2, 128.0, 127.9, 127.6, 113.7, 79.5, 55.6, 50.4, 37.2. mp 150–151 °C. HRMS (ESI-TOF) *m*/*z*: [M + H]^+^ calcd for C_24_H_22_NO_2_, 356.1645; found, 356.1650.

#### (±)-(*trans*-2,5-Diphenyl-3,4-dihydro-2*H*-pyrrol-3-yl)(4-nitrophenyl)methanone (**3c**)

The product was obtained as a yellow solid in 53% yield (29.5 mg, 0.80 mmol). ^1^H NMR (400 MHz, CDCl_3_): δ 8.24 (d, *J* = 7.6 Hz, 2H), 7.94 (m, 4H), 7.54–7.38 (m, 3H), 7.36–7.29 (m, 3H), 7.19 (d, *J* = 6.7 Hz, 2H), 5.51 (d, *J* = 5.2 Hz, 1H), 4.19 (m, 1H) 3.65 (dd, *J* = 17.1, 6.8 Hz, 1H), 3.54 (dd, *J* = 17.1, 9.6 Hz, 1H). ^13^C{^1^H} NMR (100 MHz, CDCl_3_): δ 198.3, 171.6, 150.6, 142.4, 140.8, 133.5, 131.3, 130.0, 129.1, 128.8, 128.2, 128.1, 127.0, 124.0, 80.0, 54.6, 39.7. mp 129–131 °C. HRMS (ESI-TOF) *m*/*z*: [M + H]^+^ calcd for C_23_H_19_N_2_O_3_, 370.1317; found, 371.1397.

#### (±)-(*cis*-2,5-Diphenyl-3,4-dihydro-2*H*-pyrrol-3-yl)(4-nitrophenyl)methanone (**3c**)

The product was obtained as a yellow solid in 38% yield (21.1 mg, 0.057 mmol). ^1^H NMR (400 MHz, CDCl_3_): δ 8.16 (d, *J* = 8.8 Hz, 2H), 8.00 (d, *J* = 6.6 Hz, 2H), 7.72 (d, *J* = 8.8 Hz, 2H), 7.54–7.46 (m, 3H), 7.13–6.88 (m, 3H), 6.89–6.72 (m, 2H), 5.88 (dd, *J* = 9.6, 1.5 Hz, 1H), 4.74 (td, *J* = 9.5, 7.1 Hz, 1H), 4.03 (ddd, *J* = 17.5, 7.0, 2.2 Hz, 1H), 3.28 (dd, *J* = 17.5, 9.4 Hz, 1H). ^13^C{^1^H} NMR (100 MHz, CDCl_3_): δ 197.2, 173.5, 150.1, 142.2, 137.5, 133.5, 131.5, 129.1, 128.8, 128.3, 128.3, 128.2, 128.0, 123.6, 79.2, 51.0, 37.4. mp 216–218 °C. HRMS (ESI-TOF) *m*/*z*: [M + H]^+^ calcd for C_23_H_19_N_2_O_3_, 370.1317; found, 370.1403.

#### (±)-4-(*trans*-2,5-Diphenyl-3,4-dihydro-2*H*-pyrrole-3-carbonyl)benzonitrile (**3d**)

The product was obtained as an off-white solid in 37% yield (19.5 mg, 0.056 mmol). ^1^H NMR (400 MHz, CDCl_3_): δ 7.98 (d, *J* = 7.3 Hz, 2H), 7.87 (d, *J* = 8.4 Hz, 2H), 7.71 (d, *J* = 8.3 Hz, 2H), 7.55–7.42 (m, 3H), 7.39–7.29 (m, 3H), 7.23–7.17 (m, 2H), 5.52 (d, *J* = 5.9 Hz, 1H), 4.17 (dt, *J* = 9.5, 6.5 Hz, 1H), 3.66 (dd, *J* = 17.1, 6.4 Hz, 1H), 3.54 (ddd, *J* = 17.2, 9.6, 1.4 Hz, 1H). ^13^C{^1^H} NMR (100 MHz, CDCl_3_): δ 198.2, 171.9, 142.0, 139.1, 133.1, 132.5, 131.5, 129.2, 129.0, 128.7, 128.3, 128.0, 126.9, 117.8, 116.8, 79.5, 54.0, 39.5. mp 162.1–163.0 °C. HRMS (ESI-TOF) *m*/*z*: [M + H]^+^ calcd for C_24_H_19_N_2_O, 351.1497; found, 351.1511.

#### (±)-4-(*cis*-2,5-Diphenyl-3,4-dihydro-2*H*-pyrrole-3-carbonyl)benzonitrile (**3d**)

The product was obtained as an off-white solid in 31% yield (16.4 mg, 0.047 mmol). ^1^H NMR (400 MHz, CDCl_3_): δ 8.09–7.94 (m, 2H), 7.74–7.59 (m, 4H), 7.55–7.41 (m, 3H), 7.10–6.94 (m, 3H), 6.85–6.66 (m, 2H), 5.87 (dd, *J* = 9.6, 1.8 Hz, 1H), 4.72 (td, *J* = 9.5, 7.1 Hz, 1H), 4.02 (ddd, *J* = 17.5, 7.1, 2.3 Hz, 1H), 3.27 (dd, *J* = 17.5, 9.3 Hz, 1H). ^13^C{^1^H} NMR (100 MHz, CDCl_3_): δ 197.2, 173.4, 140.6, 137.4, 133.4, 132.2, 131.4, 128.7, 128.4, 128.2, 128.1, 128.0, 127.8, 117.9, 115.9, 79.0, 50.6, 37.2. mp 181.5–182.6 °C. HRMS (ESI-TOF) *m*/*z*: [M + H]^+^ calcd for C_24_H_19_N_2_O, 351.1497; found, 351.1503.

#### (±)-[1,1′-Biphenyl]-4-yl(*trans*-2,5-diphenyl-3,4-dihydro-2*H*-pyrrol-3-yl)methanone (**3e**)

The product was obtained as an off-white solid in 39% yield (23.6 mg, 0.059 mmol). ^1^H NMR (400 MHz, CDCl_3_): δ 7.98 (d, *J* = 6.8 Hz, 2H), 7.90 (d, *J* = 8.2 Hz, 2H), 7.69–7.58 (m, 4H), 7.50–7.38 (m, 6H), 7.37–7.23 (m, 5H), 5.64 (d, *J* = 5.5 Hz, 1H), 4.21 (dt, *J* = 9.5, 6.5 Hz, 1H), 3.63 (dd, *J* = 17.1, 6.7 Hz, 1H), 3.54 (ddd, *J* = 17.1, 9.7, 1.8 Hz, 1H). ^13^C{^1^H} NMR (100 MHz, CDCl_3_): δ 199.2, 171.6, 146.1, 142.9, 139.7, 134.9, 133.7, 131.0, 129.5, 129.0, 128.8, 128.6, 128.4, 128.1, 127.6, 127.3,127.3, 126.9, 79.7, 53.7, 40.1. mp 173.6–175.2 °C. HRMS (ESI-TOF) *m*/*z*: [M + H]^+^ calcd for C_29_H_24_NO, 402.1858; found, 402.1858.

#### (±)-[1,1′-Biphenyl]-4-yl(*cis*-2,5-diphenyl-3,4-dihydro-2*H*-pyrrol-3-yl)methanone (**3e**)

The product was obtained as an off-white, yellow solid in 15% yield (12.2 mg, 0.030 mmol). ^1^H NMR (400 MHz, CDCl_3_): δ 8.05 (d, *J* = 6.6 Hz, 2H), 7.76 (d, *J* = 8.3 Hz, 2H), 7.62 (dd, *J* = 11.0, 7.9 Hz, 4H), 7.57–7.45 (m, 5H), 7.45–7.34 (m, 1H), 7.13–6.94 (m, 3H), 6.82 (dd, *J* = 7.5, 1.4 Hz, 2H), 5.96 (dd, *J* = 9.5, 1.6 Hz, 1H), 4.79 (dd, *J* = 17.7, 9.2 Hz, 1H), 4.06 (ddd, *J* = 17.5, 8.0, 2.0 Hz, 1H), 3.27 (dd, *J* = 17.5, 9.3 Hz, 1H). ^13^C{^1^H} NMR (100 MHz, CDCl_3_): δ 197.4, 174.0, 145.5, 139.8, 137.2, 136.2, 133.4, 131.4, 129.0, 128.7, 128.7, 128.3, 128.3, 127.9, 127.9, 127.6, 127.2, 127.0, 78.9, 50.4, 37.1. mp 171.7–173.9 °C. HRMS (ESI-TOF) *m*/*z*: [M + H]^+^ calcd for C_29_H_24_NO, 402.1858; found, 402.1865.

#### (±)-(*trans*-2,5-Diphenyl-3,4-dihydro-2*H*-pyrrol-3-yl)(4-fluorophenyl)methanone (**3f**)

The product was obtained as a brown oil in 40% yield (20.7 mg, 0.060 mmol). ^1^H NMR (400 MHz, CDCl_3_): δ 7.96 (dd, *J* = 7.9, 1.2 Hz, 2H), 7.87–7.78 (m, 2H), 7.53–7.41 (m, 3H), 7.36–7.26 (m, 3H), 7.24–7.19 (m, 2H), 7.14–7.04 (m, 2H), 5.55 (d, *J* = 6.0 Hz, 1H), 4.14 (dt, *J* = 9.7, 6.6 Hz, 1H), 3.61 (ddd, *J* = 17.1, 6.8, 1.3 Hz, 1H), 3.50 (ddd, *J* = 17.1, 9.7, 2.0 Hz, 1H). ^13^C{^1^H} NMR (100 MHz, CDCl_3_): δ 198.0, 171.6, 166.0 (d, *J* = 255.8 Hz), 142.7, 133.5, 132.6 (d, *J* = 2.9 Hz), 131.5 (d, *J* = 9.3 Hz), 131.1, 128.8, 128.6, 128.1, 127.7, 126.9, 115.8 (d, *J* = 21.9 Hz), 79.8, 53.7, 39.9. ^19^F NMR (376 MHz, CDCl_3_): δ −104.2. HRMS (ESI-TOF) *m*/*z*: [M + H]^+^ calcd for C_23_H_19_FNO, 344.1451; found, 344.1461.

#### (±)-(*cis*-2,5-Diphenyl-3,4-dihydro-2*H*-pyrrol-3-yl)(4-fluorophenyl)methanone (**3f**)

The product was obtained as an orange solid in 36% yield (18.7 mg, 0.054 mmol). ^1^H NMR (400 MHz, CDCl_3_): δ 8.02 (d, *J* = 6.6 Hz, 2H), 7.74–7.61 (m, 2H), 7.55–7.44 (m, 3H), 7.08–6.97 (m, 5H), 6.78 (dd, *J* = 7.5, 1.6 Hz, 2H), 5.89 (dd, *J* = 9.5, 1.5 Hz, 1H), 4.70 (dd, *J* = 17.3, 9.4 Hz, 1H), 4.02 (ddd, *J* = 17.4, 7.7, 2.0 Hz, 1H), 3.24 (dd, *J* = 17.5, 9.3 Hz, 1H). ^13^C{^1^H} NMR (100 MHz, CDCl_3_): δ 196.4, 173.6, 165.5 (d, *J* = 254.7 Hz), 137.4, 133.9 (d, *J* = 3.1 Hz), 133.6, 131.2, 130.7 (d, *J* = 9.0 Hz), 128.6, 128.2, 127.9, 127.9, 127.6, 115.5 (d, *J* = 21.8 Hz), 79.2, 50.35, 37.2. ^19^F NMR (376 MHz, CDCl_3_): δ −105.3. mp 141.9–143.9 °C. HRMS (ESI-TOF) *m*/*z*: [M + H]^+^ calcd for C_23_H_19_FNO, 344.1451; found, 344.1458.

#### (±)-(4-Bromophenyl)(*trans*-2,5-diphenyl-3,4-dihydro-2*H*-pyrrol-3-yl)methanone (**3g**)

The product was obtained as a yellow solid in 26% yield (15.7 mg, 0.039 mmol). ^1^H NMR (400 MHz, CDCl_3_): δ 8.00–7.92 (m, 2H), 7.69–7.63 (m, 2H), 7.61–7.53 (m, 2H), 7.52–7.41 (m, 3H), 7.38–7.26 (m, 3H), 7.21 (m, 2H), 5.55 (d, *J* = 5.9 Hz, 1H), 4.12 (dt, *J* = 9.7, 6.5 Hz, 1H), 3.61 (ddd, *J* = 17.1, 6.8, 1.4 Hz, 1H), 3.50 (ddd, *J* = 17.1, 9.7, 2.1 Hz, 1H). ^13^C{^1^H} NMR (100 MHz, CDCl_3_): δ 198.7, 171.7, 142.8, 135.0, 133.7, 132.1, 131.2, 130.5, 129.0, 128.9, 128.7, 128.2, 127.8, 127.0, 79.9, 53.9, 40.0. mp 168–170 °C. HRMS (ESI-TOF) *m*/*z*: [M + H]^+^ calcd for C_23_H_19_BrNO, 404.0645; found, 404.0655.

#### (±)-(4-Bromophenyl)(*cis*-2,5-diphenyl-3,4-dihydro-2*H*-pyrrol-3-yl)methanone (**3g**)

The product was obtained as a yellow solid in 35% yield (21.4 mg, 0.053 mmol). ^1^H NMR (400 MHz, CDCl_3_): δ 8.00 (m, 2H), 7.59–7.36 (m, 7H), 7.12–6.96 (m, 3H), 6.77 (m, 2H), 5.89 (d, *J* = 9.4 Hz, 1H), 4.67 (dd, *J* = 17.4, 9.3 Hz, 1H), 3.99 (ddd, *J* = 17.5, 7.8, 2.2 Hz, 1H), 3.22 (dd, *J* = 17.5, 9.3 Hz, 1H). ^13^C{^1^H} NMR (100 MHz, CDCl_3_): δ 197.2, 173.7, 137.5, 136.3, 133.8, 131.8, 131.3, 129.7, 128.8, 128.2, 128.1, 128.0, 127.8, 79.3, 50.5, 37.3 (one quaternary carbon missing/superimposed). mp 114–116 °C. HRMS (ESI-TOF) *m*/*z*: [M + H]^+^ calcd for C_23_H_19_BrNO, 404.0645; found, 404.0654.

#### (±)-(*trans*-2,5-Diphenyl-3,4-dihydro-2*H*-pyrrol-3-yl)(naphthalen-2-yl)methanone (**3h**)

The product was obtained as a yellow oil in 30% yield (17.0 mg, 0.045 mmol). ^1^H NMR (400 MHz, CDCl_3_): δ 8.15 (s, 1H), 8.00 (m, 3H), 7.87 (dd, *J* = 8.3, 3.5 Hz, 2H), 7.73 (d, *J* = 8.1 Hz, 1H), 7.60 (t, *J* = 7.5 Hz, 1H), 7.56–7.42 (m, 4H), 7.33 (m, 3H), 7.26 (m, 2H), 5.62 (d, *J* = 5.8 Hz, 1H), 4.35 (dt, *J* = 9.6, 6.5 Hz, 1H), 3.73 (dd, *J* = 17.0, 6.2 Hz, 1H), 3.57 (ddd, *J* = 17.1, 9.7, 1.8 Hz, 1H). ^13^C{^1^H} NMR (100 MHz, CDCl_3_): δ 199.4, 172.0, 143.0, 135.8, 133.7, 133.6, 132.5, 131.3, 131.2, 129.8, 128.9, 128.9, 128.7, 128.7, 128.3, 127.9, 127.8, 127.2, 127.0, 124.4, 80.1, 54.0, 40.0. HRMS (ESI-TOF) *m*/*z*: [M + H]^+^ calcd for C_27_H_22_NO, 376.1696; found, 376.1713.

#### (±)-(*cis*-2,5-Diphenyl-3,4-dihydro-2*H*-pyrrol-3-yl)(naphthalen-2-yl)methanone (**3h**)

The product was obtained as a yellow solid in 22% yield (12.2 mg, 0.033 mmol). ^1^H NMR (400 MHz, CDCl_3_): δ 8.30 (s, 1H), 8.06 (d, *J* = 6.6 Hz, 2H), 7.95 (d, *J* = 7.8 Hz, 1H), 7.85 (d, *J* = 7.8 Hz, 1H), 7.78 (d, *J* = 8.6 Hz, 1H), 7.58 (m, 6H), 7.03–6.89 (m, 3H), 6.77 (d, *J* = 7.1 Hz, 2H), 6.02 (d, *J* = 9.4 Hz, 1H), 4.93 (dd, *J* = 17.9, 9.1 Hz, 1H), 4.08 (ddd, *J* = 17.6, 8.0, 1.7 Hz, 1H), 3.29 (dd, *J* = 17.5, 9.3 Hz, 1H). ^13^C{^1^H} NMR (100 MHz, CDCl_3_): δ 197.8, 174.1, 137.3, 135.6, 135.0, 133.6, 132.5, 131.5, 129.8, 129.7, 128.8, 128.6, 128.5, 128.4, 128.0, 128.0, 127.9, 127.7, 126.9, 124.1, 79.2, 50.6, 37.3. mp 160–162 °C. HRMS (ESI-TOF) *m*/*z*: [M + H]^+^ calcd for C_27_H_22_NO, 376.1696; found, 376.1708.

#### (±)-Benzo[*d*][1,3]dioxol-5-yl(*trans*-2,5-diphenyl-3,4-dihydro-2*H*-pyrrol-3-yl)methanone (**3i**)

The product was obtained as a brown oil in 23% yield (12.8 mg, 0.035 mmol). ^1^H NMR (400 MHz, CDCl_3_): δ 7.98 (d, *J* = 7.1 Hz, 2H), 7.53–7.40 (m, 3H), 7.39–7.20 (m, 7H), 6.77 (d, *J* = 8.2 Hz, 1H), 6.05 (s, 2H), 5.58 (d, *J* = 5.9 Hz, 1H), 4.21–3.96 (m, 1H), 3.60 (dd, *J* = 17.0, 6.5 Hz, 1H), 3.50 (ddd, *J* = 17.2, 9.7, 1.9 Hz, 1H). ^13^C{^1^H} NMR (100 MHz, CDCl_3_): δ 197.6, 171.7, 152.2, 148.4, 142.8, 133.5, 131.1, 130.3, 128.7, 128.6, 128.1, 127.6, 126.9, 125.3, 108.5, 107.8, 102.0, 79.7, 53.5, 40.2. HRMS (ESI-TOF) *m*/*z*: [M + H]^+^ calcd for C_24_H_20_NO_3_, 370.1443; found, 370.1442.

#### (±)-Benzo[*d*][1,3]dioxol-5-yl(*cis*-2,5-diphenyl-3,4-dihydro-2*H*-pyrrol-3-yl)methanone (**3i**)

The product was obtained as a brown oil in 19% yield (10.5 mg, 0.028 mmol). ^1^H NMR (400 MHz, CDCl_3_): δ 8.06 (d, *J* = 6.8 Hz, 2H), 7.60–7.45 (m, 3H), 7.41 (dd, *J* = 8.2, 1.6 Hz, 1H), 7.11–7.01 (m, 4H), 6.85–6.79 (m, 3H), 6.03 (s, 2H), 5.91 (dd, *J* = 9.5, 1.5 Hz, 1H), 4.67 (q, *J* = 9.1 Hz, 1H), 4.04 (ddd, *J* = 17.6, 8.1, 1.8 Hz, 1H), 3.25 (dd, *J* = 17.6, 9.2 Hz, 1H).^13^C{^1^H} NMR (100 MHz, CDCl_3_): δ 195.4, 174.5, 151.7, 148.1, 136.9, 132.9, 132.2, 131.8, 128.8, 128.5, 1278.0, 127.9, 127.7, 124.5, 107.9, 107.7, 101.8, 78.5, 50.0, 37.1. HRMS (ESI-TOF) *m*/*z*: [M + H]^+^ calcd for C_24_H_20_NO_3_, 370.1443; found, 370.1442.

#### (±)-(*trans*-2,5-Diphenyl-3,4-dihydro-2*H*-pyrrol-3-yl)(furan-2-yl)methanone (**3j**)

The product was obtained as a colorless oil in 41% yield (19.5 mg, 0.062 mmol). ^1^H NMR (400 MHz, CDCl_3_): δ 8.04–7.86 (m, 2H), 7.57 (d, *J* = 1.0 Hz, 1H), 7.52–7.41 (m, 3H), 7.29 (m, 5H), 7.00 (d, *J* = 3.6 Hz, 1H), 6.50 (dd, *J* = 3.6, 1.7 Hz, 1H), 5.60 (d, *J* = 6.4 Hz, 1H), 3.96 (dt, *J* = 9.7, 7.0 Hz, 1H), 3.58 (ddd, *J* = 17.1, 7.3, 1.6 Hz, 1H), 3.49 (ddd, *J* = 17.1, 9.7, 2.0 Hz, 1H). ^13^C{^1^H} NMR (100 MHz, CDCl_3_): δ 188.5, 171.8, 152.4, 147.2, 143.0, 133.7, 131.2, 128.8, 128.7, 128.2, 127.6, 126.9, 118.7, 112.6, 79.6, 54.5, 39.7. HRMS (ESI-TOF) *m*/*z*: [M + H]^+^ calcd for C_21_H_18_NO_2_, 316.1332; found, 316.1342.

#### (±)-(*cis*-2,5-Diphenyl-3,4-dihydro-2*H*-pyrrol-3-yl)(furan-2-yl)methanone (**3j**)

The product was obtained as a yellow oil in 39% yield (18.4 mg, 0.058 mmol). ^1^H NMR (400 MHz, CDCl_3_): δ 8.12–7.91 (m, 2H), 7.59–7.41 (m, 4H), 7.17–7.02 (m, 3H), 6.96–6.76 (m, 3H), 6.42 (dd, *J* = 3.4, 1.6 Hz, 1H), 5.97 (dd, *J* = 9.7, 1.7 Hz, 1H), 4.56 (dd, *J* = 17.5, 9.6 Hz, 1H), 3.94 (ddd, *J* = 17.4, 7.8, 2.2 Hz, 1H), 3.16 (dd, *J* = 17.4, 9.6 Hz, 1H). ^13^C{^1^H} NMR (100 MHz, CDCl_3_): δ 187.2, 173.4, 153.1, 145.7, 138.0, 133.9, 131.2, 128.7, 128.2, 128.1, 127.6, 127.6, 116.7, 112.6, 79.3, 50.8, 36.3. HRMS (ESI-TOF) *m*/*z*: [M + H]^+^ calcd for C_21_H_18_NO_2_, 316.1332; found, 316.1348.

#### (±)-(*trans*-2,5-Diphenyl-3,4-dihydro-2*H*-pyrrol-3-yl)(thiophen-2-yl)methanone (**3k**)

The product was obtained as a yellow oil in 49% yield (24.6 mg, 0.074 mmol). ^1^H NMR (400 MHz, CDCl_3_): δ 7.98 (d, *J* = 6.9 Hz, 2H), 7.67 (dd, *J* = 4.9, 1.0 Hz, 1H), 7.52–7.42 (m, 3H), 7.39–7.28 (m, 4H), 7.28–7.21 (m, 2H), 7.05 (dd, *J* = 4.9, 3.9 Hz, 1H), 5.59 (d, *J* = 4.9 Hz, 1H), 4.02 (dt, *J* = 9.7, 6.8 Hz, 1H), 3.65 (ddd, *J* = 17.3, 7.1, 1.3 Hz, 1H), 3.52 (ddd, *J* = 17.1, 9.7, 2.0 Hz, 1H). ^13^C{^1^H} NMR (100 MHz, CDCl_3_): δ 192.6, 171.6, 144.0, 142.9, 134.7, 133.67, 132.9, 131.0, 128.7, 128.6, 128.3, 128.1, 127.6, 126.9, 80.2, 55.3, 40.3. HRMS (ESI-TOF) *m*/*z*: [M + H]^+^ calcd for C_21_H_18_NOS, 332.1109; found, 332.1112.

#### (±)-(*cis*-2,5-Diphenyl-3,4-dihydro-2*H*-pyrrol-3-yl)(thiophen-2-yl)methanone (**3k**)

The product was obtained as a yellow oil in 34% yield (19 mg, 0.051 mmol). ^1^H NMR (400 MHz, CDCl_3_): δ 8.08–7.95 (m, 2H), 7.64 (dd, *J* = 3.8, 0.9 Hz, 1H), 7.60–7.43 (m, 4H), 7.37 (ddd, *J* = 21.7, 12.3, 6.5 Hz, 1H), 7.10–7.04 (m, 3H), 6.89–6.83 (m, 2H), 5.94 (dd, *J* = 9.6, 1.6 Hz, 1H), 4.59 (dd, *J* = 17.3, 9.4 Hz, 1H), 3.97 (ddd, *J* = 17.5, 7.8, 2.1 Hz, 1H), 3.24 (dd, *J* = 17.5, 9.3 Hz, 1H). ^13^C{^1^H} NMR (100 MHz, CDCl_3_): δ 190.4, 173.6, 144.8, 137.3, 133.7, 133.7, 131.7, 131.2, 128.6, 128.2, 127.9, 127.9, 127.6, 79.7, 51.8, 37.1. HRMS (ESI-TOF) *m*/*z*: [M + H]^+^ calcd for C_21_H_18_NOS, 332.1109; found, 332.1122.

#### *tert*-Butyl (3-(*trans*-2,5-Diphenyl-3,4-dihydro-2*H*-pyrrole-3-carbonyl)phenyl)carbamate (**3l**)

The product was obtained as a yellow oil in 52% yield (34.1 mg, 0.077 mmol). ^1^H NMR (400 MHz, MeOD): δ 8.03 (br, 1H), 7.98–7.88 (m, 2H), 7.61 (dd, *J* = 8.1, 1.0 Hz, 1H), 7.56–7.44 (m, 4H), 7.40–7.27 (m, 4H), 7.26–7.21 (m, 2H), 5.50 (d, *J* = 5.9 Hz, 1H), 4.29 (dt, *J* = 9.7, 6.4 Hz, 1H), 3.70 (ddd, *J* = 17.5, 9.7, 2.1 Hz, 1H), 3.52 (ddd, *J* = 17.6, 6.7, 1.3 Hz, 1H), 3.35 (br, 1H), 1.51 (s, 9H).^13^C{^1^H} NMR (100 MHz, MeOD): δ 199.7, 173.6, 153.7, 142.3, 140.0, 136.7, 133.1, 131.1, 128.8, 128.5, 128.4, 127.8, 127.4, 126.7, 123.2, 122.7, 118.3, 79.8, 79.2, 53.2, 39.8, 27.3. HRMS (ESI-TOF) *m*/*z*: [M + H]^+^ calcd for C_28_H_29_N_2_O_3_, 441.2178; found, 441.2184.

#### *tert*-Butyl (3-(*cis*-2,5-Diphenyl-3,4-dihydro-2*H*-pyrrole-3-carbonyl)phenyl)carbamate (**3l**)

The product was obtained as a yellow oil in 41% yield (26.8 mg, 0.061 mmol). ^1^H NMR (400 MHz, CDCl_3_): δ 8.01 (d, *J* = 7.1 Hz, 2H), 7.69 (br, 1H), 7.61–7.54 (m, 1H), 7.53–7.44 (m, 3H), 7.36–7.31 (m, 1H), 7.30–7.23 (m, 1H), 7.06–6.97 (m, 3H), 6.80 (dd, *J* = 7.6, 1.7 Hz, 2H), 6.67 (br, 1H, N–H), 5.96 (d, *J* = 9.6 Hz, 1H), 4.73 (dd, *J* = 17.9, 9.2 Hz, 1H), 4.00 (ddd, *J* = 17.5, 8.1, 2.0 Hz, 1H), 3.21 (dd, *J* = 17.5, 9.3 Hz, 1H), 1.53 (s, 9H). ^13^C{^1^H} NMR (100 MHz, CDCl_3_): δ 197.7, 173.8, 152.6, 138.8, 138.18, 137.3, 133.5, 131.3, 129.1, 128.7, 128.3, 128.0, 128.0, 127.9, 127.5, 122.6, 117.9, 80.9, 78.9, 50.5, 37.0, 28.3. HRMS (ESI-TOF) *m*/*z*: [M + H]^+^ calcd for C_28_H_29_N_2_O_3_, 441.2178; found, 441.2191.

#### (±)-(*trans*-2,5-Diphenyl-3,4-dihydro-2*H*-pyrrol-3-yl)(2-methoxyphenyl)methanone (**3m**)

The product was obtained as a yellow oil in 29% yield (15.5 mg, 0.044 mmol). ^1^H NMR (400 MHz, CDCl_3_): δ 7.92–7.81 (m, 2H), 7.55 (dt, *J* = 16.2, 8.1 Hz, 1H), 7.42–7.30 (m, 4H), 7.24–7.12 (m, 3H), 7.10–7.03 (m, 2H), 6.94 (t, *J* = 7.4, 1H), 6.78 (d, *J* = 8.4 Hz, 1H), 5.48 (m, 1H), 4.25 (dt, *J* = 9.8, 6.6 Hz, 1H), 3.53–3.44 (dd + s (OMe), 4H), 3.37 (ddd, *J* = 17.2, 9.8, 2.2 Hz, 1H). ^13^C{^1^H} NMR (100 MHz, CDCl_3_): δ 202.6, 172.0, 158.2, 143.5, 133.7, 131.0, 130.6, 128.5 (2C), 128.4, 128.2, 128.1, 127.1, 126.8, 120.8, 111.4, 79.3, 57.6, 55.1, 39.3. mp 87–89 °C. HRMS (ESI-TOF) *m*/*z*: [M + H]^+^ calcd for C_24_H_22_NO_2_, 356.1645; found, 356.1657.

#### (±)-(*cis*-2,5-Diphenyl-3,4-dihydro-2*H*-pyrrol-3-yl)(2-methoxyphenyl)methanone (**3m**)

The product was obtained as a yellow oil in 19% yield (9.9 mg, 0.028 mmol). ^1^H NMR (400 MHz, CDCl_3_): δ 8.00 (dd, *J* = 7.6, 1.4 Hz, 2H), 7.87 (d, *J* = 7.5 Hz, 1H), 7.55–7.43 (m, 4H), 7.40–7.33 (m, 1H), 7.09–6.95 (m, 3H), 6.91 (d, *J* = 8.3 Hz, 1H), 6.81 (dd, *J* = 7.5, 1.6 Hz, 2H), 6.75 (t, *J* = 7.5 Hz, 1H), 5.85 (dd, *J* = 9.6, 2.0 Hz, 1H), 4.92 (dd, *J* = 18.1, 9.4 Hz, 1H), 3.97 (s, 3H), 3.96–3.89 (m, 1H), 3.22 (dd, *J* = 17.5, 9.5 Hz, 1H). ^13^C{^1^H} NMR (100 MHz, CDCl_3_): δ 199.5, 157.9, 138.6, 134.0, 133.4, 133.1, 130.9, 130.8, 128.9, 128.6, 128.1, 127.9, 127.9, 127.8, 127.3, 120.6, 111.0, 78.3, 55.6, 54.8, 37.3. HRMS (ESI-TOF) *m*/*z*: [M + H]^+^ calcd for C_24_H_22_NO_2_, 356.16451; found, 356.1653. Note: this compound showed enhanced instability observed by TLC before and after NMR analysis.

#### (±)-1-(*trans*-2,5-Diphenyl-3,4-dihydro-2*H*-pyrrol-3-yl)ethan-1-one (**3n**)

The product was obtained as a yellow oil in 26% yield (10.4 mg, 0.040 mmol). ^1^H NMR (400 MHz, CDCl_3_): δ 7.86 (m, 2H), 7.45–7.34 (m, 3H), 7.30 (m, 2H), 7.26–7.18 (m, 3H), 5.36–5.30 (m, 1H), 3.45–3.21 (m, 3H), 2.15 (s, 3H). ^13^C{^1^H} NMR (100 MHz, CDCl_3_): δ 207.2, 171.8, 143.1, 133.6, 131.1, 128.8, 128.6, 128.0, 127.6, 126.7, 78.9, 59.3, 38.2, 29.9. HRMS (ESI-TOF) *m*/*z*: [M + H]^+^ calcd for C_18_H_17_NO + H^+^], 264.13829; found, 264.1393.

#### (±)-1-(*cis*-2,5-Diphenyl-3,4-dihydro-2*H*-pyrrol-3-yl)ethan-1-one (**3n**)

The product was obtained as a white solid in 27% yield (10.5 mg, 0.040 mmol). ^1^H NMR (400 MHz, CDCl_3_): δ 8.04–7.83 (m, 2H), 7.54–7.39 (m, 3H), 7.34–7.21 (m, 3H), 7.15 (d, *J* = 6.8 Hz, 2H), 5.77 (d, *J* = 9.4 Hz, 1H), 3.89 (m, 1H), 3.74 (ddd, *J* = 17.3, 6.6, 2.1 Hz, 1H), 3.07 (dd, *J* = 17.3, 9.3 Hz, 1H), 1.70 (s, 3H). ^13^C{^1^H} NMR (100 MHz, CDCl_3_): δ 206.7, 173.3, 138.6, 133.9, 131.1, 128.7, 128.6, 128.1, 128.1 (2C), 78.4, 55.9, 37.3, 30.45. mp 108–110 °C. HRMS (ESI-TOF) *m*/*z*: [M + H]^+^ calcd for C_18_H_17_NO + H^+^], 264.13829; found, 264.1395.

#### (±)-(*trans*-5-(4-Chlorophenyl)-2-phenyl-3,4-dihydro-2*H*-pyrrol-3-yl)(phenyl)methanone (**3o**)

The product was obtained as a white solid in 37% yield (20.0 mg, 0.055 mmol). ^1^H NMR (400 MHz, CDCl_3_): δ 7.89 (d, *J* = 8.4 Hz, 2H), 7.82 (d, *J* = 7.5 Hz, 2H), 7.57 (t, *J* = 7.3 Hz, 1H), 7.42 (m, 4H), 7.36–7.28 (m, 3H), 7.21 (d, *J* = 6.9 Hz, 2H), 5.60 (d, *J* = 5.5 Hz, 1H), 4.19 (m, 1H), 3.57 (dd, *J* = 17.0, 6.5 Hz, 1H), 3.48 (dd, *J* = 17.0, 9.7 Hz, 1H). ^13^C{^1^H} NMR (100 MHz, CDCl_3_): δ 199.5, 170.6, 142.7, 137.3, 136.2, 133.7, 132.2, 129.6, 129.0, 129.0, 128.9, 128.8, 127.8, 127.0, 79.8, 53.7, 40.0. mp 133–134 °C. HRMS (ESI-TOF) *m*/*z*: [M + H]^+^ calcd for C_23_H_19_ClNO, 360.11497; found, 360.1157.

#### (±)-(*cis*-5-(4-Chlorophenyl)-2-phenyl-3,4-dihydro-2*H*-pyrrol-3-yl)(phenyl)methanone (**3o**)

The product was obtained as an orange solid in 25% yield (13.6 mg, 0.038 mmol). ^1^H NMR (400 MHz, CDCl_3_): δ 7.96 (d, *J* = 6.6 Hz, 2H), 7.66 (d, *J* = 6.3 Hz, 2H), 7.47 (m, 3H), 7.37 (m, 2H), 7.01 (m, 3H), 6.76 (d, *J* = 5.5 Hz, 1H), 5.90 (d, *J* = 6.5 Hz, 1H), 4.76 (d, *J* = 6.1 Hz, 1H), 4.00 (m, 1H), 3.21 (m, 1H). ^13^C{^1^H} NMR (100 MHz, CDCl_3_): δ 197.9, 172.8, 137.5, 137.5, 137.3, 133.1, 132.2, 129.6, 129.0, 128.6, 128.2, 128.0 (2C), 127.8, 79.2, 50.5, 37.2. mp 146–147 °C. HRMS (ESI-TOF) *m*/*z*: [M + H]^+^ calcd for C_23_H_19_ClNO, 360.11497; found, 360.1165.

#### (±)-(*trans*-5-(4-Methoxyphenyl)-2-phenyl-3,4-dihydro-2*H*-pyrrol-3-yl)(phenyl)methanone (**3p**)

The product was obtained as a yellow solid in 39% yield (20.5 mg, 0.058 mmol). ^1^H NMR (400 MHz, CDCl_3_): δ 7.90 (d, *J* = 8.7 Hz, 2H), 7.82 (d, *J* = 7.5 Hz, 2H), 7.56 (t, *J* = 7.4 Hz, 1H), 7.42 (m, 2H), 7.36–7.17 (m, 5H), 6.94 (d, *J* = 8.8 Hz, 2H), 5.57 (d, *J* = 5.8 Hz, 1H), 4.15 (dt, *J* = 9.4, 6.6 Hz, 1H), 3.61–3.36 (m, 1H). ^13^C{^1^H} NMR (100 MHz, CDCl_3_): δ 199.8, 170.8, 161.8, 143.2, 136.3, 133.4, 129.8, 128.8, 128.7, 128.7, 127.5, 126.9, 126.5, 113.9, 79.5, 55.4, 53.8, 39.9. mp 117–120 °C. HRMS (ESI-TOF) *m*/*z*: [M + H]^+^ calcd for C_24_H_22_NO_2_, 356.1645; found, 356.1654.

#### (±)-(*cis*-5-(4-Methoxyphenyl)-2-phenyl-3,4-dihydro-2*H*-pyrrol-3-yl)(phenyl)methanone (**3p**)

The product was obtained as a yellow in 20% yield (10.8 mg, 0.030 mmol). ^1^H NMR (400 MHz, CDCl_3_): δ 7.96 (d, *J* = 8.8 Hz, 2H), 7.73–7.63 (m, 2H), 7.49 (t, *J* = 7.4 Hz, 1H), 7.36 (t, *J* = 7.7 Hz, 2H), 7.05–6.96 (m, 5H), 6.75 (m, 2H), 5.87 (dd, *J* = 9.6, 1.8 Hz, 1H), 4.73 (dd, *J* = 17.8, 9.4 Hz, 1H), 3.97 (ddd, *J* = 17.3, 8.2, 2.2 Hz, 1H), 3.88 (s, 3H), 3.19 (dd, *J* = 17.3, 9.3 Hz, 1H). ^13^C{^1^H} NMR (100 MHz, CDCl_3_): δ 198.1, 172.8, 161.9, 137.7, 137.5, 132.8, 129.8, 128.4, 128.1, 127.9, 127.8, 127.4, 126.6, 113.9, 79.1, 55.4, 50.6, 36.9. mp 130–133 °C. HRMS (ESI-TOF) *m*/*z*: [M + H]^+^ calcd for C_24_H_22_NO_2_, 356.16451; found, 356.1651.

#### (±)-(*trans*-5-(4-Fluorophenyl)-2-phenyl-3,4-dihydro-2*H*-pyrrol-3-yl)(phenyl)methanone (**3q**)

The product was obtained as a colorless oil in 46% yield (23.6 mg, 0.069 mmol). ^1^H NMR (400 MHz, CDCl_3_): δ 7.98–7.91 (m, 2H), 7.86–7.79 (m, 2H), 7.57 (t, *J* = 7.4 Hz, 1H), 7.42 (t, *J* = 7.8 Hz, 2H), 7.36–7.26 (m, 3H), 7.23–7.18 (m, 2H), 7.16–7.07 (m, 2H), 5.58 (d, *J* = 5.8 Hz, 1H), 4.18 (dt, *J* = 9.7, 6.5 Hz, 1H), 3.56 (ddd, *J* = 17.0, 6.8, 1.5 Hz, 1H), 3.48 (ddd, *J* = 17.1, 9.7, 2.1 Hz, 1H). ^13^C{^1^H} NMR (100 MHz, CDCl_3_): δ 199.6, 170.4, 164.6 (d, *J* = 251.3 Hz), 142.9, 136.3, 133.6, 130.3 (d, *J* = 8.7 Hz), 130.2 (d, *J* = 3.4 Hz), 129.0, 128.9, 128.8, 127.7, 127.0, 115.7 (d, *J* = 21.7 Hz), 79.8, 53.8, 40.1. ^19^F NMR (376 MHz, CDCl_3_): δ −109.08 (s). mp 85–87 °C. HRMS (ESI-TOF) *m*/*z*: [M + H]^+^ calcd for C_23_H_19_FN, 344.1445; found, 344.1464.

#### (±)-(*cis*-5-(4-Fluorophenyl)-2-phenyl-3,4-dihydro-2*H*-pyrrol-3-yl)(phenyl)methanone (**3q**)

The product was obtained as a yellow solid in 30% yield (15.4 mg, 0.044 mmol). ^1^H NMR (400 MHz, CDCl_3_): δ 8.03–7.96 (m, 2H), 7.66 (d, *J* = 7.3 Hz, 2H), 7.49 (t, *J* = 7.4 Hz, 1H), 7.35 (t, *J* = 7.7 Hz, 2H), 7.15 (t, *J* = 8.6 Hz, 2H), 7.05–6.95 (m, 3H), 6.79–6.67 (m, 2H), 5.88 (dd, *J* = 9.6, 1.8 Hz, 1H), 4.75 (dd, *J* = 17.4, 9.4 Hz, 1H), 3.98 (ddd, *J* = 17.4, 7.8, 2.2 Hz, 1H), 3.19 (dd, *J* = 17.4, 9.4 Hz, 1H). ^13^C{^1^H} NMR (100 MHz, CDCl_3_): δ 198.1, 172.4, 164.7 (d, *J* = 251.5 Hz), 137.6, 137.5, 133.0, 130.3 (d, *J* = 8.5 Hz), 130.2 (d, *J* = 3.2 Hz), 128.5, 128.2, 128.0, 128.0, 127.6, 115.8 (d, *J* = 21.9 Hz), 79.4, 50.6, 37.3. ^19^F NMR (376 MHz, CDCl_3_): δ −108.93 (s). mp 126–127 °C. HRMS (ESI-TOF) *m*/*z*: [M + H]^+^ calcd for C_23_H_19_FNO, 344.1445; found, 344.1458.

#### (±)-(*trans*-2-(4-Chlorophenyl)-5-(*p*-tolyl)-3,4-dihydro-2*H*-pyrrol-3-yl)(phenyl)methanone (**3r**)

The product was obtained as a yellow oil in 49% yield (27.6 mg, 0.074 mmol). ^1^H NMR (400 MHz, CDCl_3_): δ 7.79–7.68 (m, 4H), 7.55–7.47 (m, 1H), 7.37 (m, 2H), 7.25–7.14 (m, 4H), 7.11–7.03 (m, 2H), 5.52 (d, *J* = 6.3 Hz, 1H), 4.02 (td, *J* = 8.5, 6.4 Hz, 1H), 3.44 (dd, *J* = 8.6, 1.9 Hz, 2H), 2.33 (3H, s). ^13^C{^1^H} NMR (100 MHz, CDCl_3_): δ 199.6, 171.7, 141.8, 141.6, 136.4, 133.7, 133.3, 130.9, 129.4, 128.9, 128.9, 128.9, 128.4, 128.1, 78.7, 54.0, 40.4, 21.7. HRMS (ESI-TOF) *m*/*z*: [M + H]^+^ calcd for C_24_H_21_ClNO, 374.1306; found, 374.1315.

#### (±)-(*cis*-2-(4-Chlorophenyl)-5-(*p*-tolyl)-3,4-dihydro-2*H*-pyrrol-3-yl)(phenyl)methanone (**3r**)

The product was obtained as a yellow solid in 41% yield (23.1 mg, 0.062 mmol). ^1^H NMR (400 MHz, CDCl_3_): δ 7.88 (d, *J* = 8.1 Hz, 2H), 7.71–7.66 (m, 2H), 7.52 (t, *J* = 7.4 Hz, 1H), 7.39 (t, *J* = 7.7 Hz, 2H), 7.28 (d, *J* = 8.0 Hz, 2H), 6.96 (d, *J* = 8.5 Hz, 2H), 6.73–6.68 (m, 2H), 5.85 (dd, *J* = 9.6, 1.9 Hz, 1H), 4.73 (dd, *J* = 17.6, 9.3 Hz, 1H), 3.96 (ddd, *J* = 17.4, 8.0, 2.2 Hz, 1H), 3.21 (dd, *J* = 17.4, 9.3 Hz, 1H), 2.43 (s, 3H). ^13^C{^1^H} NMR (100 MHz, CDCl_3_): δ 197.9, 173.9, 141.7, 137.4, 136.3, 133.2, 133.1, 130.9, 129.4, 129.2, 128.6, 128.1, 128.1, 128.0, 78.4, 50.3, 37.1, 21.6. mp 133–135 °C. HRMS (ESI-TOF) *m*/*z*: [M + H]^+^ calcd for C_24_H_21_ClNO, 374.1306; found, 374.1323.

#### (±)-Benzyl *trans*-2,5-Diphenyl-3,4-dihydro-2*H*-pyrrole-3-carboxylate (**3s**)

The product was obtained as a colorless oil in 23% yield (12.3 mg, 0.035 mmol). ^1^H NMR (400 MHz, CDCl_3_): δ 7.93 (d, *J* = 6.8 Hz, 2H), 7.53–7.40 (m, 3H), 7.39–7.22 (m, 10H), 5.55 (d, *J* = 6.5 Hz, 1H), 5.25 (d, *J* = 12.3 Hz, 1H), 5.17 (d, *J* = 12.3 Hz, 1H), 3.54–3.37 (m, 2H), 3.23 (dd, *J* = 16.3, 7.5 Hz, 1H). ^13^C{^1^H} NMR (100 MHz, CDCl_3_): δ 173.9, 171.8, 143.0, 135.8, 133.8, 131.2, 128.8, 128.7, 128.7, 128.5, 128.4, 128.1, 127.5, 126.7, 79.7, 67.0, 51.4, 39.6. HRMS (ESI-TOF) *m*/*z*: [M + H]^+^ calcd for C_24_H_22_NO_2_, 356.16451; found, 356.1662. IR (ν_max_): 1730, 1622, 1494, 1448, 1336, 1240, 1232, 1161, 1026, 761, 694 cm^–1^.

#### (±)-Benzyl *cis*-2,5-Diphenyl-3,4-dihydro-2*H*-pyrrole-3-carboxylate (**3s**)

The product was obtained as a colorless oil in 39% yield (20.6 mg, 0.058 mmol). ^1^H NMR (400 MHz, CDCl_3_): δ 8.04–7.86 (m, 2H), 7.54–7.39 (m, 3H), 7.34–7.21 (m, 6H), 7.20–7.14 (m, 2H), 7.11–7.05 (m, 2H), 5.76 (d, *J* = 9.3 Hz, 1H), 4.71 (d, *J* = 12.1 Hz, 1H), 4.35 (d, *J* = 12.1 Hz, 1H), 3.78 (m, 1H), 3.68 (ddd, *J* = 17.1, 6.9, 2.2 Hz, 1H), 3.22 (dd, *J* = 17.1, 9.5 Hz, 1H). ^13^C{^1^H} NMR (100 MHz, CDCl_3_): δ 173.2, 172.1, 138.5, 135.4, 133.8, 131.2, 128.7, 128.6, 128.6, 128.4, 128.3, 128.2, 127.9, 127.9, 78.6, 66.8, 48.2, 38.3. HRMS (ESI-TOF) *m*/*z*: [M + H]^+^ calcd for C_24_H_22_NO_2_, 356.1645; found, 356.1649. IR (ν_max_): 1730, 1622, 1494, 1454, 1340, 1244, 1174, 1163, 1076, 1026, 758, 694 cm^–1^.

#### (±)-2-(4-Isobutylphenyl)propyl (*trans*)-2,5-Diphenyl-3,4-dihydro-2*H*-pyrrole-3-carboxylate (**3t**)

The product was obtained as a colorless oil in 27% yield (17.6 mg, 0.040 mmol)—mixture of two *trans* diastereoisomers at 1:1 ratio (from racemic ibuprofen). ^1^H NMR (400 MHz, CDCl_3_): δ 7.96–7.85 (m, 2H), 7.52–7.40 (m, 3H), 7.35–7.22 (m, 3H), 7.21–7.17 (m, 2H), 7.14–7.04 (m, 4H), 5.48 (m, 1H), 4.36–4.17 (m, 2H), 3.37–3.30 (m, 2H), 3.18–3.06 (m, 2H), 2.44 (d, *J* = 7.1 Hz, 2H), 1.83 (sep, *J* = 6.6 Hz, 1H), 1.29 (d, *J* = 7.0 Hz) and 1.28 (d, *J* = 7.0 Hz) overlapped (3H), 0.88 (d, *J* = 6.6 Hz, 6H). Note: many signals between the *trans*-diastereoisomers are overlapped. ^13^C{^1^H} NMR (100 MHz, CDCl_3_): δ 173.9 and 173.9, 171.7, 143.0, 140.2, 140.1, 133.7, 131.0, 129.3, 128.6, 128.0, 127.3, 127.0, 127.0, 126.5, 79.5 and 79.4, 70.1 and 70.0, 51.1 and 51.0, 45.1, 39.3 and 39.2, 38.6 and 38.6, 30.2, 22.4, 18.3, and 18.1. Note: some signals between the *trans*-diastereoisomers are overlapped. HRMS (ESI-TOF) *m*/*z*: [M + H]^+^ calcd for C_30_H_34_NO_2_, 440.2584; found, 440.2605.

#### (±)-2-(4-Isobutylphenyl)propyl (*cis*)-2,5-Diphenyl-3,4-dihydro-2*H*-pyrrole-3-carboxylate (**3t**)

The product was obtained as a colorless oil in 40% yield (26.4 mg, 0.06 mmol)—mixture of two *cis* diastereoisomers at a 1:1 ratio (from racemic ibuprofen). ^1^H NMR (400 MHz, CDCl_3_): δ 7.92 (m, 2H), 7.55–7.37 (m, 3H), 7.27–7.15 (m, 3H), 7.13–7.02 (m, 4H), 7.01–6.93 (m, 2H), 5.70 (m, 1H), 3.83–3.54 (m, 3H), 3.50 and 3.36 (dd, *J* = 10.7, 7.3 Hz, 1H), 3.16 (m, 1H), 2.79–2.57 (m, 1H), 2.44 and 2.43 overlapped (d, *J* = 7.1, 2H), 1.91–1.75 overlapped (m, 1H), 1.12 and 1.09 overlapped (d, *J* = 7.0 Hz, 3H), 0.89 and 0.89 overlapped (d, *J* = 6.6 Hz, 6H). Note: many signals between the *cis*-diastereoisomers are overlapped. ^13^C{^1^H} NMR (100 MHz, CDCl_3_): δ 173.2 and 173.2, 172.0, 140.27, 140.0 and 140.0, 138.5 and 138.4, 133.71, 131.07, 129.12, 128.60, 128.11, 128.07, 127.7, 127.6, 127.0 and 127.0, 78.5 and 78.4, 69.9 and 69.8, 48.2 and 48.2, 45.1, 38.2 and 38.2, 38.1 and 38.1, 30.2, 22.4, 18.0, and 17.9. Note: some signals between the *cis*-diastereoisomers are overlapped. HRMS (ESI-TOF) *m*/*z*: [M + H]^+^ calcd for C_30_H_34_NO_2_, 440.2584; found, 440.2599.

#### (3a*S*,5*S*,6*R*,6a*S*)-5-((*S*)-2,2-Dimethyl-1,3-dioxolan-4-yl)-2,2-dimethyltetrahydrofuro[2,3-*d*][1,3]dioxol-6-yl (*trans*)-2,5-Diphenyl-3,4-dihydro-2*H*-pyrrole-3-carboxylate (**3u**)

The product was obtained as a yellow oil in 35% yield (26.9 mg, 0.053 mmol)—mixture of two *trans-*diastereoisomers at approximately 1:1 ratio by NMR and GC–MS analyses. ^1^H NMR (400 MHz, CDCl_3_): δ 7.88–7.82 (m, 2H), 7.44–7.34 (m, 3H), 7.31–7.17 (m, 5H), 5.81–5.76 (m, 1H), 5.50 (m, 2H), 5.28 (m, 1H), 4.44 (d, *J* = 3.6 Hz, 1H), 4.17–3.84 (m, 4H), 3.50–3.31 (m, 2H), 3.21–3.11 (m, 1H), 1.47 (s, 3H), 1.34 (m, 3H), 1.28–1.18 (m, 6H). ^13^C{^1^H} NMR (100 MHz, CDCl_3_): δ 173.0 and 172.5, 171.8 and 171.7, 142.8 and 142.7, 133.5, 131.3 and 131.2, 128.8 and 128.7, 128.8, 128.1 and 128.0, 127.7, 126.7 and 126.7, 112.6 and 112.5, 109.7 and 109.6, 105.3 and 105.2, 83.6 and 83.5, (80.1, 80.0, 80.0, 79.5) 2C, 77.0 (overlapped with CDCl_3_), 72.5, 67.6 and 67.5, 51.4 and 50.9, 39.6 and 39.4, (27.1, 27.0, 26.9 (2C), 26.3 (2C), 25.4, 25.4—8 diastereotopic –CH_3_). HRMS (ESI-TOF) *m*/*z*: [M + H]^+^ calcd for C_29_H_34_NO_7_, 508.2330; found, 508.2351.

#### (3a*S*,5*S*,6*R*,6a*S*)-5-((*S*)-2,2-Dimethyl-1,3-dioxolan-4-yl)-2,2-dimethyltetrahydrofuro[2,3-*d*][1,3]dioxol-6-yl (*cis*)-2,5-Diphenyl-3,4-dihydro-2*H*-pyrrole-3-carboxylate (**3u**)

The product was obtained as a yellow oil in 15% yield (11.6 mg, 0.023 mmol). ^1^H NMR (400 MHz, CDCl_3_): δ 7.89 (d, *J* = 6.8 Hz, 2H), 7.48–7.31 (m, 5H), 7.27 (m, 3H), 5.75 (d, *J* = 9.0 Hz, 1H), 5.27 (d, *J* = 3.7 Hz, 1H), 4.67 (d, *J* = 3.1 Hz, 1H), 3.88 (m, 2H), 3.82–3.69 (m, 4H), 3.51 (ddd, *J* = 17.1, 5.7, 1.8 Hz, 1H), 3.25 (dd, *J* = 17.1, 9.3 Hz, 1H), 2.94 (d, *J* = 3.7 Hz, 1H), 1.32 (s, 3H), 1.29 (s, 3H), 1.18 (s, 3H), 1.06 (s, 3H). This diastereomer was not stable enough to record clean NMR spectra. HRMS (ESI-TOF) *m*/*z*: [M + H]^+^ calcd for C_29_H_34_NO_7_, 508.2330; found, 508.2345.

#### (3*S*,8*S*,9*S*,10*R*,13*R*,14*S*,17*R*)-10,13-Dimethyl-17-((*R*)-6-methylheptan-2-yl)-2,3,4,7,8,9,10,11,12,13,14,15,16,17-tetradecahydro-1*H*-cyclopenta[*a*]phenanthren-3-yl-*trans*-2,5-diphenyl-3,4-dihydro-2*H*-pyrrole-3-carboxylate (**3v**)

The product was obtained as a colorless oil in 22% yield (20.7 mg, 0.0325 mmol), a mixture of two *trans* diastereoisomers at approximately 1:1 ratio (NMR analysis). ^1^H NMR (400 MHz, CDCl_3_): δ 7.99 (d, *J* = 6.0 Hz, 2H), 7.54–7.42 (m, 3H), 7.41–7.28 (m, 5H), 5.57 (d, *J* = 5.7 Hz, 1H), 5.48–5.32 (m, 1H), 4.80–4.61 (m, 1H), 3.61–3.35 (m, 2H), 3.17 (m, 1H), 2.42–2.29 (m, 2H), 2.01 (m, 2H), 1.91–1.80 (m, 3H), 1.71–1.55 (m, 5H), 1.48 (m, 5H), 1.35 (m, 3H), 1.23–1.07 (m, 7H), 1.05–1.00 (m, 4H), 0.92 (d, *J* = 6.5 Hz, 3H), 0.87 and 0.86–2(CH_3_) overlapped (d, *J* = 6.6, 6H), 0.68 (s, 3H). Note: many signals between the *trans*-diastereoisomers are overlapped. ^13^C{^1^H} NMR (100 MHz, CDCl_3_): δ 173.4, 171.9 and 171.9, 143.1, 139.5 and 139.4, 133.6, 131.0, 130.9, 128.6, 128.0, 127.4, 126.6, 122.9 and 122.9, 79.6, 77.2, 74.8, 71.8, 56.70, 56.2, 51.4, 50.0, 42.3, 39.7, 39.5 and 39.5, 38.3, 37.0, 36.6, 36.2, 35.8, 31.9 and 31.9, 29.7, 28.2, 28.0, 27.8, 24.3, 23.8, 22.8, 22.6, 21.0, 19.4, 18.7, 11.9. Note: many signals between the *trans*-diastereoisomers are overlapped. HRMS (ESI-TOF) *m*/*z*: [M + H]^+^ calcd for C_44_H_60_NO_2_, 634.4624; found, 634.4646.

#### (3*S*,8*S*,9*S*,10*R*,13*R*,14*S*,17*R*)-10,13-Dimethyl-17-((*R*)-6-methylheptan-2-yl)-2,3,4,7,8,9,10,11,12,13,14,15,16,17-tetradecahydro-1*H*-cyclopenta[*a*]phenanthren-3-yl-*cis*-2,5-diphenyl-3,4-dihydro-2*H*-pyrrole-3-carboxylate (**3t**)

The product was obtained as a yellow solid in 19% yield (18.3 mg, 0.029 mmol)—mixture of two *cis* diastereoisomers at approximately 1:1 ratio (NMR analysis). ^1^H NMR (400 MHz, CDCl_3_): δ 7.98 (d, *J* = 7.2 Hz, 2H), 7.55–7.36 (m, 4H), 7.33–7.27 (m, 1H), 7.26–7.22 (m, 1H), 7.21–7.16 (m, 2H), 5.74 (d, *J* = 5.6 Hz, 1H), 5.25 (d, *J* = 4.9 Hz, 1H), 4.21–4.02 (m, 1H), 3.77–3.57 (m, 2H), 3.34–3.04 (m, 1H), 2.09–1.87 (m, 3H), 1.85–1.64 (m, 3H), 1.60–1.45 (m, 4H), 1.45–1.40 (m, 2H), 1.40–1.29 (m, 4H), 1.28–1.18 (m, 2H), 1.18–1.05 (m, 7H), 1.05–0.94 (m, 3H), 0.92 (s, 3H), 0.90 (d, *J* = 6.5 Hz, 3H), 0.86 (dd, *J* = 6.6, 1.7 Hz, 6H), 0.65 (s, 3H). Note: many signals between the *cis*-diastereoisomers are overlapped. ^13^C{^1^H} NMR (100 MHz, CDCl_3_): δ 173.3, 173.2, 171.4 and 171.4, 139.7, 138.5, 138.4, 133.6 and 133.6, 131.2, 131.2, 129.0, 128.6, 128.2 and 128.2, 128.1, and 128.1 and 128.1, 127.8, 127.7, 122.5, 122.3, 77.3, 74.5, 74.4, 56.7, 56.1, 49.9, 48.0, 48.0, 42.3, 39.7, 39.5, 37.9, 37.1, 37.1, 36.8, 36.5, 36.2, 35.8, 31.8, 28.2, 28.0, 27.5, 26.8, 24.3, 23.8, 22.8, 22.6, 21.0, 19.2, 18.7, 11.8. Note: many signals between the *cis*-diastereoisomers are overlapped. mp 108–110 °C. HRMS (ESI-TOF) *m*/*z*: [M + H]^+^ calcd for C_44_H_60_NO_2_, 634,4624; found, 634.4638.

### General Procedure for Preparation of Pyrroles

In a Schlenk tube were added chalcone **4** (0.18 mmol), azirine **2a** (0.27 mmol), mesityl acridinium tetrafluoroborate (5.03 mg, 0.0126 mmol, 7 mol %), and anhydrous DCE (1.8 mL). This mixture was degassed (freeze–pump–thaw) three times. The reaction was then submitted to continuous flow in Syrris ASIA equipment. The solvent (DCE) in the solvent reservoir flow was previously degassed for 5 min in an ultrasound bath with an N_2_ balloon. The mixture was transferred with the aid of a syringe to a 1.5 mL loop coupled to an injection pump and pumped at a flow rate of 66.6 μL/min into a 2 mL reactor under irradiation from two lamps of 440 nm blue LED (40 W each) at a distance of 10 cm from the reactor with an RT of 30 min. The photochemical reactor was made on a 3D printer and coupled with a 3.98 m × 0.8 mm PTFE tube. The reaction crude was collected and concentrated under vacuum, solubilized in toluene (2 mL), 2,3-dichloro-5,6-dicyano-1,4-benzoquinone (68.12 mg, 0.3 mmol), and refluxed in a tube sealed for 30 min. The reaction mixture was concentrated under a vacuum and purified in a chromatographic column with silica gel and hexane-EtOAc 85:15 as the eluent. Note: since the sample loop has a capacity of 1.5 mL and the reaction mixture has a concentration of 0.1 M of acrylophenone, the value of 0.15 mmol of limiting reagent was used for yield calculation purposes.

#### (*anti*/*anti*)-Phenyl(2,4,5-triphenyl-3,4-dihydro-2*H*-pyrrol-3-yl)methanone (**3aa**)^24^

^1^H NMR (400 MHz, CDCl_3_): δ 7.72–7.67 (m, 2H), 7.46–7.41 (m, 2H), 7.41–7.35 (m, 1H), 7.32–7.13 (m, 12H), 7.12–7.09 (m, 1H), 7.08–7.04 (m, 2H), 5.49 (dd, *J* = 7.3, 1.8 Hz, 1H), 5.10 (dd, *J* = 7.7, 1.9 Hz, 1H), 4.10 (t, *J* = 7.5 Hz, 1H). ^13^C{^1^H} NMR (100 MHz, CDCl_3_): δ 200.0, 172.3, 142.7, 141.4, 136.5, 133.5, 133.1, 130.5, 129.2, 129.0, 128.9, 128.7, 128.4, 128.3, 128.2, 127.6, 127.2, 127.0, 78.6, 65.7, 60.3. HRMS (ESI-TOF) *m*/*z*: [M + H]^+^ calcd for C_29_H_24_NO, 402.1852; found, 402.1857.

#### (*anti*/*syn*)-Phenyl(2,4,5-triphenyl-3,4-dihydro-2*H*-pyrrol-3-yl)methanone (**3aa**)^24^

^1^H NMR (400 MHz, CDCl_3_): δ 7.91–7.86 (m, 2H), 7.62–7.56 (m, 2H), 7.47–7.29 (m, 8H), 7.27–7.19 (m, 3H), 7.07–6.98 (m, 3H), 6.92–6.83 (m, 2H), 6.00 (dd, *J* = 9.4, 1.7 Hz, 1H), 5.61 (dd, *J* = 6.7, 1.8 Hz, 1H), 4.61 (dd, *J* = 9.4, 6.8 Hz, 1H). ^13^C{^1^H} NMR (100 MHz, CDCl_3_): δ 197.6, 175.0, 141.4, 137.3, 133.0, 132.9, 130.9, 129.3, 129.1, 128.5, 128.4, 128.3, 128.1, 128.1, 128.1, 127.7, 127.2, 77.8, 62.5, 56.4. HRMS (ESI-TOF) *m*/*z*: [M + H]^+^ calcd for C_29_H_24_NO, 402.1852; found, 402.1854.

#### Phenyl(2,4,5-triphenyl-1*H*-pyrrol-3-yl)methanone (**5a**)

The product was obtained as a yellow solid in 44% yield (28 mg, 0.07 mmol). ^1^H NMR (400 MHz, CDCl_3_): δ 8.56 (br, 1H, N–H), 7.65 (d, *J* = 7.8 Hz, 2H), 7.34 (d, *J* = 7.4 Hz, 2H), 7.24–7.10 (m, 11H), 7.08–6.98 (m, 5H). ^13^C{^1^H} NMR (100 MHz, CDCl_3_): δ 194.7, 138.7, 134.6, 133.9, 132.2, 132.0, 131.6, 130.4, 129.9, 129.2, 128.7, 128.6, 128.0, 127.8, 127.7, 127.6, 127.4, 127.2, 126.4, 124.1, 122.3. mp 169.5–170.6 °C. HRMS (ESI-TOF) *m*/*z*: [M + H]^+^ calcd for C_29_H_22_NO, 400.1701; found, 400.1705.

#### (4-Nitrophenyl)(2,4,5-triphenyl-1*H*-pyrrol-3-yl)methanone (**5b**)

The product was obtained as an orange solid in 47% yield (31.3 mg, 0.07 mmol). ^1^H NMR (400 MHz, CDCl_3_): δ 8.84 (br, 1H, N–H), 7.93–7.88 (m, 2H), 7.82–7.67 (m, 2H), 7.41 (dd, *J* = 7.9, 1.5 Hz, 2H), 7.28–7.25 (m, 5H), 7.25–7.20 (m, 3H), 7.17–7.06 (m, 5H). ^13^C{^1^H} NMR (100 MHz, CDCl_3_): δ 192.2, 149.3, 143.9, 135.8, 134.2, 131.5, 131.2, 130.6, 130.5, 129.7, 128.7, 128.7, 128.4, 128.2, 128.2, 127.5, 127.4, 126.8, 124.1, 122.8, 121.4. mp 145.0–145.5 °C. HRMS (ESI-TOF) *m*/*z*: [M + H]^+^ calcd for C_29_H_21_N_2_O_3_, 445.1552; found, 445.1551.

#### (4-(4-Nitrophenyl)-2,5-diphenyl-1*H*-pyrrol-3-yl)(phenyl)methanone (**5c**)

The product was obtained as a yellow solid in 40% yield (27 mg, 0.061 mmol). ^1^H NMR (400 MHz, CDCl_3_): δ 8.70 (br, 1H, N–H), 7.95–7.86 (m, 2H), 7.65–7.60 (m, 2H), 7.33–7.28 (m, 2H), 7.27–7.20 (m, 6H), 7.19–7.12 (m, 5H), 7.08 (t, *J* = 7.7 Hz, 2H). ^13^C{^1^H} NMR (100 MHz, CDCl_3_): δ 194.0, 146.2, 142.1, 138.4, 135.0, 132.7, 131.1, 131.1, 131.0, 130.6, 129.9, 129.0, 128.7, 128.1, 128.0, 128.0, 127.8, 127.8, 123.3, 121.9, 121.8. mp 176.0–177.5 °C. HRMS (ESI-TOF) *m*/*z*: [M + H]^+^ calcd for C_29_H_21_N_2_O_3_, 445.1552; found, 445.1556.

#### (4-Methoxyphenyl)(2,4,5-triphenyl-1*H*-pyrrol-3-yl)methanone (**5d**)

The product was obtained as a yellow solid in 12% yield (7.9 mg, 0.018 mmol). ^1^H NMR (400 MHz, CDCl_3_): δ 8.47 (br, 1H, N–H), 7.70–7.64 (m, 2H), 7.39–7.32 (m, 2H), 7.25–7.17 (m, 7H), 7.17–7.10 (m, 3H), 7.09–7.00 (m, 3H), 6.63–6.55 (m, 2H), 3.66 (s, 3H). ^13^C{^1^H} NMR (100 MHz, CDCl_3_): δ 193.6, 163.0, 134.6, 132.7, 132.3, 132.2, 131.7, 131.6, 130.4, 130.3, 128.7, 128.7, 128.0, 127.6, 127.3, 127.2, 127.1, 126.4, 123.8, 122.6, 113.1, 55.3. mp 161.8–162.7 °C. HRMS (ESI-TOF) *m*/*z*: [M + H]^+^ calcd for C_30_H_24_NO_2_, 430.1807; found, 430.1804.

#### (4-(4-Methoxyphenyl)-2,5-diphenyl-1*H*-pyrrol-3-yl)(phenyl)methanone (**5e**)

The product was obtained as an orange oil in 16% yield (10.2 mg, 0.024 mmol). ^1^H NMR (400 MHz, CDCl_3_): δ 8.46 (br, 1H, N–H), 7.67–7.61 (m, 2H), 7.33 (d, *J* = 6.4 Hz, 2H), 7.25–7.13 (m, 8H), 7.12–7.01 (m, 4H), 7.00–6.84 (m, 1H), 6.65–6.59 (m, 2H), 3.65 (s, 3H). ^13^C{^1^H} NMR (100 MHz, CDCl_3_): δ 194.8, 158.2, 138.7, 133.7, 132.2, 132.2, 131.7, 131.5, 130.5, 130.0, 129.6, 129.1, 128.7, 128.6, 127.8, 127.7, 127.5, 127.2, 127.1, 125.8, 123.8, 113.5, 55.1. HRMS (ESI-TOF) *m*/*z*: [M + H]^+^ calcd for C_30_H_24_NO_2_, 430.1807; found, 430.1809.

#### (4-Fluorophenyl)(2,4,5-triphenyl-1*H*-pyrrol-3-yl)methanone (**5f**)

The product was obtained as a yellow solid in 31% yield (19.1 mg, 0.046 mmol). ^1^H NMR (400 MHz, CDCl_3_): δ 8.60 (br, 1H, N–H), 7.71–7.59 (m, 2H), 7.35–7.30 (m, 2H), 7.23–7.13 (m, 8H), 7.12–7.01 (m, 5H), 6.75–6.68 (m, 2H). ^13^C{^1^H} NMR (100 MHz, CDCl_3_): δ 193.1, 165.2 (d, *J* = 253.7 Hz), 135.1 (d, *J* = 2.9 Hz),134.5, 134.0, 132.4 (d, *J* = 9.3 Hz), 131.9, 131.5, 130.4, 129.3, 128.7, 128.7, 128.1, 127.9, 127.7, 127.4, 127.2, 126.5, 124.0, 122.0, 114.8 (d, *J* = 21.9 Hz). ^19^F NMR (376 MHz, CDCl_3_): δ −106.5. mp 129.1–131.4 °C. HRMS (ESI-TOF) *m*/*z*: [M + H]^+^ calcd for C_29_H_21_FNO, 418.1607; found, 418.1613.

#### Thiophen-2-yl(2,4,5-triphenyl-1*H*-pyrrol-3-yl)methanone (**5g**)

The product was obtained as a yellow solid in 24% yield (14.9 mg, 0.037 mmol). ^1^H NMR (400 MHz, CDCl_3_): δ 8.50 (br, 1H, N–H), 7.42–7.40 (m, 2H), 7.33 (dd, *J* = 4.9, 1.1 Hz, 1H), 7.26–7.20 (m, 7H), 7.20–7.14 (m, 4H), 7.12–7.02 (m, 3H), 6.69 (dd, *J* = 4.8, 3.9 Hz, 1H). ^13^C{^1^H} NMR (100 MHz, CDCl_3_): δ 186.7, 145.8, 134.8, 134.4, 133.6, 133.1, 132.0, 131.6, 130.3, 129.3, 128.8, 128.7, 128.1, 127.7, 127.5, 127.4, 127.4, 127.2, 126.5, 123.6, 122.4. mp 158.6–160.5 °C. HRMS (ESI-TOF) *m*/*z*: [M + H]^+^ calcd for C_27_H_20_NOS, 406.1266; found, 406.1263.

## Data Availability

The data underlying this study are available in the published article and its Supporting Information. The Crystallographic Information Files (CIF) of *trans*-**3c**, *cis*-**3c**, *cis*-**3g** and *trans*-**3p** were deposited in the Cambridge Structural Database under the Cambridge Crystallographic Data Centre (CCDC) numbers 2405576, 2405577, 2405578 and 2405579, respectively. Copies of the data can be obtained, free of charge, via www.ccdc.cam.ac.uk.

## Supporting Information Available

Scheme with starting materials used; NMR spectra; crystallographic data; and mechanistic studies (PDF)

## References

[ref1] Baumann M.; Baxendale I. R.; Ley S. V.; Nikbin N. An Overview of the Key Routes to the Best Selling 5-Membered Ring Heterocyclic Pharmaceuticals. Beilstein J. Org. Chem. 2011, 7, 442–495. 10.3762/bjoc.7.57. 21647262 PMC3107522

[ref2] Marusich J. A.; Darna M.; Wilson A. G.; Denehy E. D.; Ebben A.; Deaciuc A. G.; Dwoskin L. P.; Bardo M. T.; Lefever T. W.; Wiley J. L.; Reissig C. J.; Jackson K. J. Tobacco’s Minor Alkaloids: Effects on Place Conditioning and Nucleus Accumbens Dopamine Release in Adult and Adolescent Rats. Eur. J. Pharmacol. 2017, 814, 196–206. 10.1016/j.ejphar.2017.08.029. 28844873 PMC6563910

[ref3] Rother M.; Krzycki J. A. Selenocysteine, Pyrrolysine, and the Unique Energy Metabolism of Methanogenic Archaea. Archaea 2010, 2010, 453642 10.1155/2010/453642. 20847933 PMC2933860

[ref4] Xia Q.; Ganem B. Asymmetric Total Synthesis of (−)-α-Kainic Acid using an Enantioselective, Metal-Promoted Ene Cyclization. Org. Lett. 2001, 3, 485–487. 10.1021/ol007009q. 11434316

[ref5] Zhou J.; Jia M.; Song M.; Huang Z.; Steiner A.; An Q.; Ma J.; Guo Z.; Zhang Q.; Sun H.; Robertson C.; Bacsa J.; Xiao J.; Li C. Chemoselective Oxyfunctionalization of Functionalized Benzylic Compounds with a Manganese Catalyst. Angew. Chem., Int. Ed. 2022, 61, e202205983 10.1002/anie.202205983. PMC9400980 35594169

[ref6] aMa B.; Xia Q.; Wang D.; Jin J.-K.; Li Z.; Liang Q.-J.; Sun M.-Y.; Liu D.; Liu L.-J.; Shu H.-X.; Yang J.; Li D.; He J. Metal-Organic Framework Supported Copper Photoredox Catalysts for Iminyl Radical-Mediated Reactions. Angew. Chem., Int. Ed. 2023, 62, e202300233 10.1002/anie.202300233. 36896733

[ref7] Rodríguez R. I.; Mollari L.; Alemán J. Light-Driven Enantioselective Synthesis of Pyrroline Derivatives by a Radical/Polar Cascade Reaction. Angew. Chem., Int. Ed. 2021, 60, 4555–4560. 10.1002/anie.202013020. 33180379

[ref8] Liang Y.; Dong D.; Lu Y.; Wang Y.; Pan W.; Chai Y.; Liu Q. One-Pot Synthesis of Substituted Δ^1^-Pyrrolines through the Michael Addition of Nitroalkanes to Chalcones and Subsequent Reductive Cyclization in Aqueous Media. Synthesis 2006, 2006, 3301–3304. 10.1055/s-2006-950227.

[ref9] Sandvoß A.; Wahl J. M. From Cycloalkanols to Heterocycles via Nitrogen Insertion. Org. Lett. 2023, 25, 5795–5799. 10.1021/acs.orglett.3c02048. 37503963

[ref10] aCampos P. J.; Soldevilla A.; Sampedro D.; Rodríguez M. A. Simple and Versatile Synthesis of 1-Pyrroline Derivatives through Thermal Rearrangement of N-Cyclopropylimines. Tetrahedron Lett. 2002, 43, 8811–8813. 10.1016/s0040-4039(02)02238-4.

[ref11] Klausfelder B.; Blach P.; de Jonge N.; Kempe R. Synthesis of 3,4-Dihydro-2*H*-Pyrroles from Ketones, Aldehydes, and Nitro Alkanes via Hydrogenative Cyclization. Chem.—Eur. J. 2022, 28, e202201307 10.1002/chem.202201307. 35638452 PMC9545131

[ref12] Long L.; Li X.; Huang Z.; Yu Z.; Yu D.; Luo W.; Qiao L.; Chen Z.; Wang Z. Hypervalent Iodine Promoted Selective [2 + 2 + 1] Cycloaddition of Aromatic Ketones and Methylamines: A One-Pot Access to 1-Pyrrolines. J. Org. Chem. 2024, 89, 9958–9971. 10.1021/acs.joc.4c00830. 38981120

[ref13] Bidusenko I. A.; Schmidt E. Y.; Protsuk N. I.; Ushakov I. A.; Trofimov B. A. Diversifying the Superbase-Catalyzed C=N Bond Ethynylation: Triaryl-1-Pyrrolines and Triaryl-1*H*-pyrroles from N-Benzyl Aldimines and Arylacetylenes. Mendeleev Commun. 2023, 33, 24–26. 10.1016/j.mencom.2023.01.007.

[ref14] aFilippov I. P.; Novikov M. S.; Khlebnikov A. F.; Rostovskii N. V. One-Pot Synthesis of Multifunctionalized 1-Pyrrolines from 2-Alkyl-2*H*-Azirines and Diazocarbonyl Compounds. J. Org. Chem. 2022, 87, 8835–8840. 10.1021/acs.joc.2c00977. 35732058

[ref15] Lim R. K. V.; Lin Q. Azirine Ligation: Fast and Selective Protein Conjugation via Photoinduced Azirine–Alkene Cycloaddition. Chem. Commun. 2010, 46, 7993–7995. 10.1039/c0cc02863k. PMC2964347 20865197

[ref16] Guo H.; Wang H.; Zhao H.; Zhang D. Visible Light-Promoted Synthesis of 4,6a-Dihydropyrrolo[3,4-c]Pyrrole-1,3(2 H, 3aH)-Diones via [3 + 2] Cycloaddition Reaction of 2H-Azirines with Maleimides. New J. Chem. 2023, 47, 5634–5638. 10.1039/D2NJ06257G.

[ref17] aKarki B. S.; Devi L.; Pokhriyal A.; Kant R.; Rastogi N. Visible Light-Induced, Metal-Free Denitrative [3 + 2] Cycloaddition for Trisubstituted Pyrrole Synthesis. Chem.—Asian J. 2019, 14, 4793–4797. 10.1002/asia.201901068. 31605568

[ref18] aXuan J.; Xia X.; Zeng T.; Feng Z.; Chen J.; Lu L.; Xiao W. Visible-Light-Induced Formal [3 + 2] Cycloaddition for Pyrrole Synthesis under Metal-Free Conditions. Angew. Chem., Int. Ed. 2014, 53, 5653–5656. 10.1002/anie.201400602. 24729379

[ref19] aCambié D.; Bottecchia C.; Straathof N. J. W.; Hessel V.; Noël T. Applications of Continuous-Flow Photochemistry in Organic Synthesis, Material Science, and Water Treatment. Chem. Rev. 2016, 116, 10276–10341. 10.1021/acs.chemrev.5b00707. 26935706

[ref20] Donnelly K.; Baumann M. Scalability of Photochemical Reactions in Continuous Flow Mode. J. Flow Chem. 2021, 11, 223–241. 10.1007/s41981-021-00168-z.

[ref21] aKnowles J. P.; Elliott L. D.; Booker-Milburn K. I. Flow Photochemistry: Old Light through New Windows. Beilstein J. Org. Chem. 2012, 8, 2025–2052. 10.3762/bjoc.8.229. 23209538 PMC3511038

[ref22] Romero N. A.; Nicewicz D. A. Organic Photoredox Catalysis. Chem. Rev. 2016, 116, 10075–10166. 10.1021/acs.chemrev.6b00057. 27285582

[ref23] Shang T.; Lu L.; Cao Z.; Liu Y.; He W.; Yu B. Recent Advances of 1,2,3,5-Tetrakis(carbazol-9-yl)-4,6-dicyanobenzene (4CzIPN) in Photocatalytic Transformations. Chem. Commun. 2019, 55, 5408–5419. 10.1039/C9CC01047E. 31020957

[ref24] Guo Z.-W.; Huang X.; Mao J.-M.; Zhu W.-D.; Xie J.-W. Diastereoselective Synthesis of Polysubstituted Δ^1^-Pyrroline Derivatives from In Situ Generated Nitrile Ylides. RSC Adv. 2013, 3, 25103–25109. 10.1039/c3ra44736g.

[ref25] aXuan J.; Xia X.; Zeng T.; Feng Z.; Chen J.; Lu L.; Xiao W. Visible-Light-Induced Formal [3 + 2] Cycloaddition for Pyrrole Synthesis under Metal-Free Conditions. Angew. Chem., Int. Ed. 2014, 53, 5653–5656. 10.1002/anie.201400602. 24729379

[ref26] Perrin D. D.; Armarego W. L. Purification of Laboratory Chemicals, 3rd ed.; Pergamon Press: Oxford, 1988.

