# Supplementary material for: Synthesis of Δ1-Pyrrolines via Formal (3 + 2)-Cycloaddition of 2H-Azirines with Enones Promoted by Visible Light under Continuous Flow
Source: ACS Omega. 2025 Apr 23;10(17):18017–28. doi: 10.1021/acsomega.5c01416 (PMC12060062; doi:10.1021/acsomega.5c01416)
Supplement: Supplementary file 1 — ao5c01416_si_001.pdf [file ao5c01416_si_001.pdf]

Supporting Information

Synthesis of Δ^1-Pyrrolines via Formal (3 + 2)-Cycloaddition of *2H*-Azirines with Enones Promoted by Visible Light under Continuous Flow

Lorena S. R. Martelli[a‡], Lucas G. Furniel[a‡], Pedro H. O. Santiago[b], Javier Ellena[b], Arlene G. Corrêa[a*]

‡Equal contribution

[a] Centre of Excellence for Research in Sustainable Chemistry, Department of Chemistry, Federal University of São Carlos, 13565-905 São Carlos - SP, Brazil. E-mail: agcorrea@ufscar.br

[b] São Carlos Institute of Physics, University of São Paulo, 13563-120 São Carlos, SP, Brazil.

Table of Contents

1. **General information** 3
2. **Starting materials** 4
3. **Control experiments** 5
   a. **Cyclic Voltammetry Measurement** 7
   b. **Reaction with TEMPO** 8
4. **Epimerization attempts** ..... **Error! Bookmark not defined.**
5. **Unsuccessful substrates** 12
6. **Crystal structure description** 13
7. **References** 16
8. **NMR spectra** ..... **Error! Bookmark not defined.**8

1. General information

All reagents used were commercially available from Sigma-Aldrich, Synth, Exodus and Merck. The solvents used are from commercial sources and when necessary dry solvents were treated as recommended in the literature.[1] Purification of the products was performed by flash column chromatography, with silica gel 60, 230-400 mesh ASTM Merck, silica gel 60 A, 70-230 mesh AldrichCo. TLC analysis was performed on silica gel chromatoplates 60 F_{254} Merck KGaA. Nuclear Magnetic Resonance spectra were recorded on Bruker ARX 400 MHz spectrometers. Chemical shifts (δ) are expressed in ppm referenced by the residual solvent signal or TMS and coupling constants (*J*) in Hertz (Hz). To indicate the multiplicity of signs, the following abbreviation was used: *s* (singlet), *br* (broad singlet), *d* (doublet), *t* (triplet), *q* (quadruplet), *m* (multiplet). HRMS-ESI analyses were performed on an Agilent 6545 qTOF MS system (Agilent Technologies, Santa Clara, CA, USA) with a Jet electrospray interface (ESI) in positive mode. IR spectra were generated on a Shimadzu spectrophotometer, IR Spirit-X Series. The samples were diluted in dichloromethane and applied in a diamond ATR module. GC-MS analyses were performed on a Shimadzu GCMS-QP2010S with electron impact (EI) ionization using a Zebron-ZB-5MS Column. Melting points were obtained using Büchi equipment, model M-560, and reported in degrees Celsius (°C). For the photocatalyzed reactions, Kessil lamps, model PR160L, 440 nm blue LED (40 W) were used. The continuous flow reactions were carried out on Syrris ASIA Flow Chemistry Systems model 2200292 equipment. The photochemical reactor was made on a 3D printer and coupled with a 3.98 m x 0.8 mm PTFE tube. The reduction potential was determined on the IKA ElectraSyn 2.0 equipment in against Ag/Ag^+ pseudoreference electrode. Single crystal X-ray diffraction analyses were executed at 210 K using a Rigaku XtaLAB Synergy-S Dualflex diffractometer, equipped with a HyPix-6000HE detector system, and a Cu Kα (1.54184 Å) radiation.

2. Starting materials

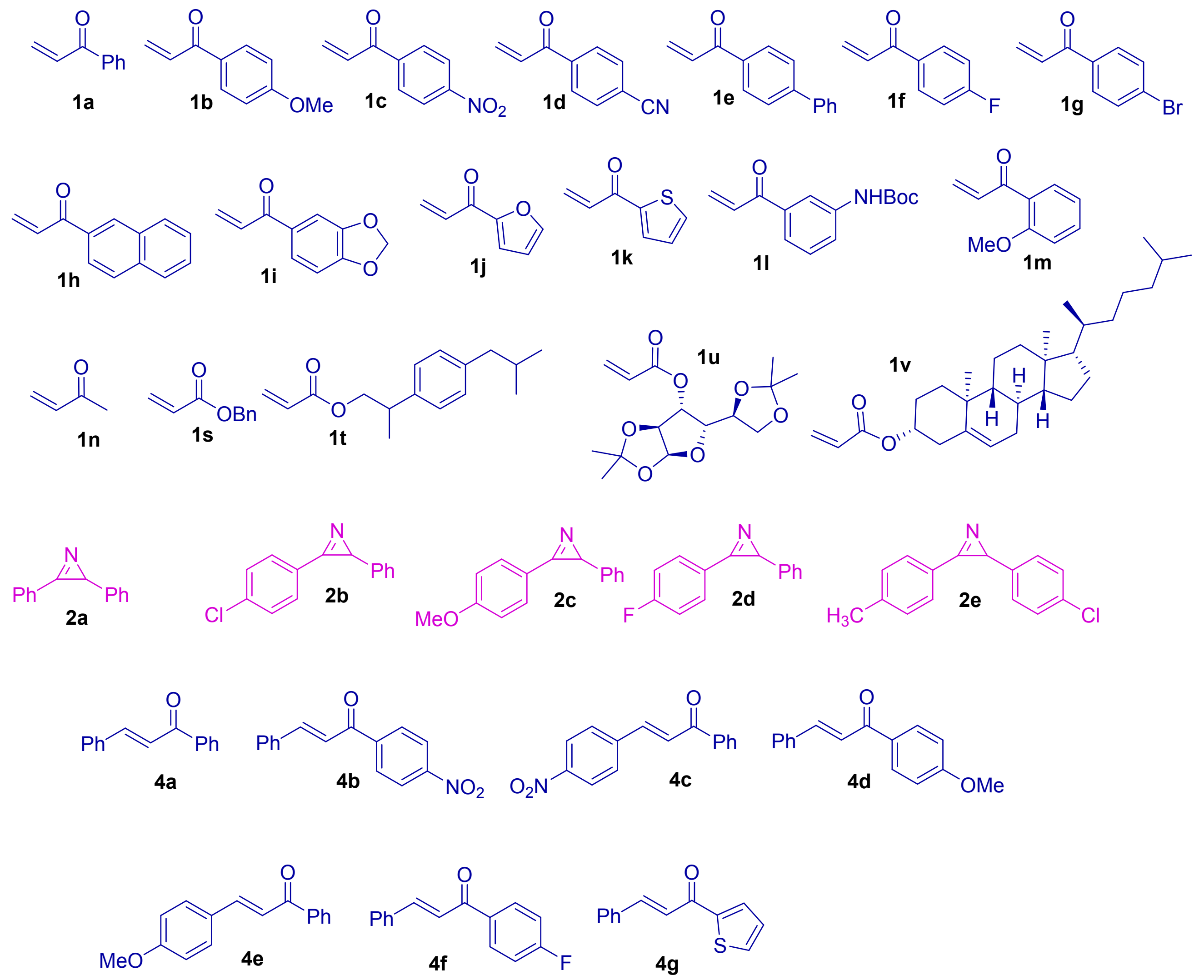

Figure S1. Starting materials used in this work.

Acryloacetophenones **1a-h**,[2] **1i**,[3] **1j**,[2] **1k**,[4] **1m**,[3] **1s**,[5] **1t**,[6] **1u**,[7] **1v**,[8] azirines **2a-d**,[9] **2e**,[10] and chalcones[11] **4a-g** were synthesized according to the literature. The experimental data is in good agreement with the reported values. Compound **1n** was obtained from commercial source and used without purification.

Procedure for the preparation of *tert*-butyl (3-acryloylphenyl)carbamate(1l)[2,12]

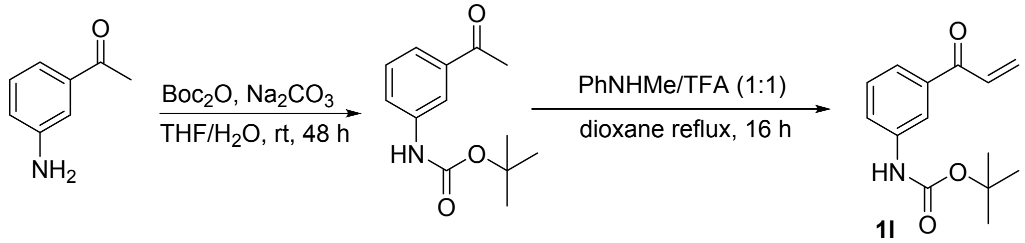

In a THF (56 mL) and water (16 mL) solution containing 3'-aminoacetophenone (2.7 g, 20 mmol) and Na_2CO_3 (2.15 g, 20.3 mmol), di-*tert*-butyl dicarbonate (5.24 g, 24 mmol) was slowly added. This mixture was stirred at room temperature for 48 hours. After this period, it was concentrated under vacuum, water was added (10 mL) and extracted with chloroform (3 x 15 mL), dried with Na_2SO_4 and concentrated under vacuum. The residue was purified in a chromatographic column with silica gel and Hexane-AcOEt 80:20 as eluent, and the *tert*-butyl(3-acetylphenyl)carbamate was obtained as an off-white solid in 93% yield (4.38 g, 18.6 mmol). This compound (1.18 g, 5 mmol) was added to a solution of *N*-methylanilinium trifluoroacetate (1.11 g, 5 mmol), trifluoroacetic acid (38.3 µL, 0.5 mmol) in dioxane (10 mL) and refluxed for 16 hs. After being cooled to room temperature, the solvent was removed by under vacuum and the residue was extracted with ethyl acetate (3 x 20 mL) and then dried with anhydrous Na_2SO_4 and concentrated under vacuum. The residue was purified in a chromatographic column with silica gel and Hexane-AcOEt 90:10 as eluent. The product **1l** was obtained as a white solid in 10% yield (0.124 g, 0.5 mmol). ^1H NMR (400 MHz, $CDCl_3$) δ: 7.88 (br, 1H), 7.63 (d, *J* = 7.3 Hz, 1H), 7.52 (d, *J* = 7.7 Hz, 1H), 7.32 (t, *J* = 7.9 Hz, 1H), 7.08 (dd, *J* = 17.1, 10.6 Hz, 1H), 6.86 (br, 1H, N-H), 6.38 (dd, *J* = 17.1, 1.3 Hz, 1H), 5.85 (dd, *J* = 10.5, 1.2 Hz, 1H), 1.45 (s, 9H). $^{13}C\{^1H\}$ NMR (100 MHz, $CDCl_3$) δ: 189.8, 151.7, 138.1, 136.9, 131.3, 129.4, 128.2, 122.2, 121.9, 117.5, 79.7, 76.3, 27.3. mp: 86.3 – 87.8 °C. HRMS (ESI-TOF) m/z: $[M+Na]^+$ calcd. for $C_{14}H_{17}NO_3Na$ 270.1106; Found: 270.1106. IR (ν_{max}): 3334, 2978, 1728, 1703, 1666, 1589, 1537, 1489, 1404, 1367, 1315, 1234, 1197, 1157, 769 cm^{-1}.

3. Control experiments

In a Schlenk tube were added the corresponding azirine **2** (0.27 mmol), acrylophenone **1** (0.18 mmol), mesityl acridinium tetrafluoroborate (5.03 mg, 0.0126 mmol, 7 mol%), and anhydrous DCE (1.8 mL). This mixture was degassed (freeze-pump-thaw) three times. The reaction was then submitted to continuous

flow in Syrris ASIA equipment. The solvent (DCE) in the solvent reservoir flow was previously degassed for 5 min in an ultrasound bath with an N_2 balloon. The reaction setup is shown in Figure S2. The mixture was then transferred with the aid of a syringe to a 1.5 mL loop coupled to an injection pump and pumped at a flow rate of 66.6 µL/min into a 2 mL reactor under irradiation from a lamp of 440 nm blue LED (40 W) at 10 cm distance from the reactor with a residence time of 30 min. The photochemical reactor was made on a 3D printer and coupled with a 3.98 m x 0.8 mm PTFE tube. The reaction crude was collected and concentrated under vacuum, purified with flash column chromatography (silica gel) using Hexane-EtOAc 90:10 to 80:20 as eluent. Note: since the sample loop has a capacity of 1.5 mL and the reaction mixture has a concentration of 0.1 M of acrylophenone, the value of 0.15 mmol of limiting reagent was used for yield calculation purposes.

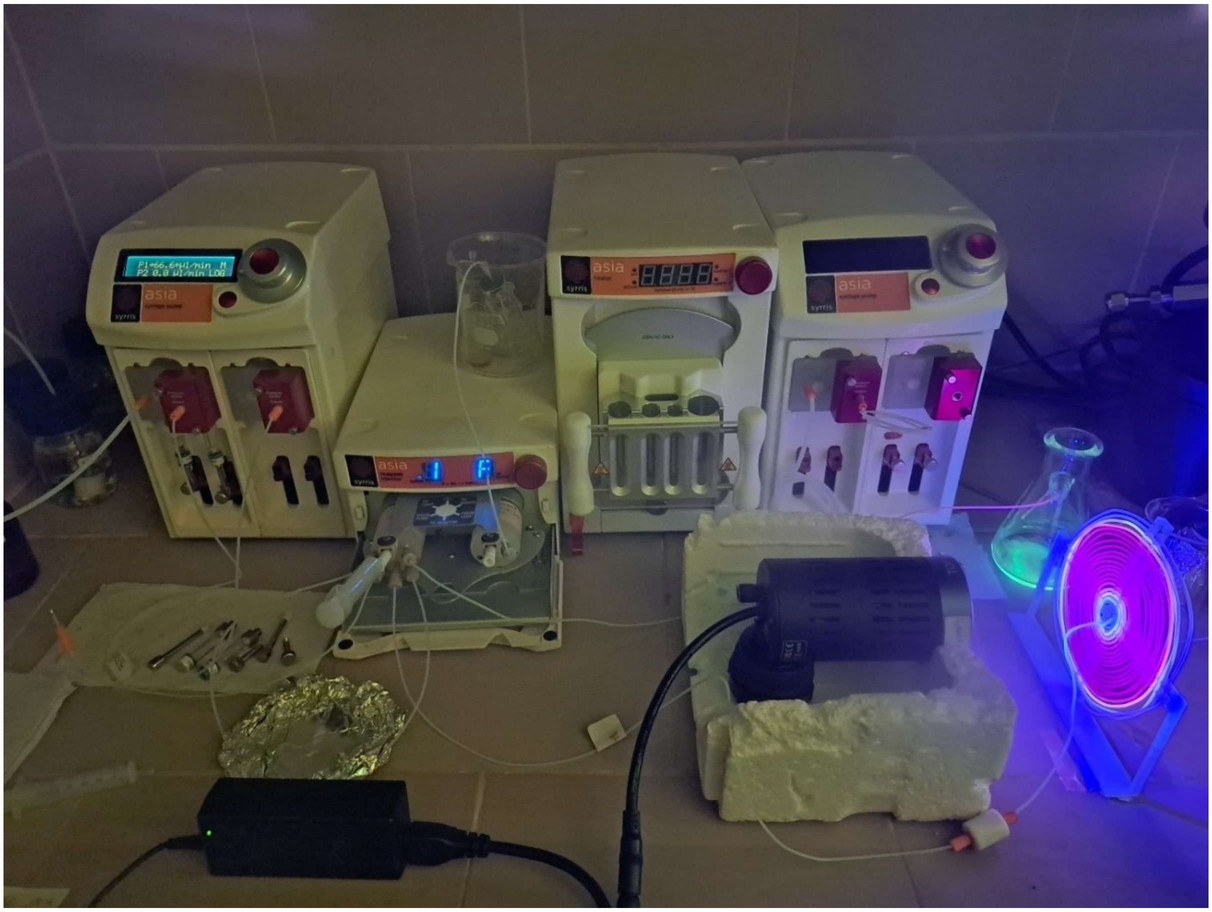

Figure S2. Reaction setup for the photo-flow reaction.

a. Cyclic Voltammetry Measurement

Cyclic voltammetry (CV) was performed on an IKA ElectraSyn 2.0 equipment. Voltammograms were obtained in degassed MeCN solution using n-Bu_4NPF_6 (0.2 M) as electrolyte. Substrate measurements occurred at a concentration of 0.02 M in MeCN and at room temperature. CVs were performed with three electrodes using a glassy carbon working electrode, a platinum counter electrode, and a saturated Ag/Ag^+ reference electrode. The CV was performed from +2,5 V to -2,5V at a 200 mV/s scan rate. The reported potentials were obtained at the top of the peak of the voltammograms.

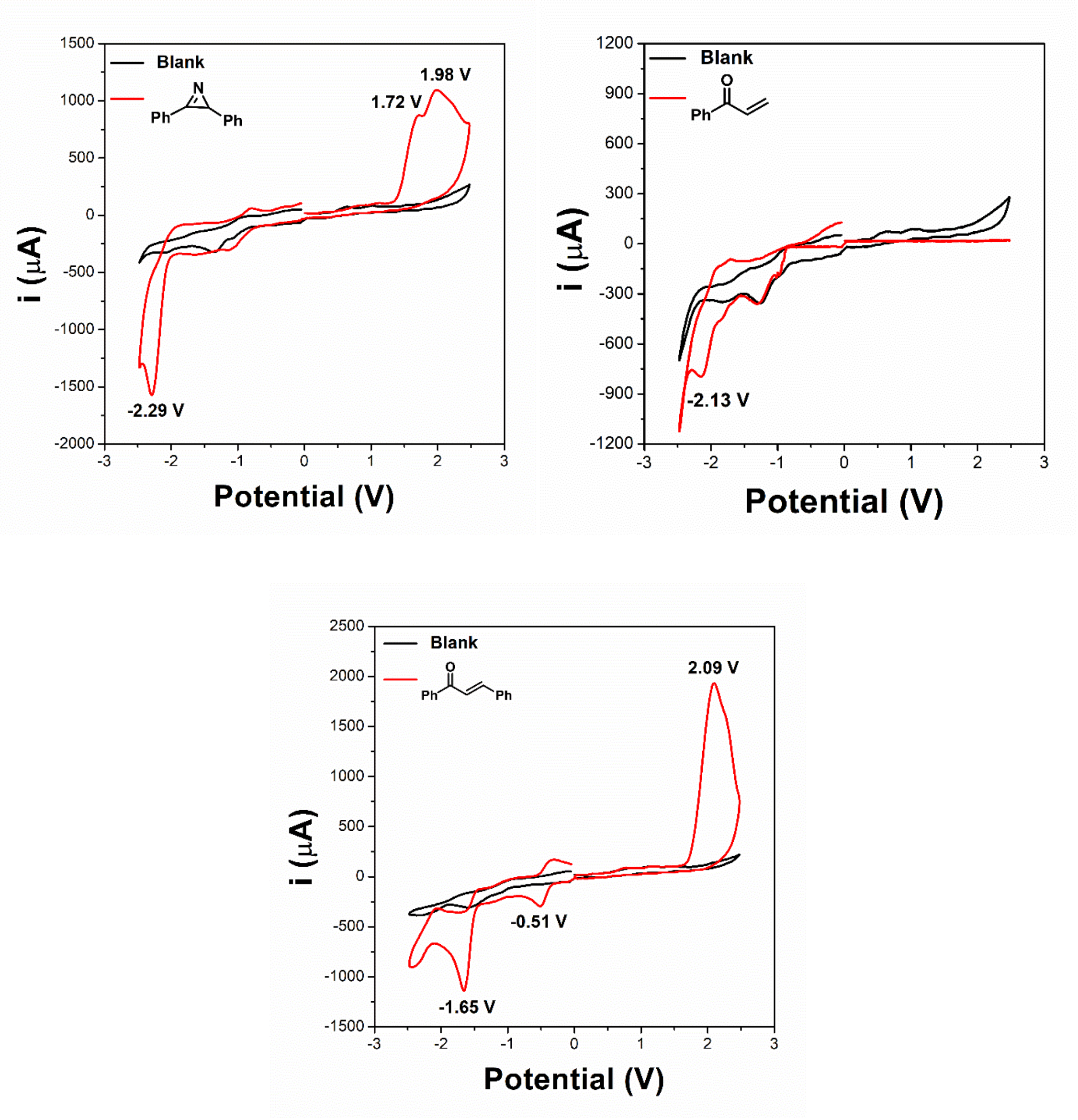

Figure S3. Voltammograms of cyclic voltammetry analysis.

b. Reaction with TEMPO

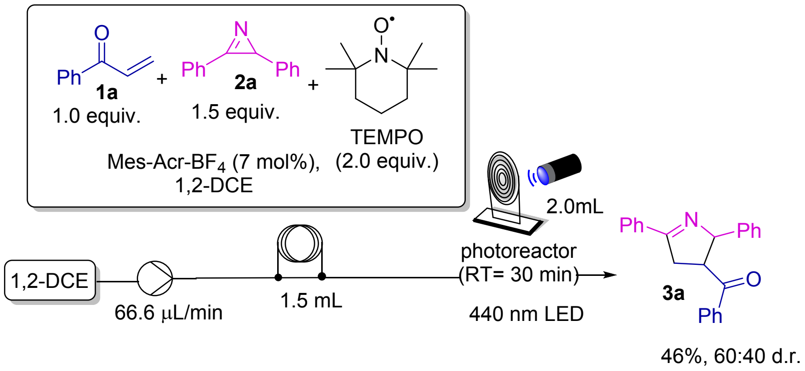

Scheme S1. Photocatalytic reaction with TEMPO as radical scavenger.

In a Schlenk tube were added the corresponding azirine **2a** (52.2 mg, 0.27 mmol), acrylophenone **1a** (23.8 mg, 0.18 mmol, 0.1 M), mesityl acridinium tetrafluoroborate (5.03 mg, 0.011 mmol, 7 mol%), TEMPO (56.2 mg, 0.36 mmol, 2.0 eq) and anhydrous DCE (1.8 mL). This mixture was degassed (Freeze-pump-thaw) three times. The reaction was then submitted to continuous flow in Syrris ASIA equipment. The solvent (DCE) in the solvent reservoir flow was previously degassed for 5 min in ultrasound with an N_2 balloon. The mixture was then transferred with the aid of a syringe to a 1.5 mL loop coupled to an injection pump and pumped at a flow rate of 66.6 µL/min into a 2 mL reactor under irradiation from a lamp of 440 nm blue LED (40 W) at 10 cm distance from the reactor with a residence time of 30 minutes. The reaction crude was collected and analyzed in GC-MS. Afterward, it was concentrated under vacuum, and purified with flash column chromatography (silica gel) using Hexane-EtOAc 90:10 to 80:20 as eluent. The product **3a** was obtained in 46% yield (22.6 mg, 0.069 mmol, 60:40 d.r.). GC-MS analysis (Zebron-ZB-5MS Column):

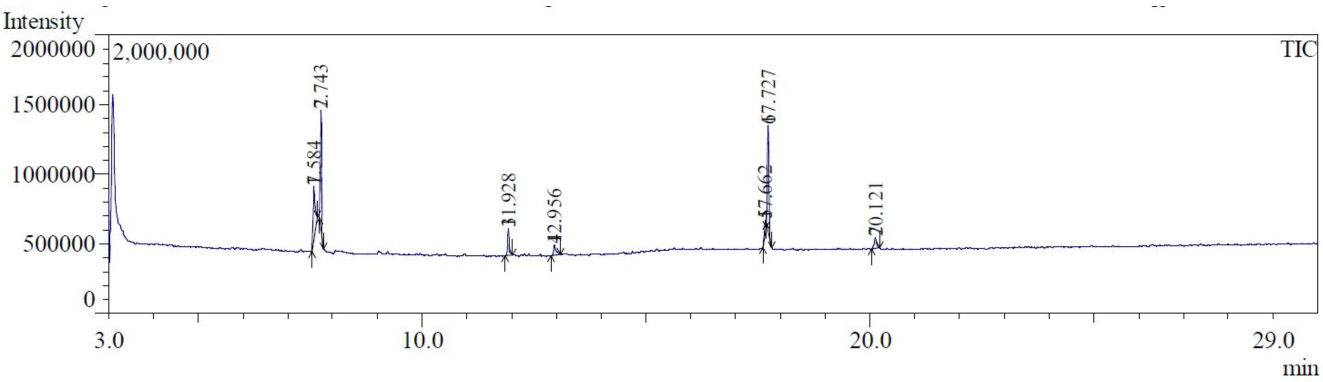

Figure S4. GC-MS chromatogram of the crude reaction mixture using TEMPO.

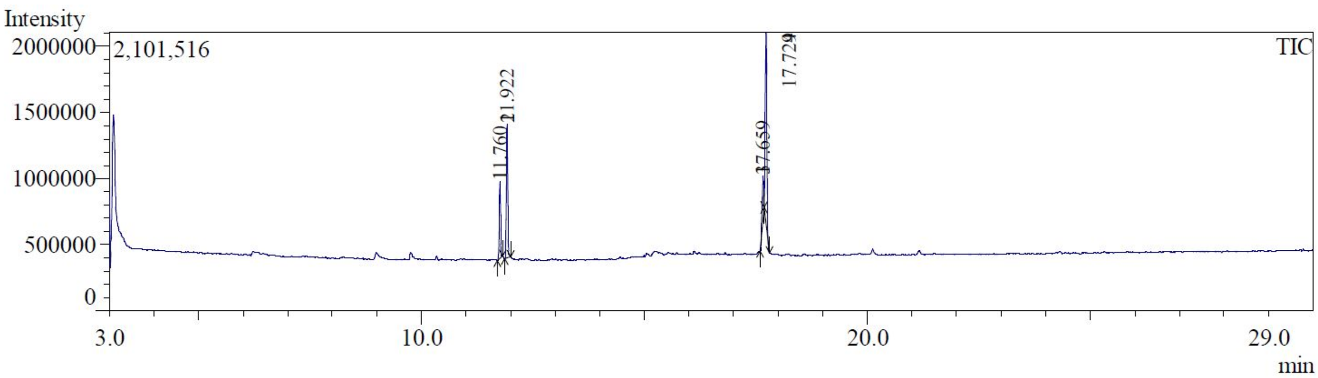

Figure S5. GC-MS chromatogram of the crude reaction mixture without TEMPO.

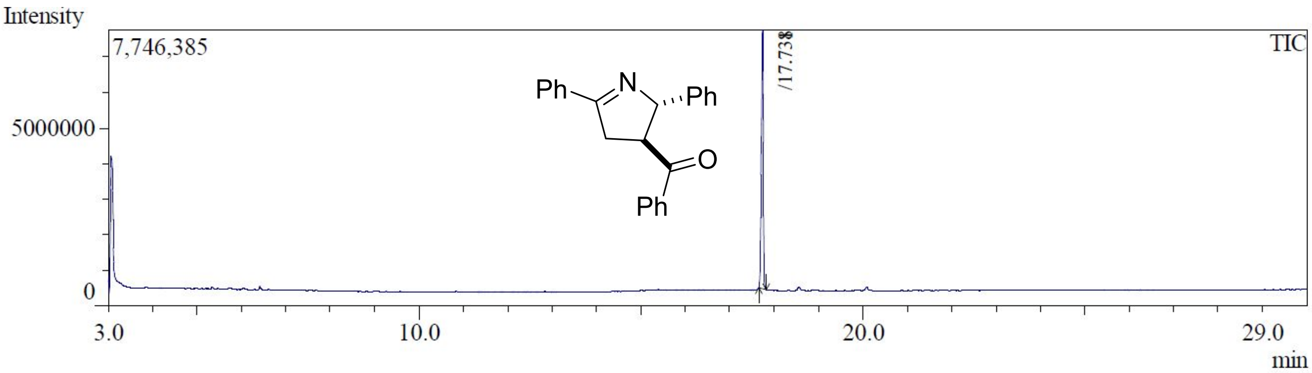

Figure S6. GC-MS chromatogram of *trans*-**3a**.

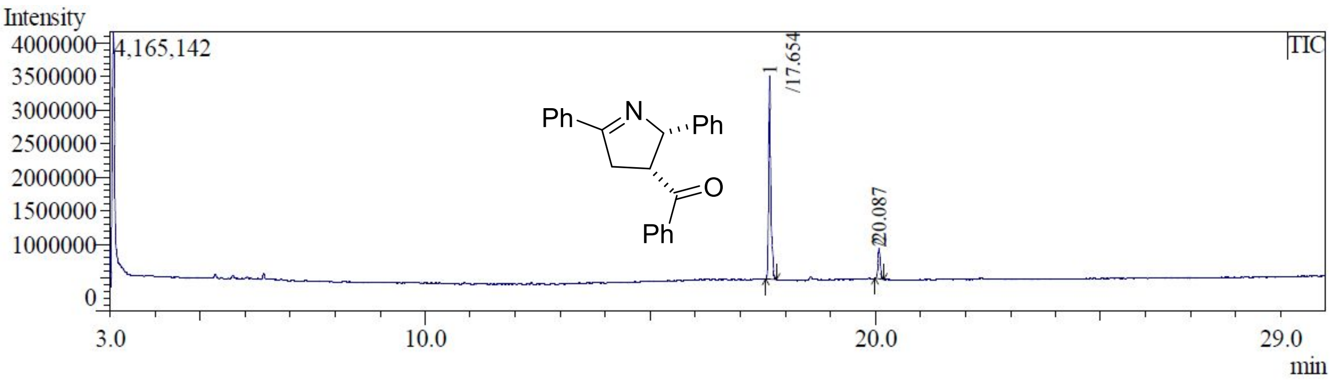

Figure S7. GC-MS chromatogram of *cis*-**3a**.

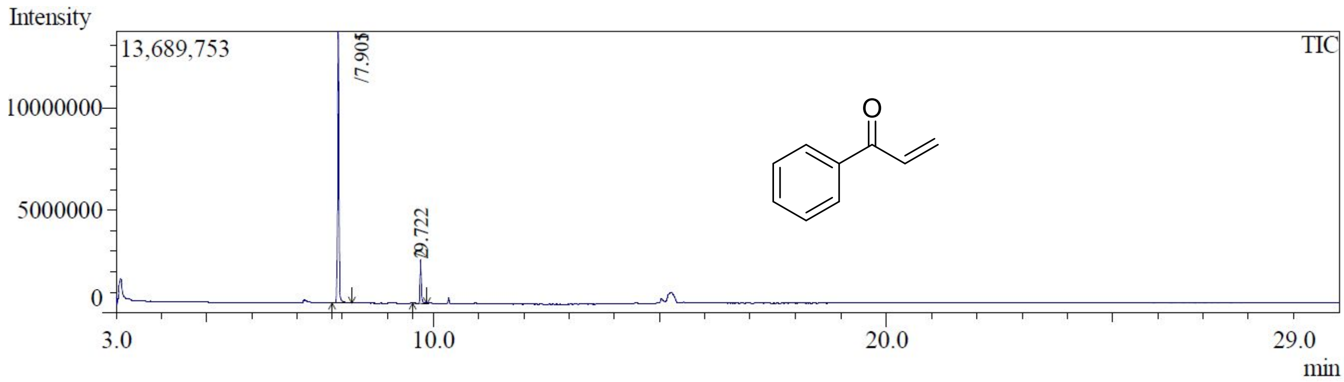

Figure S8. GC-MS chromatogram of acrylophenone **1a**.

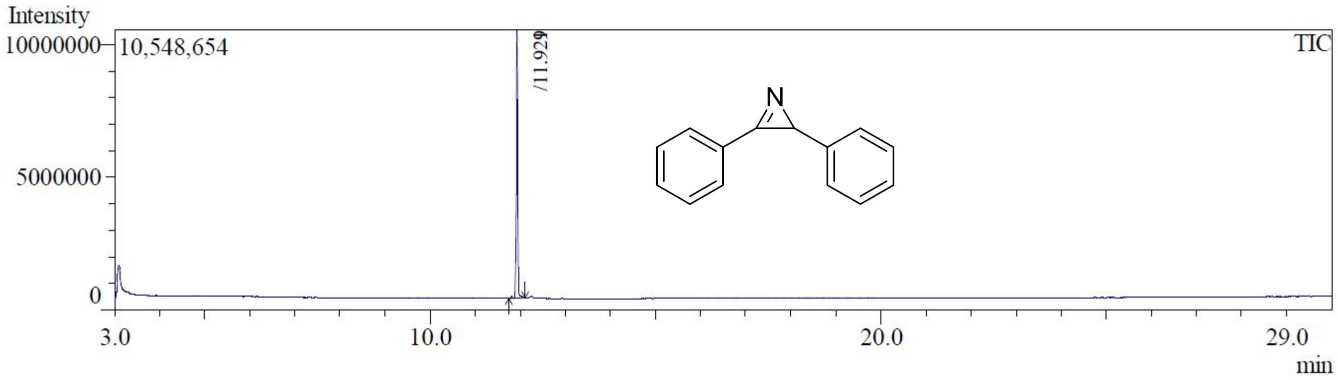

Figure S9. GC-MS chromatogram of azirine **2a**.

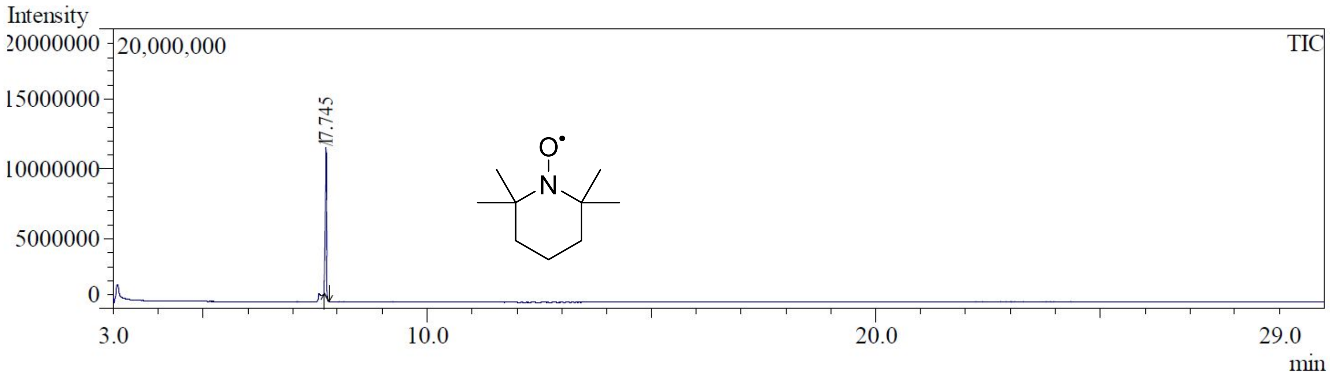

Figure S10. GC-MS chromatogram of TEMPO.

[GC-2010]
Column Oven Temp. :50.0 °C
Injection Temp. :250.00 °C
Injection Mode :Split
Flow Control Mode :Linear Velocity
Pressure :22.4 kPa
Total Flow :16.4 mL/min
Column Flow :0.64 mL/min
Linear Velocity :29.0 cm/sec
Purge Flow :3.0 mL/min
Split Ratio :20.0
High Pressure Injection :OFF
Carrier Gas Saver :OFF
Splitter Hold :OFF
Oven Temp. Program

Rate	Temperature(°C)	Hold Time(min)
-	50.0	2.50
20.00	300.0	15.00

< Ready Check Heat Unit >
Column Oven : Yes
SPL1 : Yes
MS : Yes
< Ready Check Detector(FTD) >
< Ready Check Baseline Drift >
< Ready Check Injection Flow >
SPL1 Carrier : Yes
SPL1 Purge : Yes
< Ready Check APC Flow >
< Ready Check Detector APC Flow >
External Wait :No
Equilibrium Time :3.0 min

Figure S11. GC-MS conditions used in all the runs depicted above.

4. Epimerization attempts

Initial attempts to epimerize pyrroline **3a** from the crude reaction mixture with NaOH (2.0 equiv.), first without evaporation of 1,2-DCE, then with evaporation of 1,2-DCE and addition of MeCN (2.0 mL) resulted in the formation of a complex mixture after stirring for 16 h at room temperature. Next, we evaluated the epimerization of both isolated diastereoisomers (0.02 mmol scale) in four conditions, as depicted in Table S1. The qualitative results were obtained by analyzing the TLC of each mixture.

Table S1. Epimerization attempts from pure *trans*- and *cis*-**3a**.

**Diastereomer**	**Reaction conditions**			
	2.0 equiv. Et_3N, 2.0 mL of 1,2-DCE	2.0 equiv. NaOH, 2.0 mL of 1,2-DCE	2.0 equiv. NaOH, 2.0 mL of MeCN	20 mg of Amberlyst 15, 2.0 mL of MeCN
***Trans*-3a**	No reaction	Formation of a mixture of diastereoisomers (*trans* major), plus degradation products after 48h	Formation of a mixture of diastereoisomers (*trans* major), plus degradation products after 16h	No reaction
***Cis*-3a**	No reaction	Formation of a mixture of diastereoisomers (*trans* major), plus degradation products after 48h	Formation of a mixture of diastereoisomers (*trans* major), plus degradation products after 16h	No reaction

Using the most promising conditions, the mixture of **3a** diastereoisomers (34.5 mg, 0.106 mmol, 61:39 d.r.) was dissolved in MeCN (2.0 mL). To this solution, NaOH (2.0 equiv.) was added, and the reaction was stirred at room temperature for 16 h (Scheme S2). After this time, the solvent was removed, water (10 mL) was added and the aqueous phase was extracted with DCM (3 x 10 mL). Combined organic phases were washed with brine, dried with Na_2SO_4, and evaporated. This residue was purified with flash column chromatography (10% EtOAc/Hexanes to 20% EtOAc/Hexanes). *trans*-**3a** (13.5 mg, 0.042 mmol) and *cis*-**3a** (2.0 mg, 0.0062 mmol) were obtained, in 45% combined yield and 87:13 d.r.

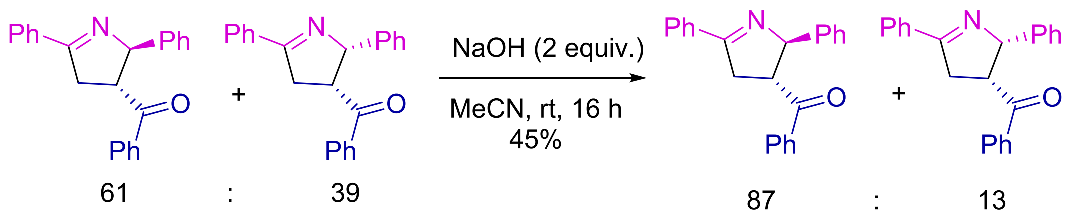

Scheme S3. Epimerization of **3a** in basic conditions.

Since the best result only allowed incomplete epimerization and a significant amount of byproduct formation, no further epimerization was attempted for any other product.

5. Unsuccessful substrates:

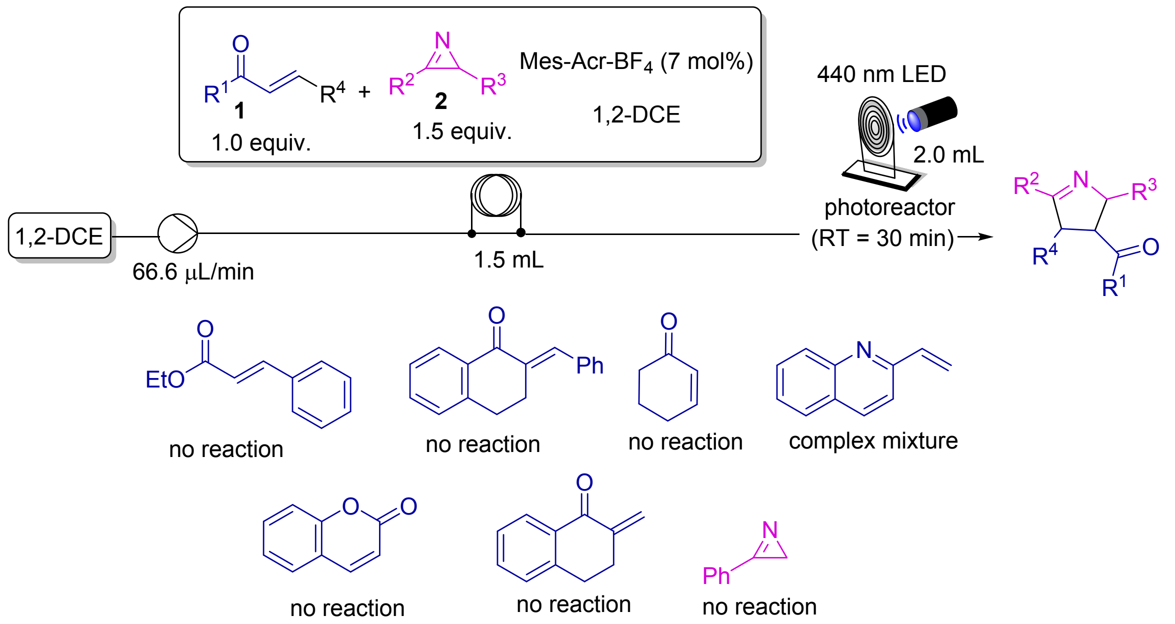

Scheme S4. Unsuccessful substrates.

6. Crystal structure description

Data collection and reduction, cell refinement and absorption correction were performed on CrysAlisPro program[15]. The structures were solved using the intrinsic phasing method from the SHELXT software[16] and refined with the least-squares method from SHELXL,[17] both integrated within the Olex2 program.[18] The non-hydrogen atoms were refined considering anisotropic displacement parameters, while the hydrogen atoms were refined isotropically at idealized positions using the riding model. Data collection and refinement parameters are summarized in Table S2.

Table S2. Crystallographic data and refinement parameters for *trans*-**3c**, *cis*-**3c**, *cis*-**3g** and *trans*-**3p**.

	trans-3c	*cis*-3c	*cis*-3g	*trans*-3p
Molecular formula	$C_{23}H_{18}N_2O_3$	$C_{23}H_{18}N_2O_3$	$C_{23}H_{18}BrNO$	$C_{23}H_{18}BrNO$
Formula weight (g mol^{-1})	370.39	370.39	404.29	359.83
Crystal system	Monoclinic	Monoclinic	Triclinic	Monoclinic
Space group	$P2_1/c$	$P2_1/c$	P-1	$P2_1/c$
a (Å)	14.6403(2)	10.5561(1)	9.3652(2)	9.9572(1)
b (Å)	8.6390(1)	12.3342(1)	9.7109(3)	22.3592(3)
c (Å)	16.0653(2)	15.0347(2)	12.5480(3)	8.1264(1)
α (°)	90	90	90.351(2)	90
β (°)	113.962(2)	106.848(1)	110.303(2)	94.660(1)
γ (°)	90	90	118.216(3)	90
Volume (Å^3)	1856.78(5)	1873.51(4)	922.88(5)	1803.24(4)
Z	4	4	2	4
ρ_{calc} (g/cm^3)	1.325	1.313	1.455	1.325
μ (mm^{-1})	0.719	0.713	3.111	1.951
Absorption correction	Multi-scan	Multi-scan	Gaussian	Multi-scan
T_{min}/T_{max}	0.854/1.000	0.886/1.000	0.671/1.000	0.845/1.000
2θ range for data collection (°)	11.15 to 140.08	9.44 to 140.14	10.56 to 140.15	8.91 to 140.15
Index ranges	-17, 17; -10, 10; -19, 18	-12, 11; -15, 15; -18 , 18	-11, 11; -11, 11; -15 , 13	-11, 12; -26, 27; -9 , 8
Reflections collected	20592	20024	19148	19993
Independent reflections/R_{int}	3529/0.0264	3553/0.0239	3500/0.0367	3416/0.0282
Goodness-on-fit on F^2	1.058	1.052	1.106	1.079
R_1/wR_2 [I >2σ(I)]	0.0359/0.0941	0.0351/0.0918	0.0510/0.1302	0.0329/0.0889
$\Delta\rho_{max}$; $\Delta\rho_{min}$ (e Å^{-3})	0.16/–0.15	0.20/–0.20	1.66/–0.70	0.20/-0.25

Suitable crystals of *trans*-**3c**, *cis*-**3c**, *cis*-**3g** and *trans*-**3p** were obtained and analysed by single crystal X-ray diffraction technique. The asymmetric unit of the elucidated structures are shown in Figure S12, while selected bond lengths and angles are listed in Table S3.

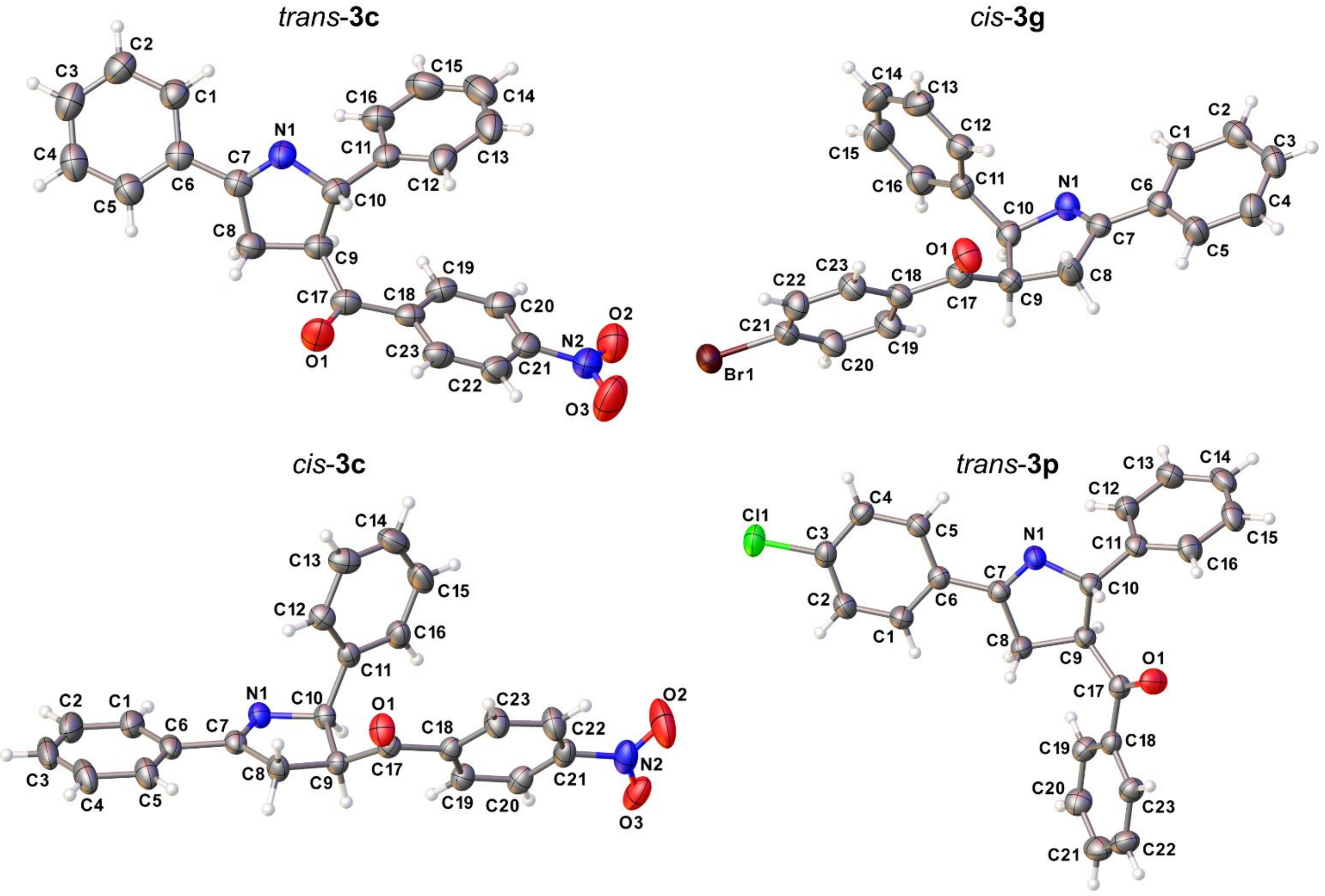

Figure S12. ORTEP-type illustration of the asymmetric unit of *trans*-**3c**, *cis*-**3c**, *cis*-**3g** and *trans*-**3p**. Ellipsoids represented at 50% of probability level.

The X-ray analyses revealed that *trans*-**3c**, *cis*-**3c**, and *trans*-**3p** crystallize in the monoclinic space group $P2_1/c$, while the *cis*-**3g** crystallizes in the triclinic space group P-1, with the four structures having asymmetric unit composed of one molecule of the correspondent compound. All the compounds were obtained as a racemic mixture, since they all crystallize in a centrosymmetric space group. It was possible to obtain single crystals of the two diastereoisomers of compound **3c**, while just the *cis* and *trans* diastereoisomers of **3g** and **3p**, respectively, were obtained as suitable crystals for X-ray diffraction analysis. The C7–N1 bonds in the four compounds have distances ranging from 1.274 Å to 1.278 Å and show a character of double bond, while the other bonds from the pyrroline ring have a character of single bond, according to the expected for pyrroline rings.[19-21]

Table S3. Selected bond lengths and angles for *trans*-**3c**, *cis*-**3c**, *cis*-**3g** and *trans*-**3p**.

	trans-3c	*cis*-3c	*cis*-3g	*trans*-3p
C7–N1	1.2762(15)	1.2778(15)	1.274(3)	1.2746(16)
C7–C8	1.5076(16)	1.5070(15)	1.500(3)	1.5082(16)
C8–C9	1.5254(17)	1.5235(14)	1.523(3)	1.5374(16)
C9–C10	1.5725(15)	1.5753(15)	1.582(3)	1.5535(15)
C10–N1	1.4705(15)	1.4712(14)	1.473(3)	1.4767(15)
C17–O1	1.2120(15)	1.2122(14)	1.214(3)	1.2165(15)
C7–N1–C10	110.10(9)	109.95(9)	110.62(18)	109.43(10)
C6–C7–N1	121.44(10)	122.40(10)	121.8(2)	123.01(11)
C9–C17–O1	121.09(11)	121.95(10)	121.2(2)	120.49(11)

7. References

(1) Perrin, D. D., Armarego, W. L. *Purification of Laboratory Chemicals*, 3rd ed.; Pergamon Press: Oxford, 1988.

(2) Duan, Y.; Zhou, B.; Lin, J.-H.; Xiao, J.-C. Diastereoselective Johnson–Corey–Chaykovsky Trifluoroethylidenation. *Chem. Commun*. **2015**, *51*, 13127–13130. DOI: 10.1039/C5CC04991A

(3) Maity, S.; Szpilman, A. M. 2-Fluoroenones via an Umpolung Morita–Baylis–Hillman Reaction of Enones. *Org. Lett*. **2023**, *25*, 1218–1222. DOI: 10.1021/acs.orglett.3c00313

(4) Ghosh, A.; Lipisa, Y. B.; Fridman, N.; Szpilman, A. M. 2-Nitro-Cyclopropyl-1-Carbonyl Compounds from Unsaturated Carbonyl Compounds and Nitromethane via Enolonium Species. *J. Org. Chem.* **2023**, *88*, 1977–1987. DOI: 10.1021/acs.joc.2c02125

(5) Lazzarotto, M.; Hartmann, P.; Pletz, J.; Belaj, F.; Kroutil, W.; Payer, S. E.; Fuchs, M. Asymmetric Allylation Catalyzed by Chiral Phosphoric Acids: Stereoselective Synthesis of Tertiary Alcohols and a Reagent-Based Switch in Stereopreference. *Adv. Synth. Catal.* **2021**, *363*, 3138–3143. DOI: 10.1002/adsc.202100037

(6) Matsumoto, A.; Maeda, N.; Maruoka, K. Bidirectional Elongation Strategy Using Ambiphilic Radical Linchpin for Modular Access to 1,4-Dicarbonyls via Sequential Photocatalysis. *J. Am. Chem. Soc.* **2023**, *145*, 20344–20354. DOI: 10.1021/jacs.3c05337

(7) Ferreira, M. L. G.; Pinheiro, S.; Perrone, C. C.; Costa, P. R. R.; Ferreira, V. F. New Carbohydrate-Based Chiral Auxiliaries in Diels–Alder Reaction. *Tetrahedron Asymmetry* **1998**, *9*, 2671–2680. DOI: 10.1016/S0957-4166(98)00286-9

(8) Choudhary, S.; Cannas, D. M.; Wheatley, M.; Larrosa, I. A manganese(i)tricarbonyl-catalyst for near room temperature alkene and alkyne hydroarylation. *Chem. Sci.* **2022**, *13*, 1325-13230. DOI: 10.1039/d2sc04295a

(9) Zhao, M.-N.; Zhang, W.; Wang, X.-C.; Zhang, Y.; Yang, D.-S.; Guan, Z.-H. Modular 2,3-Diaryl-2 H - Azirine Synthesis from Ketoxime Acetates via Cs2 CO3 -Mediated Cyclization. *Org. Biomol. Chem.* **2018**, *16*, 4333–4337. DOI: 10.1039/C8OB00923F

(10) Cai, B.-G.; Chen, Z.-L.; Xu, G.-Y.; Xuan, J.; Xiao, W.-J. [3 + 2]-Cycloaddition of *2H*-Azirines with Nitrosoarenes: Visible-Light-Promoted Synthesis of 2,5-Dihydro-1,2,4-Oxadiazoles. *Org. Lett.* **2019**, *21* (11), 4234–4238. DOI: 10.1021/acs.orglett.9b01416

(11) Martelli, L. S. R.; Vieira, L. C. C.; Paixão, M. W.; Zukerman-Schpector, J.; de Souza, J. O.; Aguiar, A. C. C.; Oliva, G.; Guido, R. V. C.; Corrêa, A. G. Organocatalytic Asymmetric Vinylogous 1,4-Addition of α,α-Dicyanoolefins to Chalcones under a Bio-Based Reaction Media: Discovery of New Michael Adducts with Antiplasmodial Activity. *Tetrahedron* **2019**, *75*, 3530–3542. DOI: 10.1016/j.tet.2019.05.022.

(12) Satyanarayana, M.; Shukla, P.; Tiwari, P.; Tripathi, B. K.; Srivastava, A. K.; Pratap, R. Synthesis of Thiourea and Sulfonylurea Derivatives of Chalcones and Flavones and Their Biological Evaluation. *Indian J. Heterocycl. Chem*. **2021**, *31*, 341–345.

(13) Xuan, J.; Xia, X.; Zeng, T.; Feng, Z.; Chen, J.; Lu, L.; Xiao, W. Visible-Light-Induced Formal [3+2] Cycloaddition for Pyrrole Synthesis under Metal-Free Conditions. *Angew. Chem. Int. Ed.* **2014**, *53*, 5653–5656. DOI: 10.1002/anie.201400602

(14) Yim, D.; Kim, Y.; Park, J. H.; Kim, H. Electronic Structure of 1,3-diphenyl-2-azaallenyl Radical Cation. *J. Phys. Org. Chem.* **2024**, *37*, e4590. DOI: 10.1002/poc.4590.

(15) CrysAlisPro, Agilent Technologies Ltd.: Yarnton, Oxfordshire, UK, 2022

(16) Sheldrick, G.M. SHELXT - Integrated Space-Group and Crystal-Structure Determination. *Acta Crystallogr. Sect. A Found. Crystallogr.* **2015**, *71*, 3–8. DOI: 10.1107/S2053273314026370

(17) Sheldrick, G.M. Crystal Structure Refinement with SHELXL. *Acta Crystallogr. Sect. C Struct. Chem.* **2015**, *71*, 3–8. DOI:10.1107/S2053229614024218

(18) Dolomanov, O. V; Bourhis, L.J.; Gildea, R.J.; Howard, J.A.K.; Puschmann, H. OLEX2: A Complete Structure Solution, Refinement and Analysis Program. *J. Appl. Crystallogr*. **2009**, *42*, 339–341. DOI:10.1107/S0021889808042726.

(19) Zhao, L.; Yan, Y.; Wei, Z.; Liao, W. Organocatalytic Allylic Alkylation of α-(Alkylideneamino)nitriles and Its Application in the Preparation of Multisubstituted 1-Pyrrolines. *J. Org. Chem.* **2022**, *87*, 15, 10090–10104. DOI: 10.1021/acs.joc.2c01072

(20) Bergner, I.; Wiebe, C.; Meyer, N.; Opatz, T.; Cyclocondensation of α-Aminonitriles and Enones: A Short Access to 3,4-Dihydro-2H-pyrrole 2-carbonitriles and 2,3,5-Trisubstituted Pyrroles. *J. Org. Chem.* **2009**, *74*, 21, 8243–8253. DOI: 10.1021/jo901759u

(21) Huang, X.; Li, X.; Xie, X; Harms, K.; Riedel, R.; Meggers, E.; Catalytic asymmetric synthesis of a nitrogen heterocycle through stereocontrolled direct photoreaction from electronically excited state. *Nat. Commun.*, **2017**, 8, 2245. DOI: 10.1038/s41467-017-02148-1

8. NMR spectra

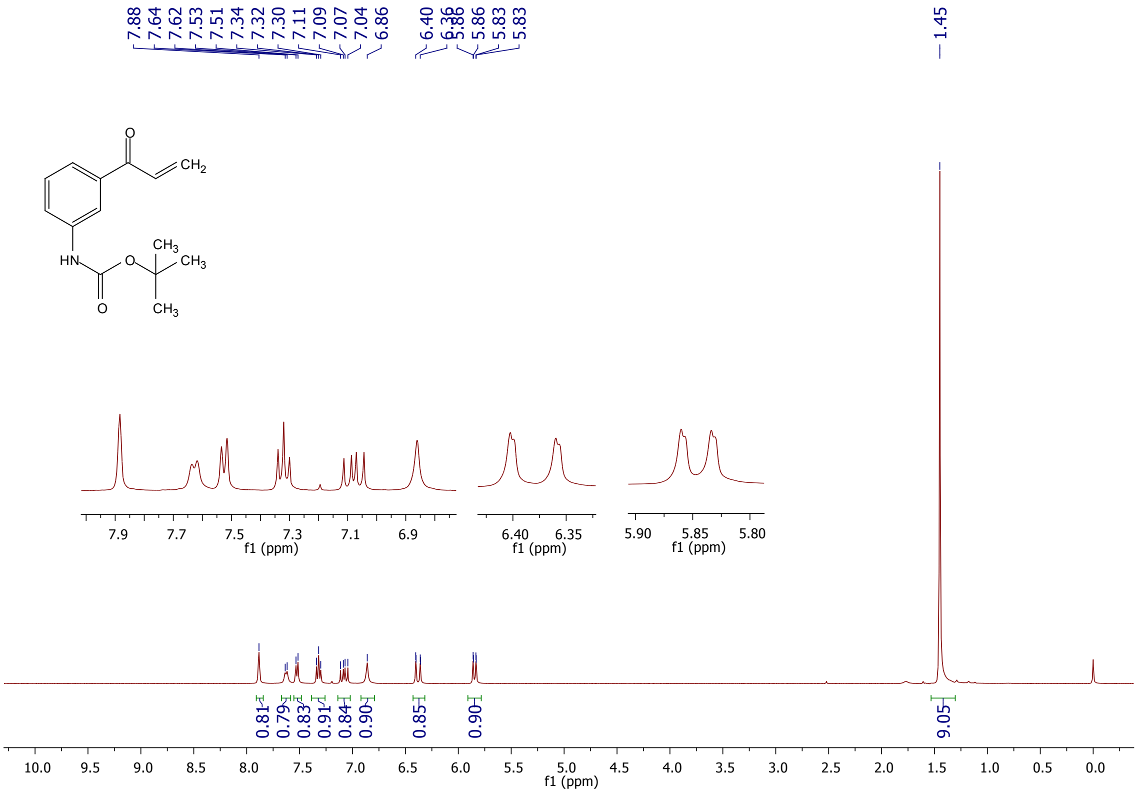

Figure S13. **¹H NMR** (400 MHz, $CDCl_3$) of compound **1l.**

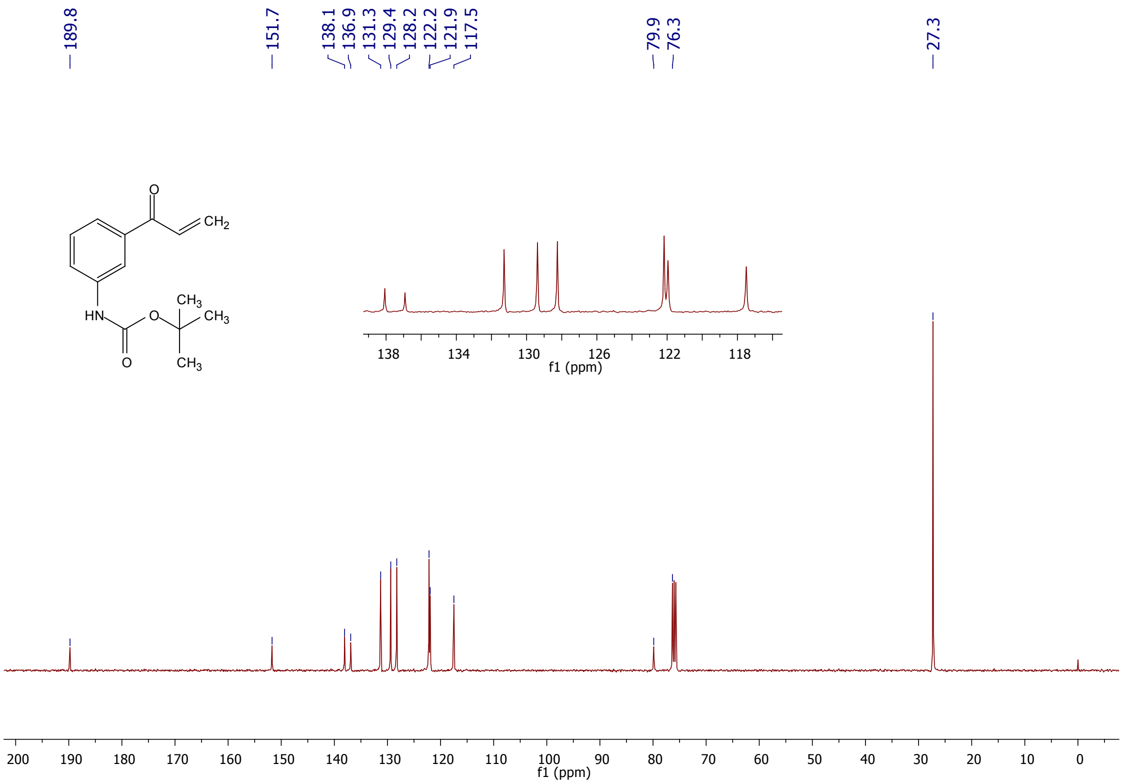

Figure S14. **$^{13}C\{^1H\}$ NMR** (100 MHz, $CDCl_3$) of compound **1l.**

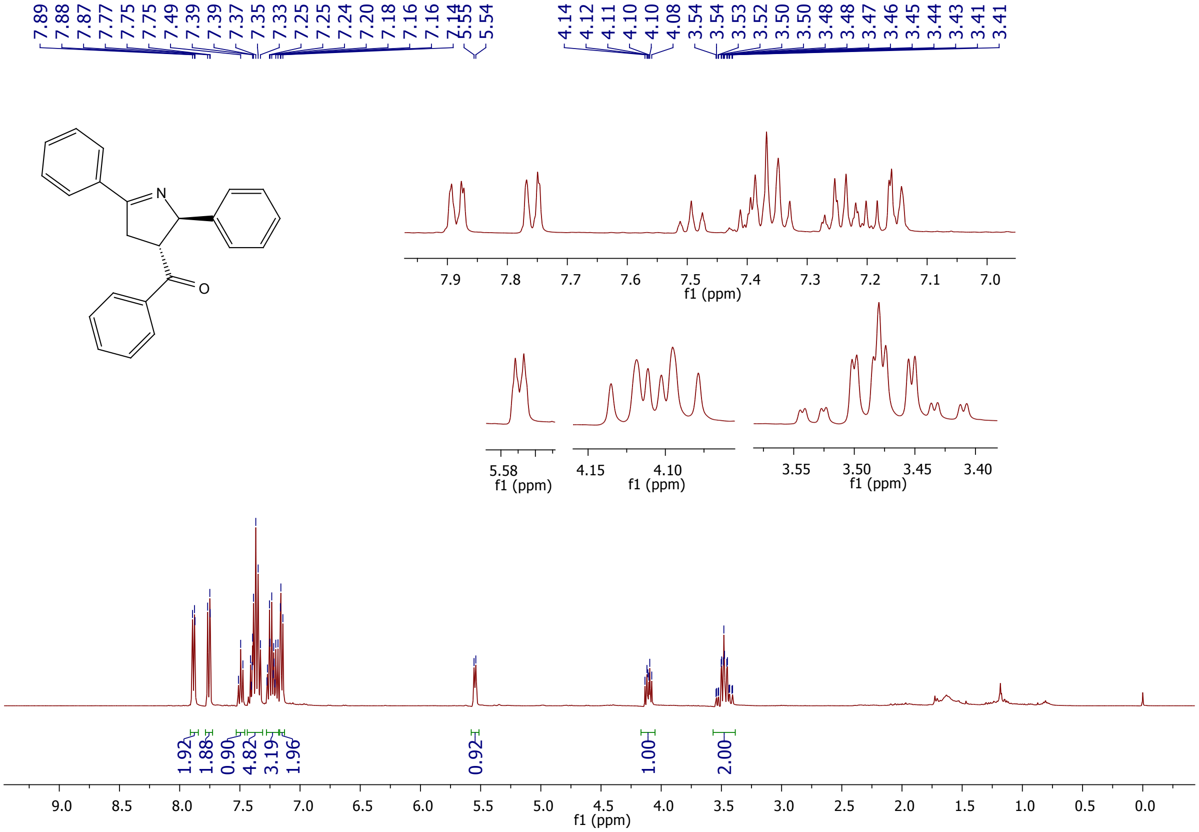

Figure S15. ^{1}**H NMR** (400 MHz, $CDCl_3$) of compound *trans*-**3a.**

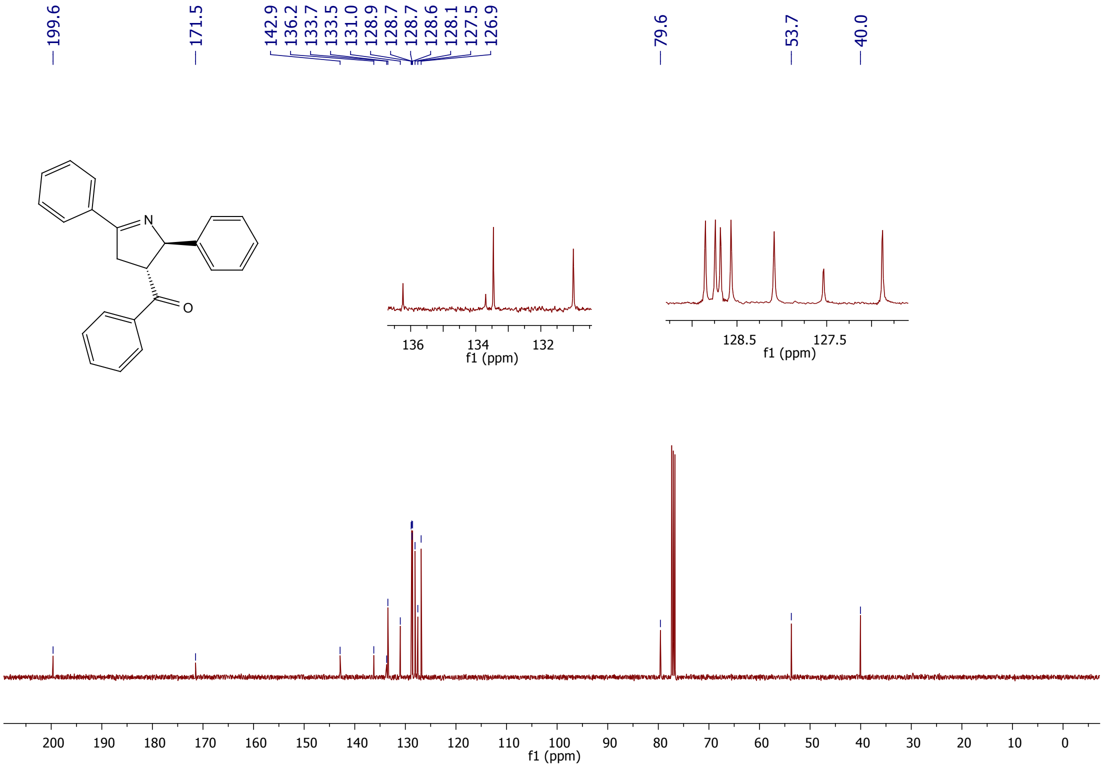

Figure S16. ^{13}**C{^{1}H} NMR** (100 MHz, $CDCl_3$) of compound *trans*-**3a.**

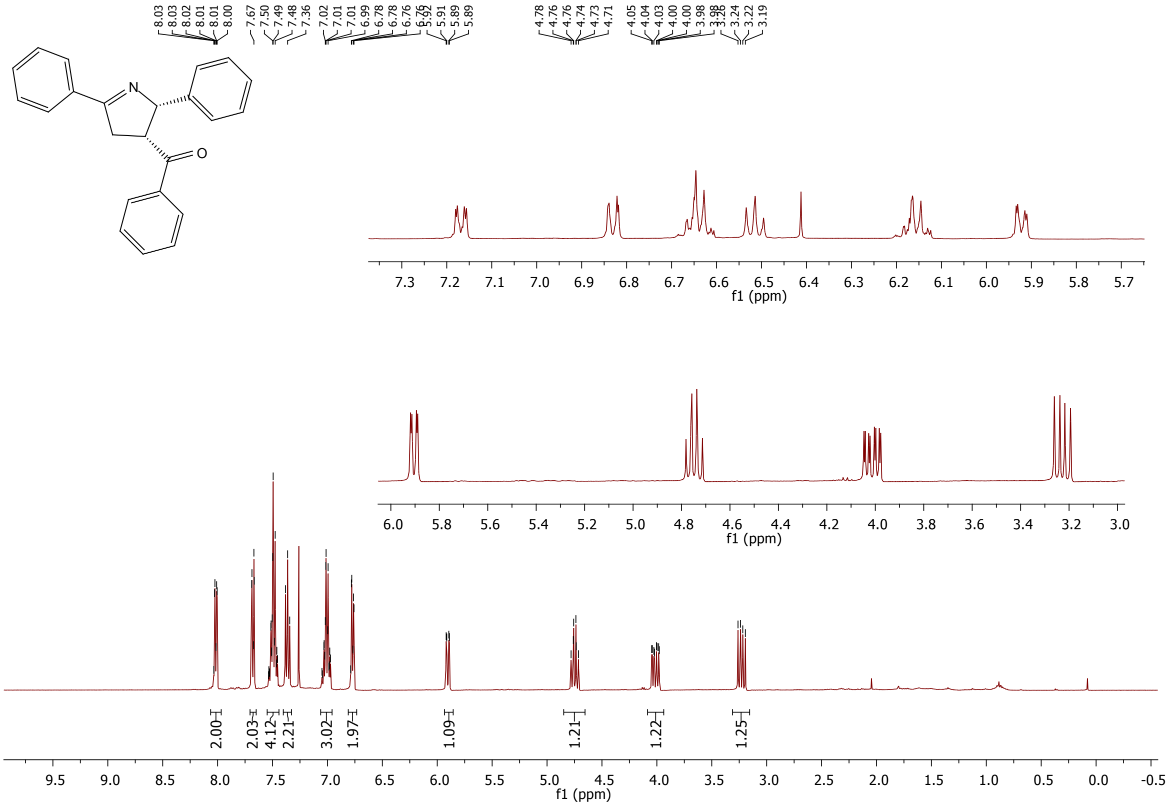

Figure S17.[1]**H NMR** (400 MHz, $CDCl_3$) of compound *cis*-**3a.**

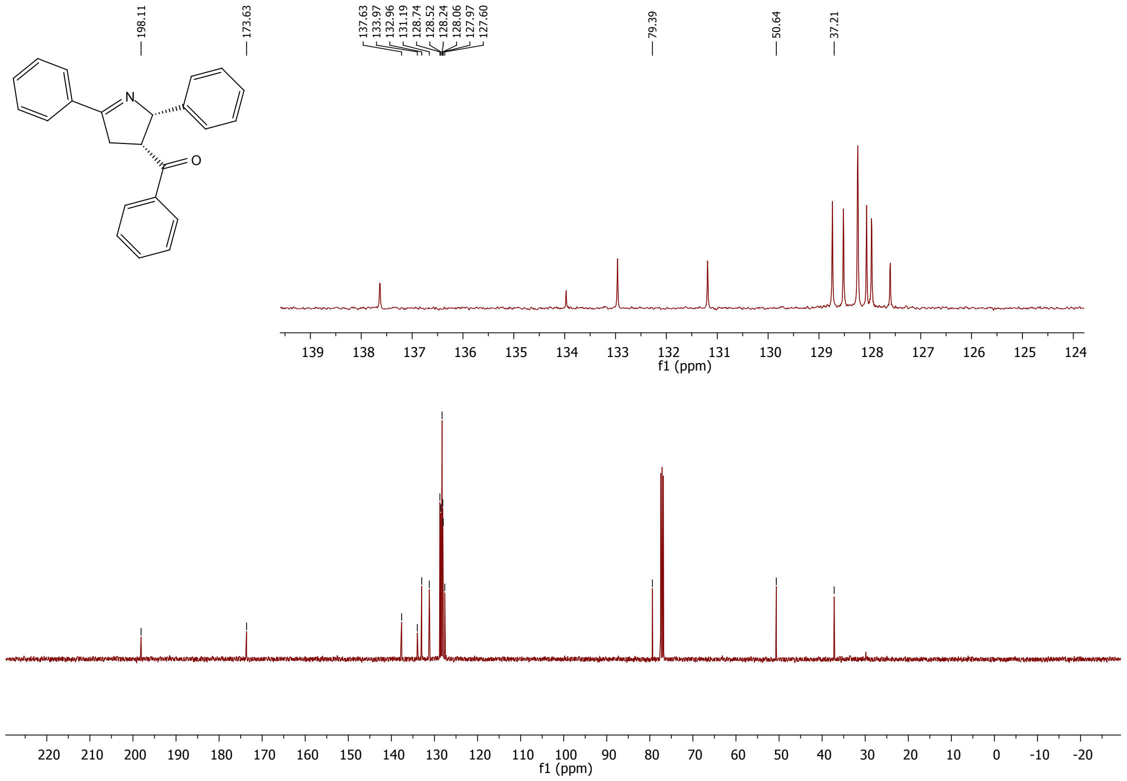

Figure S18. ^{13}**C{**^{1}**H} NMR** (100 MHz, $CDCl_3$) of compound *cis*-**3a.**

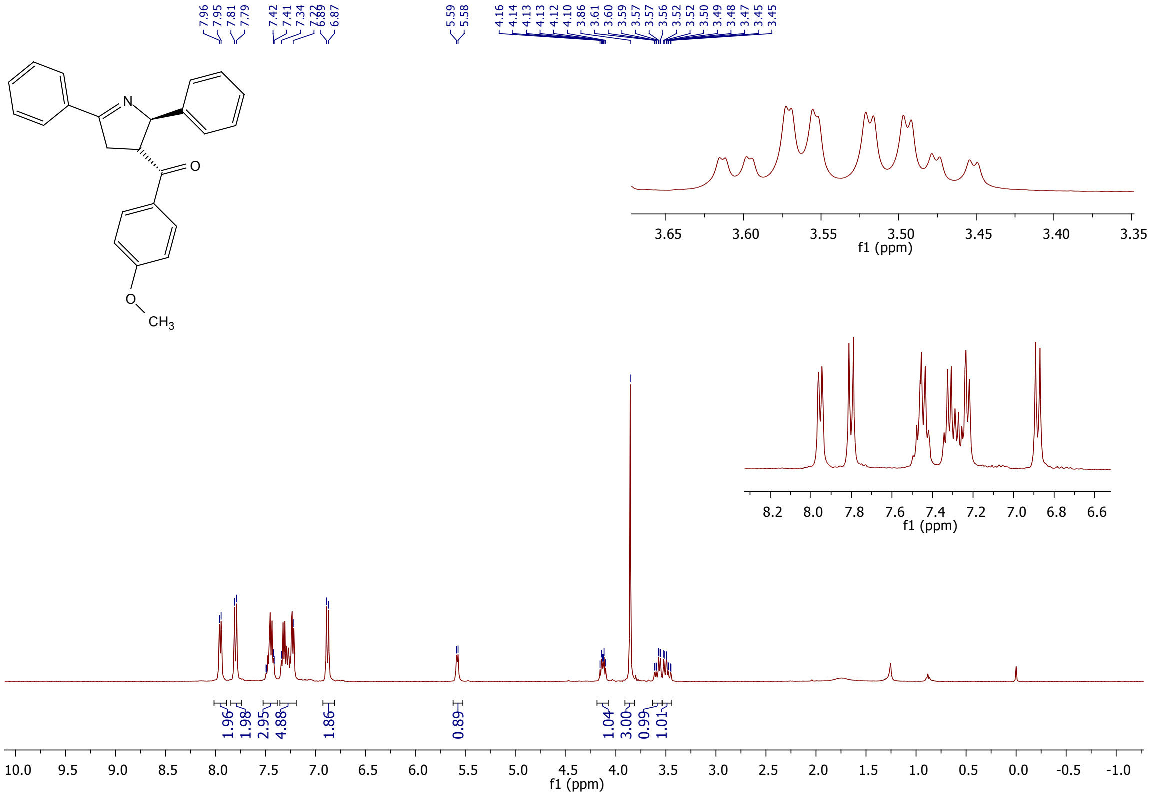

Figure S19.^{1}H NMR (400 MHz, $CDCl_3$) of compound *trans*-**3b.**

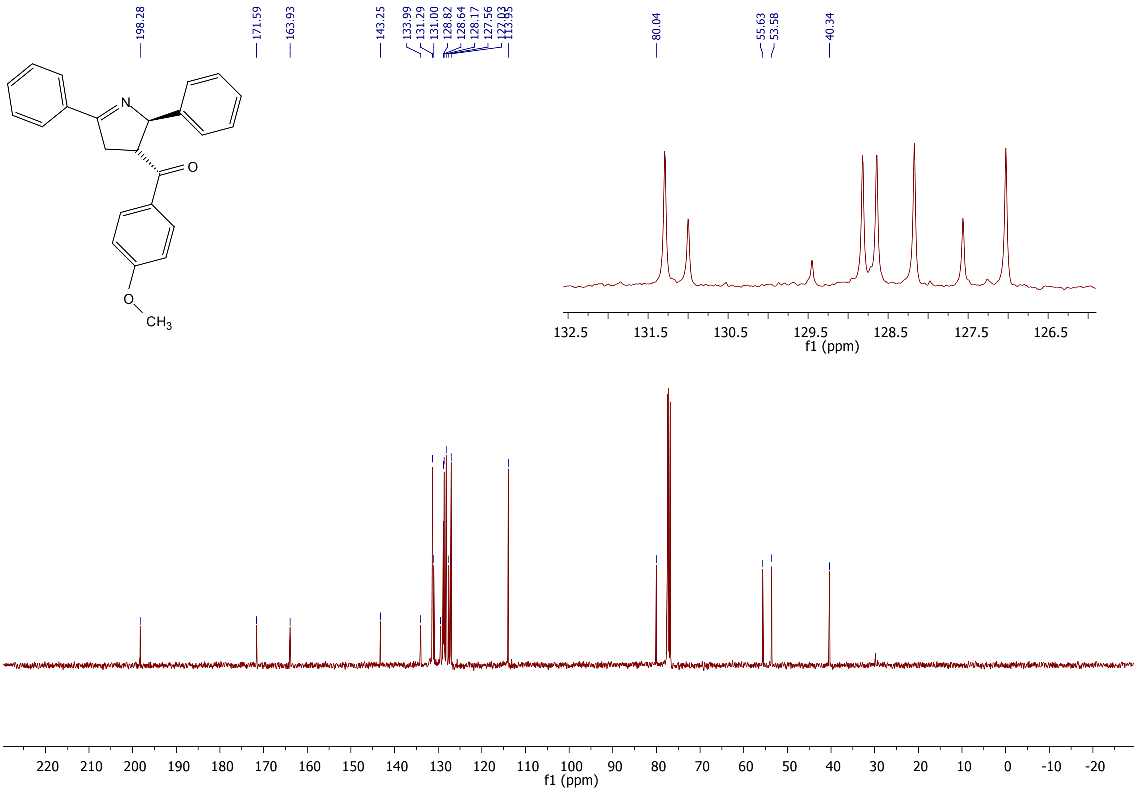

Figure S20. ^{13}C{^{1}H} NMR (100 MHz, $CDCl_3$) of compound *trans*-**3b**.

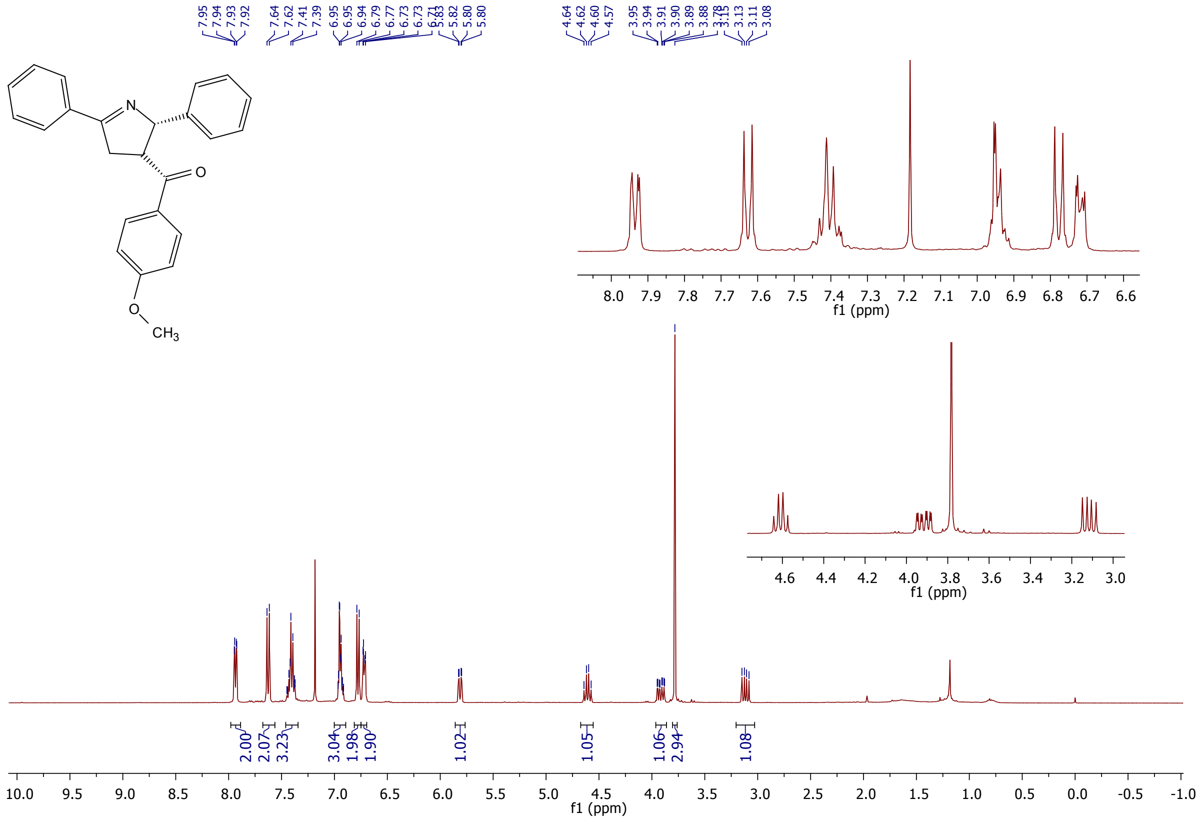

Figure S21. ^{1}H NMR (400 MHz, $CDCl_3$) of compound *cis*-**3b.**

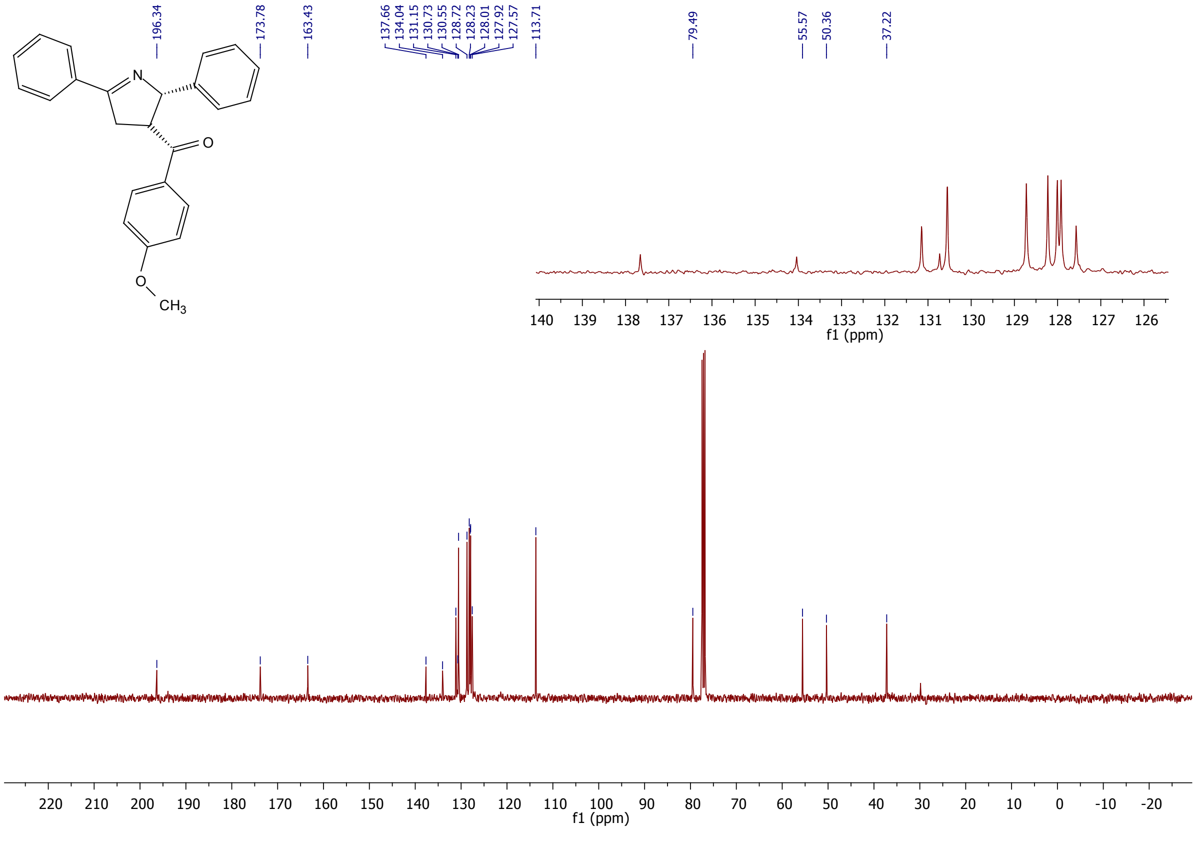

Figure S22. ^{13}C{^{1}H} NMR (100 MHz, $CDCl_3$) of compound *cis*-**3b**.

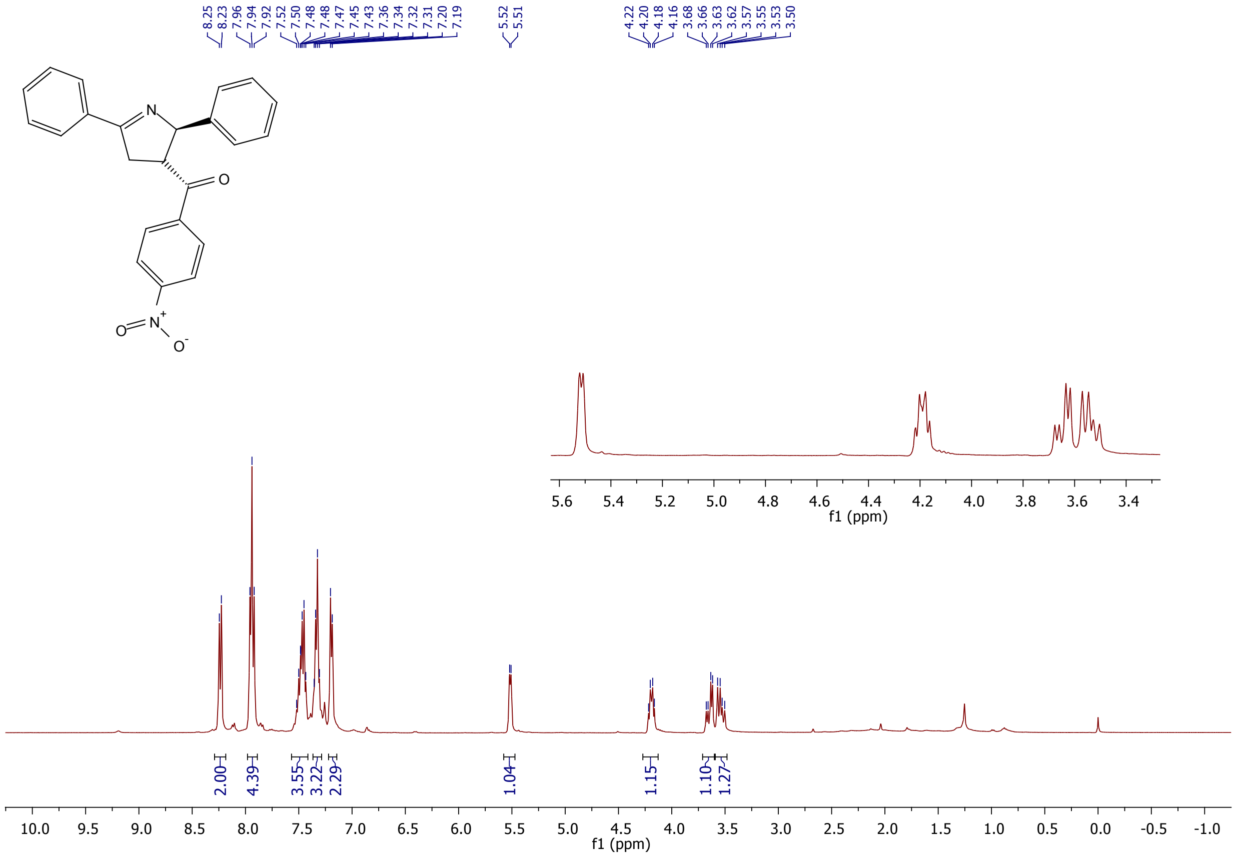

Figure S23. **^{1}H NMR** (400 MHz, $CDCl_3$) of compound *trans*-**3c.**

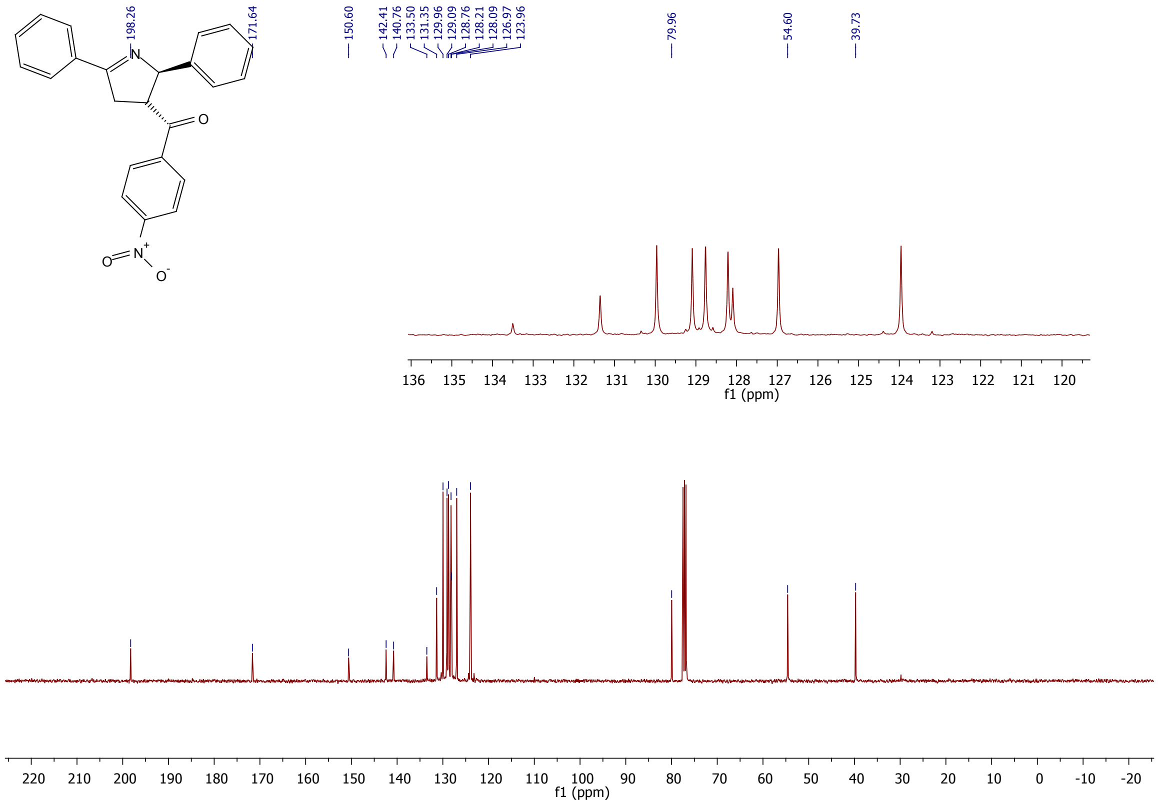

Figure S24. **$^{13}C\{^1H\}$ NMR** (100 MHz, $CDCl_3$) of compound *trans*-**3c.**

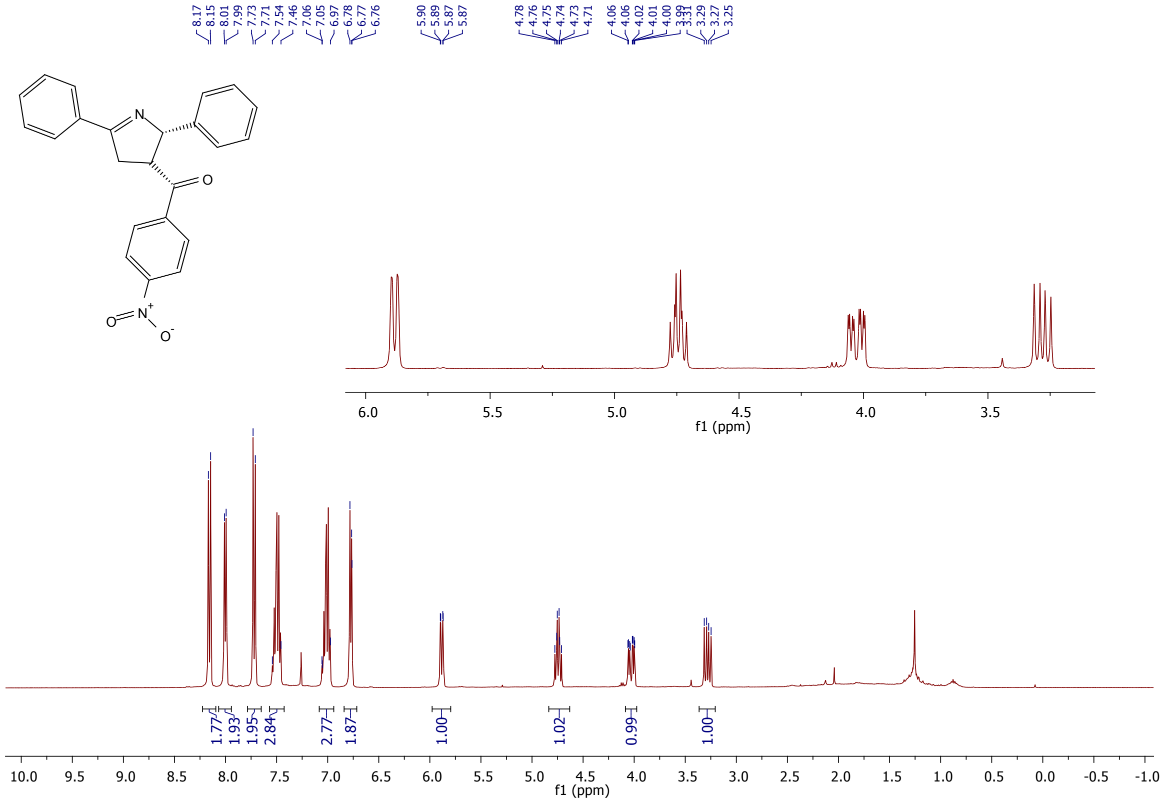

Figure S25. ^{1}H NMR (400 MHz, $CDCl_3$) of compound *cis*-**3c.**

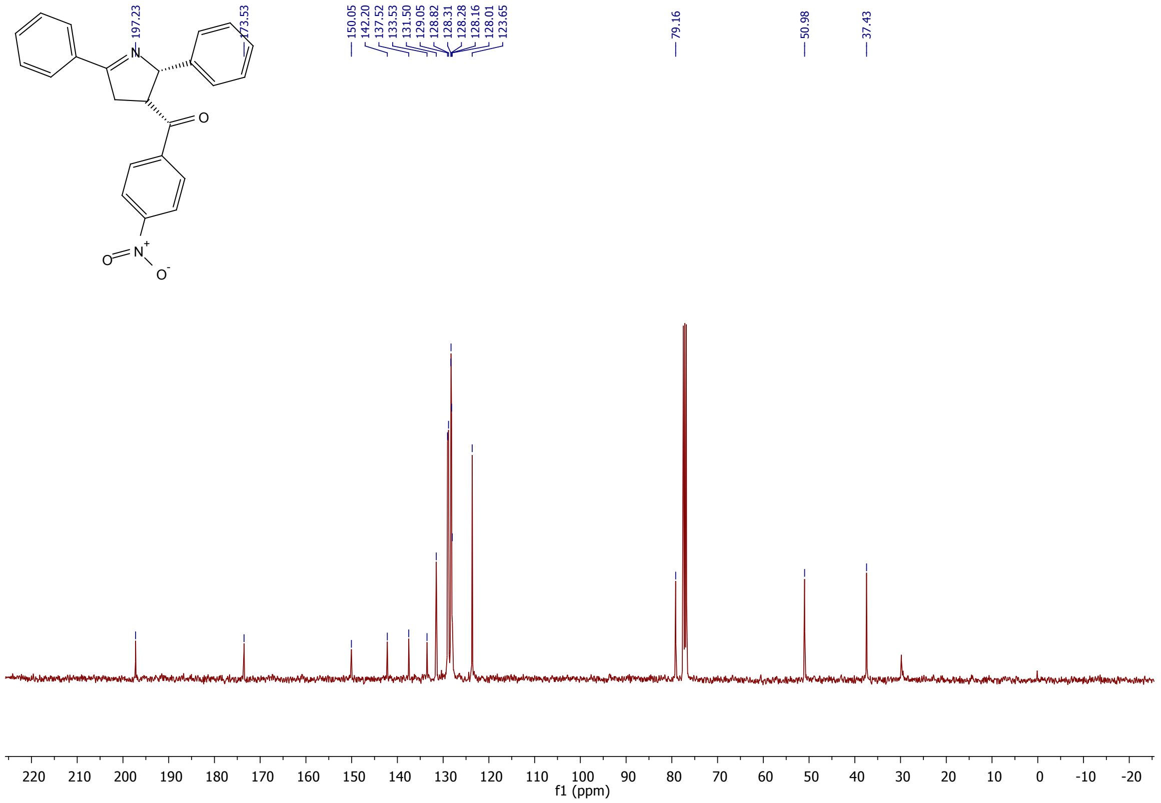

Figure S26. $^{13}C\{^{1}H\}$ NMR (100 MHz, $CDCl_3$) of compound *cis*-**3c.**

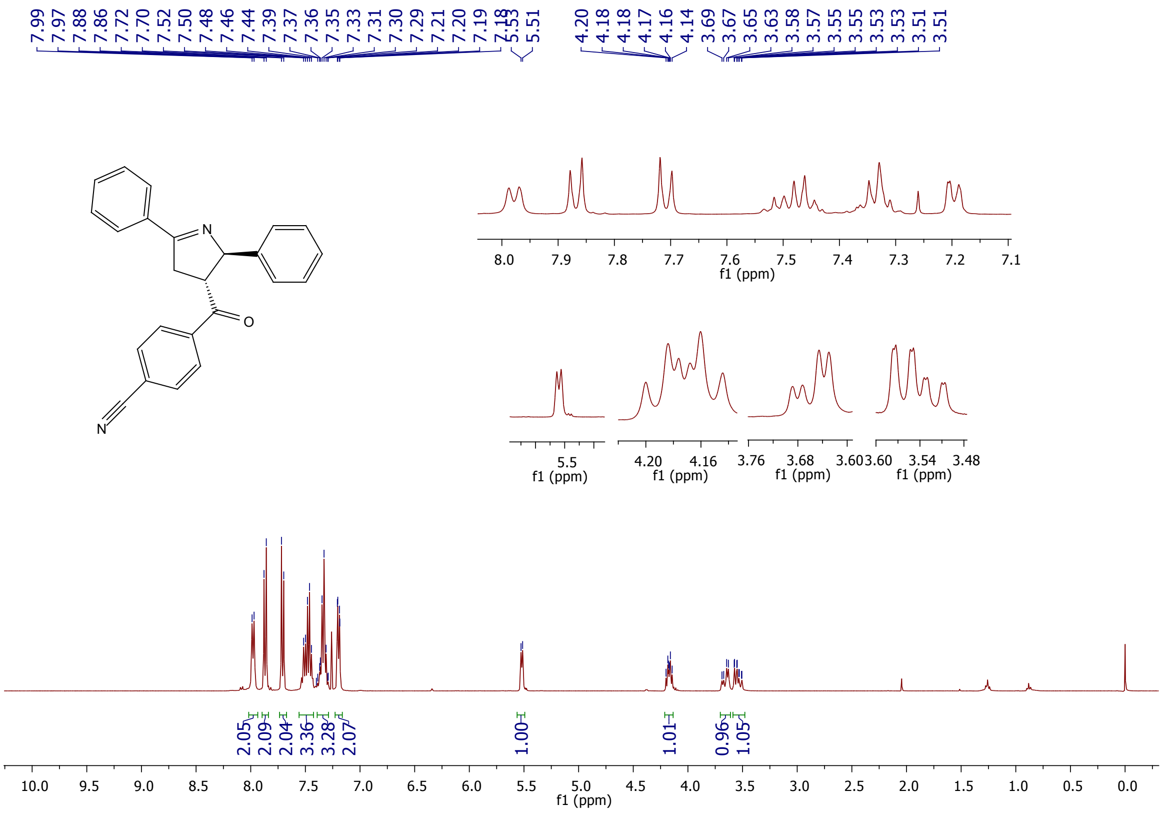

Figure S27. **^{1}H NMR** (400 MHz, $CDCl_3$) of compound *trans*-**3d.**

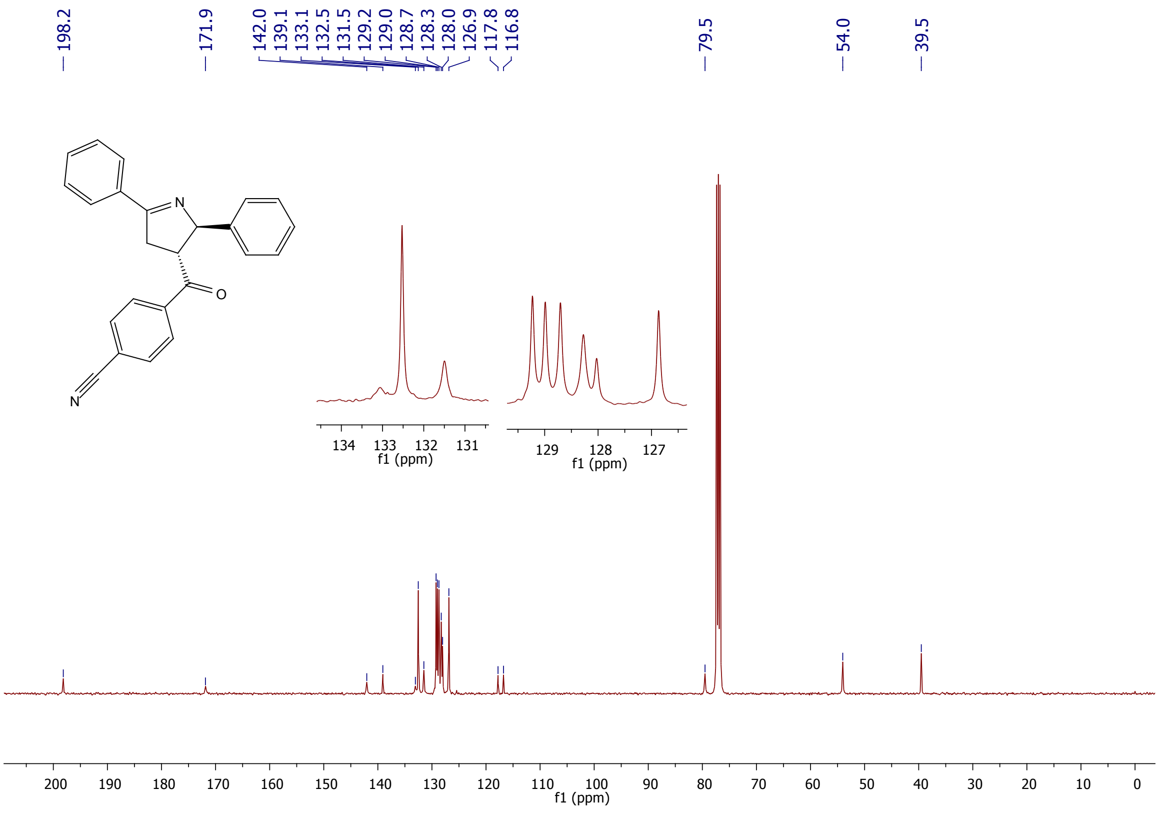

Figure S28. **$^{13}C\{^1H\}$ NMR** (100 MHz, $CDCl_3$) of compound *trans*-**3d.**

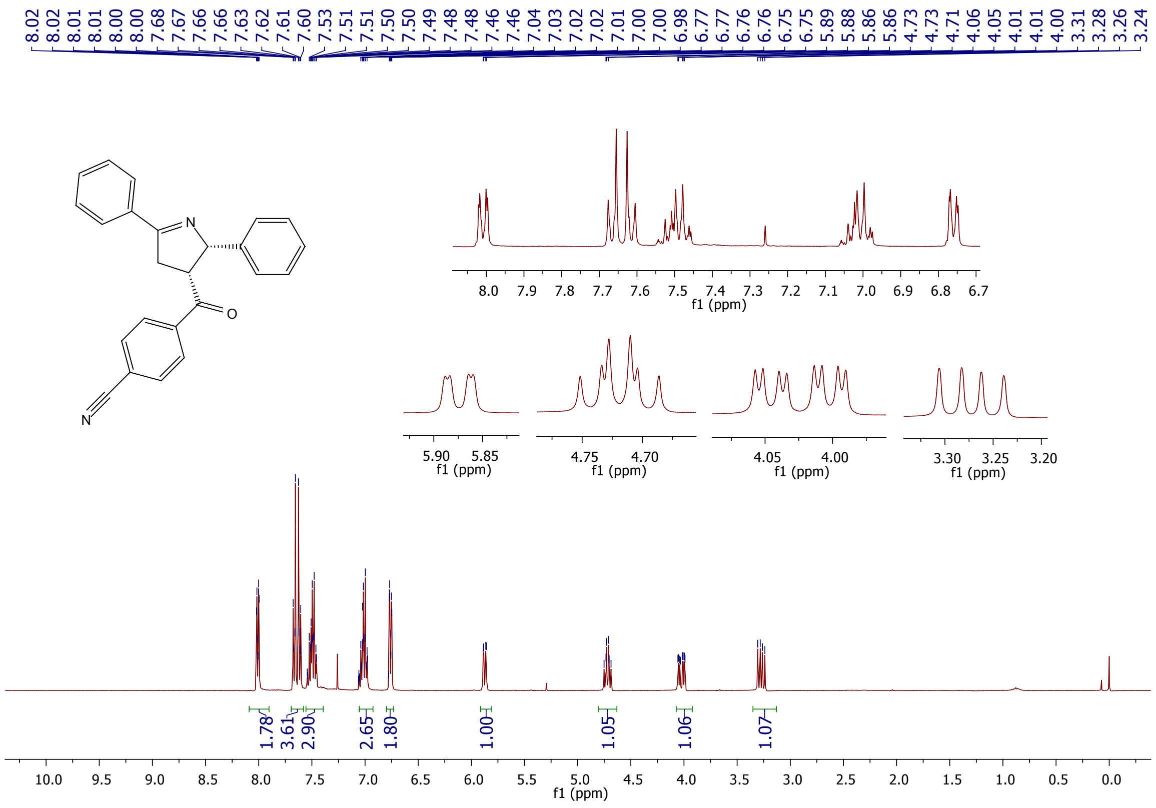

Figure S29. **^{1}H NMR** (400 MHz, $CDCl_3$) of compound *cis*-**3d.**

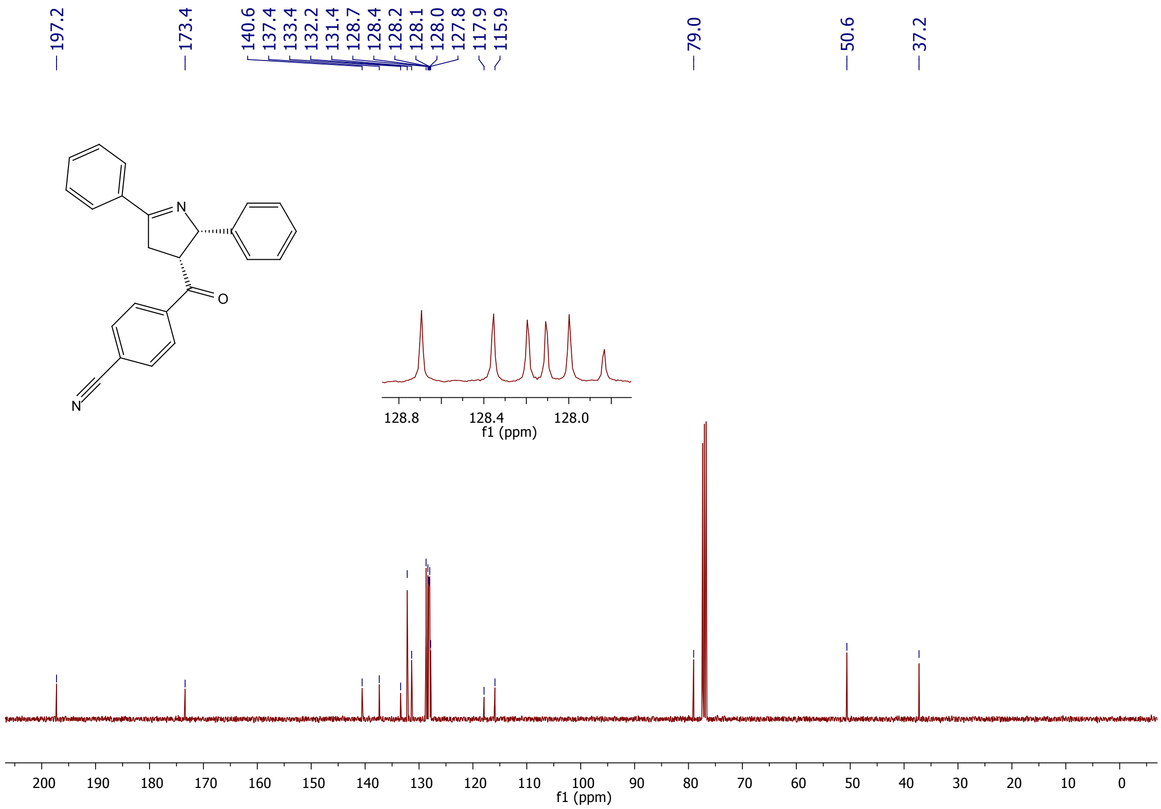

Figure S30. **$^{13}C\{^1H\}$ NMR** (100 MHz, $CDCl_3$) of compound *cis*-**3d.**

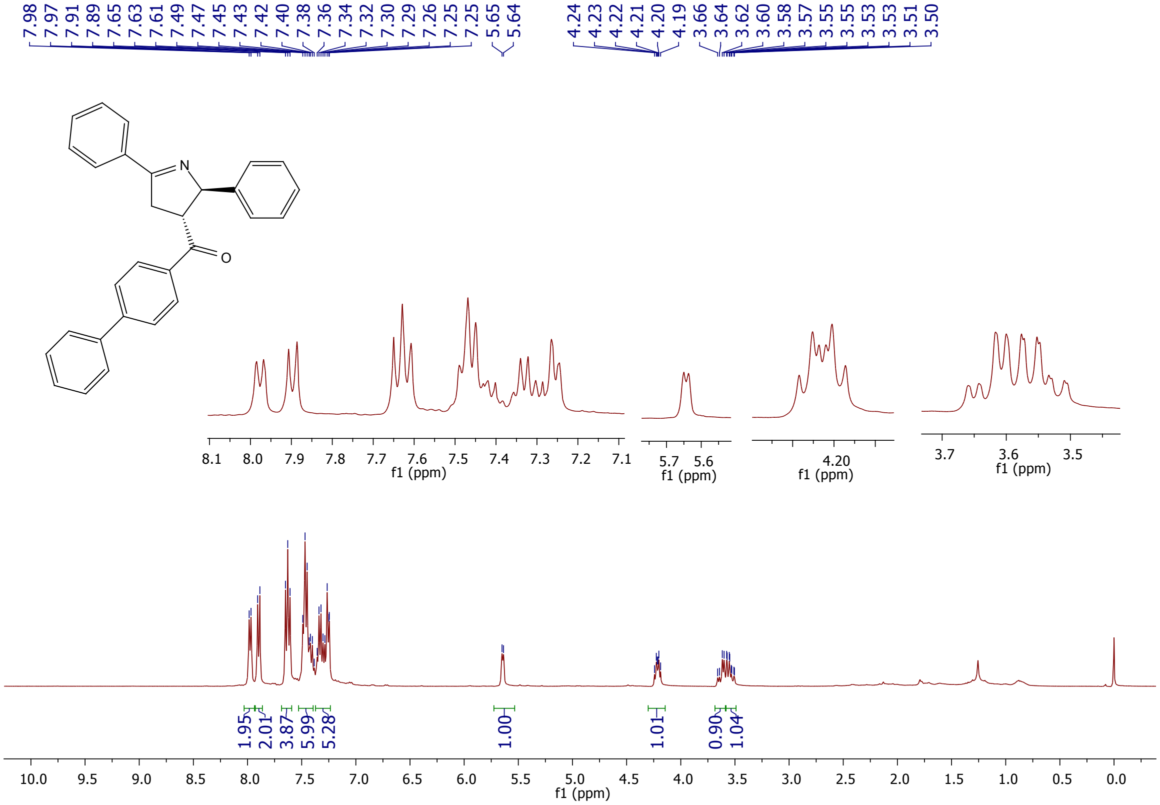

Figure S31. **^{1}H NMR** (400 MHz, $CDCl_3$) of compound *trans*-**3e.**

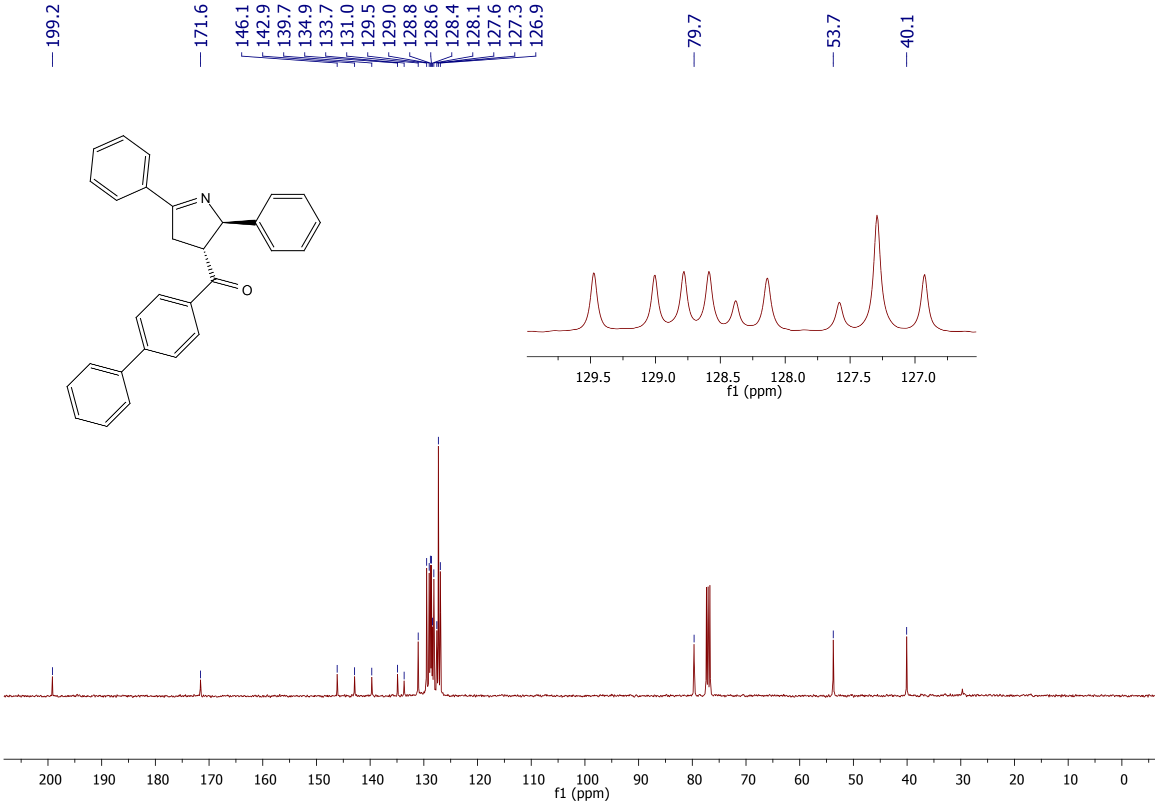

Figure S32. **^{13}C{^{1}H} NMR** (100 MHz, $CDCl_3$) of compound *trans*-**3e.**

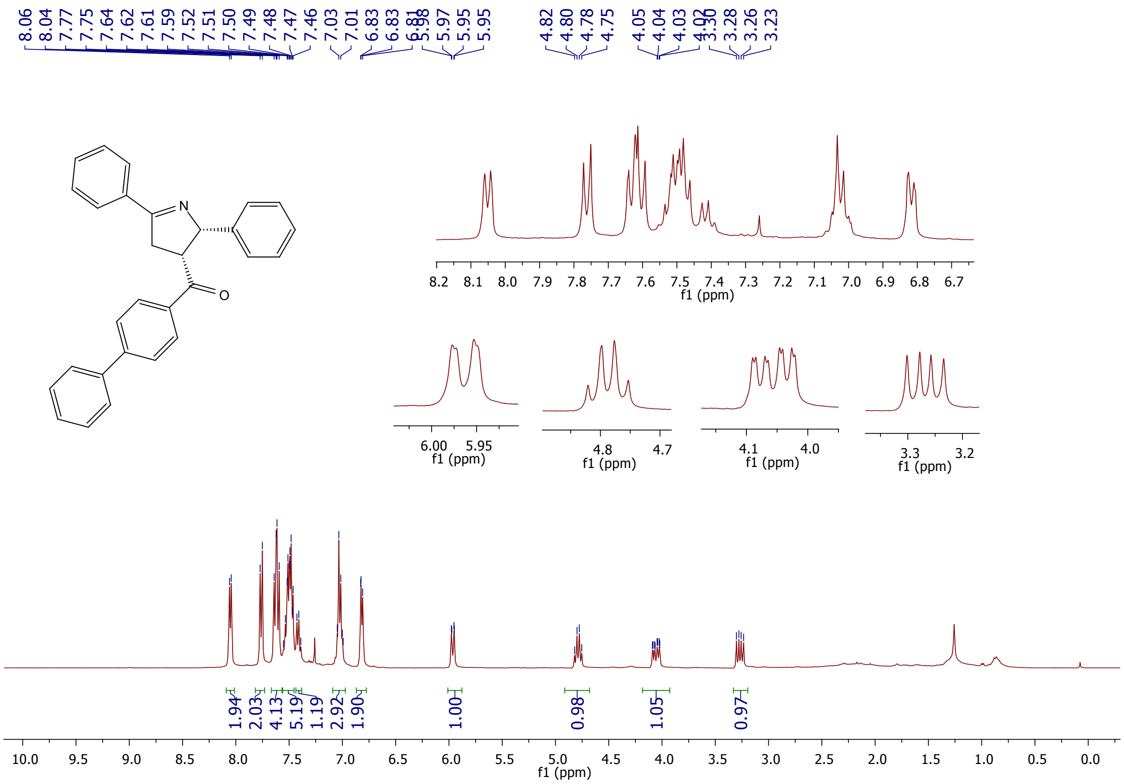

Figure S33. ^{1}H NMR (400 MHz, $CDCl_3$) of compound *cis*-**3e.**

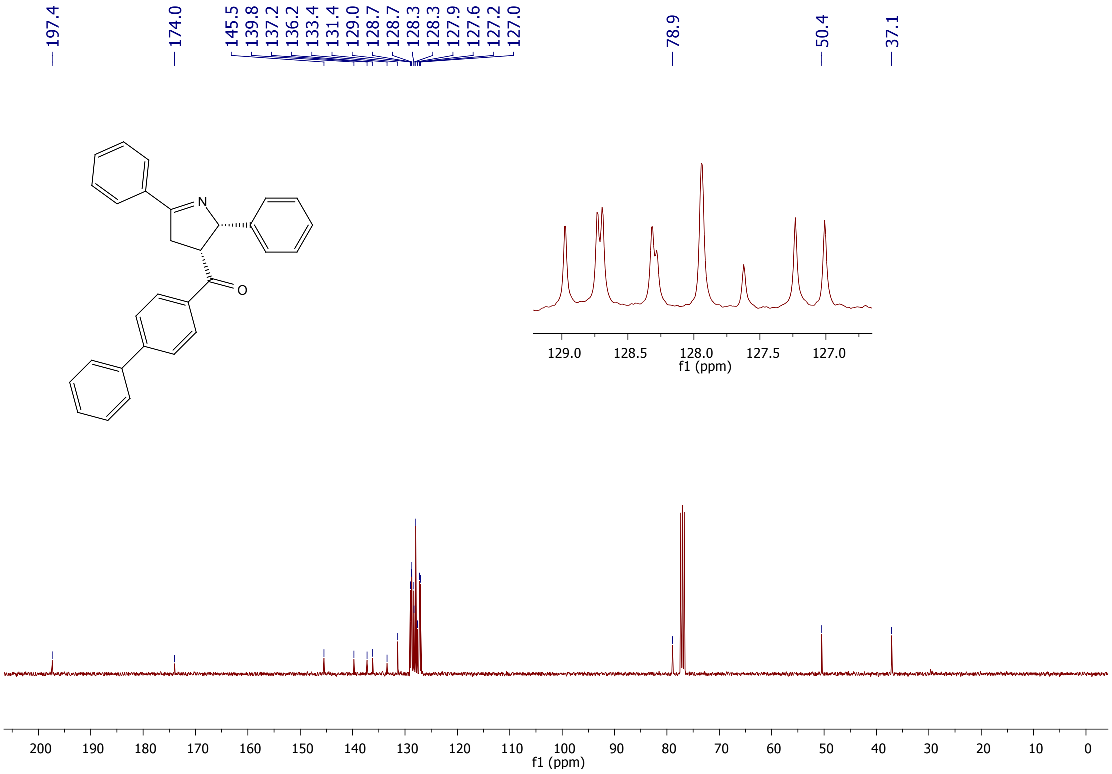

Figure S34. $^{13}C\{^{1}H\}$ NMR (100 MHz, $CDCl_3$) of compound *cis*-**3e.**

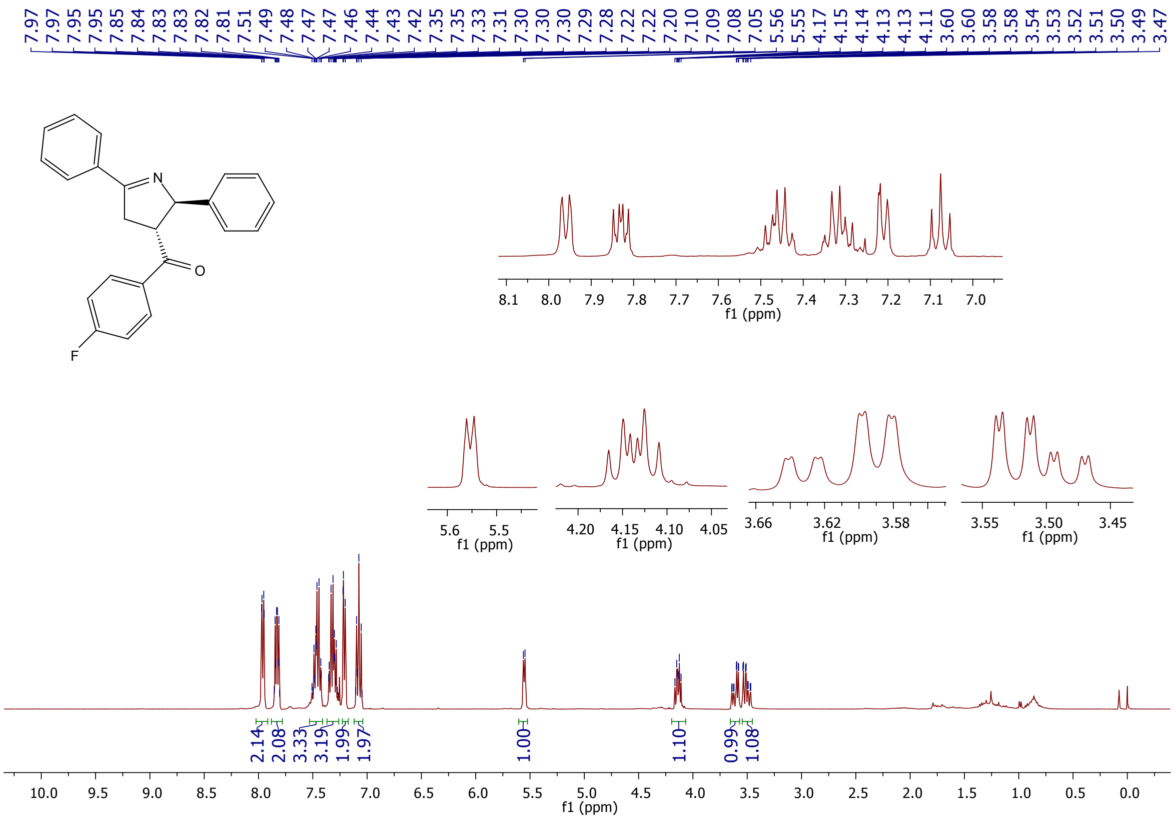

Figure S35. ¹H NMR (400 MHz, $CDCl_3$) of compound *trans*-**3f.**

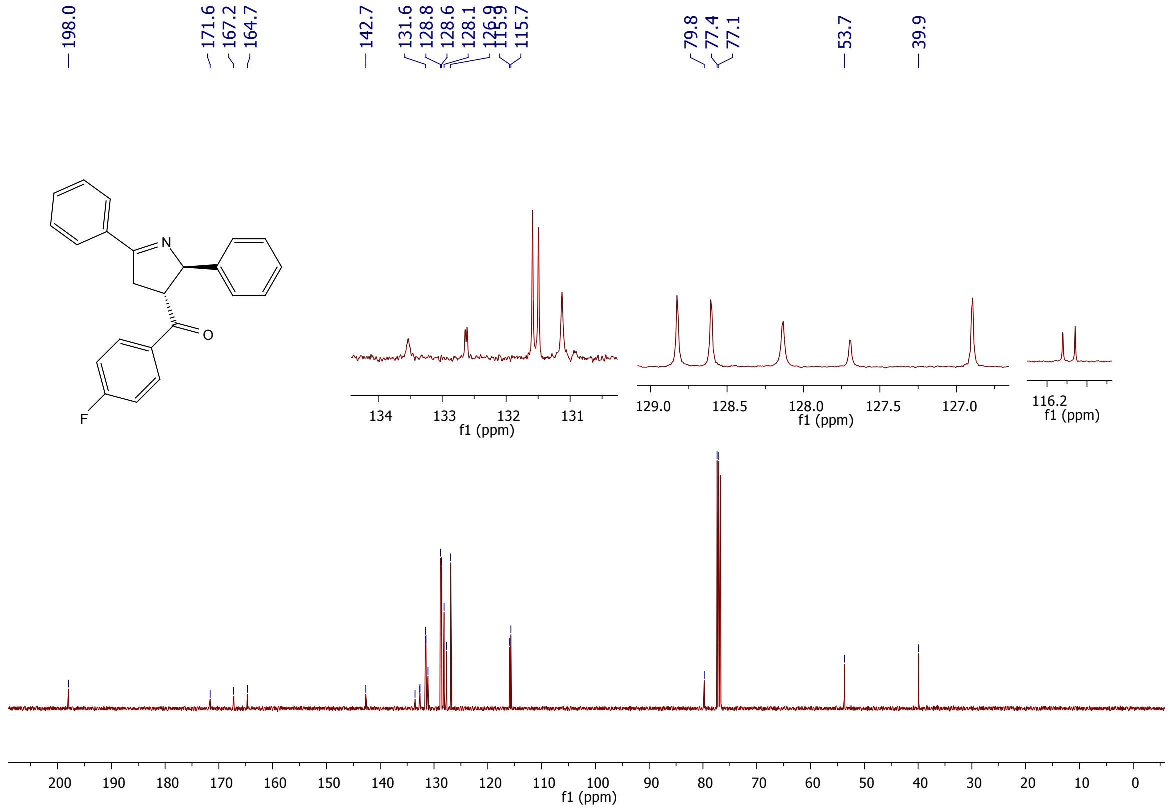

Figure S36. $^{13}C\{^1H\}$ NMR (100 MHz, $CDCl_3$) of compound *trans*-**3f.**

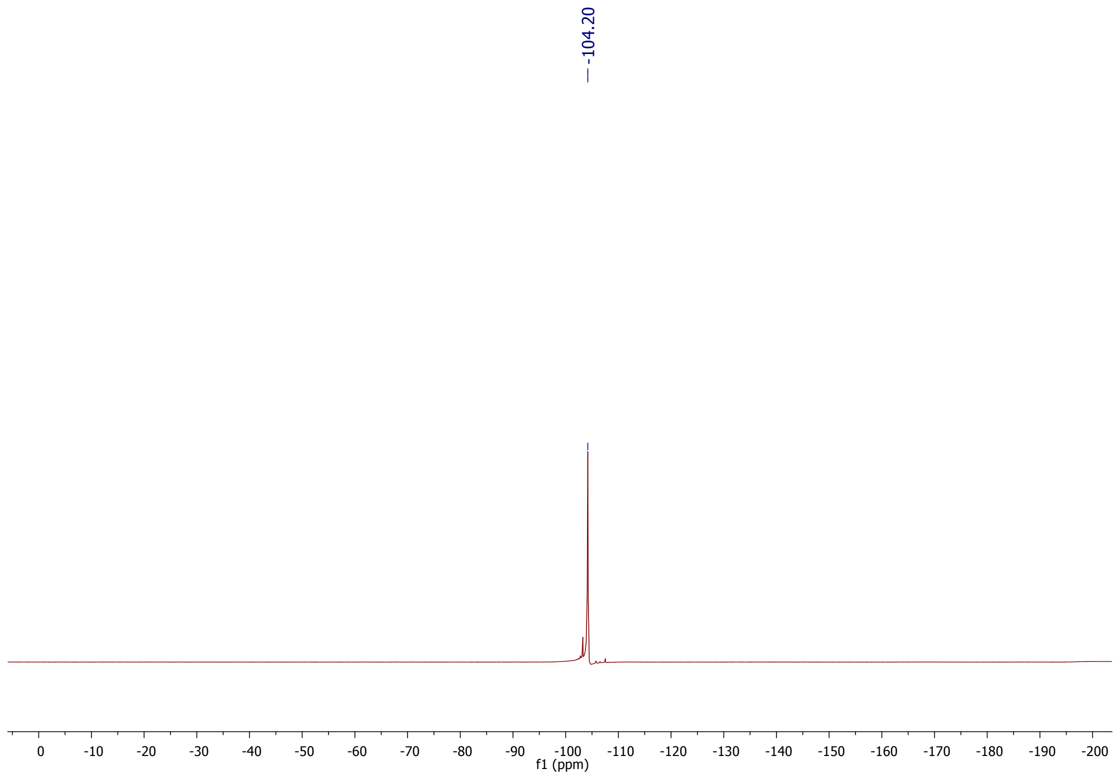

Figure S37. **^{19}F NMR** (376 MHz, $CDCl_3$) of compound *trans*-**3f.**

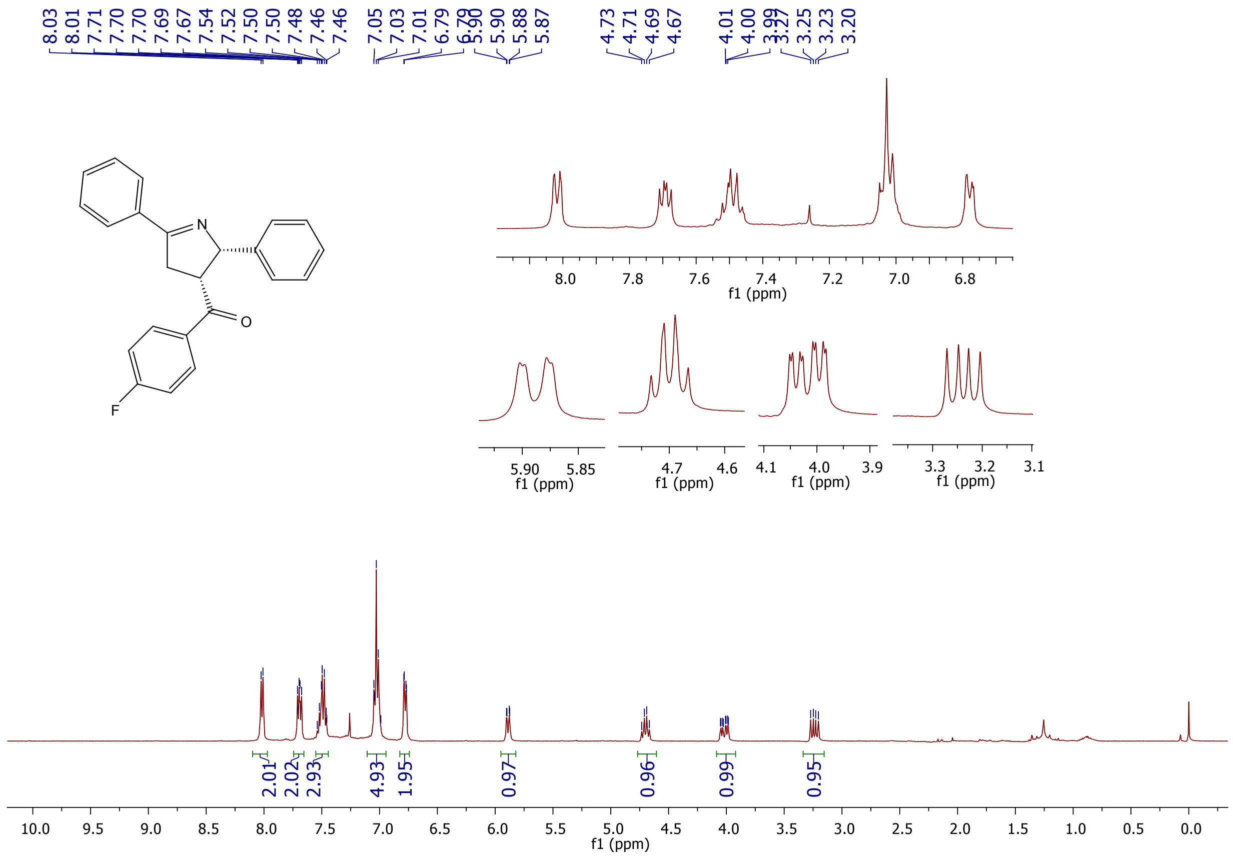

Figure S38. **^{1}H NMR** (400 MHz, $CDCl_3$) of compound *cis*-**3f.**

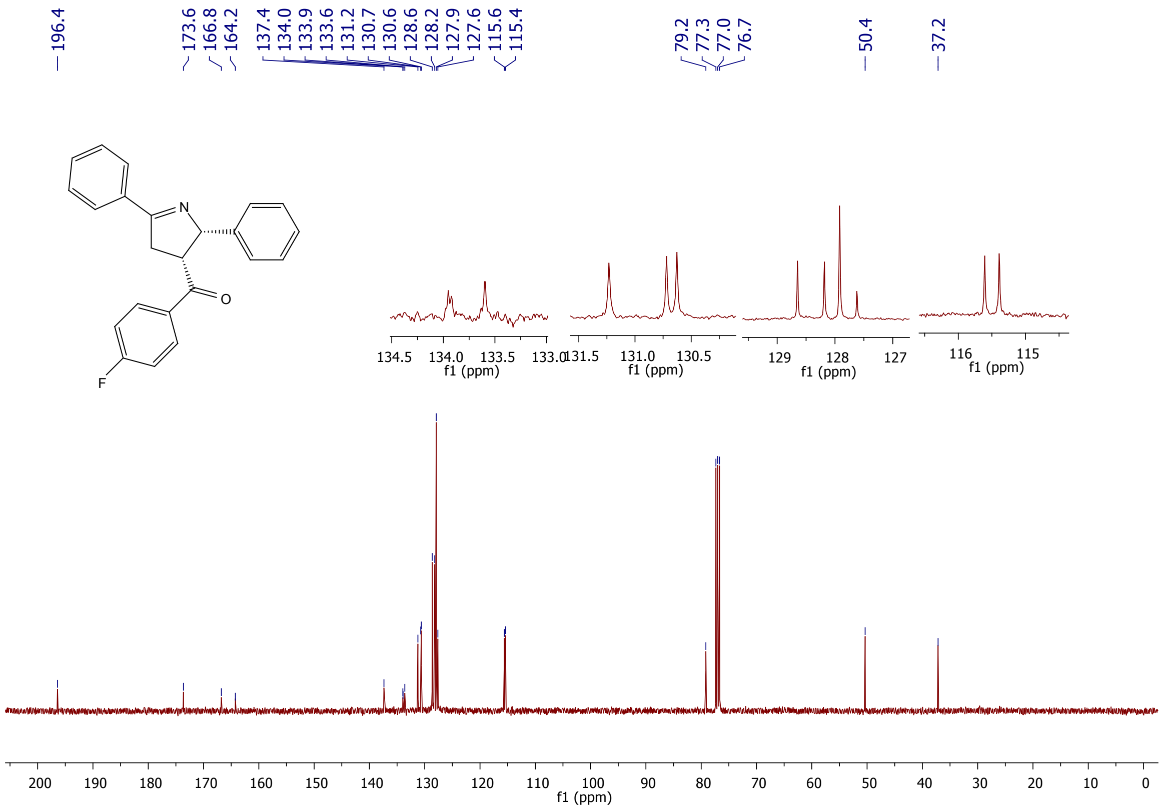

Figure S39. ^{13}**C{**^{1}**H} NMR (100 MHz**, $CDCl_3$) of compound *cis*-**3f.**

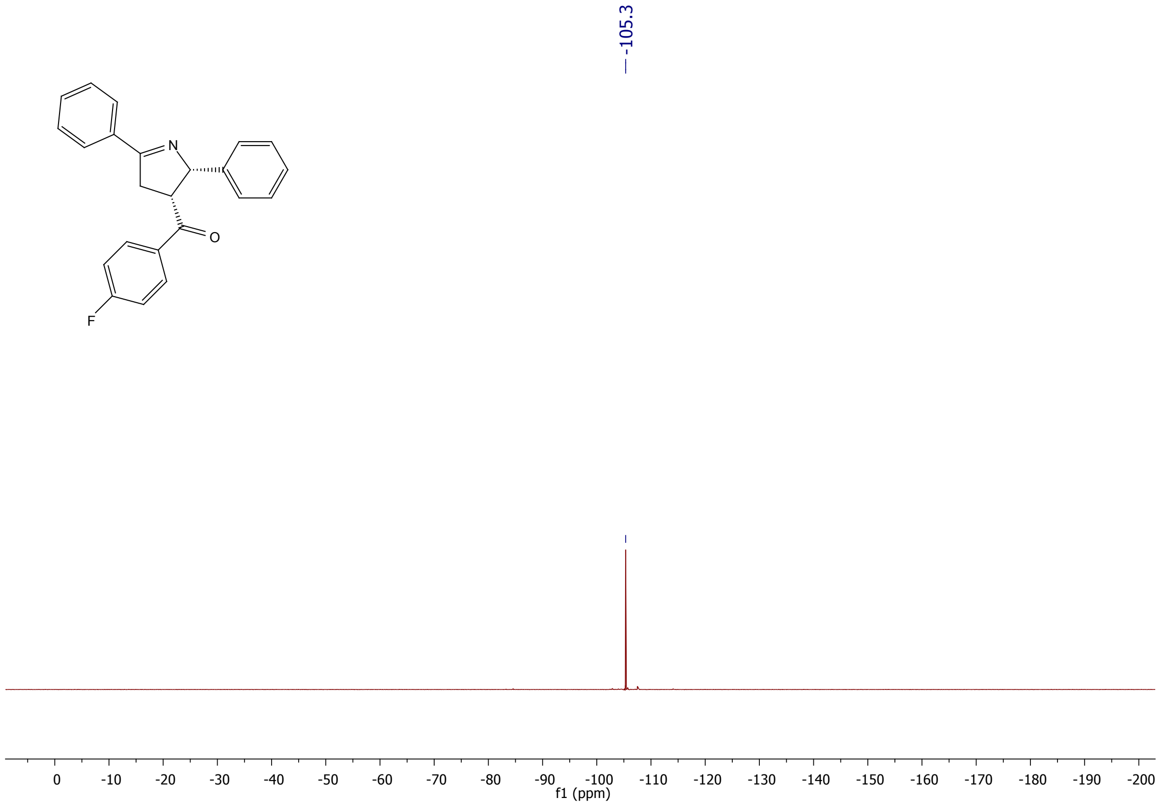

Figure S40. ^{19}**F NMR** (376 MHz, $CDCl_3$) of compound *cis*-**3f.**

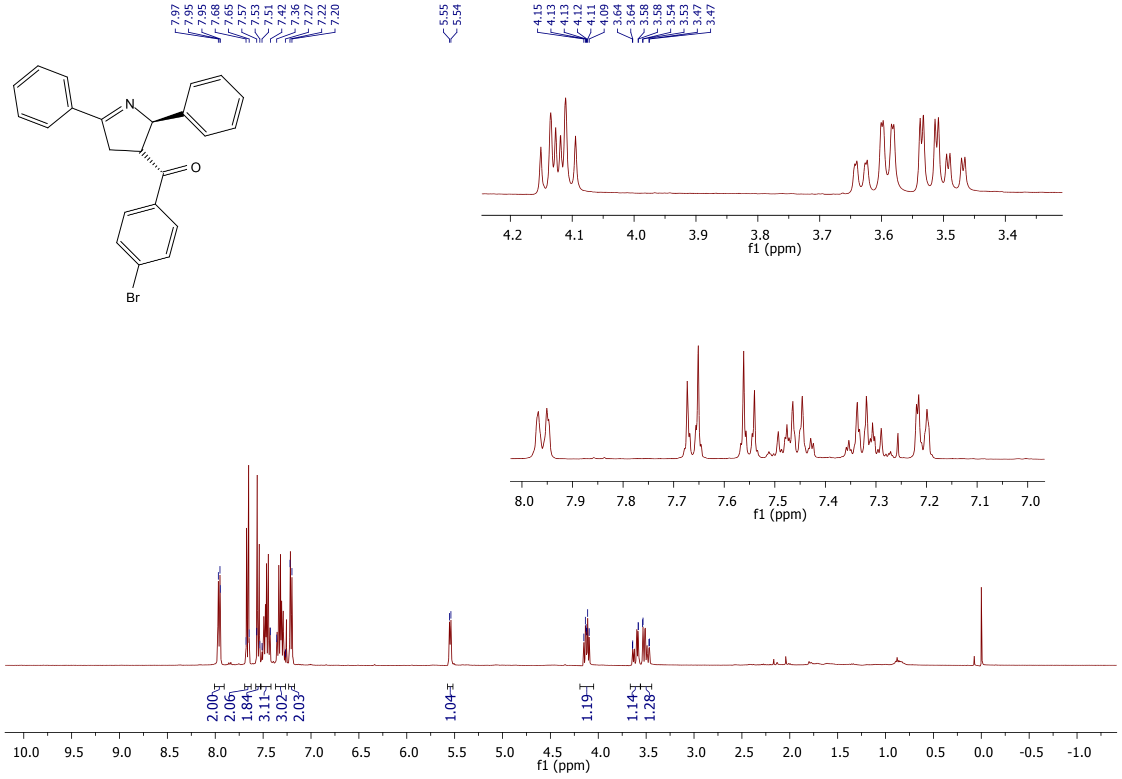

Figure S41. **^{1}H NMR** (400 MHz, $CDCl_3$) of compound *trans*-**3g.**

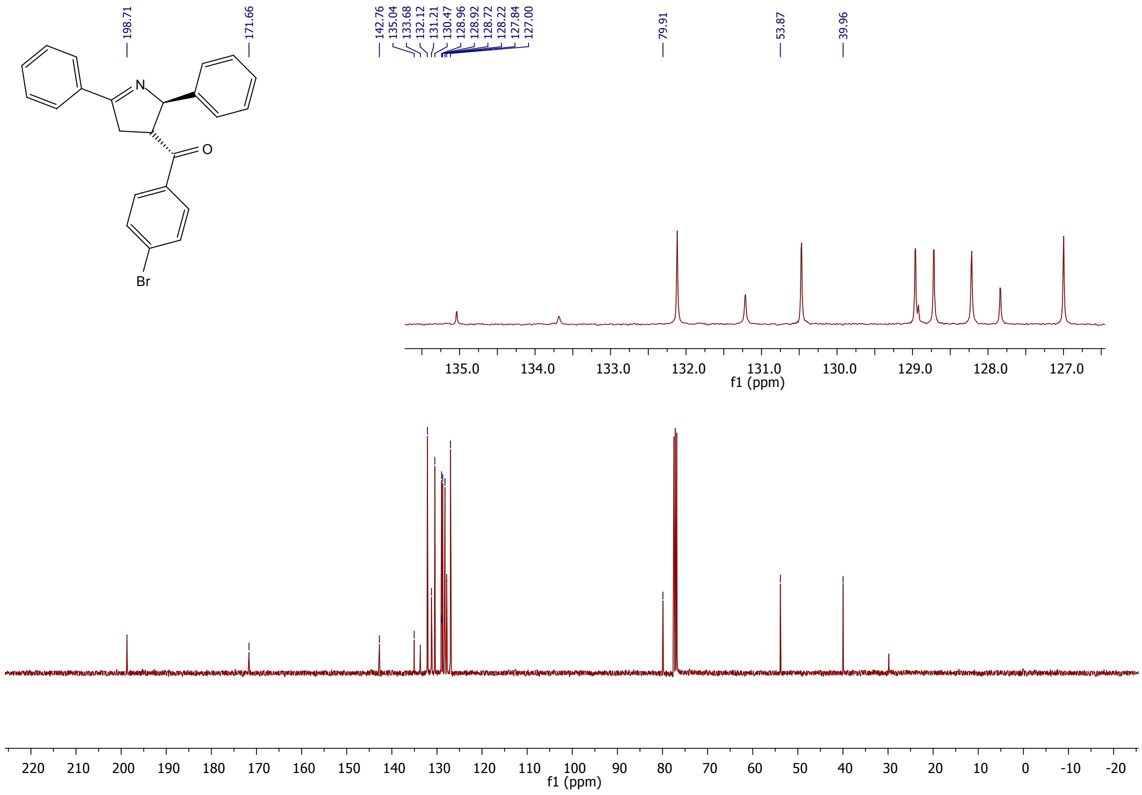

Figure S42. **^{13}C{^{1}H} NMR** (100 MHz, $CDCl_3$) of compound *trans*-**3g.**

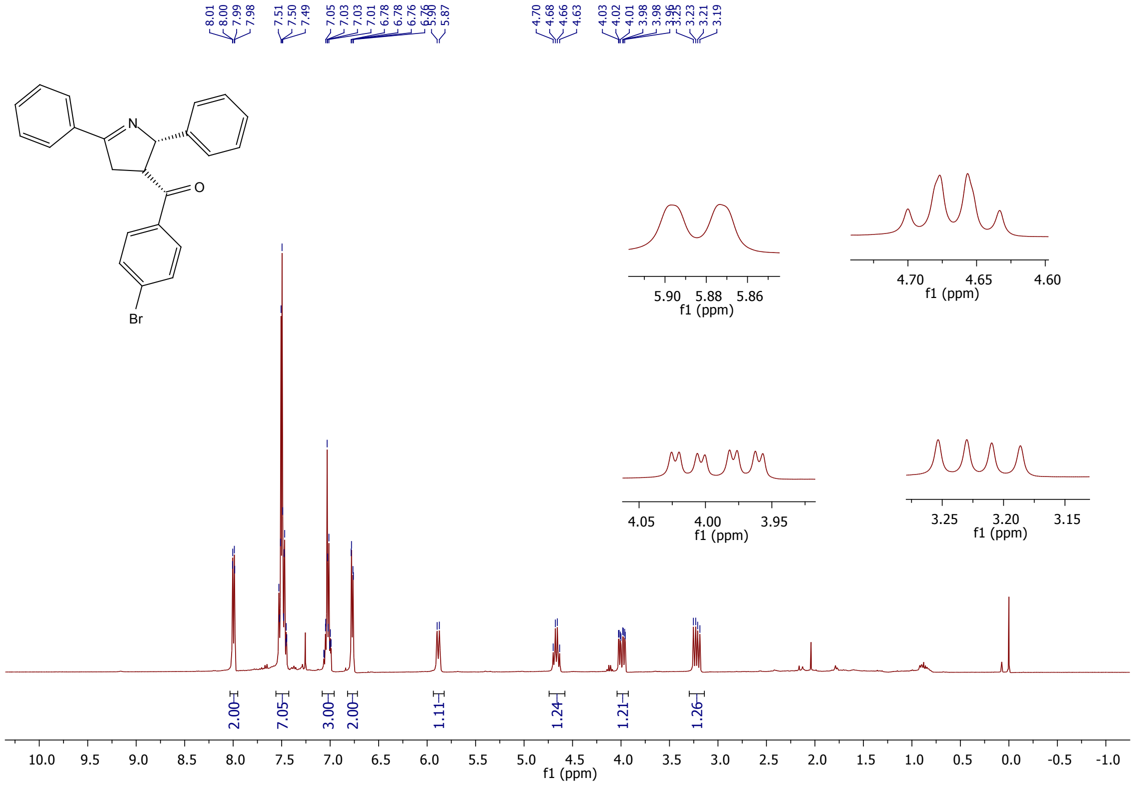

Figure S43. ^{1}H NMR (400 MHz, $CDCl_3$) of compound *cis*-**3g.**

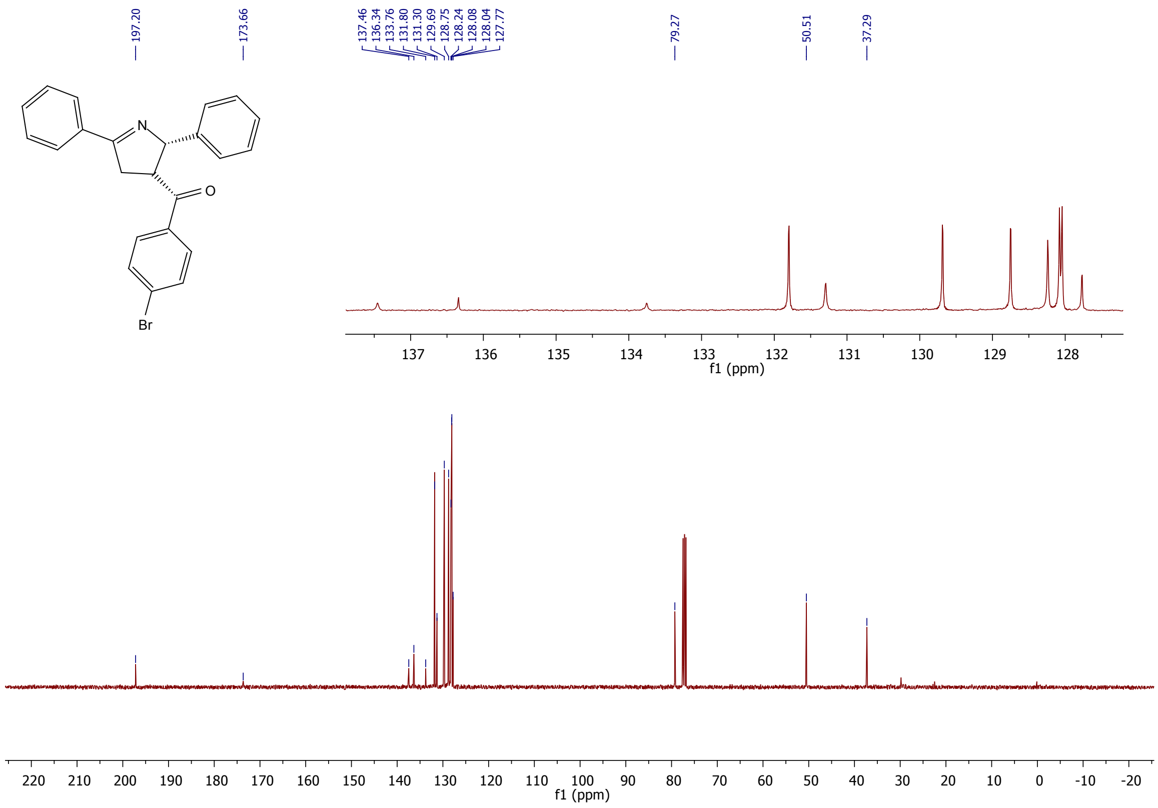

Figure S44. $^{13}C\{^{1}H\}$ NMR (100 MHz, $CDCl_3$) of compound *cis*-**3g.**

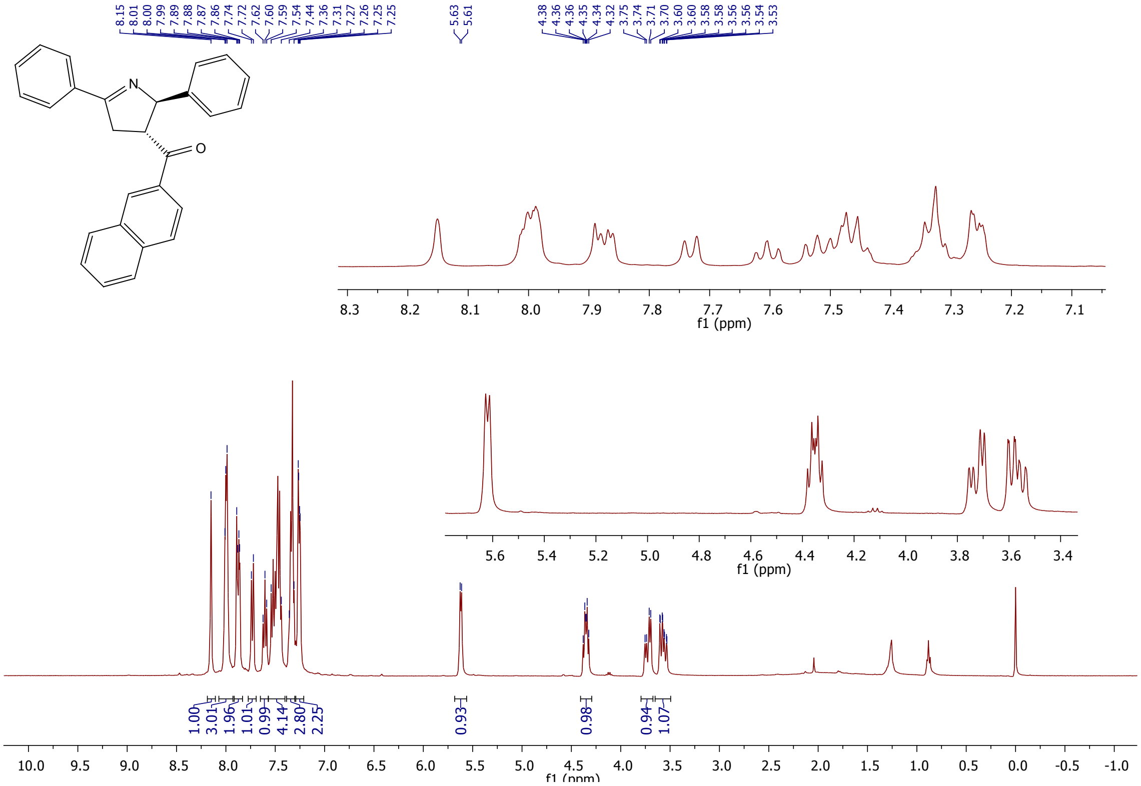

Figure S45. ^{1}H NMR (400 MHz, $CDCl_3$) of compound *trans*-**3h.**

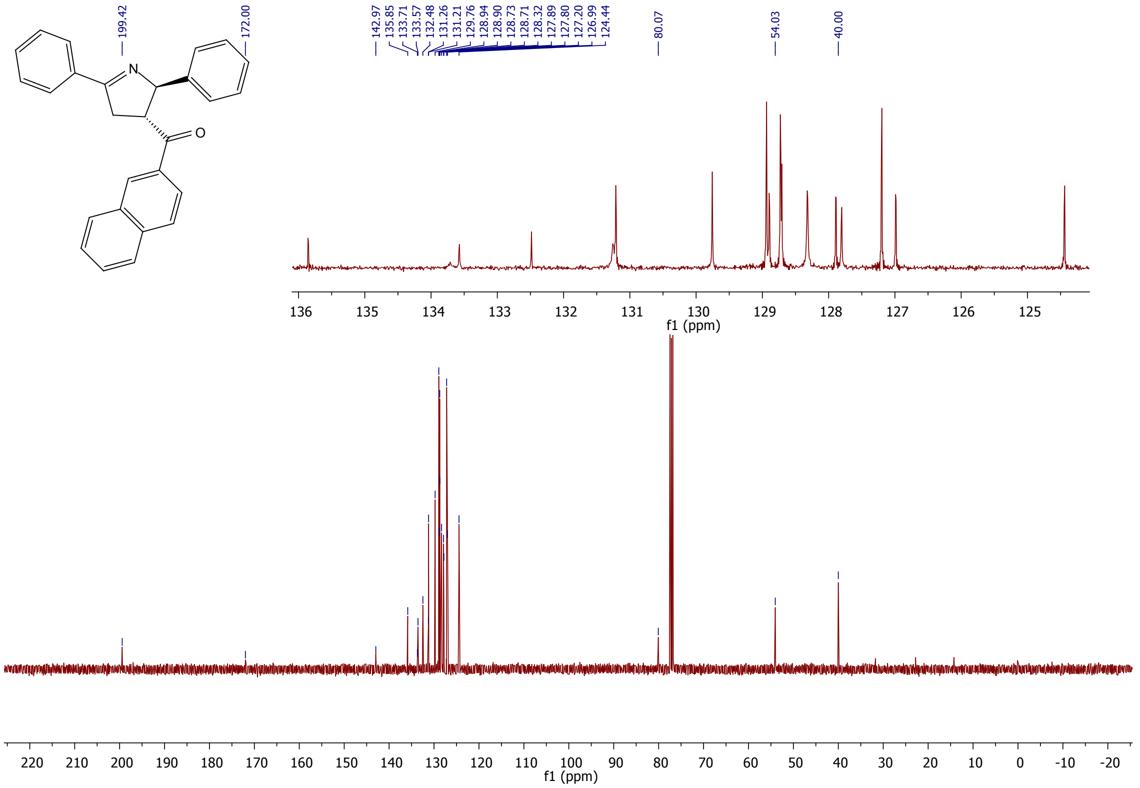

Figure S46.^{13}C{^{1}H} NMR (100 MHz, $CDCl_3$) of compound *trans*-**3h.**

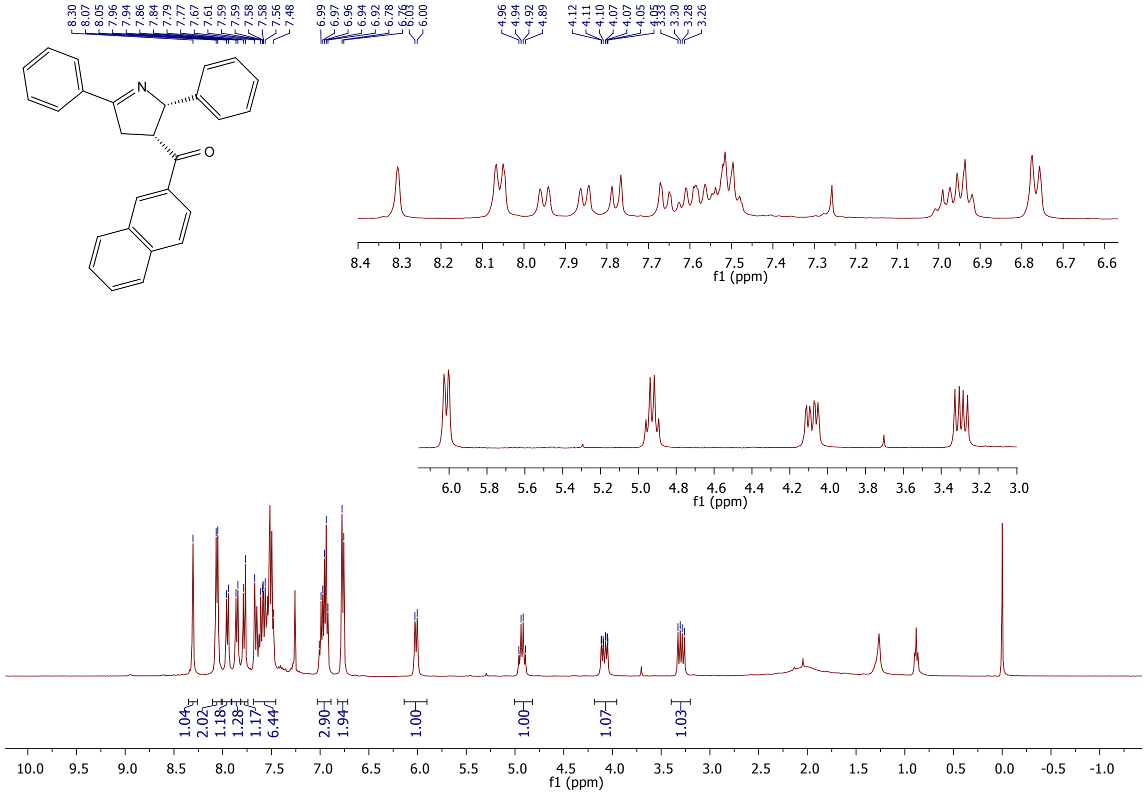

Figure S47. ^{1}H NMR (400 MHz, $CDCl_3$) of compound *cis*-**3h.**

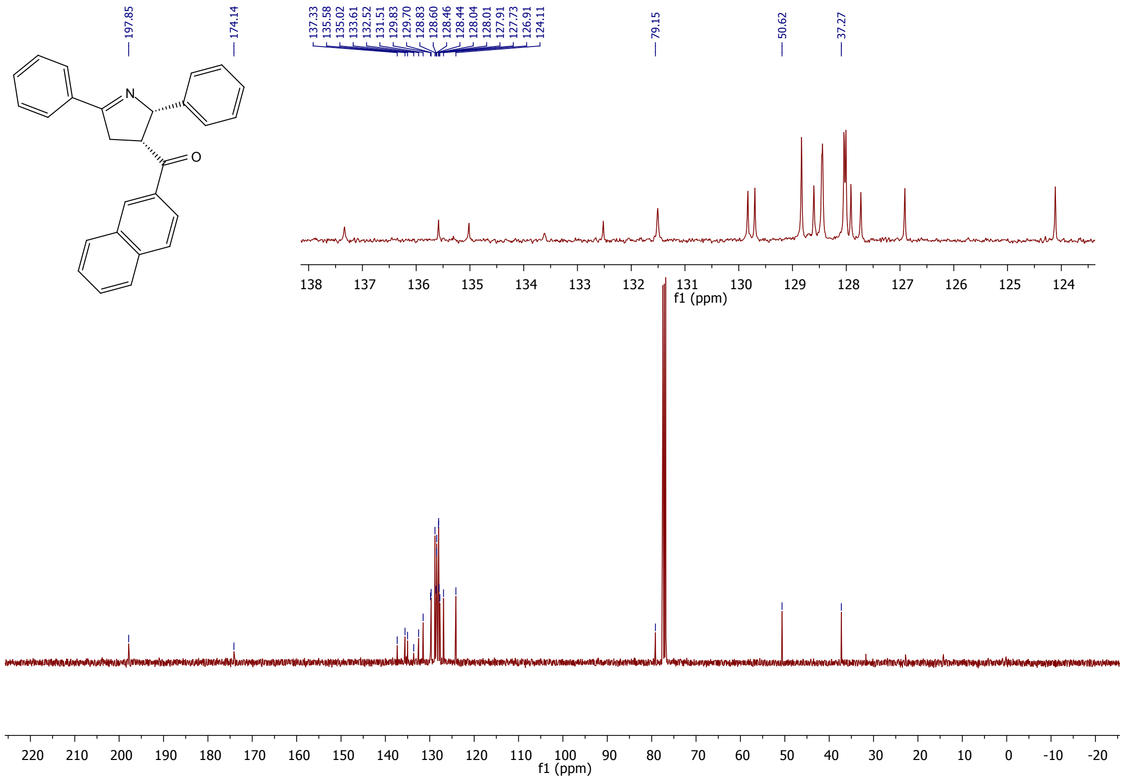

Figure S48. $^{13}C\{^{1}H\}$ NMR (100 MHz, $CDCl_3$) of compound *cis*-**3h.**

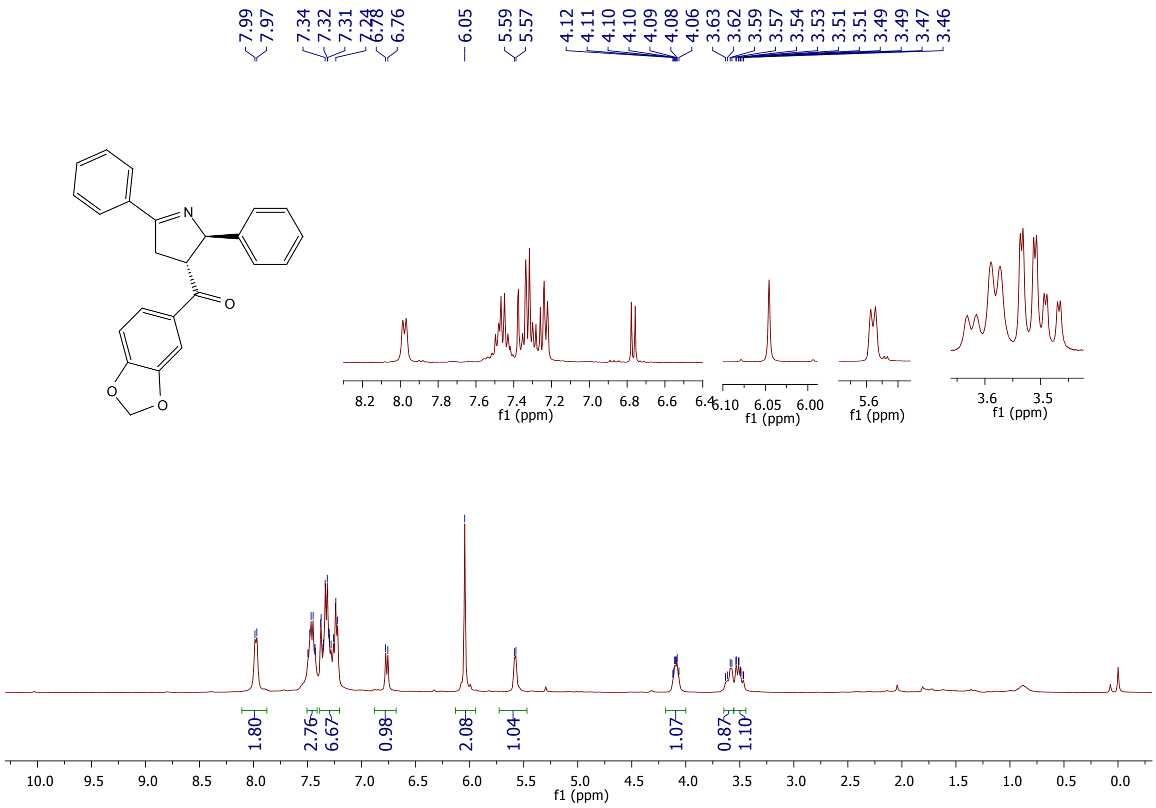

Figure S49. ^{1}H NMR (400 MHz, $CDCl_3$) of compound *trans*-**3i.**

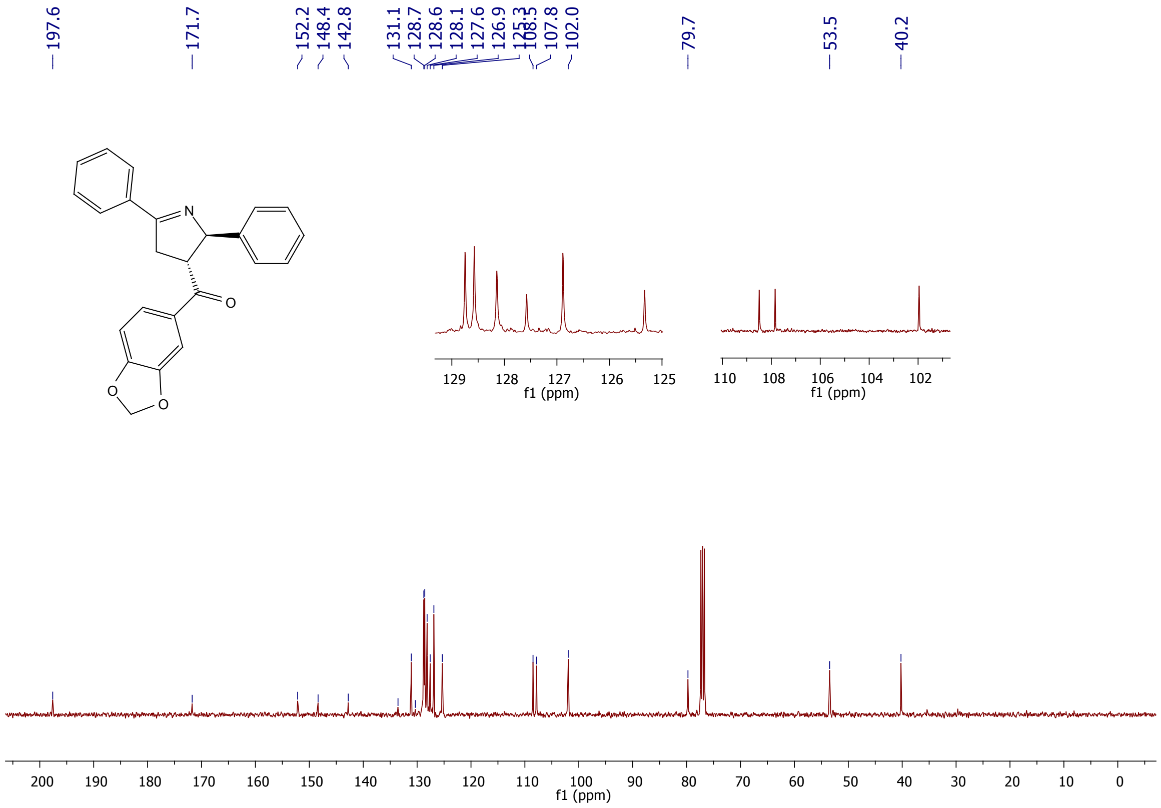

Figure S50. ^{13}C{^{1}H} NMR (100 MHz, $CDCl_3$) of compound *trans*-**3i.**

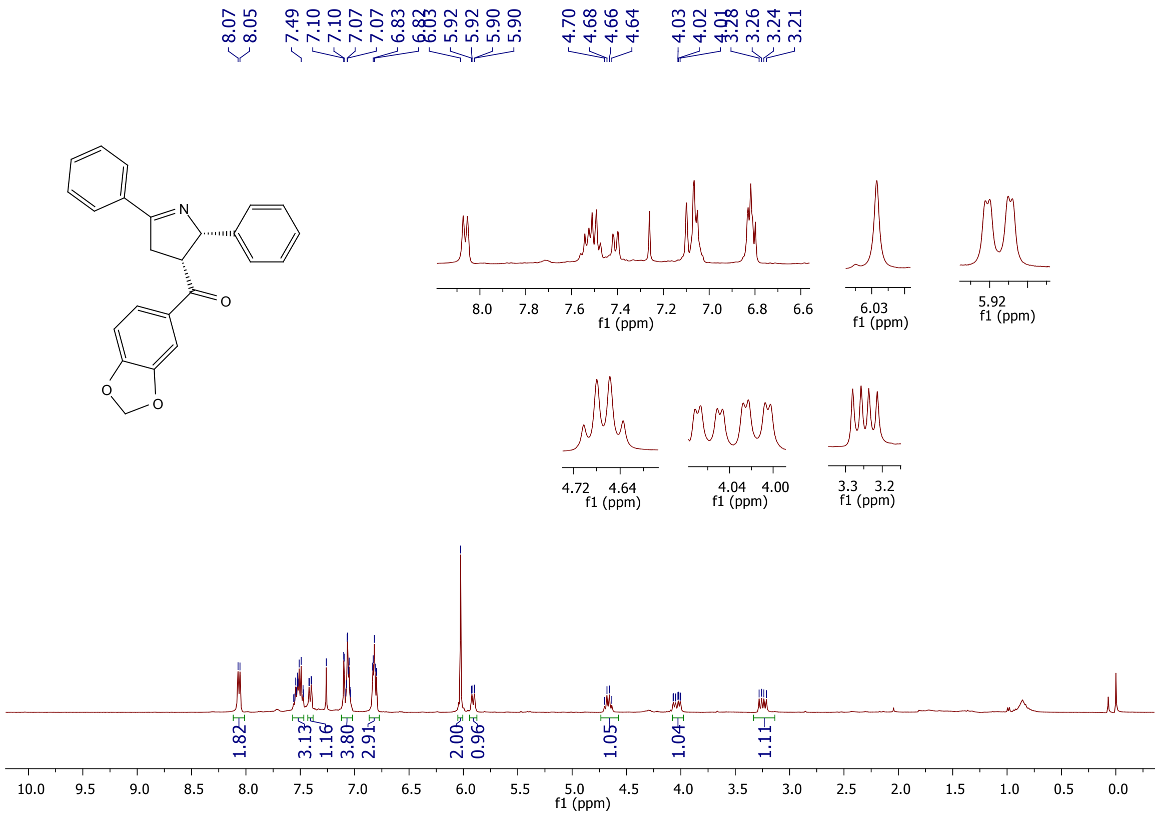

Figure S51. ^{1}H NMR (400 MHz, $CDCl_3$) of compound *cis*-**3i.**

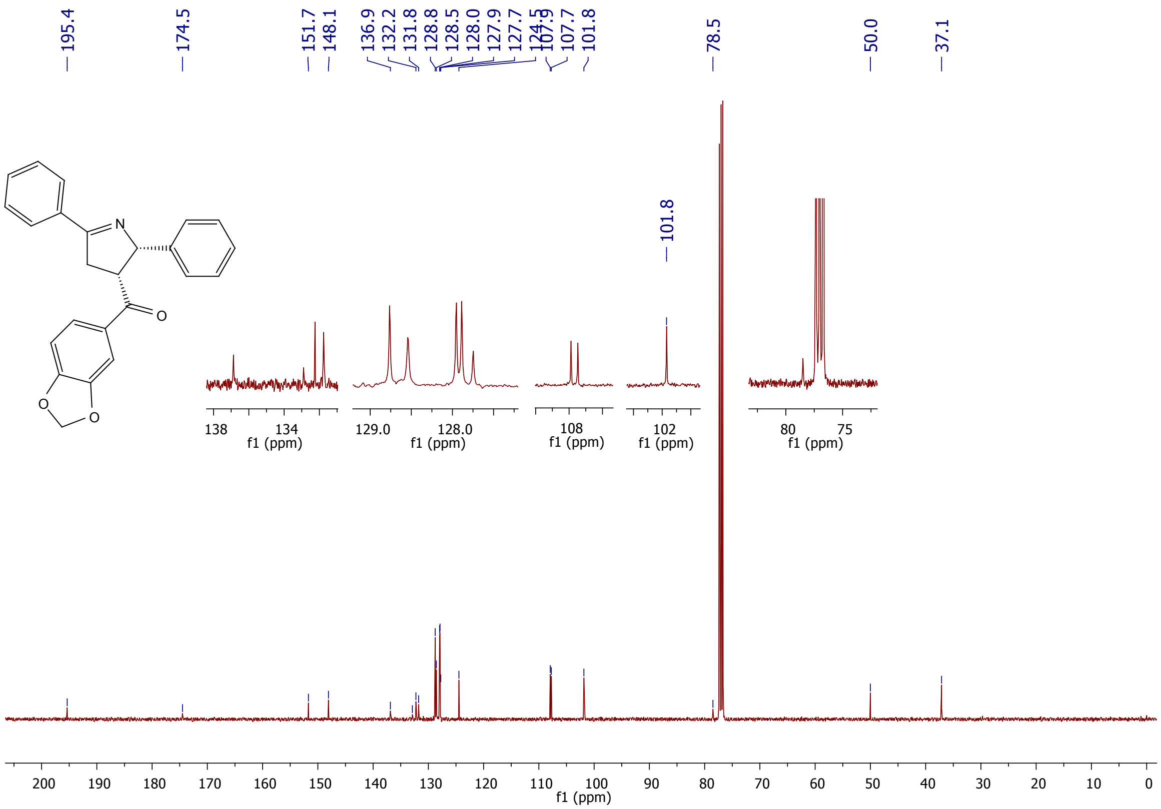

Figure S52. ^{13}C{^{1}H} NMR (100 MHz, $CDCl_3$) of compound *cis*-**3i.**

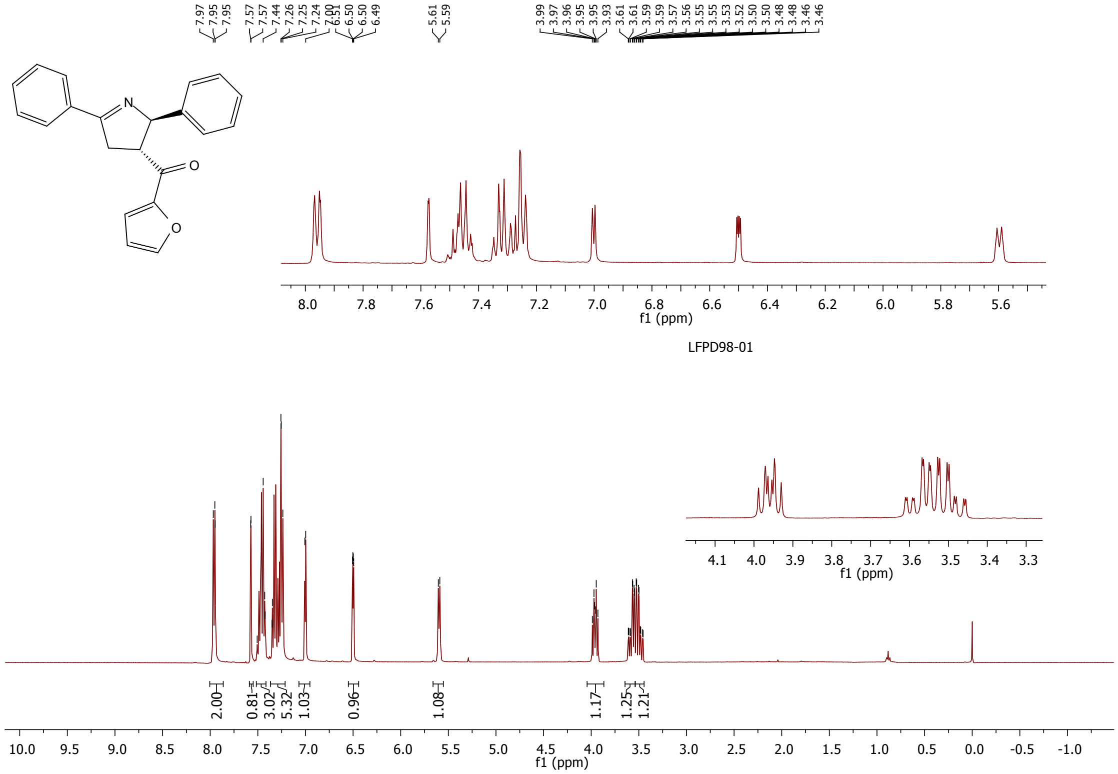

Figure S53. **^{1}H NMR** (400 MHz, $CDCl_3$) of compound *trans*-**3j.**

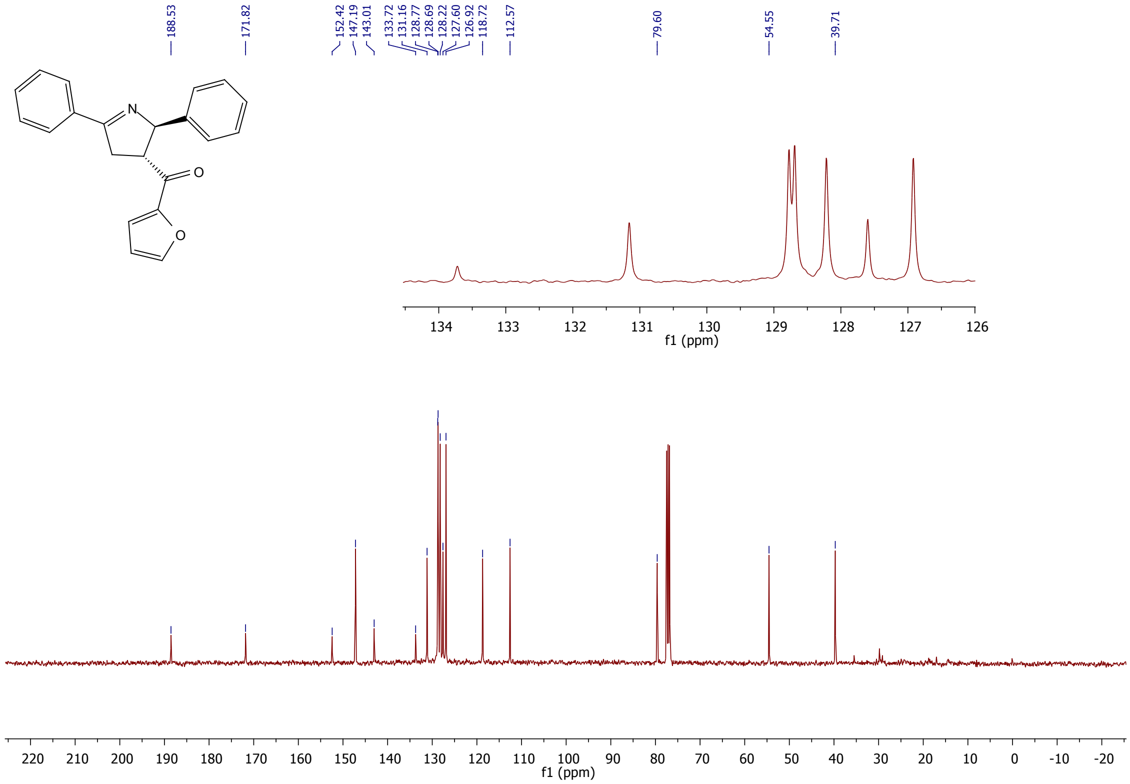

Figure S54. **$^{13}C\{^{1}H\}$ NMR** (100 MHz, $CDCl_3$) of compound *trans*-**3j.**

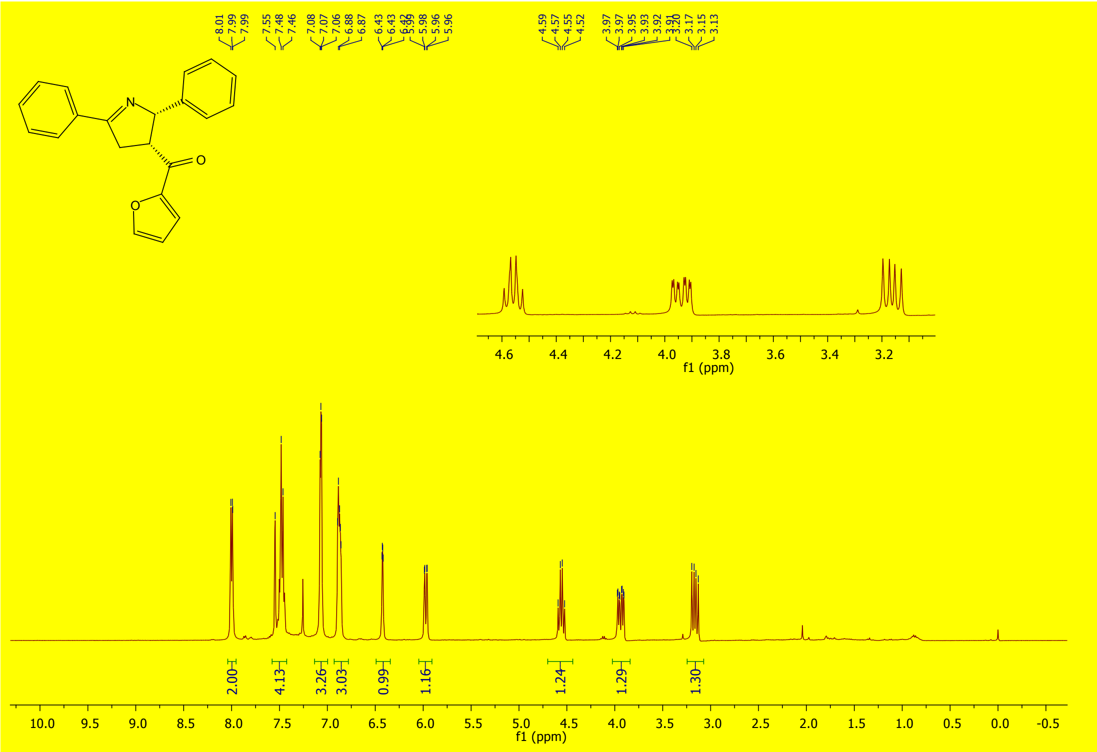

Figure S55. **^{1}H NMR** (400 MHz, $CDCl_3$) of compound *cis*-**3j.**

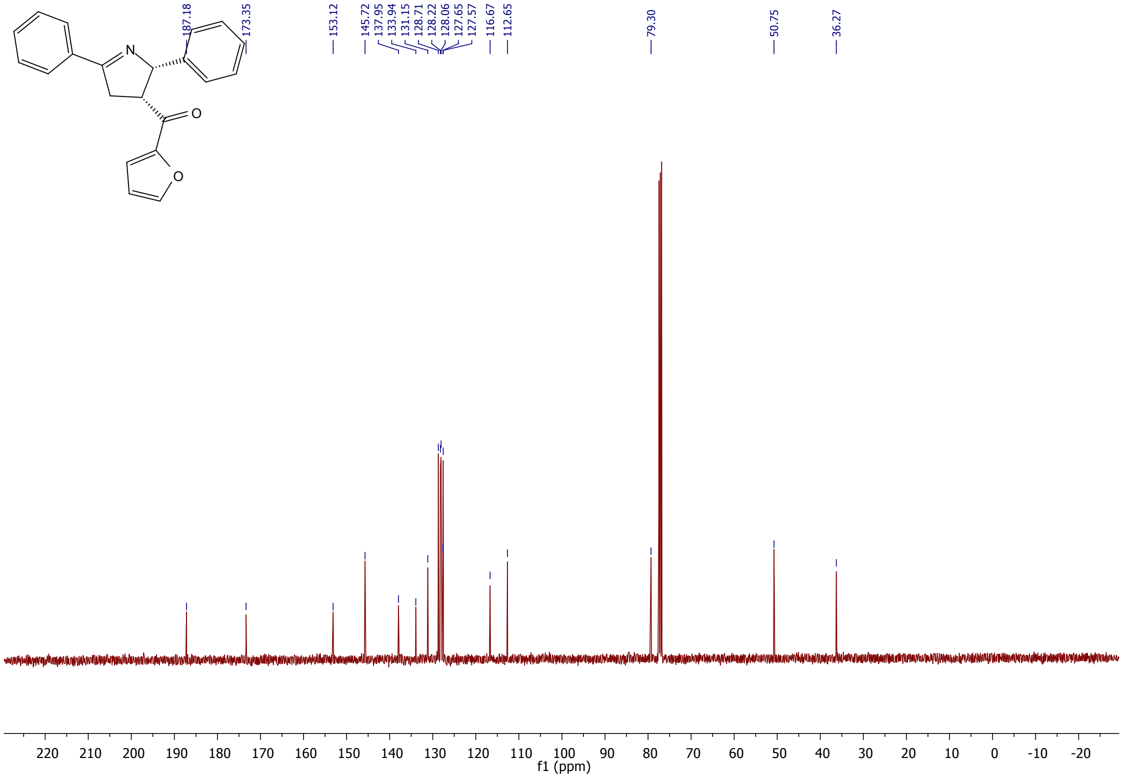

Figure S56. **^{13}C{^{1}H} NMR** (100 MHz, $CDCl_3$) of compound *cis*-**3j.**

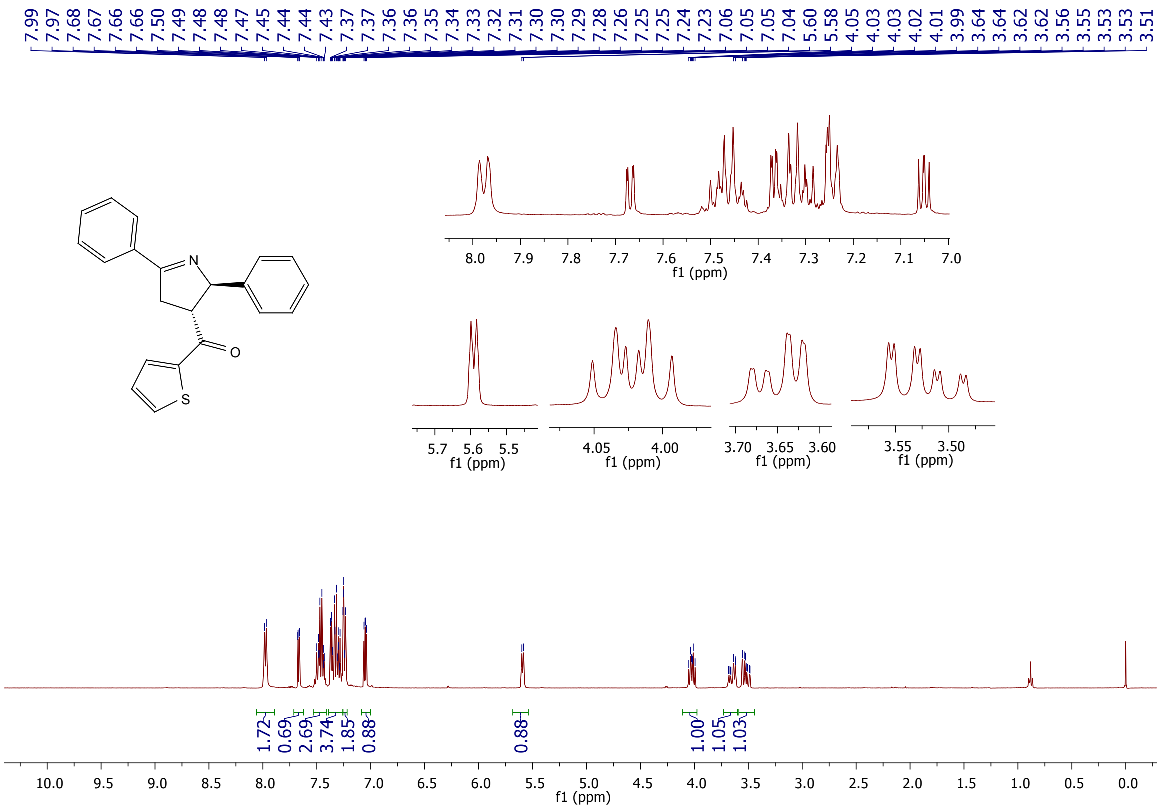

Figure S57. **^{1}H NMR** (400 MHz, $CDCl_3$) of compound *trans*-**3k.**

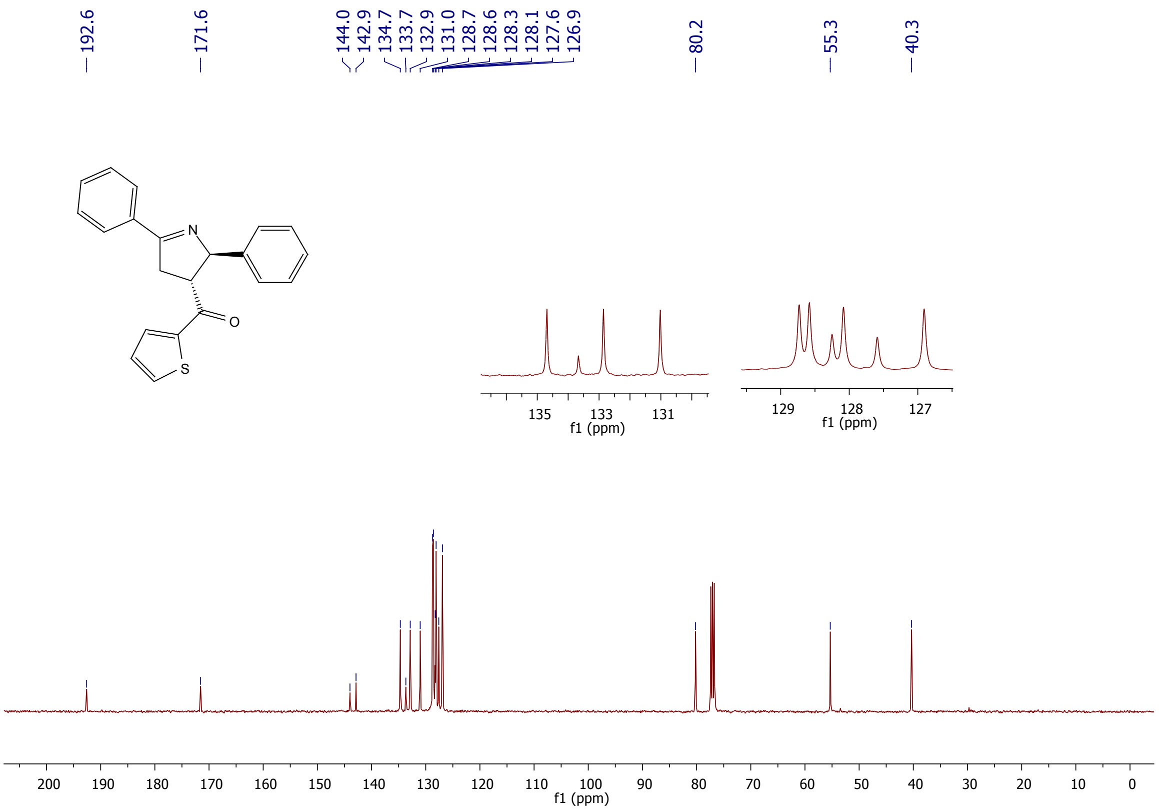

Figure S58. **^{13}C{^{1}H} NMR** (100 MHz, $CDCl_3$) of compound *trans*-**3k.**

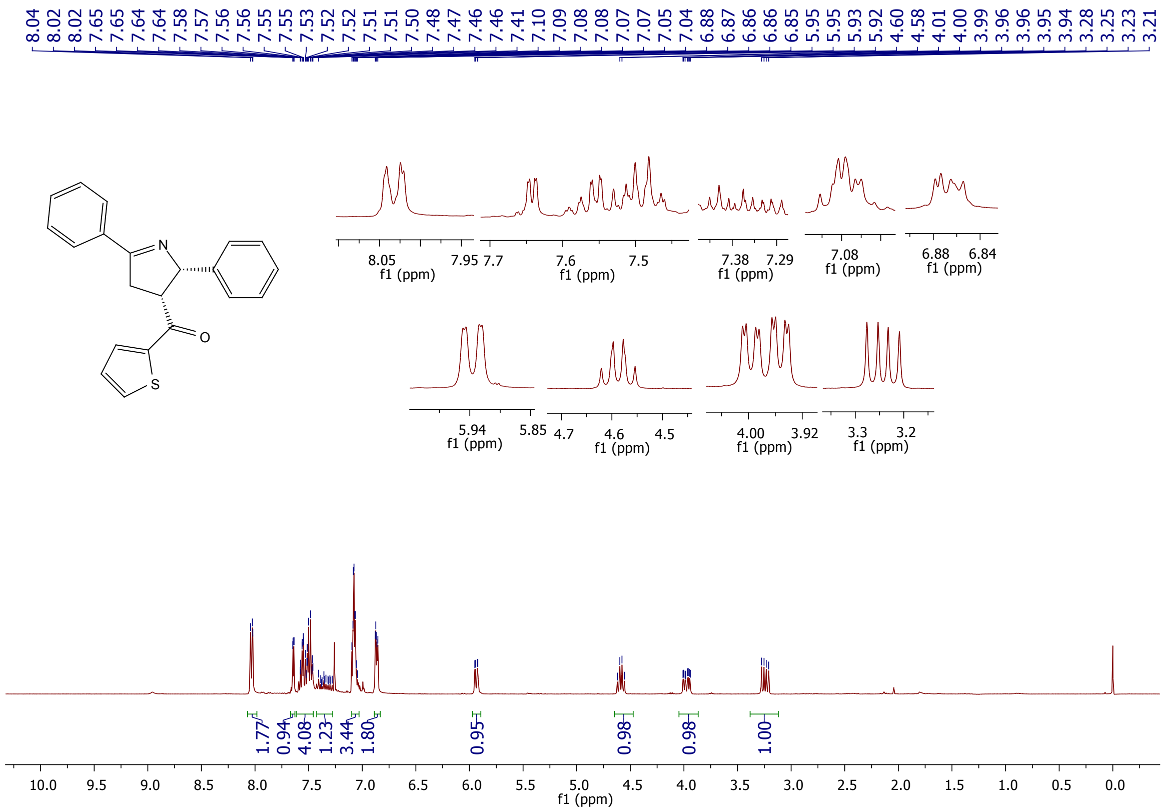

Figure S59. **^{1}H NMR** (400 MHz, $CDCl_3$) of compound *cis*-**3k.**

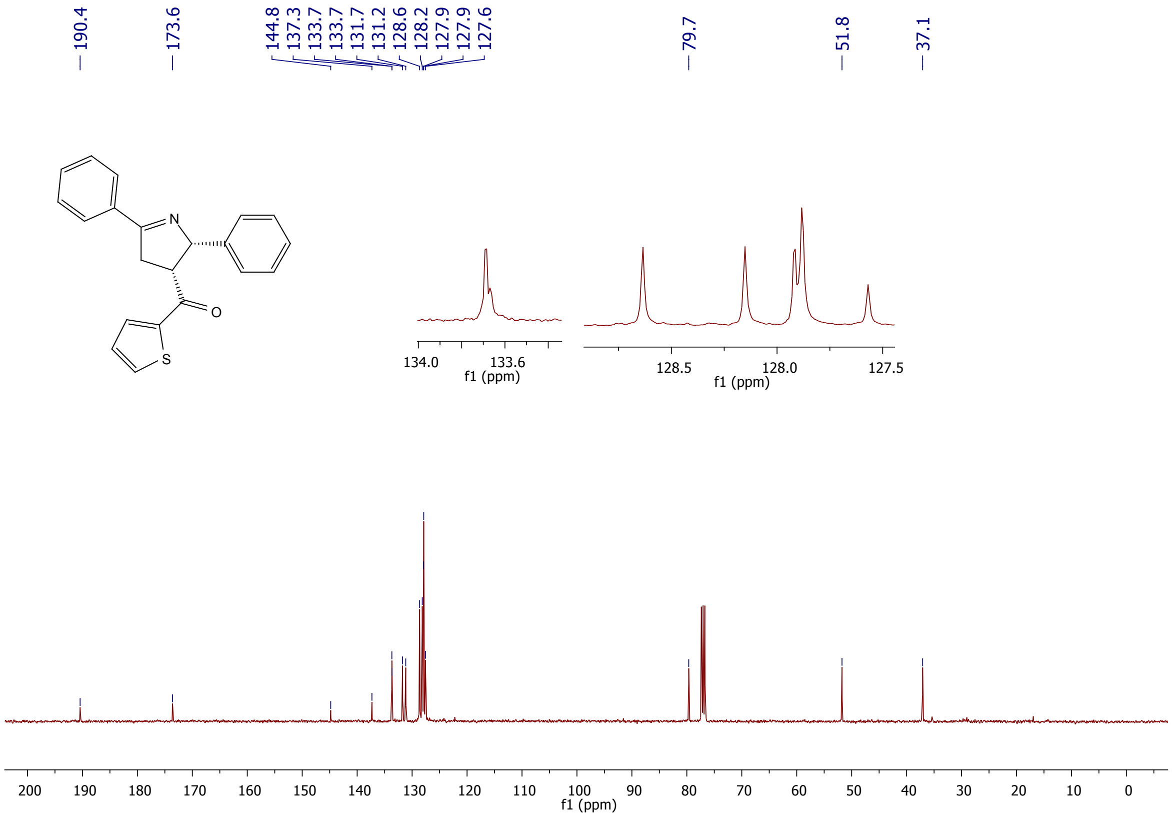

Figure S60. **$^{13}C\{^1H\}$ NMR** (100 MHz, $CDCl_3$) of compound *cis*-**3k.**

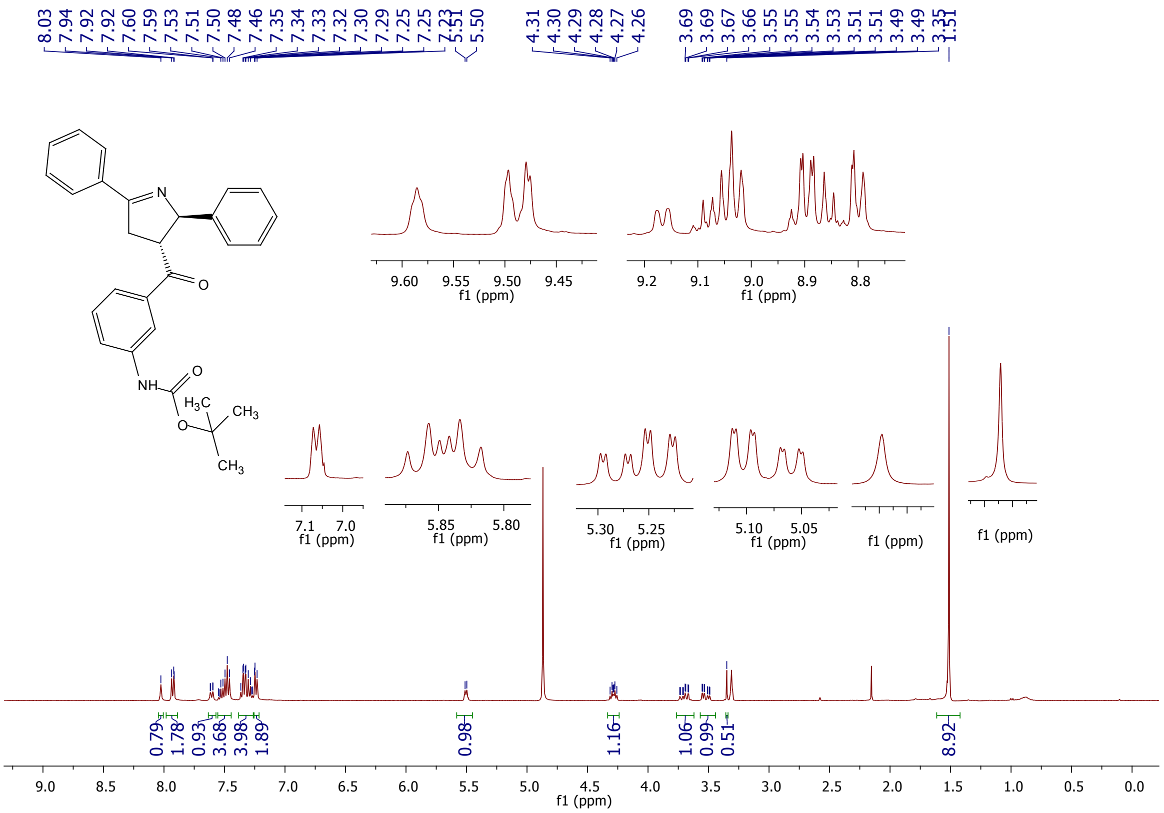

Figure S61. ^{1}H NMR (400 MHz, $CDCl_3$) of compound *trans*-**3l.**

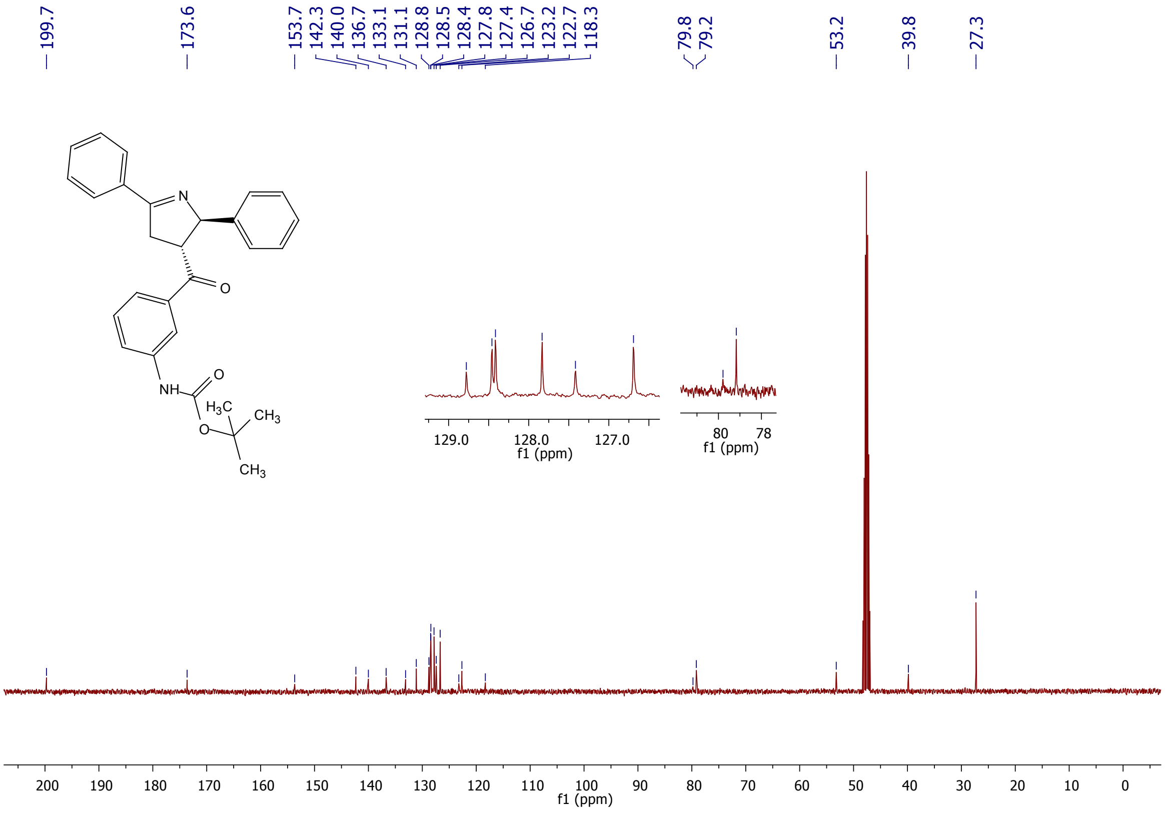

Figure S62. $^{13}C\{^{1}H\}$ NMR 100 MHz, $CDCl_3$) of compound *trans*-**3l.**

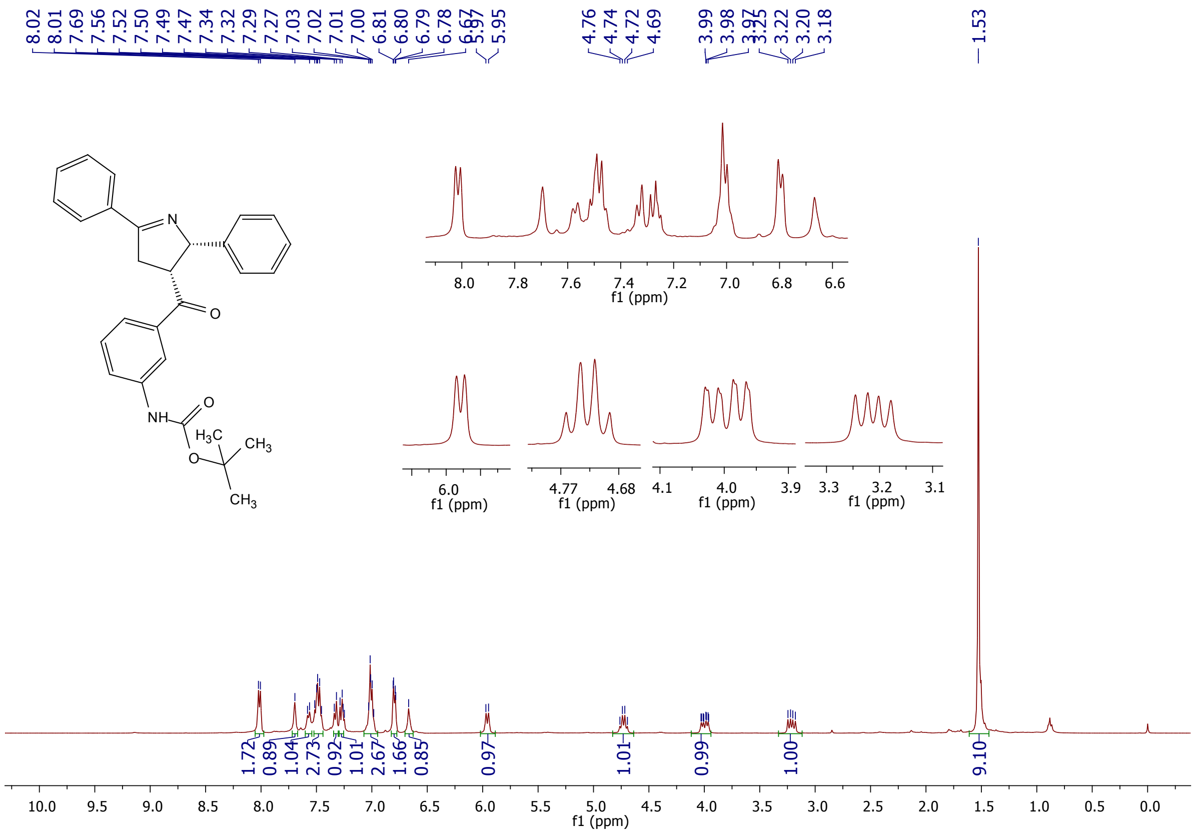

Figure S63. ^{1}H NMR (400 MHz, $CDCl_3$) of compound *cis*-**3l.**

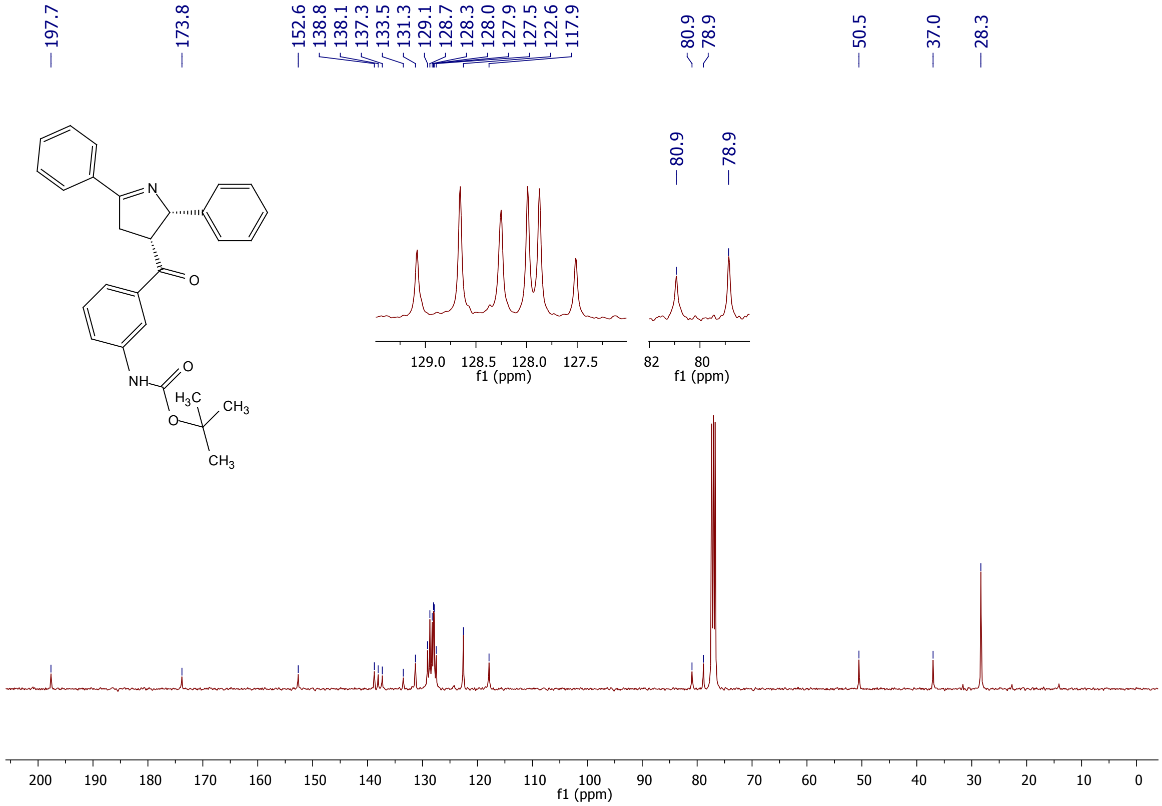

Figure S64. ^{13}C{^{1}H} NMR (100 MHz, $CDCl_3$) of compound *cis*-**3l.**

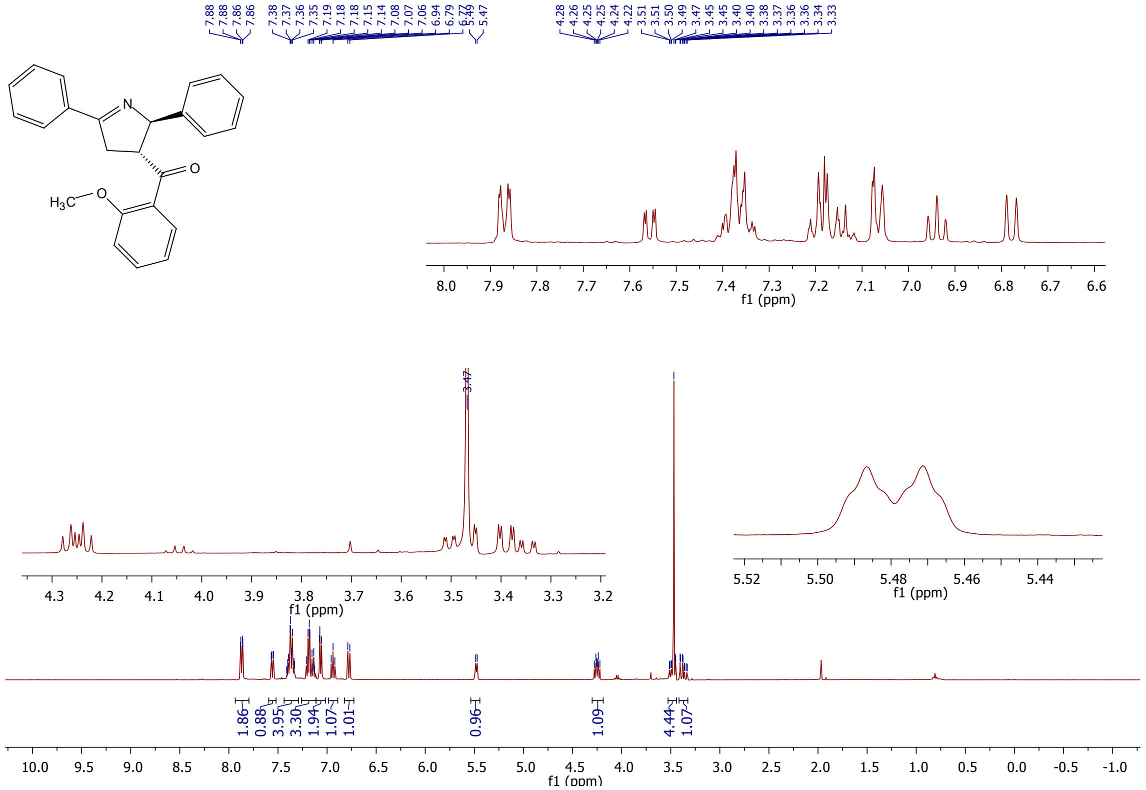

Figure S65. ^{1}H NMR (400 MHz, $CDCl_3$) of compound *trans*-**3m.**

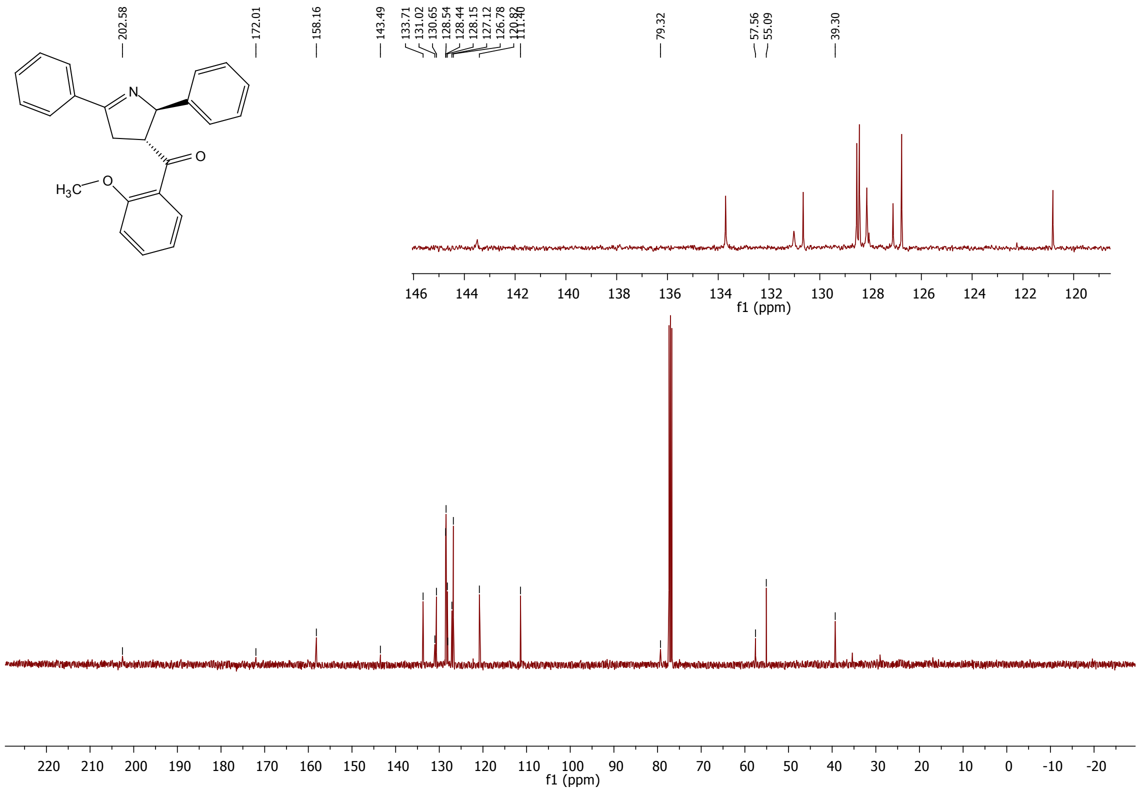

Figure S66. $^{13}C\{^{1}H\}$ NMR (100 MHz, $CDCl_3$) of compound *trans*-**3m.**

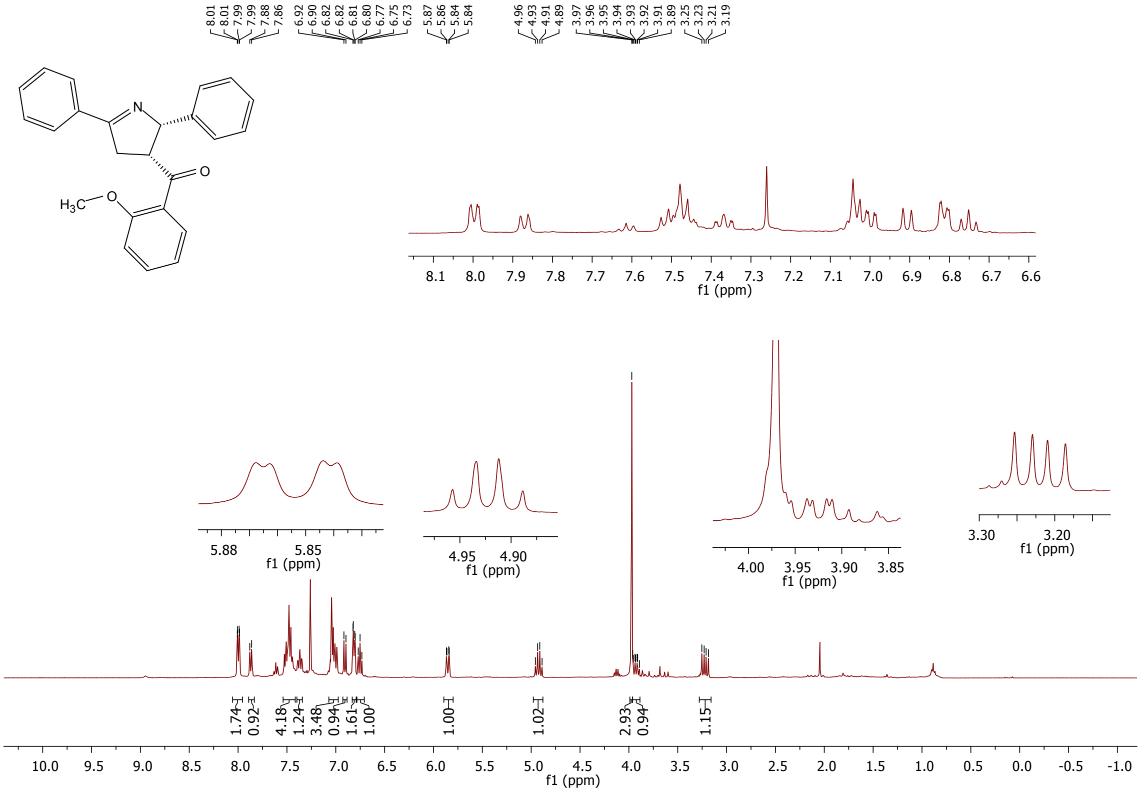

Figure S67. ^{1}H NMR (400 MHz, $CDCl_3$) of compound *cis*-**3m.**

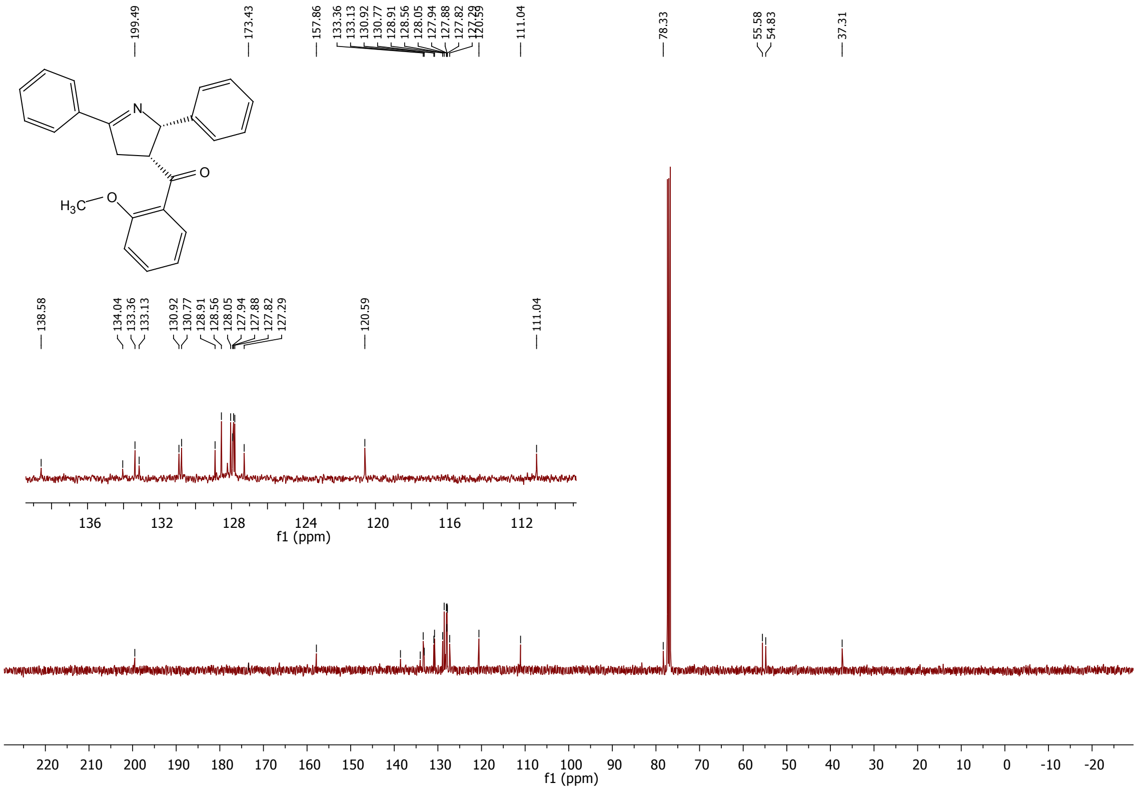

Figure S68. $^{13}C\{^{1}H\}$ NMR (100 MHz, $CDCl_3$) of compound *cis*-**3m.**

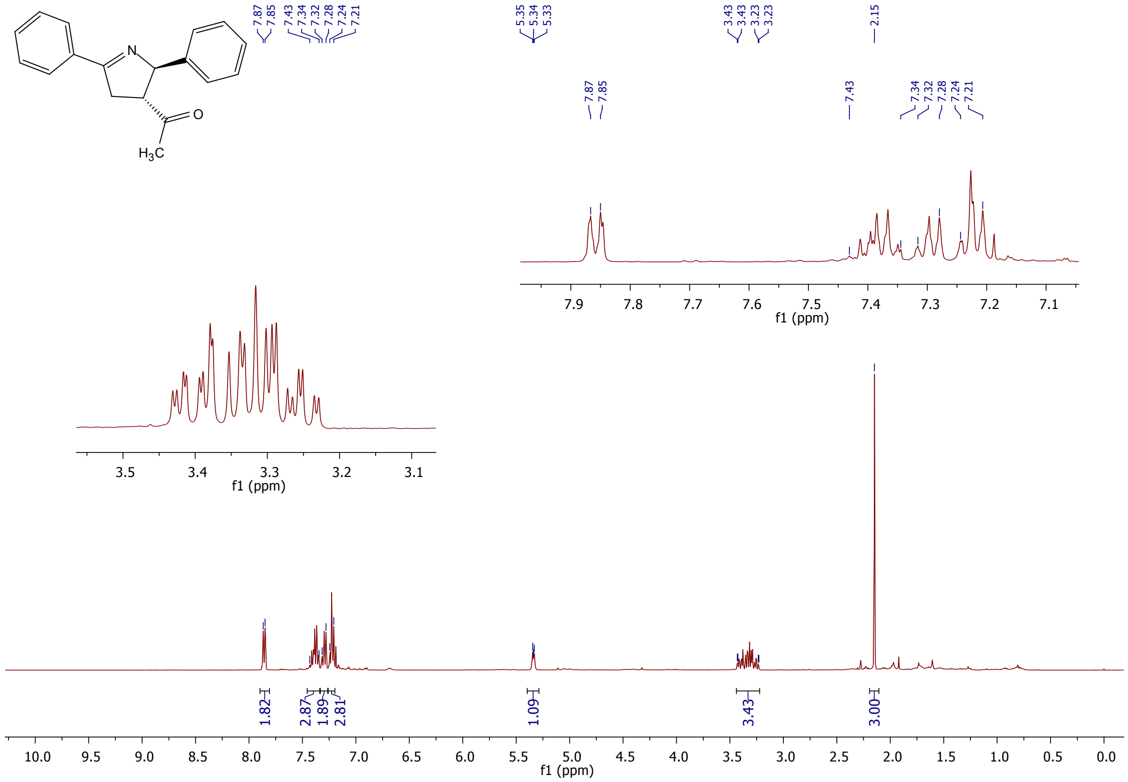

Figure S69. ¹H NMR (400 MHz, $CDCl_3$) of compound *trans*-**3n.**

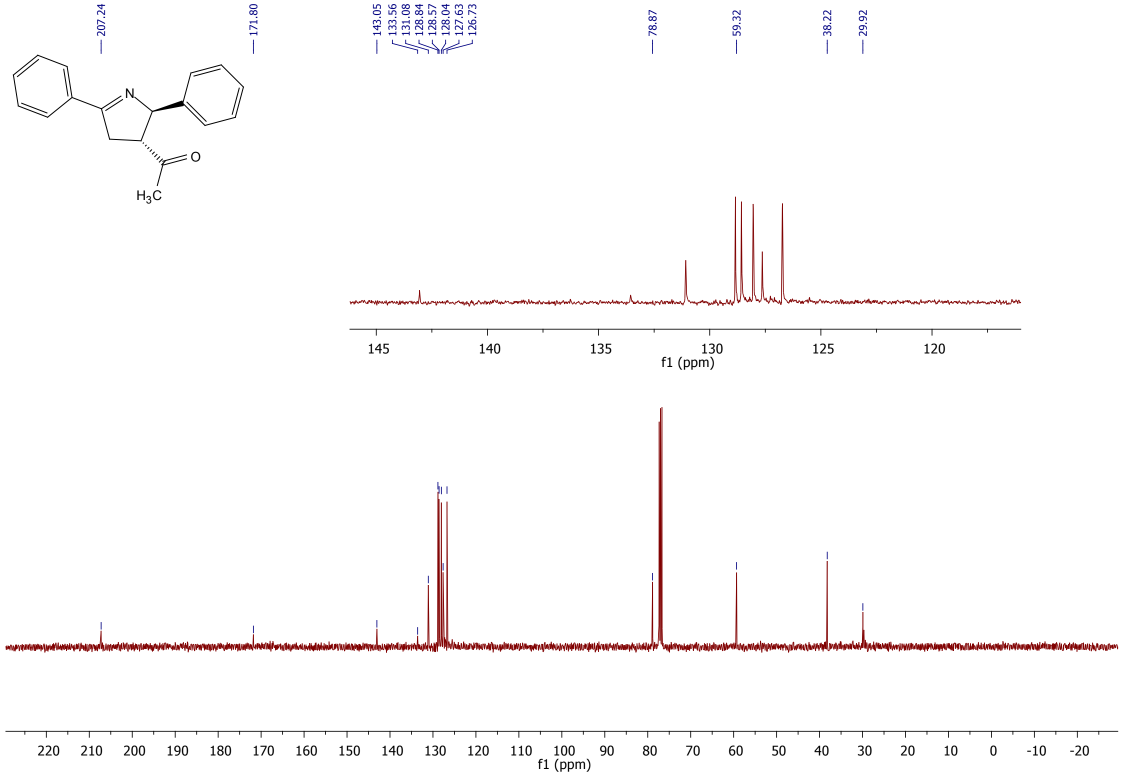

Figure S70. ¹³C{¹H} NMR (100 MHz, $CDCl_3$) of compound *trans*-**3n.**

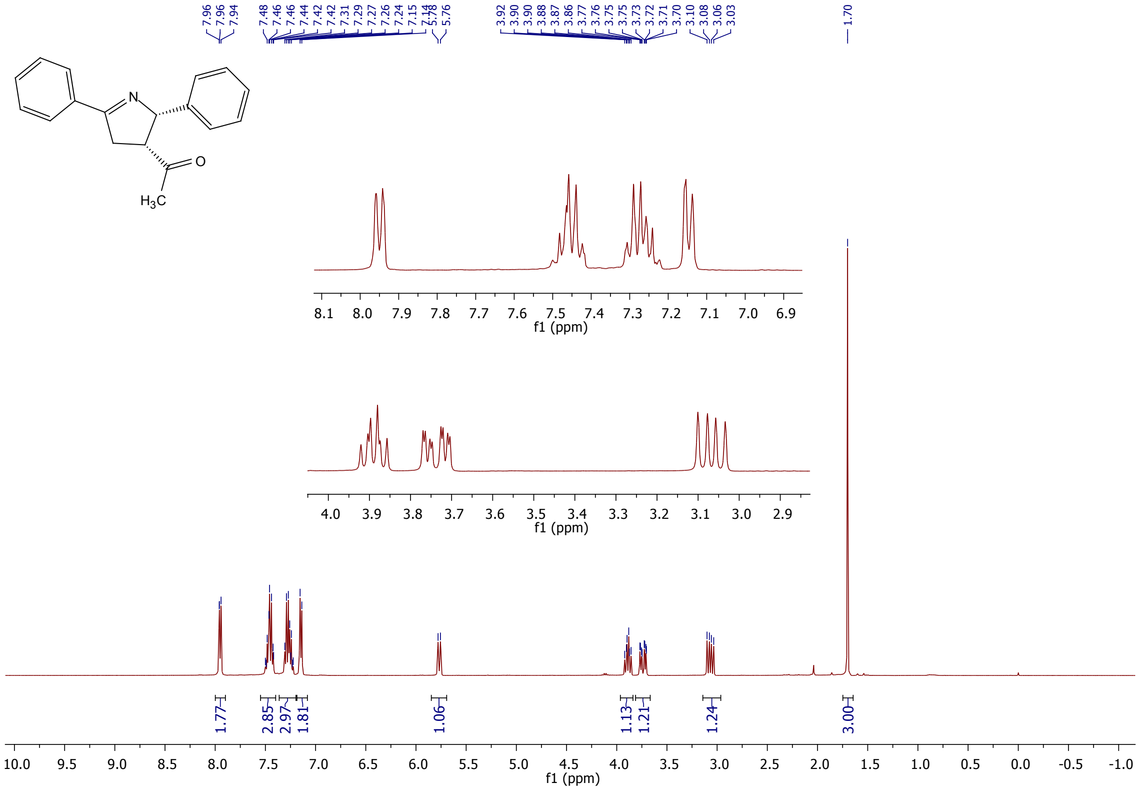

Figure S71. ^{1}**H NMR** (400 MHz, $CDCl_3$) of compound *cis*-**3n.**

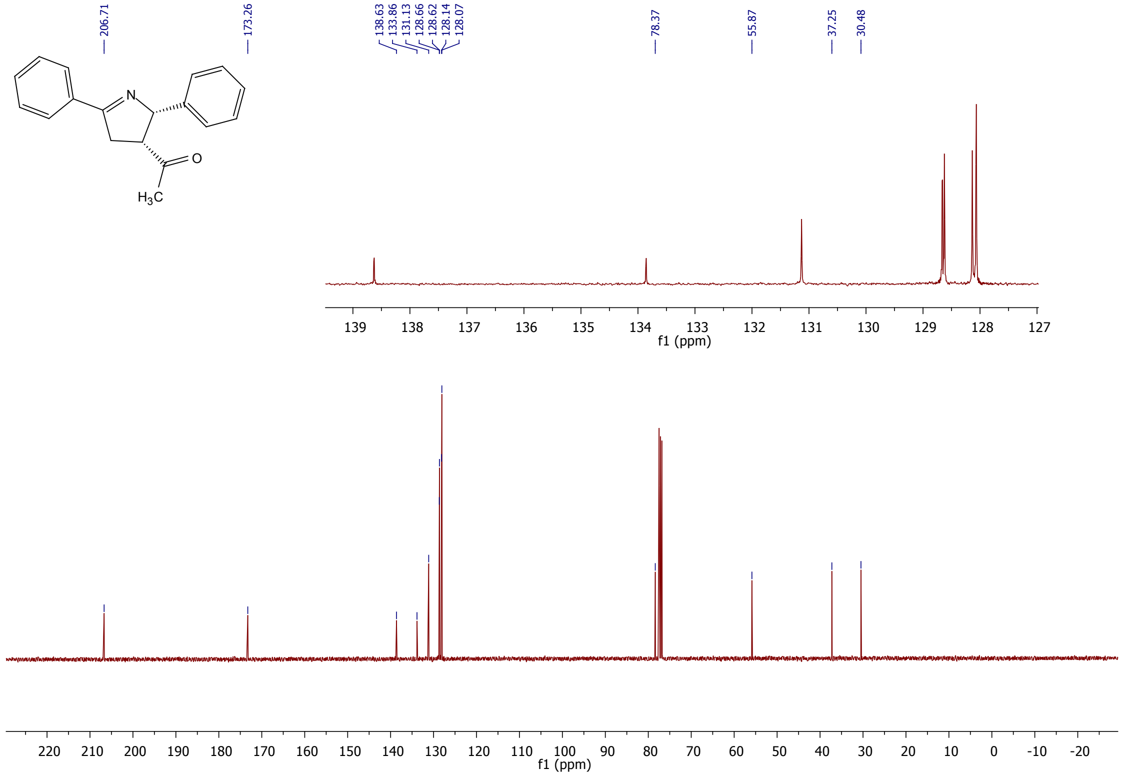

Figure S72. ^{13}**C{**^{1}**H} NMR** (100 MHz, $CDCl_3$) of compound *cis*-**3n.**

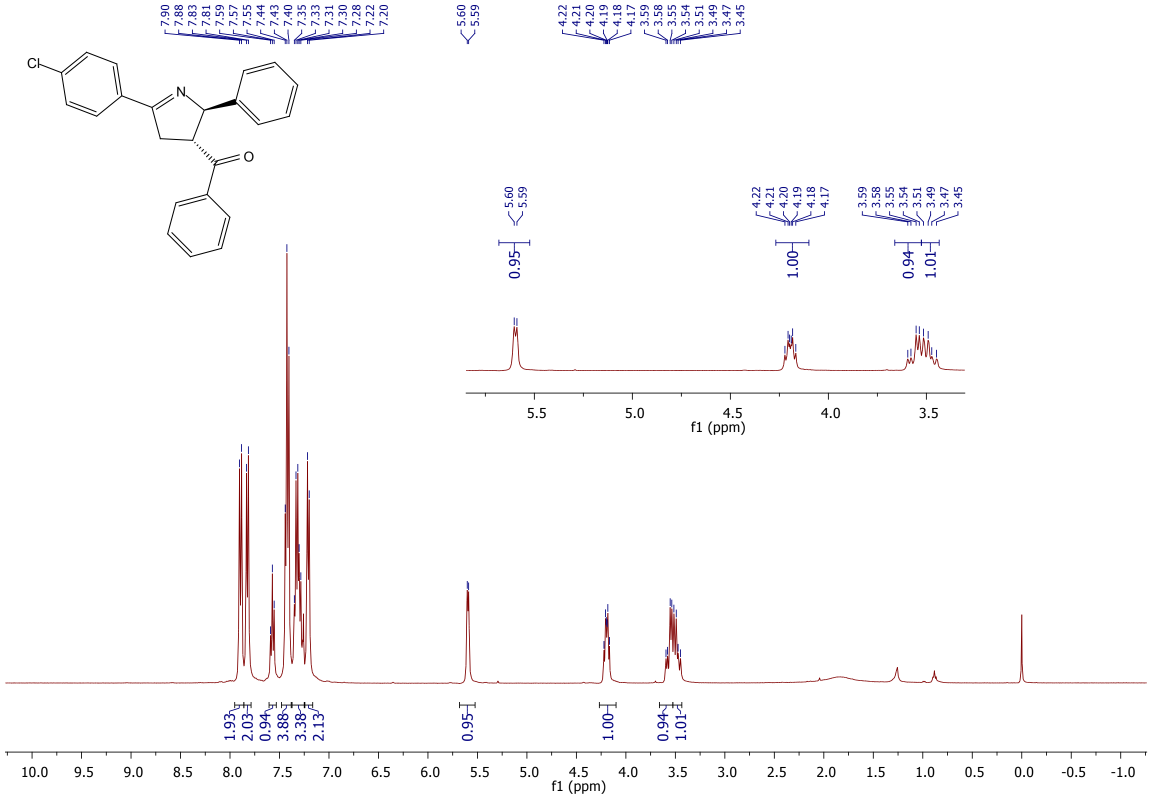

Figure S73. ¹H NMR (400 MHz, $CDCl_3$) of compound *trans*-**3o.**

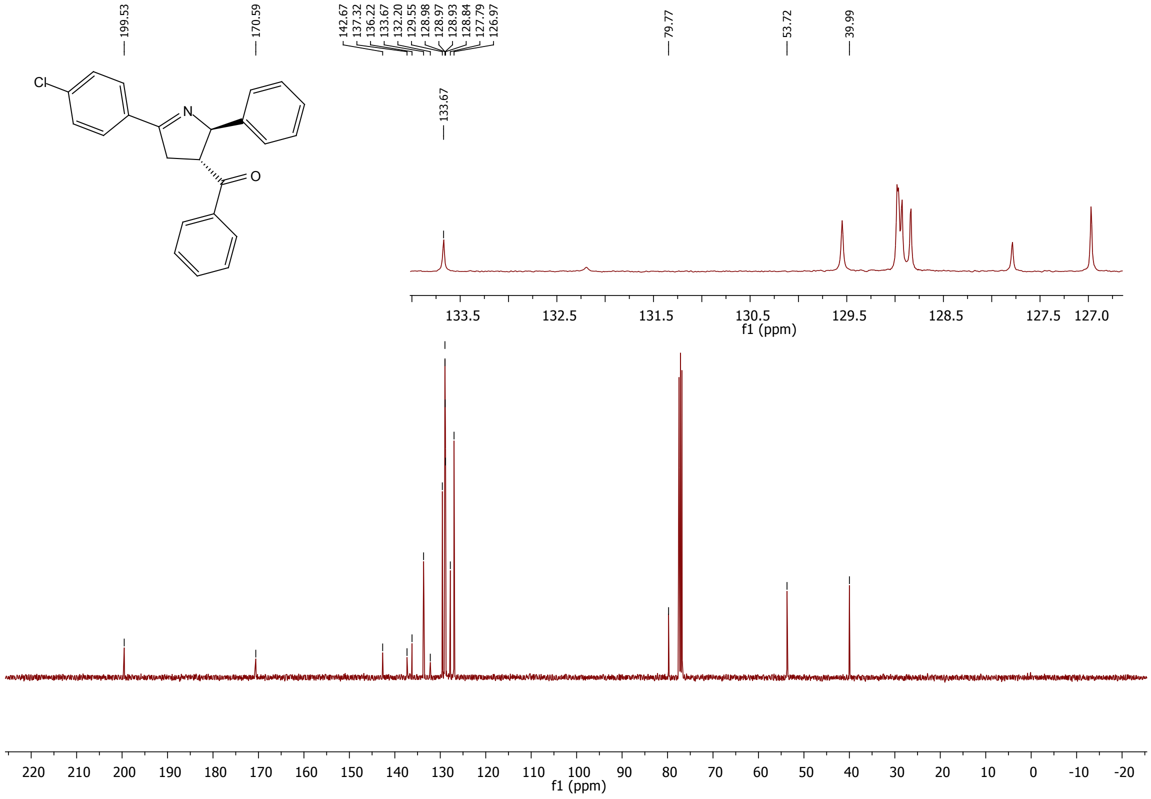

Figure S74. $^{13}C\{^{1}H\}$ NMR (100 MHz, $CDCl_3$) of compound *trans*-**3o.**

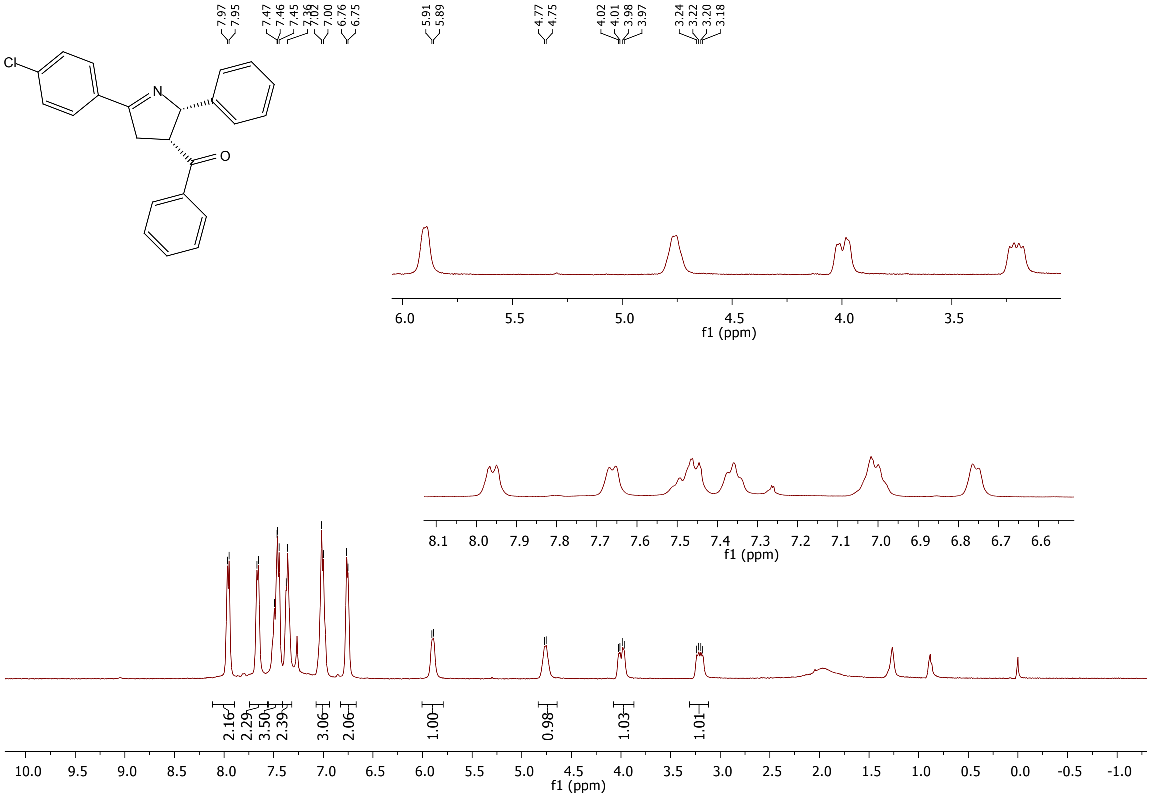

Figure S75. **^{1}H NMR** (400 MHz, $CDCl_3$) of compound *cis*-**3o.**

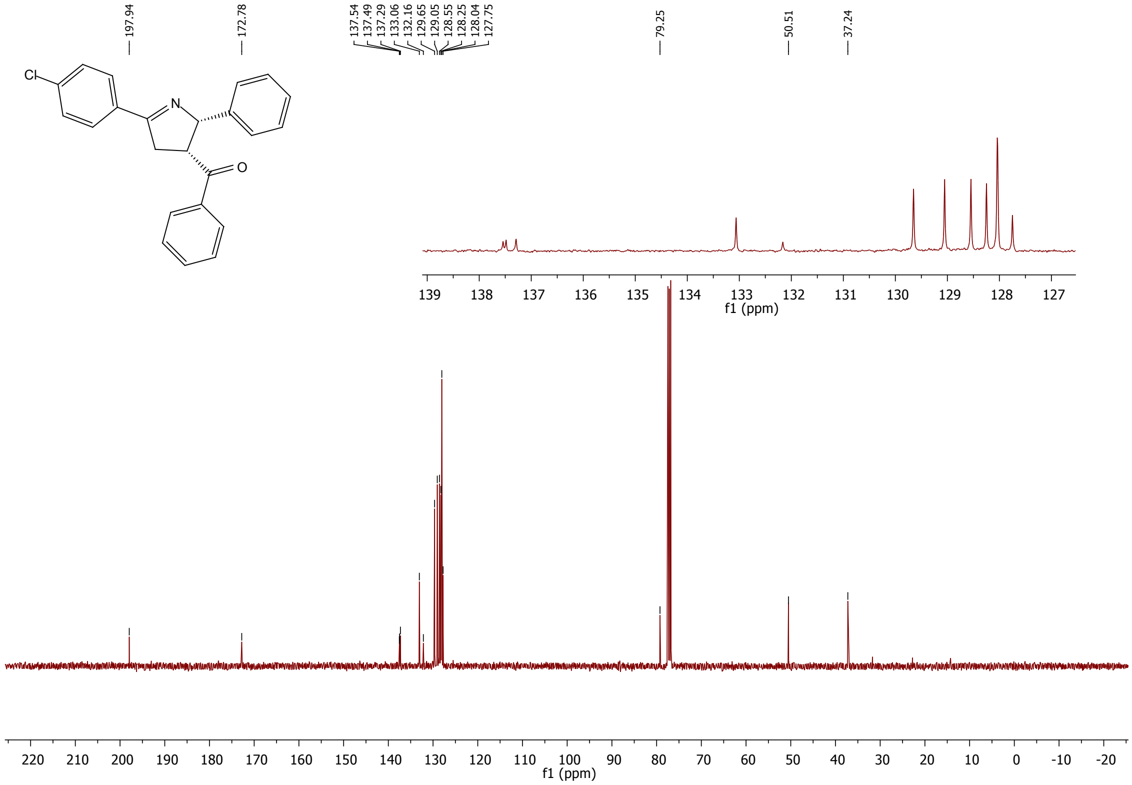

Figure S76. **$^{13}C\{^{1}H\}$ NMR** (100 MHz, $CDCl_3$) of compound *cis*-**3o.**

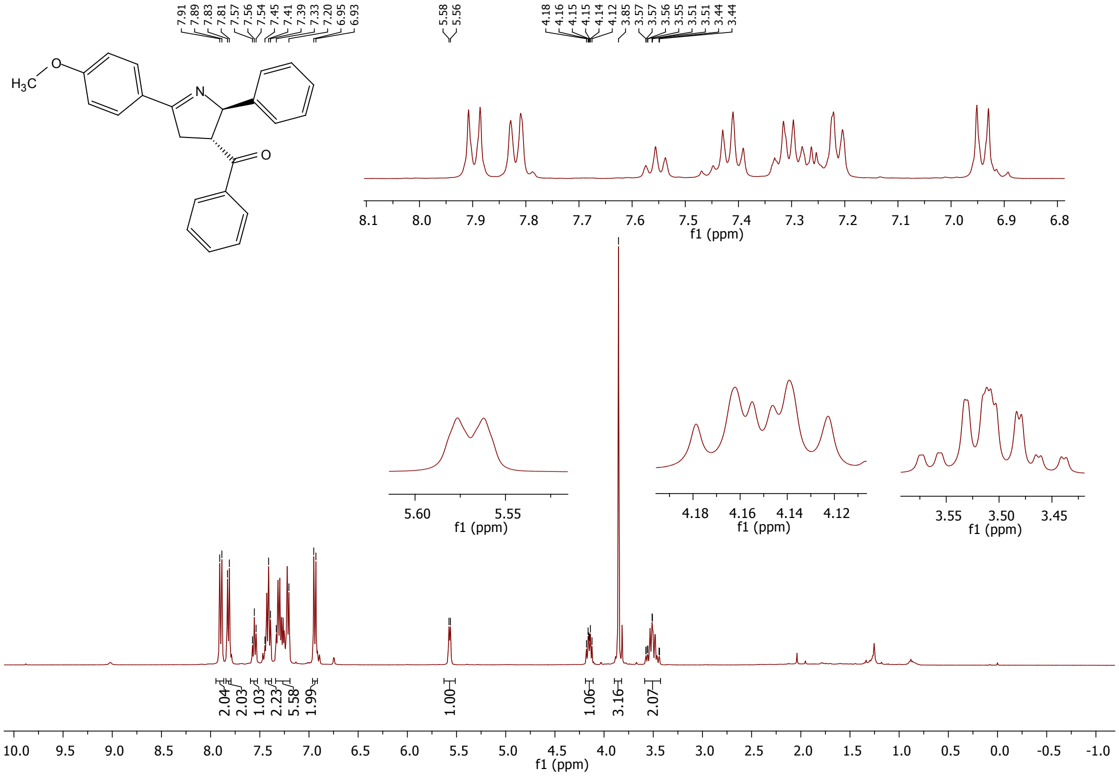

Figure S77. ^{1}**H NMR** (400 MHz, $CDCl_3$) of compound *trans*-**3p.**

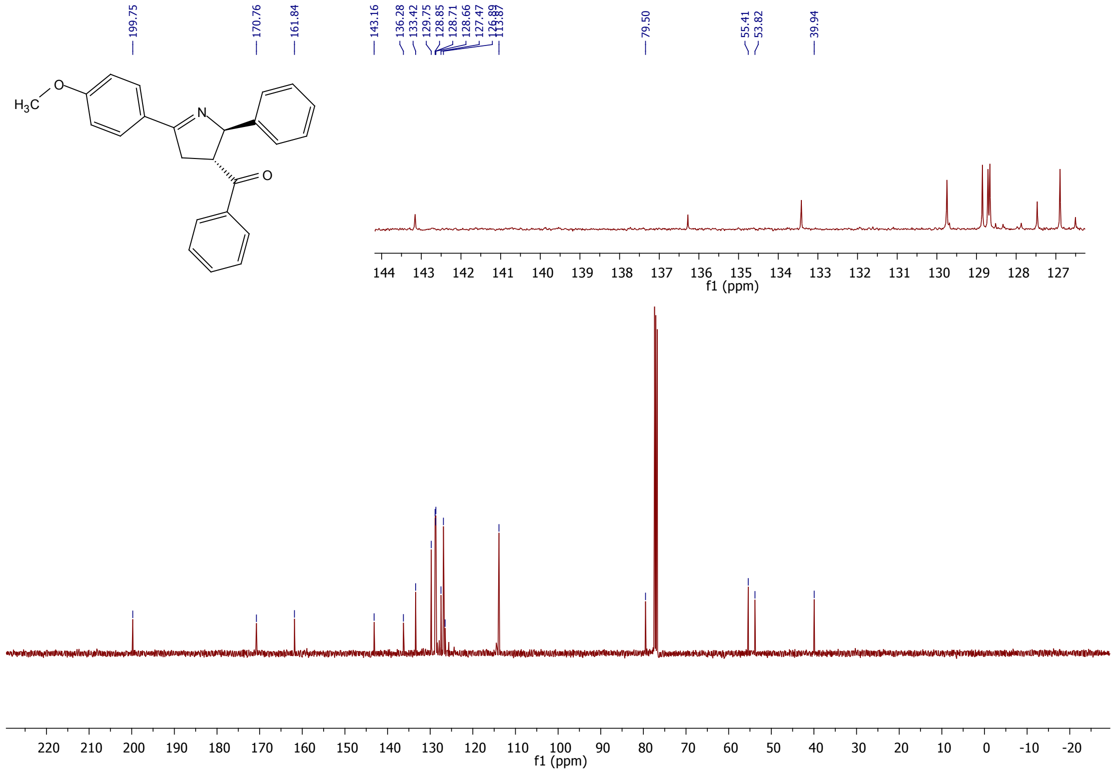

Figure S78. ^{13}**C{**^{1}**H} NMR** (100 MHz, $CDCl_3$) of compound *trans*-**3p.**

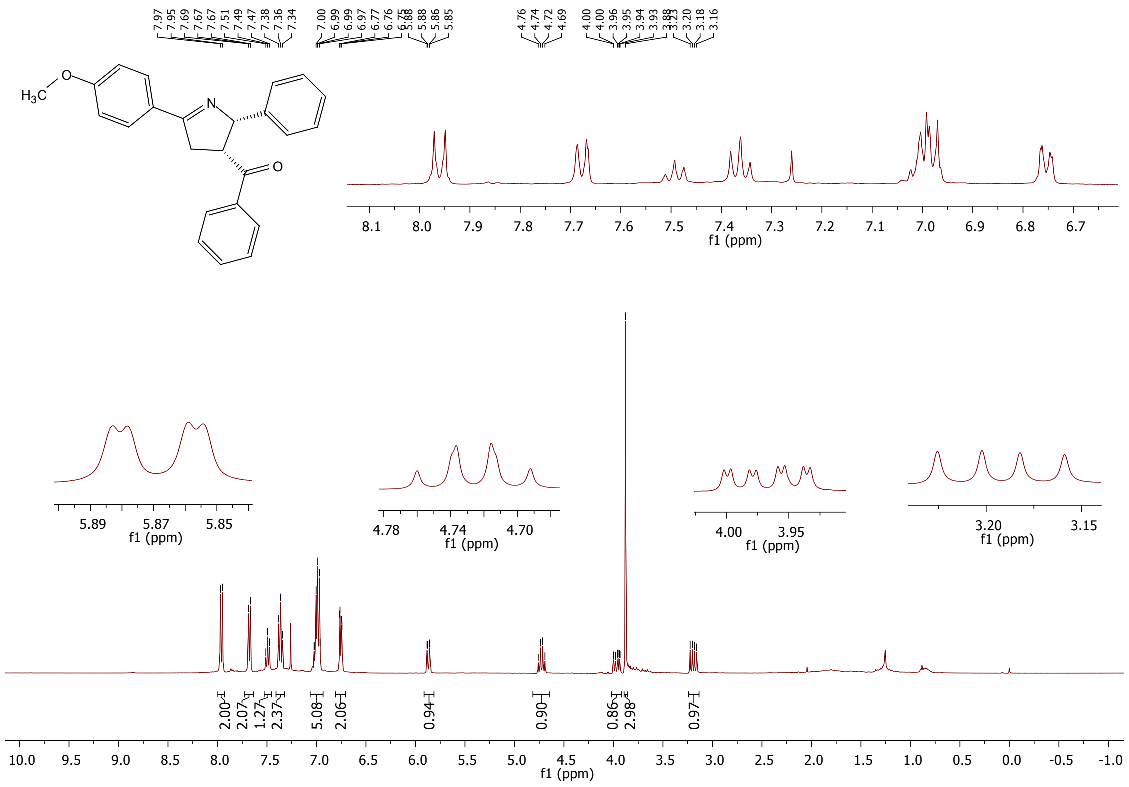

Figure S79. $^{13}C\{^1H\}$ NMR (100 MHz, $CDCl_3$) of compound *cis*-**3p.**

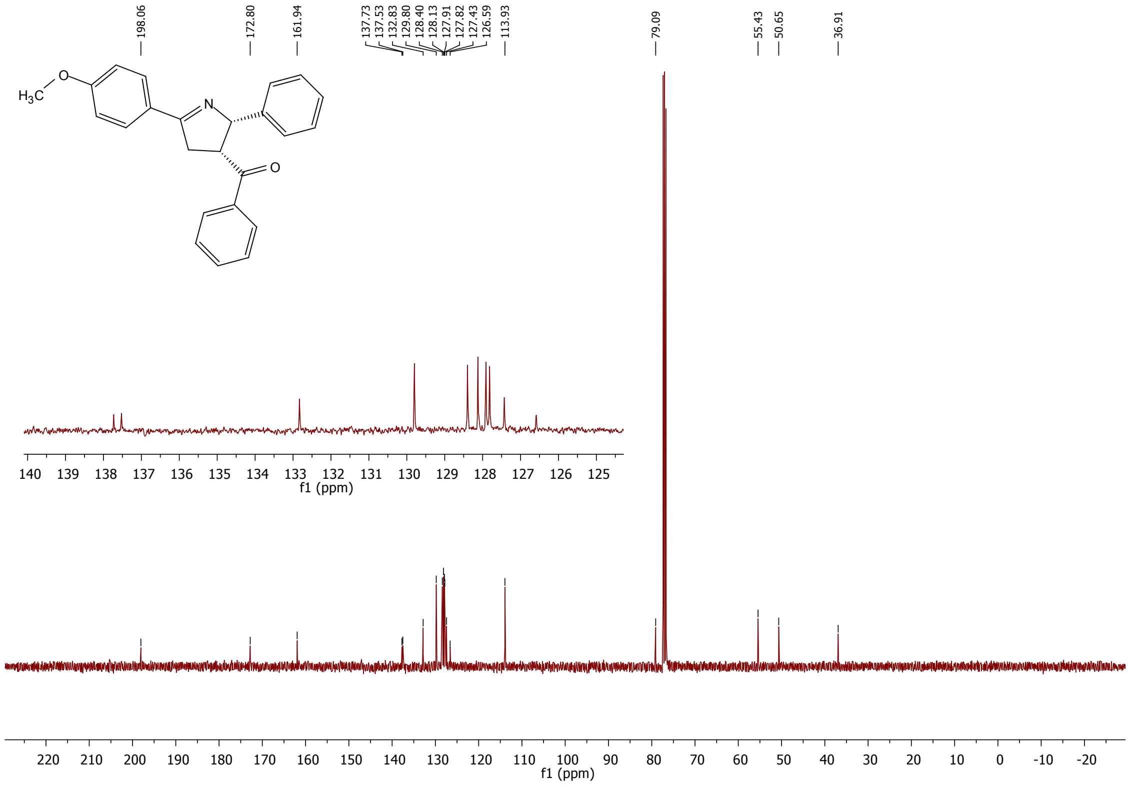

Figure S80. $^{13}C\{^1H\}$ NMR (100 MHz, $CDCl_3$) of compound *cis*-**3p** .

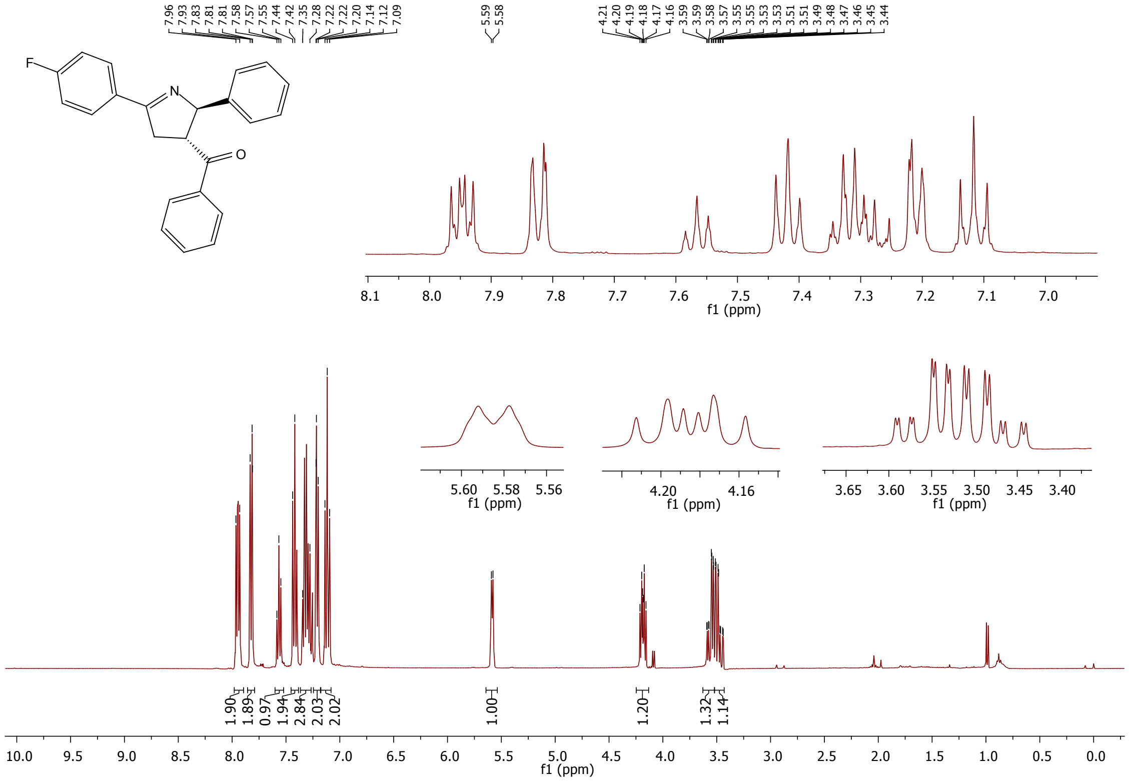

Figure S81. ^{1}**H NMR** (400 MHz, $CDCl_3$) of compound *trans*-**3q.**

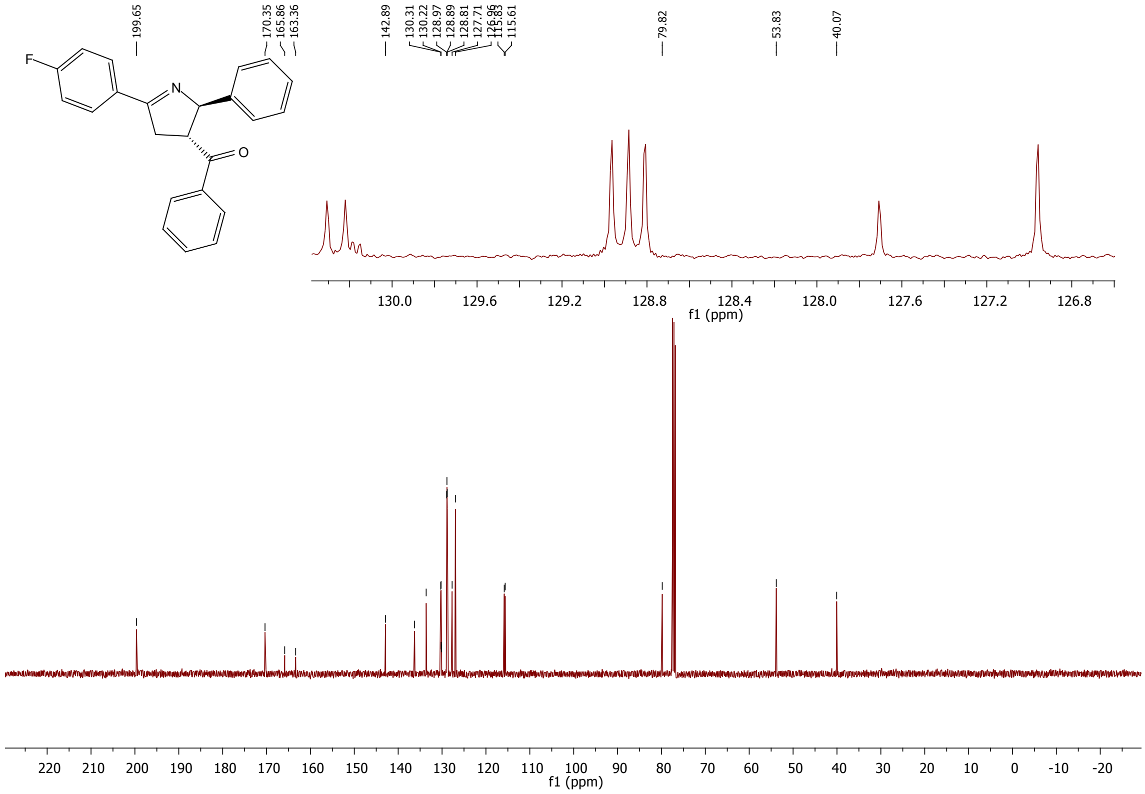

Figure S82. ^{13}**C{^{1}H} NMR** (100 MHz, $CDCl_3$) of compound *trans*-**3q.**

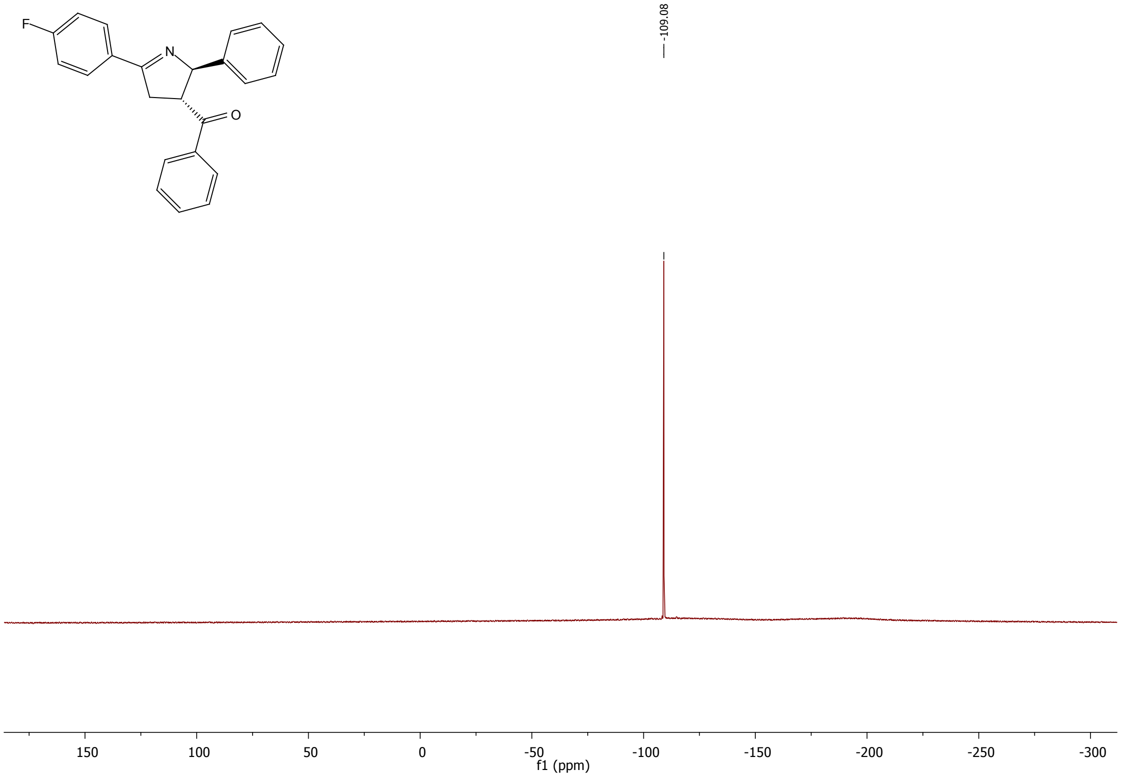

Figure S83. ^{19}**F NMR** (376 MHz, $CDCl_3$) of compound *trans*-**3q.**

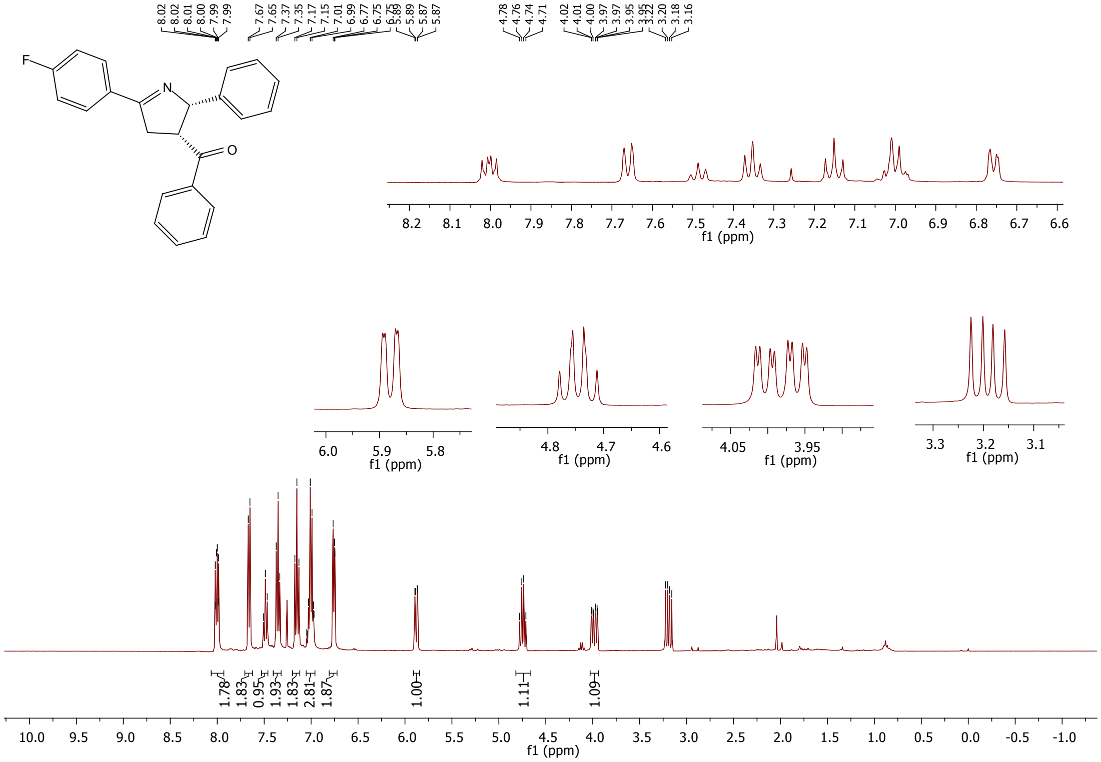

Figure S84. ^{1}**H NMR** (400 MHz, $CDCl_3$) of compound *cis*-**3q**

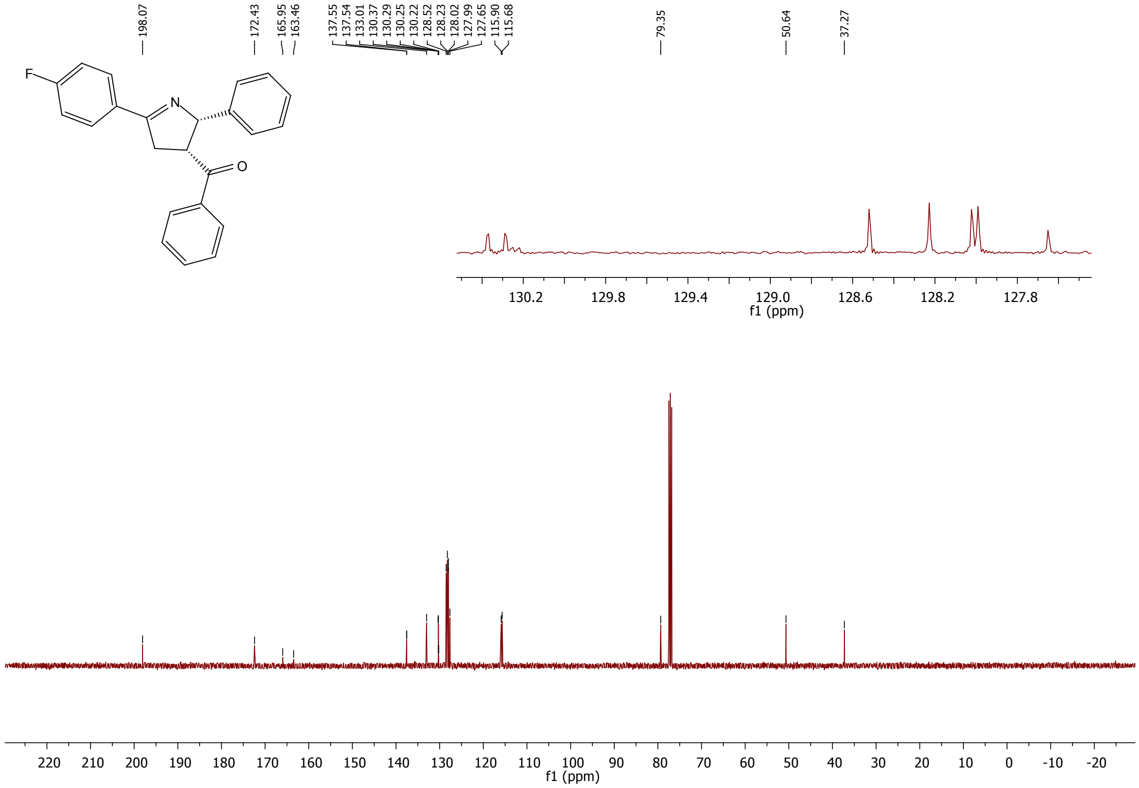

Figure S85. ^{13}**C NMR** (100 MHz, $CDCl_3$) of compound *cis*-**3q.**

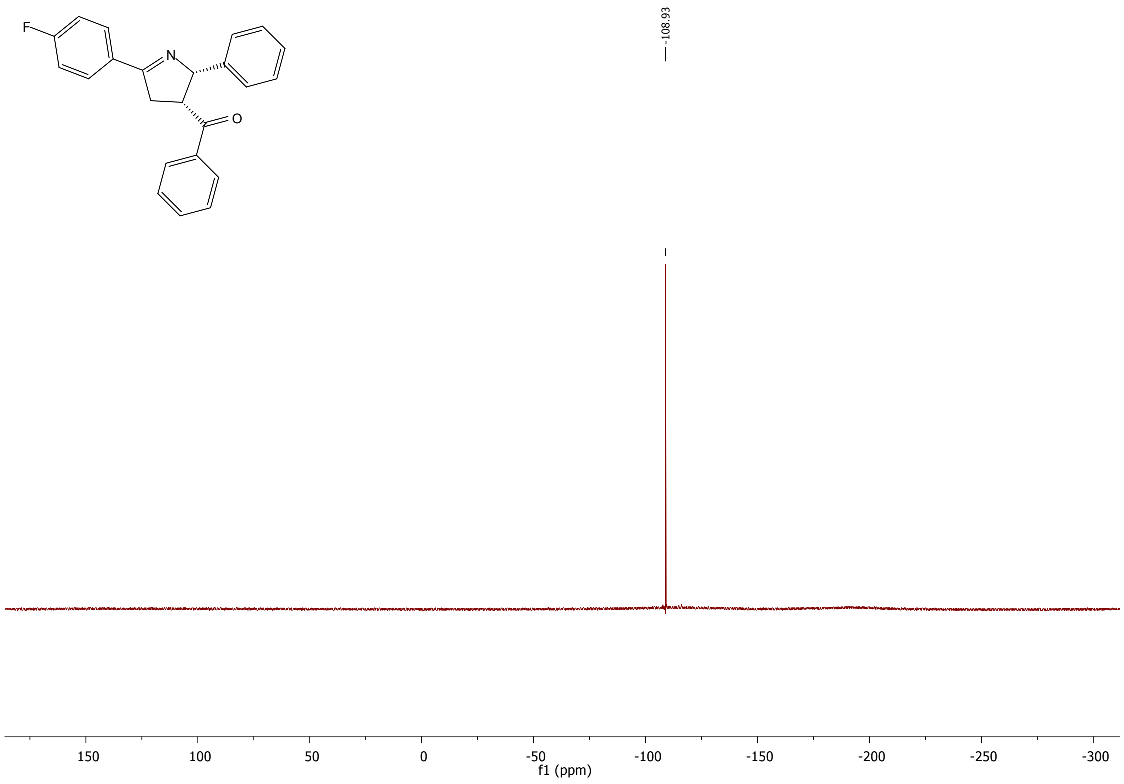

Figure S86. ^{19}**F NMR** (376 MHz, $CDCl_3$) of compound *cis*-**3q.**

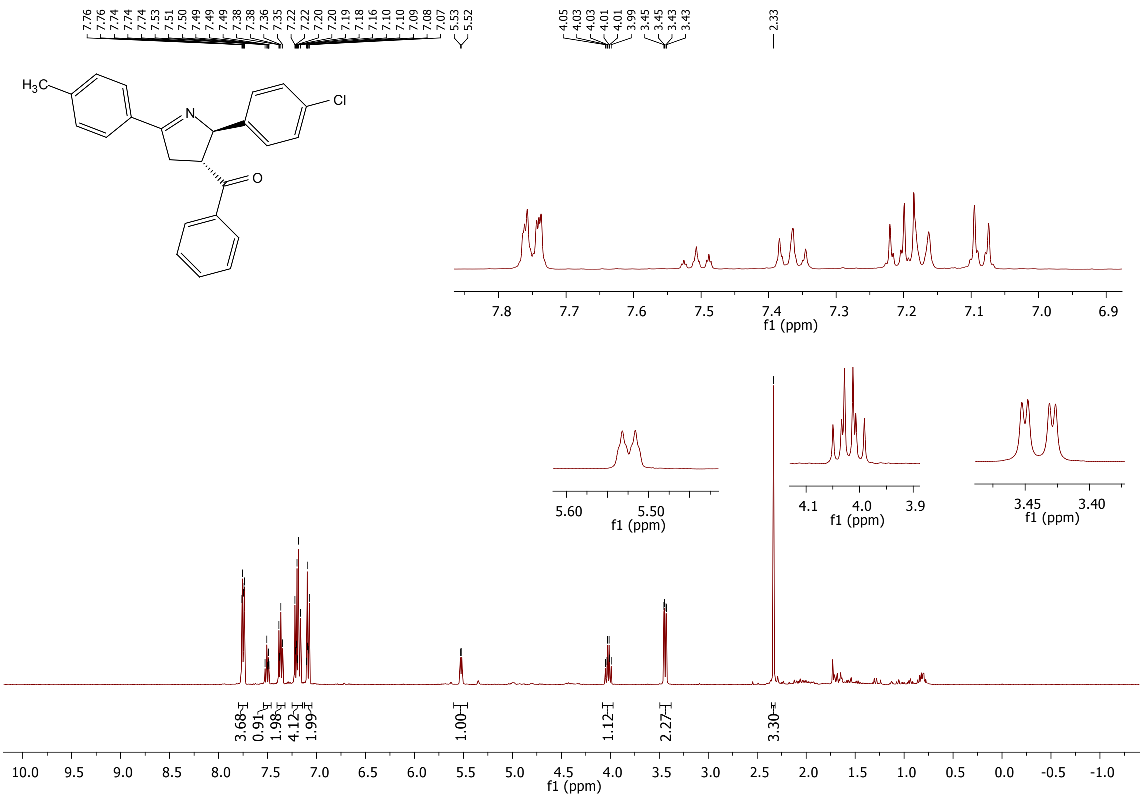

Figure S87. ^{1}**H NMR** (400 MHz, $CDCl_3$) of compound *trans*-**3r.**

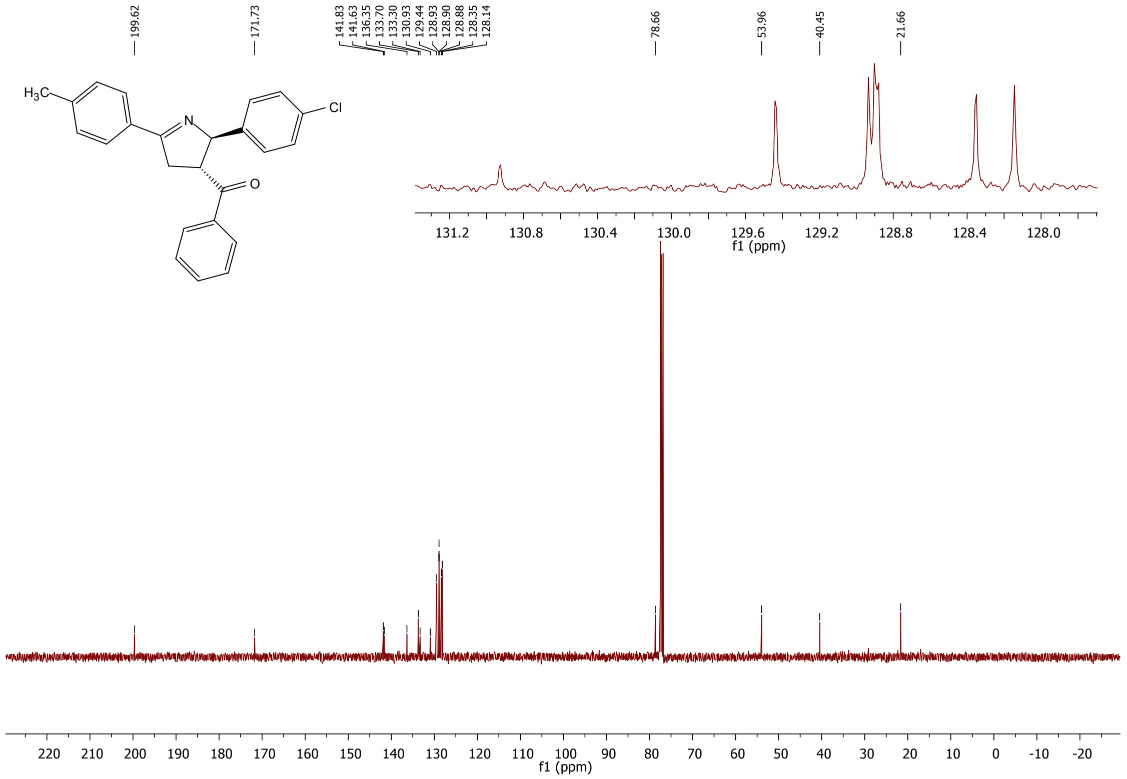

Figure S88. ^{13}**C{^{1}H} NMR** (100 MHz, $CDCl_3$) of compound *trans*-**3r.**

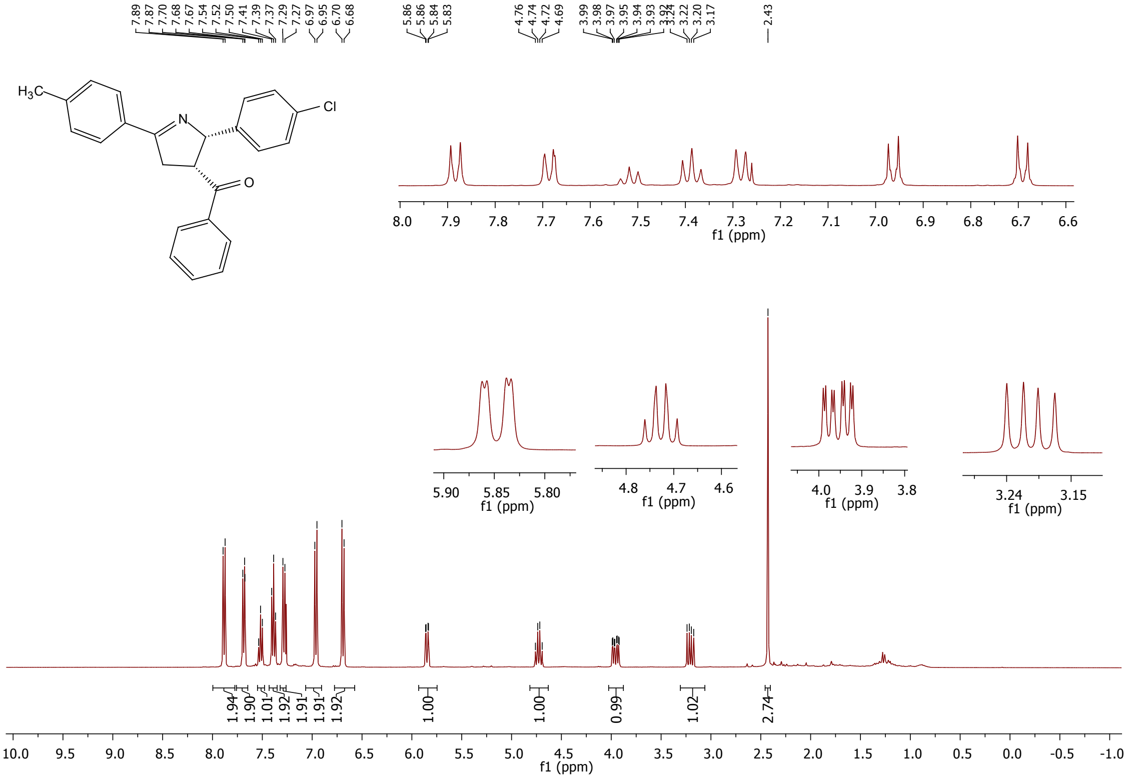

Figure S89. ^{1}H NMR (400 MHz, $CDCl_3$) of compound *cis*-**3r.**

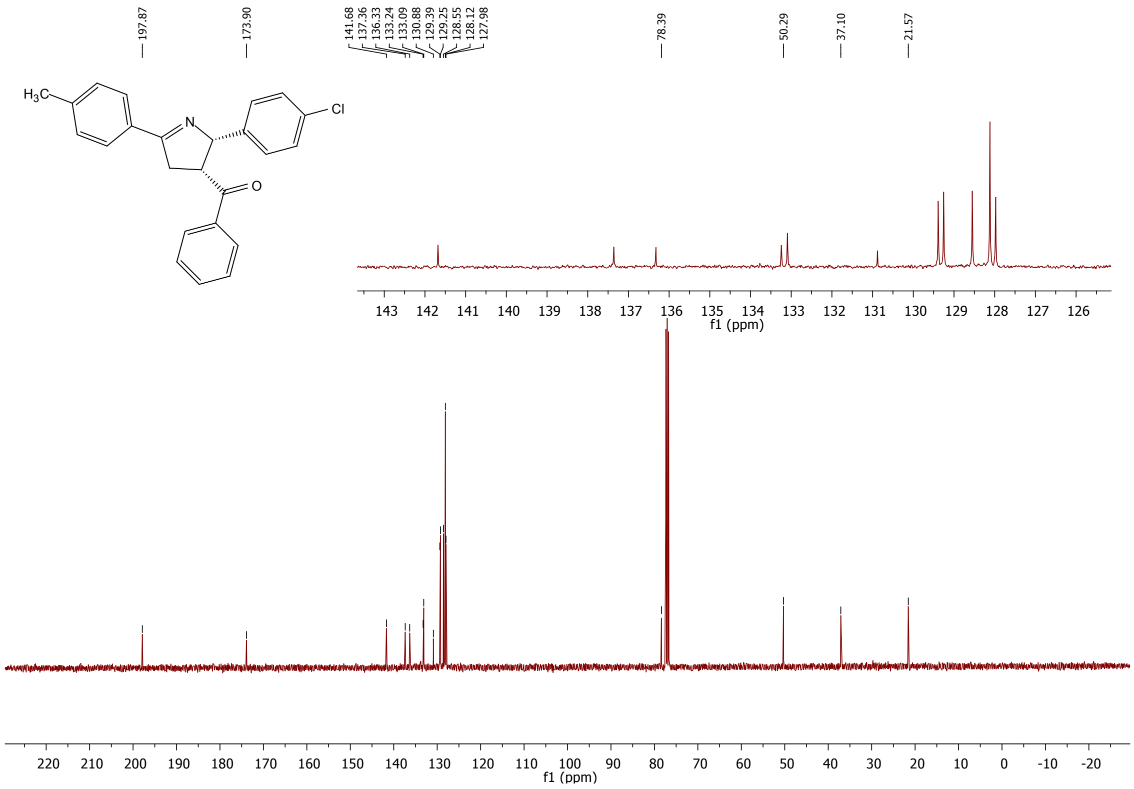

Figure S90. $^{13}C\{^1H\}$ NMR (100 MHz, $CDCl_3$) of compound *cis*-**3r.**

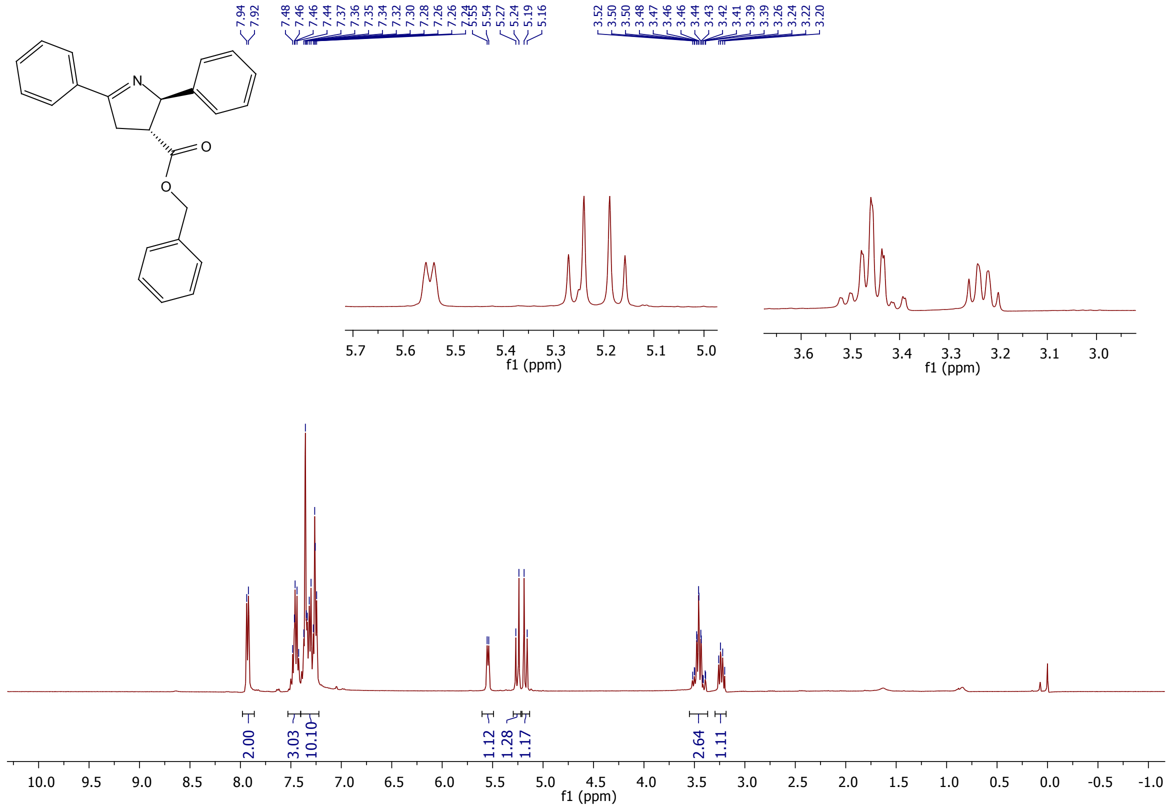

Figure S91. ^{1}H NMR (400 MHz, $CDCl_3$) of compound *trans*-**3s.**

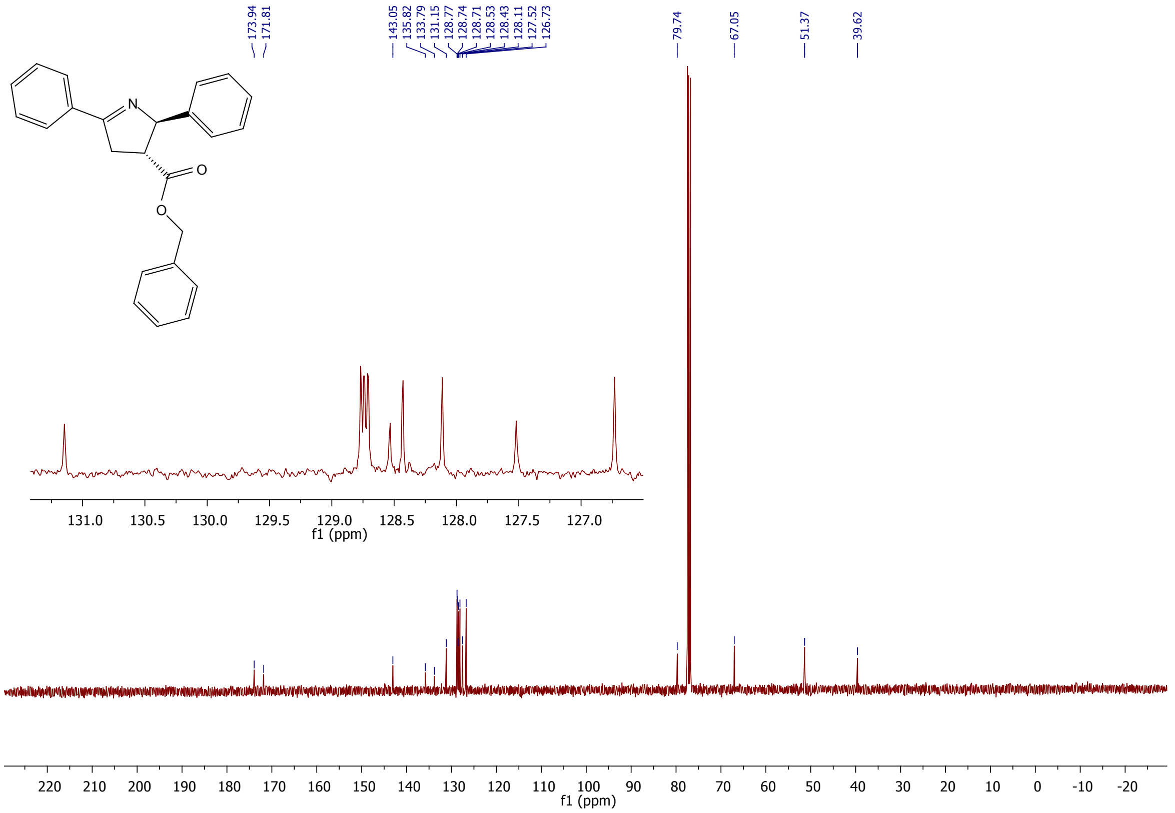

Figure S92. $^{13}C\{^{1}H\}$ NMR (100 MHz, $CDCl_3$) of compound *trans*-**3s.**

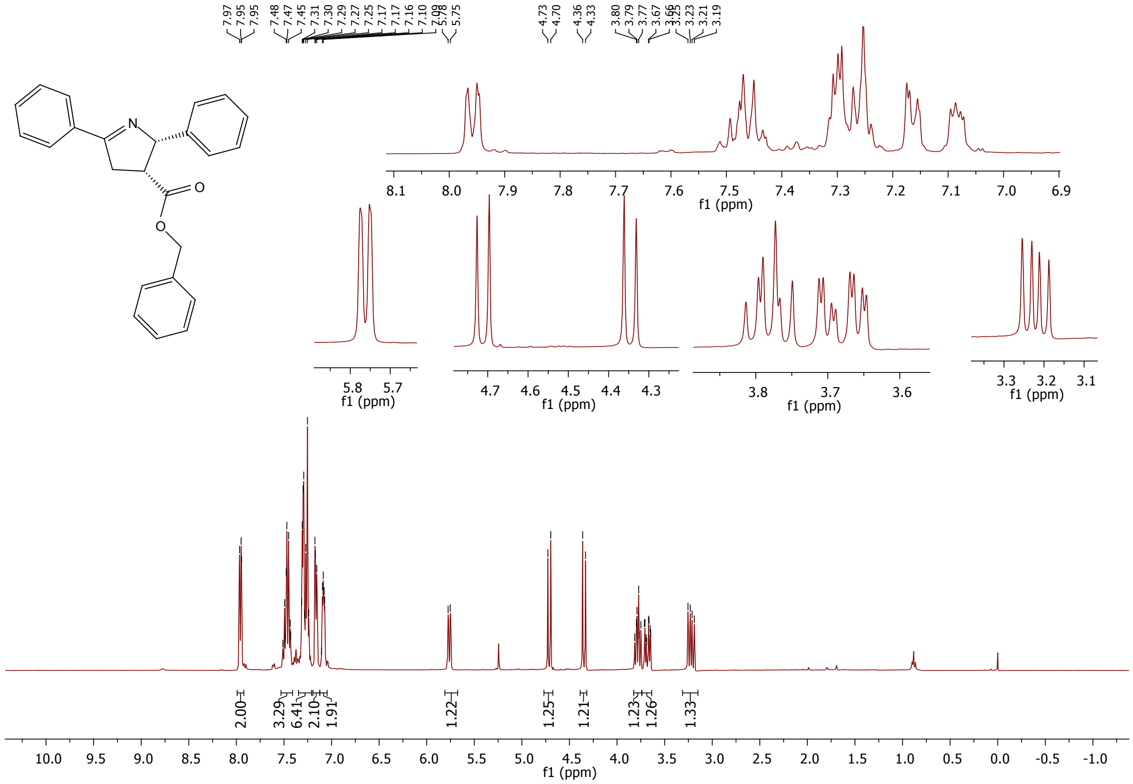

Figure S93. ^{1}H NMR (400 MHz, $CDCl_3$) of compound *cis*-**3s.**

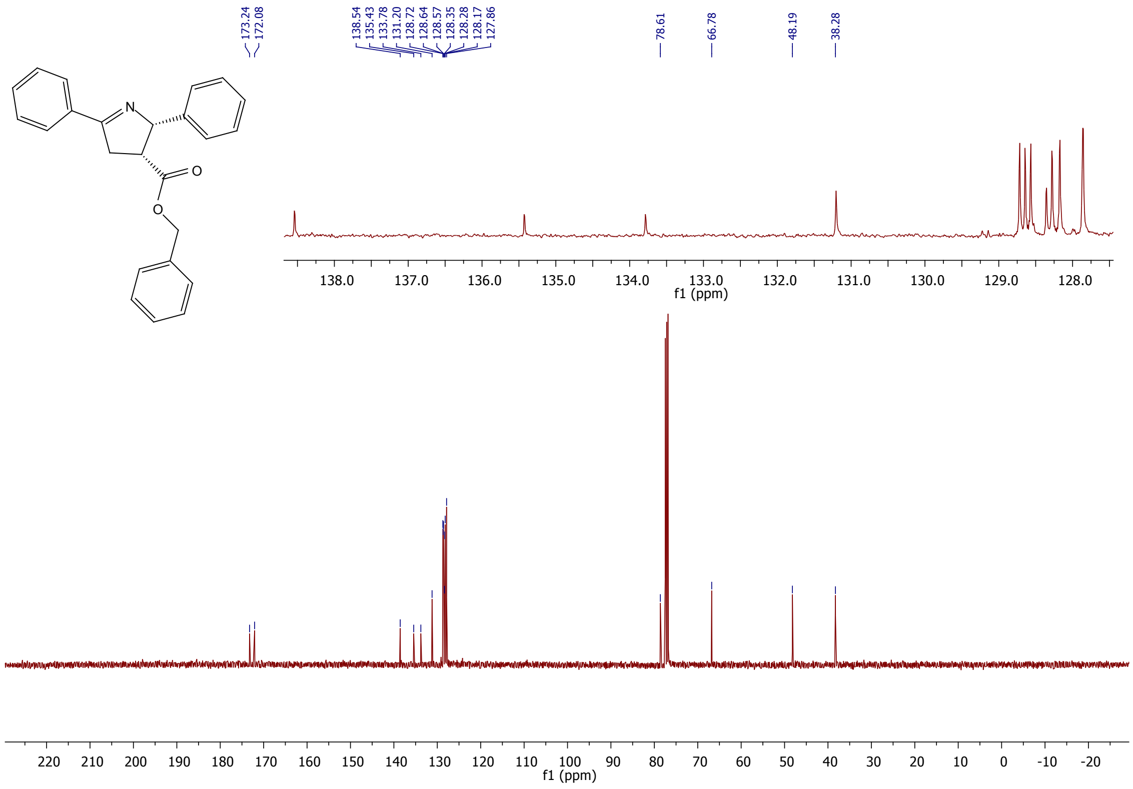

Figure S94. $^{13}C\{^{1}H\}$ NMR (100 MHz, $CDCl_3$) of compound *cis*-**3s.**

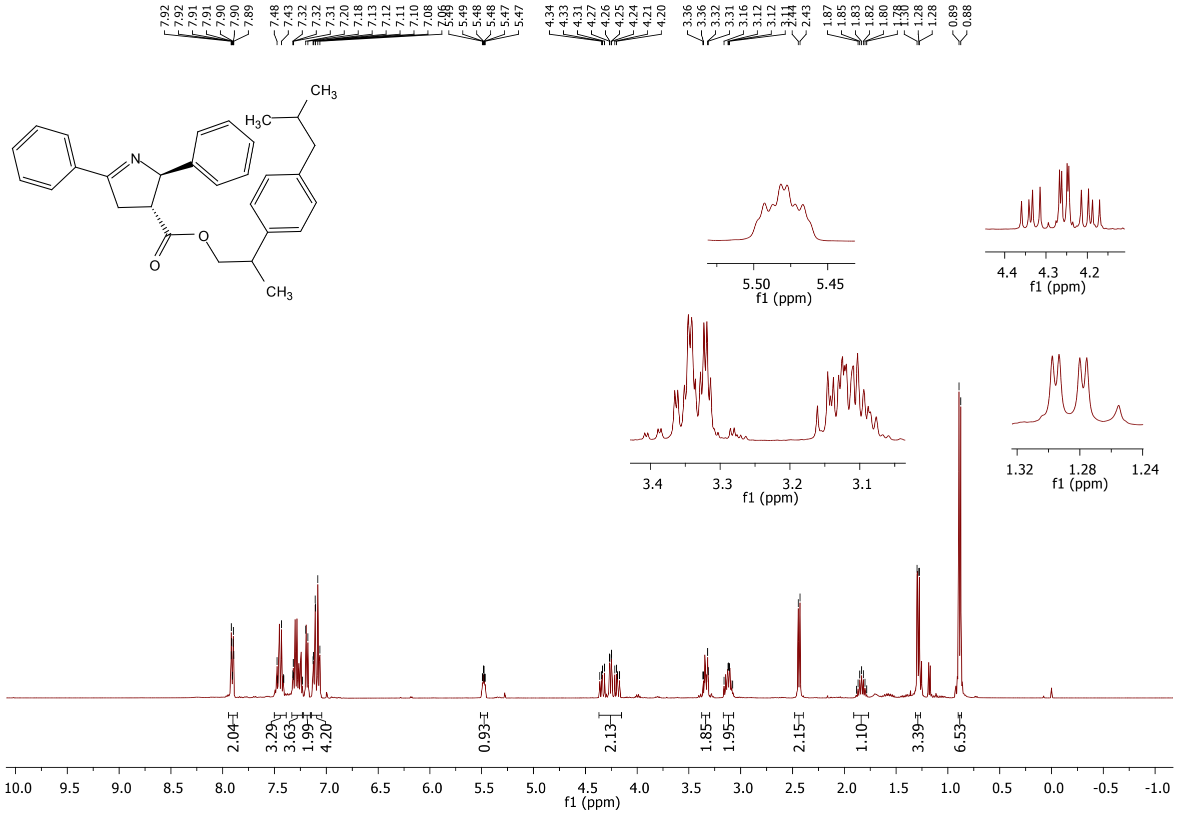

Figure S95. ^{1}H NMR (400 MHz, $CDCl_3$) of compound *trans*-**3t.**

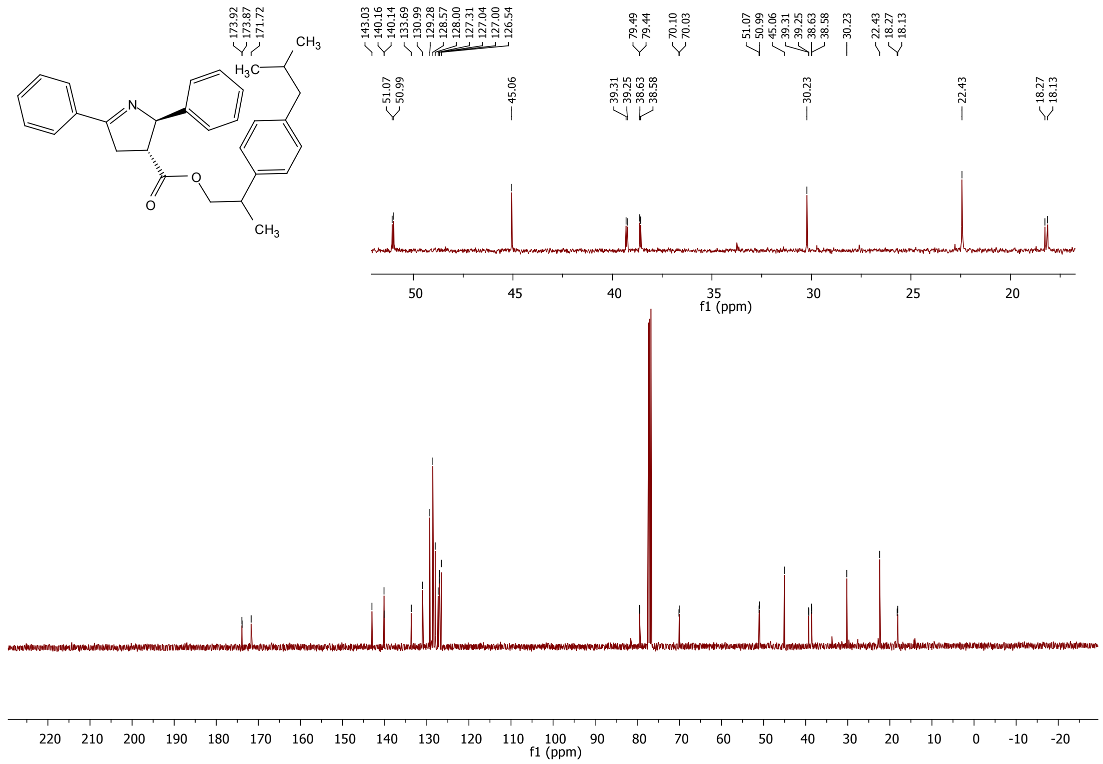

Figure S96. $^{13}C\{^{1}H\}$ NMR (100 MHz, $CDCl_3$) of compound *trans*-**3t.**

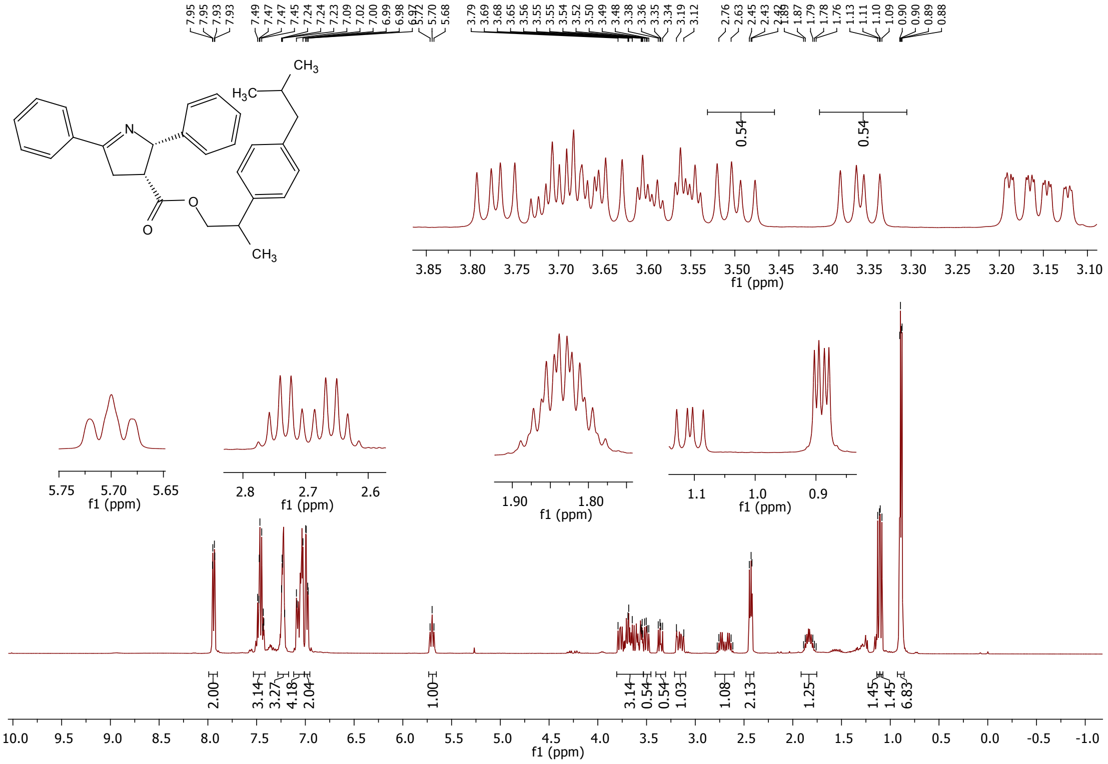

Figure S97. ^{1}H NMR (400 MHz, $CDCl_3$) of compound *cis*-**3t.**

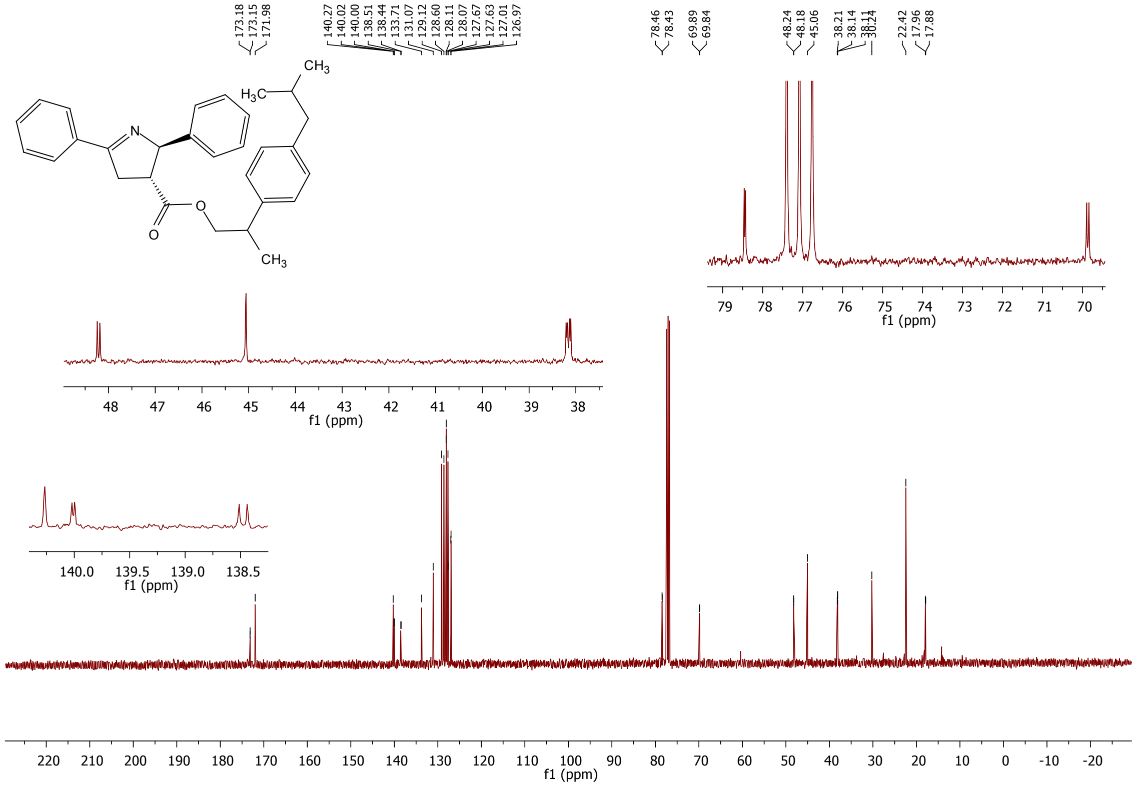

Figure S98. $^{13}C\{^{1}H\}$ NMR (100 MHz, $CDCl_3$) of compound *cis*-**3t.**

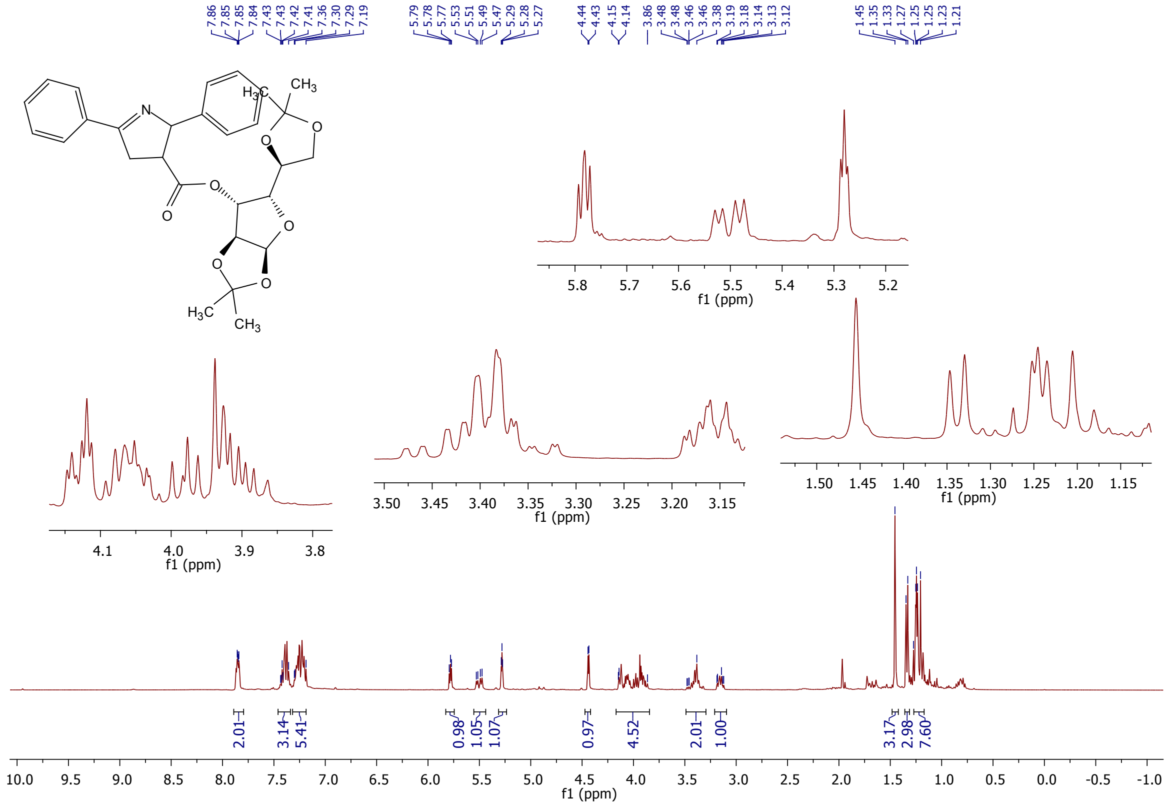

Figure S99. ^{1}H NMR (400 MHz, $CDCl_3$) of compound *trans*-**3u.**

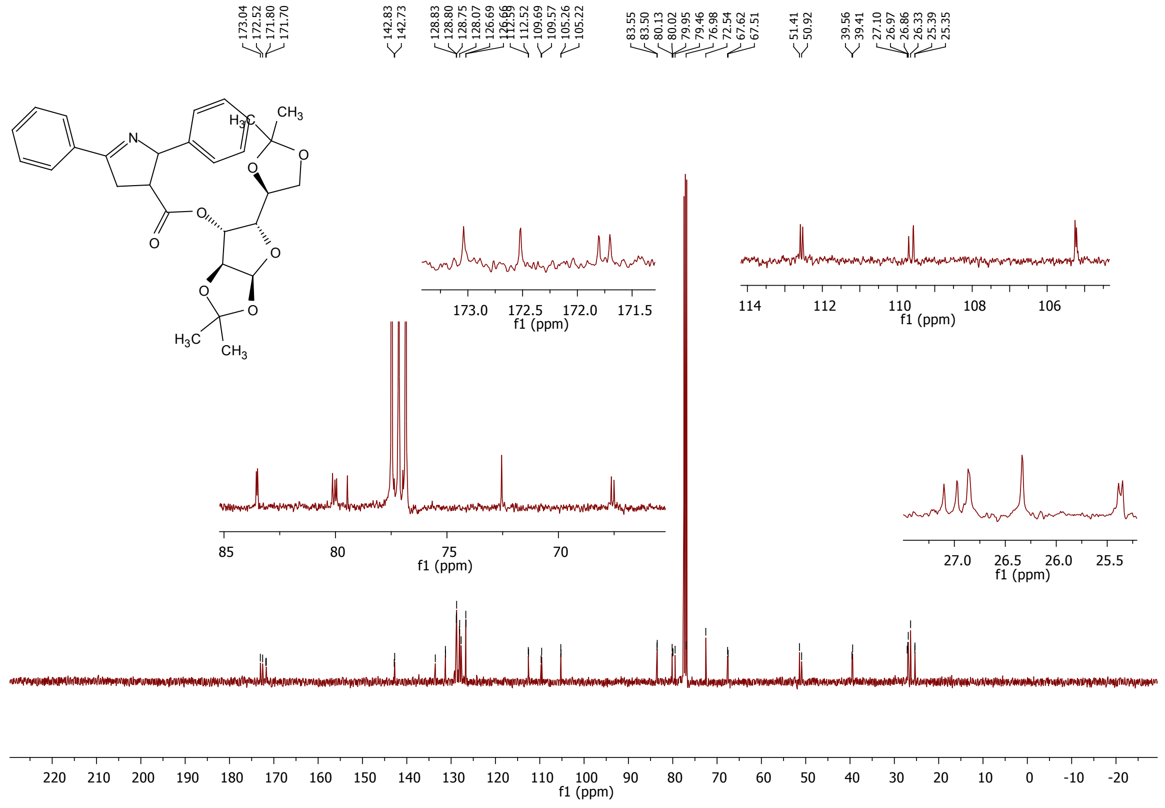

Figure S100. $^{13}C\{^{1}H\}$ NMR (100 MHz, $CDCl_3$) of compound *trans*-**3u.**

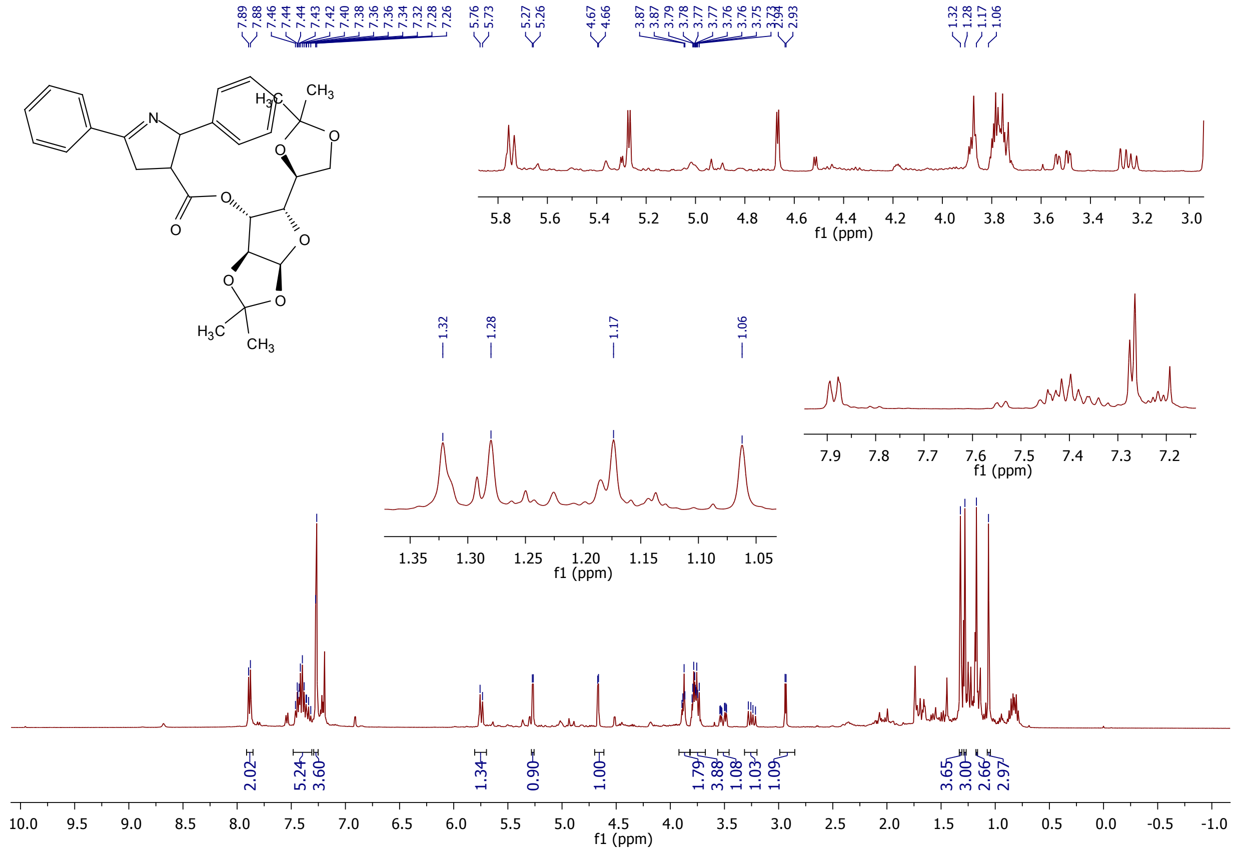

Figure S101. ^{1}H NMR (400 MHz, $CDCl_3$) of compound *cis*-**3u.**

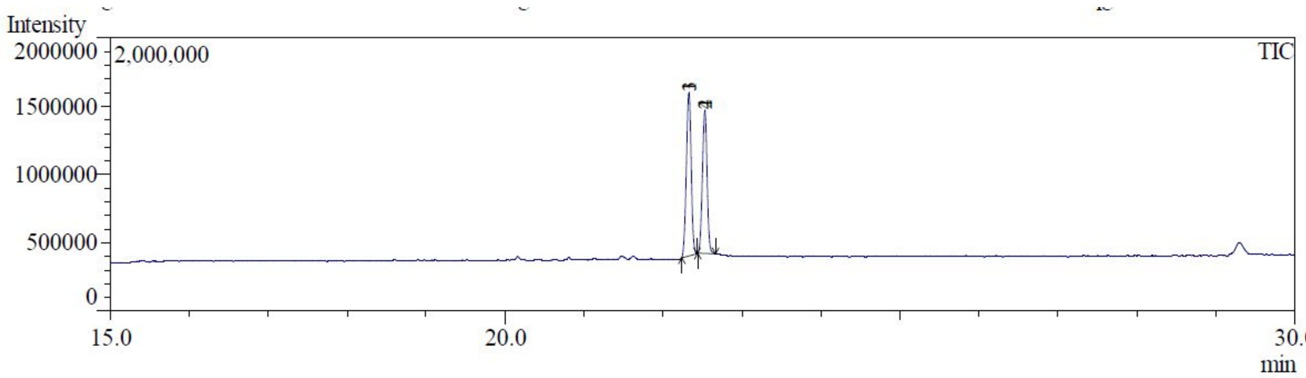

Peak Report TIC

Peak#	R.Time	Area	Area%	Name	Base m/z	Base Int.
1	22.331	5083605	54.05		220.15	119298
2	22.532	4321038	45.95		220.15	102304
		9404643	100.00			

[GC-2010]

Column Oven Temp.	:50.0 °C	
Injection Temp.	:250.00 °C	
Injection Mode	:Split	
Flow Control Mode	:Linear Velocity	
Pressure	:22.4 kPa	
Total Flow	:16.4 mL/min	
Column Flow	:0.64 mL/min	
Linear Velocity	:29.0 cm/sec	
Purge Flow	:3.0 mL/min	
Split Ratio	:20.0	
High Pressure Injection	:OFF	
Carrier Gas Saver	:OFF	
Splitter Hold	:OFF	
Oven Temp. Program		
Rate	Temperature(°C)	Hold Time(min)
-	50.0	2.50
20.00	300.0	15.00

Figure S102. GC-MS analysis (Zebron-ZB-5MS Column) of compound **3u**.

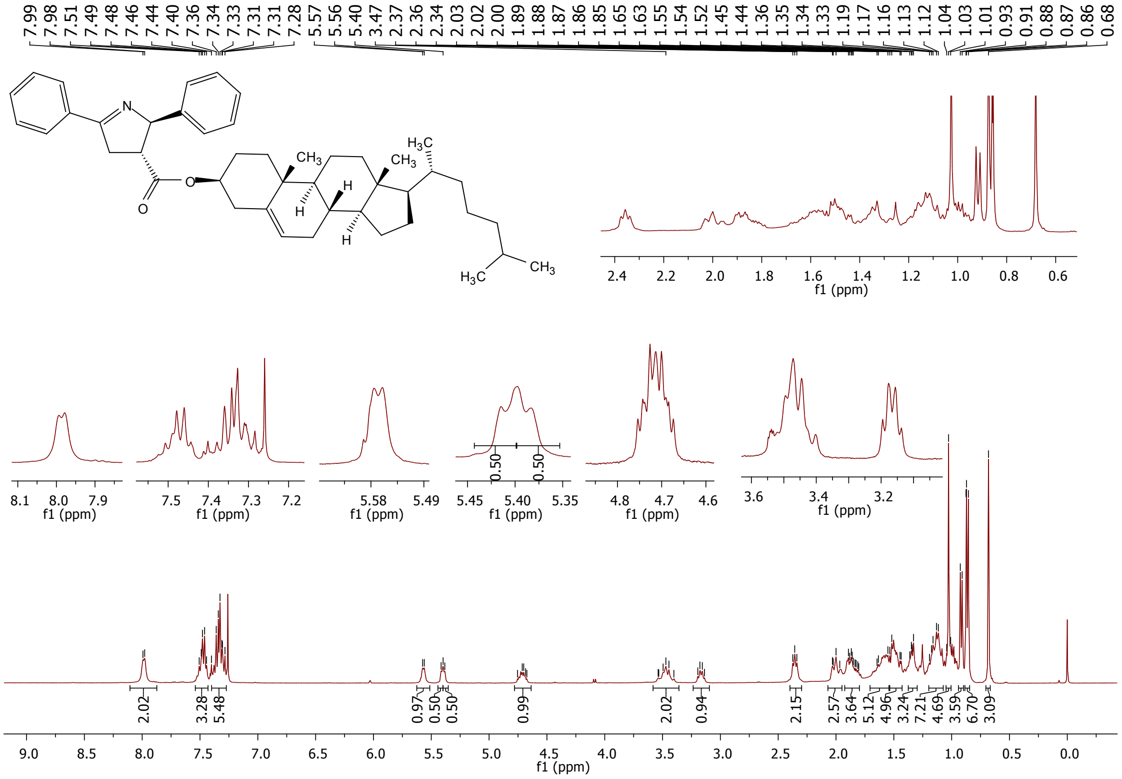

Figure S103. **^{1}H NMR** (400 MHz, $CDCl_3$) of compound *trans*-**3v.**

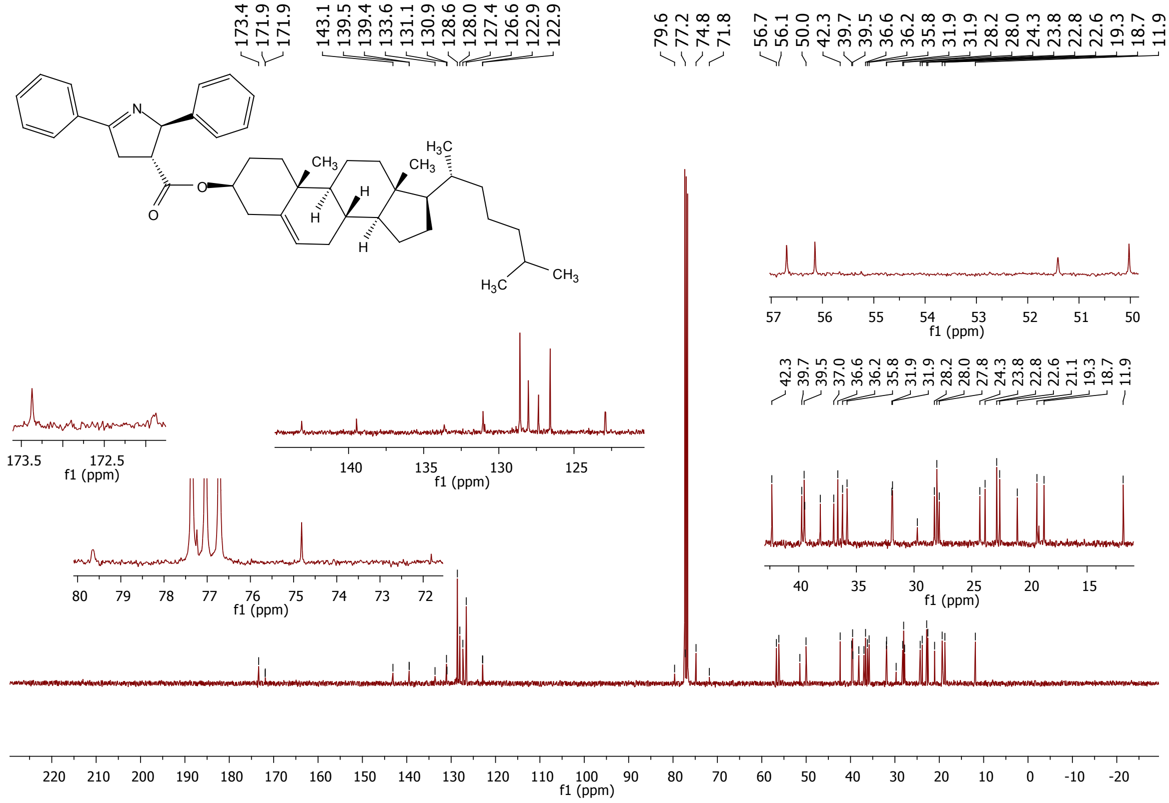

Figure S104. $^{13}C\{^1H\}$ **NMR** (100 MHz, $CDCl_3$) of compound *trans*-**3v.**

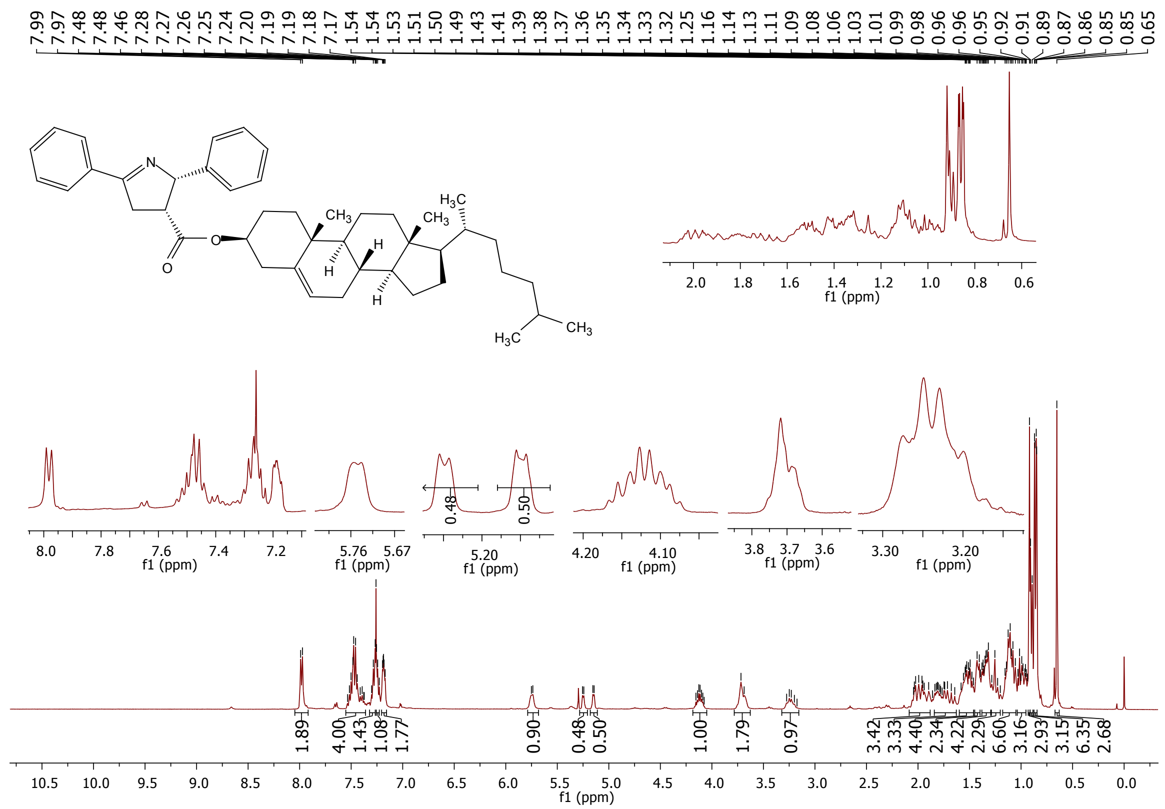

Figure S105. ^{1}H **NMR** (400 MHz, $CDCl_3$) of compound *cis*-**3v.**

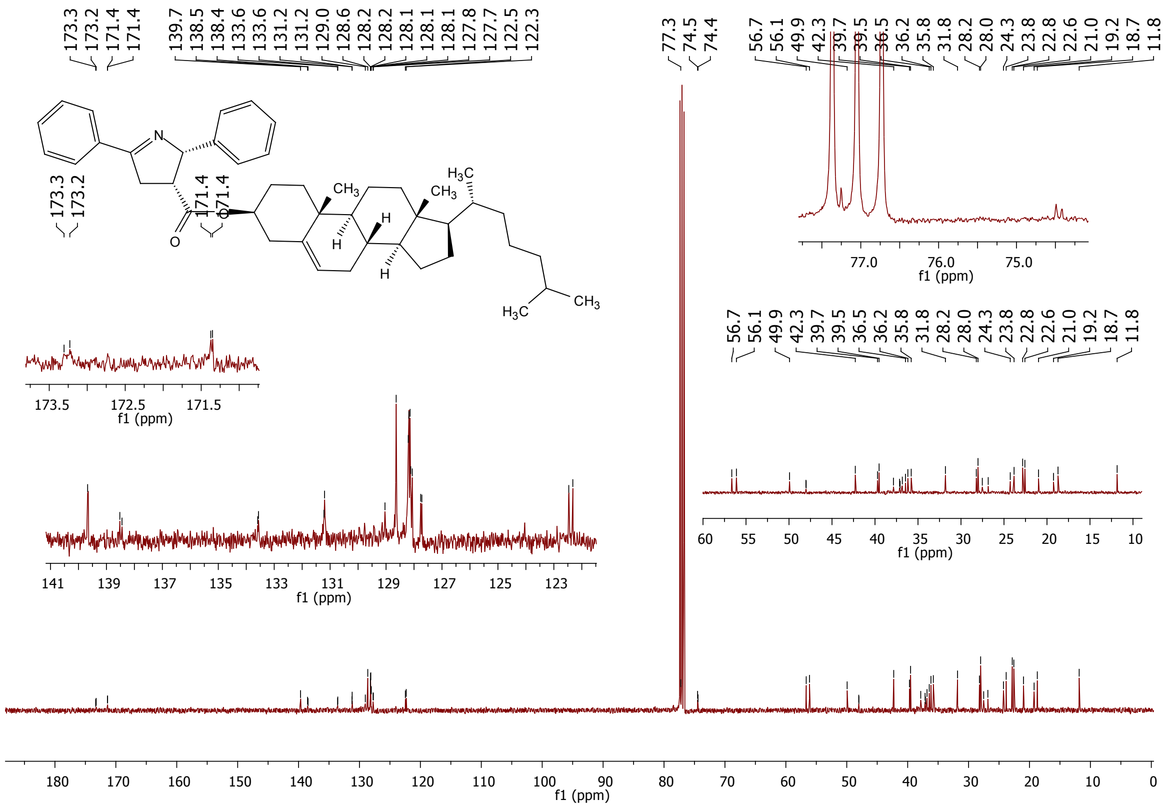

Figure S106. $^{13}C\{^1H\}$ NMR (100 MHz, $CDCl_3$) of compound *cis*-**3v.**

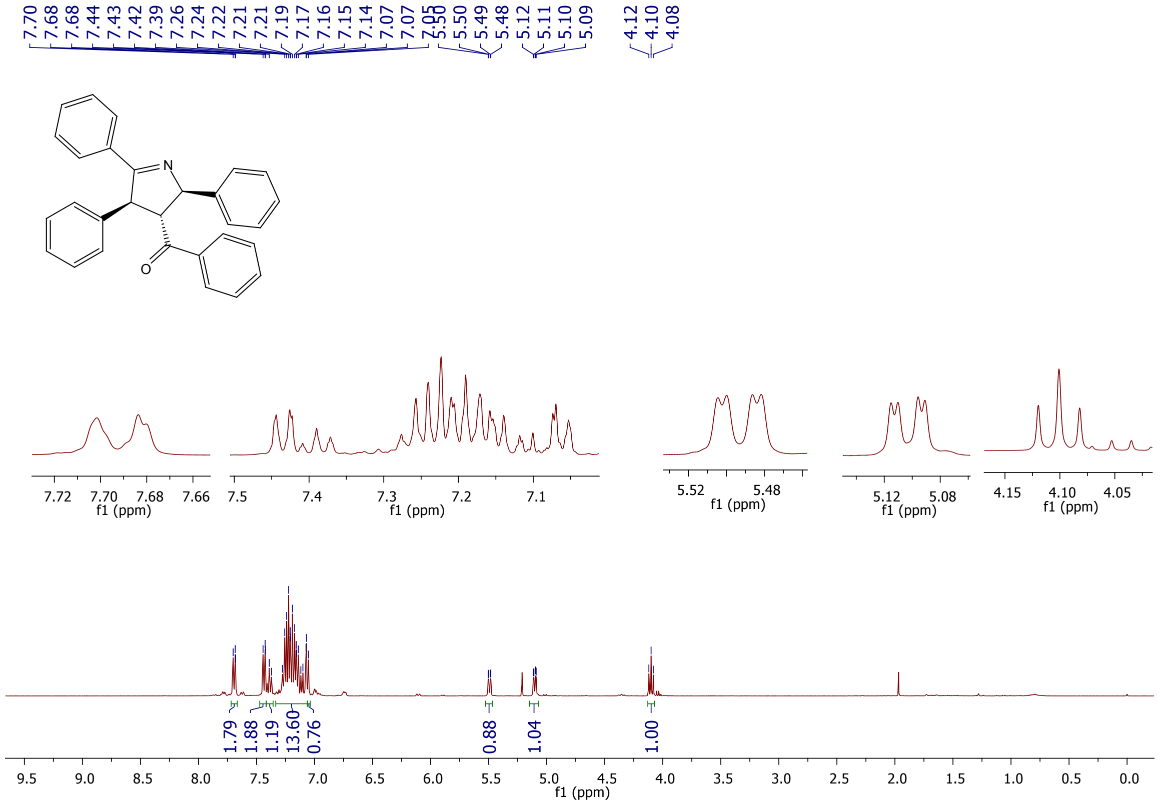

Figure S107. ^1H NMR (400 MHz, $CDCl_3$) of compound *anti/anti*-**3aa.**

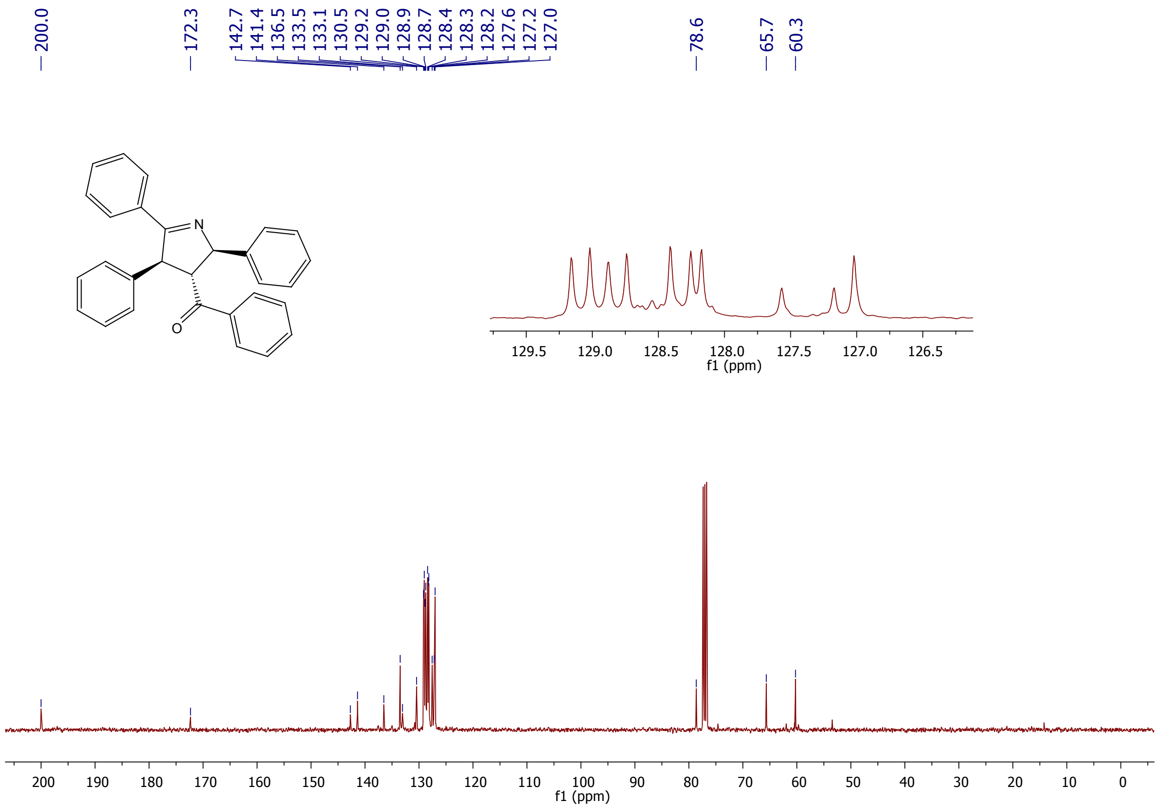

Figure S108. **$^{13}C\{^{1}H\}$ NMR** (100 MHz, $CDCl_3$) of compound *anti/anti*-**3aa.**

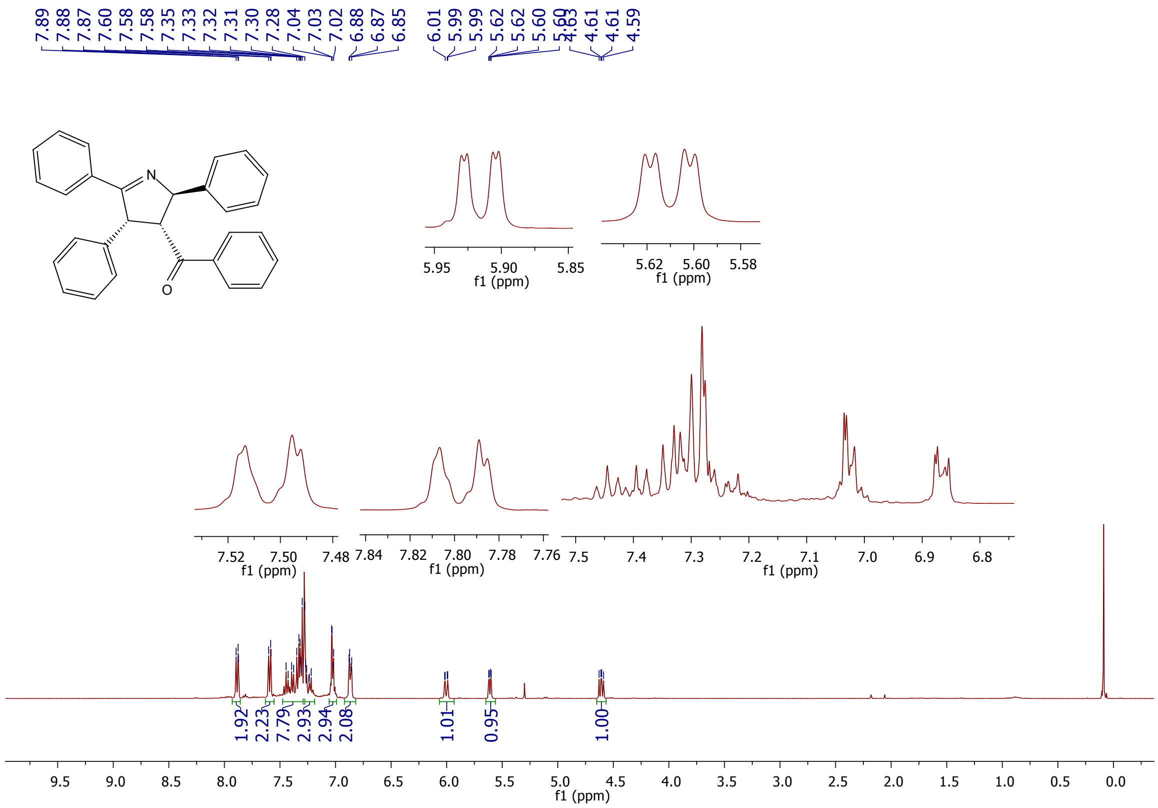

Figure S109. **^{1}H NMR** (400 MHz, $CDCl_3$) of compound *anti/syn*-**3aa.**

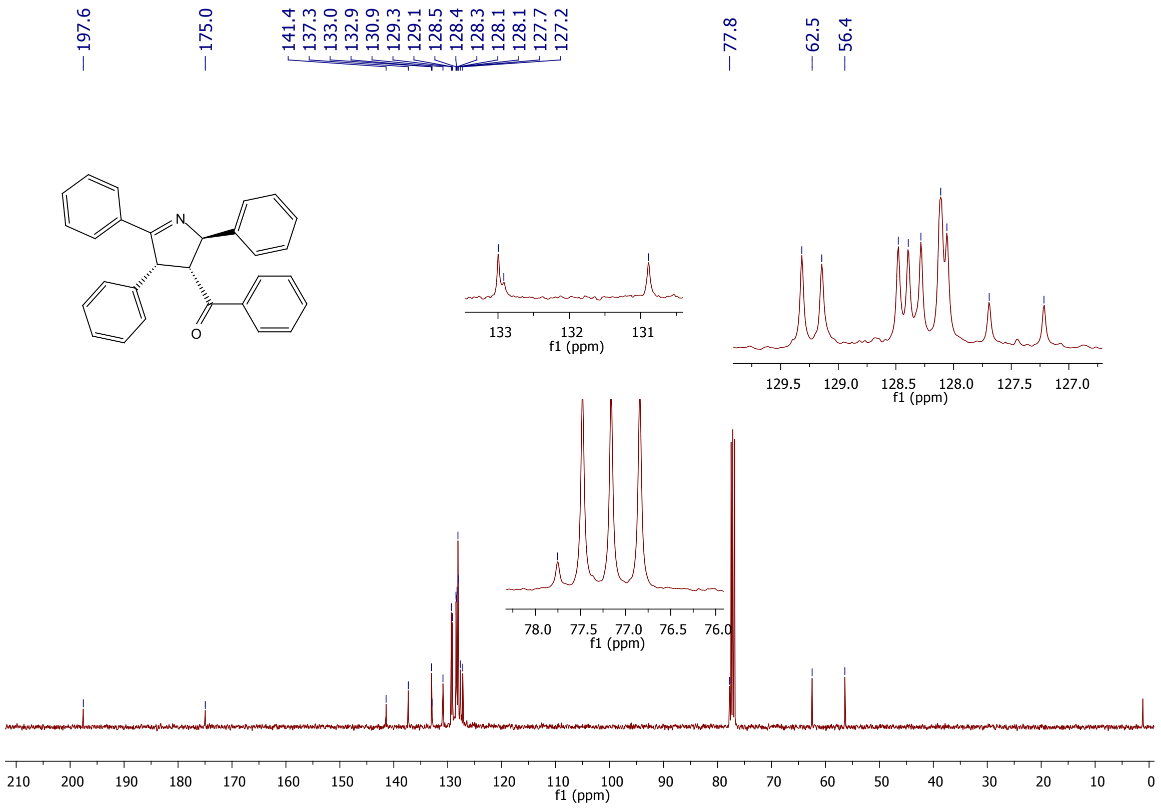

Figure S110. $^{13}C\{^{1}H\}$ **NMR** (100 MHz, $CDCl_3$) of compound *anti/syn*-**3aa.**

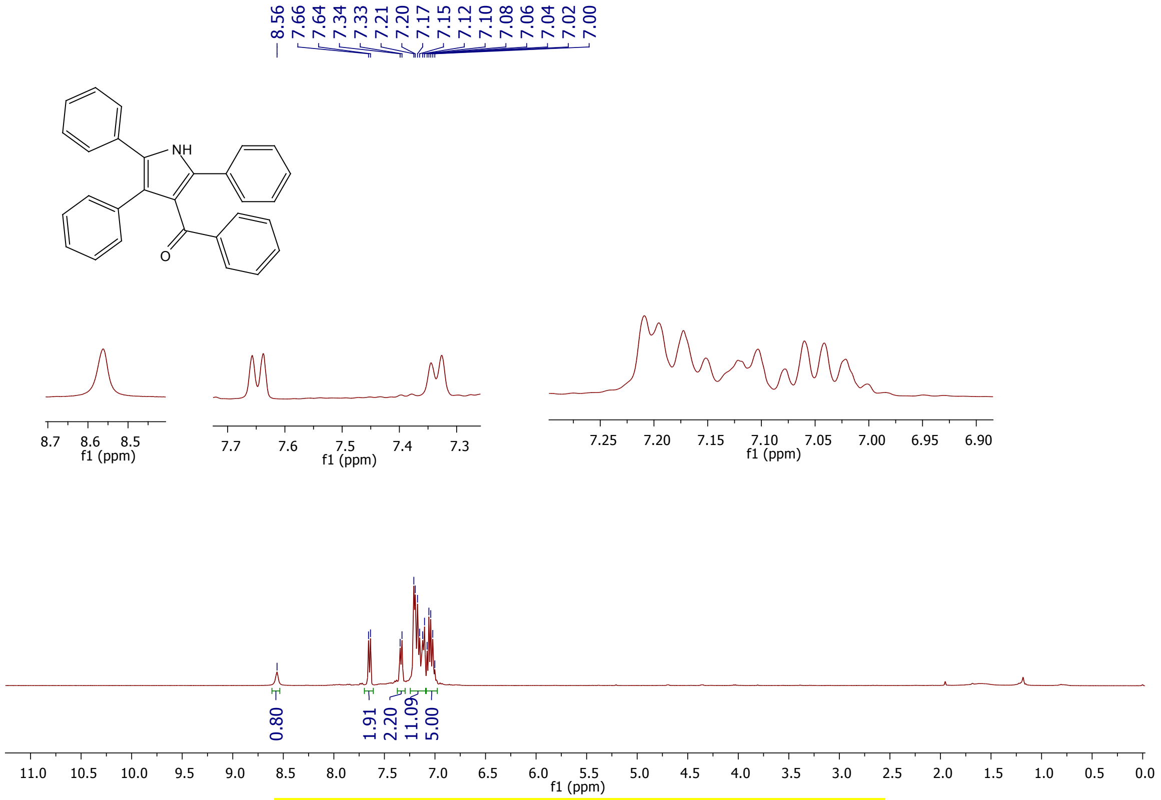

Figure S111. ^{1}H **NMR** (400 MHz, $CDCl_3$) of compound **5a.**

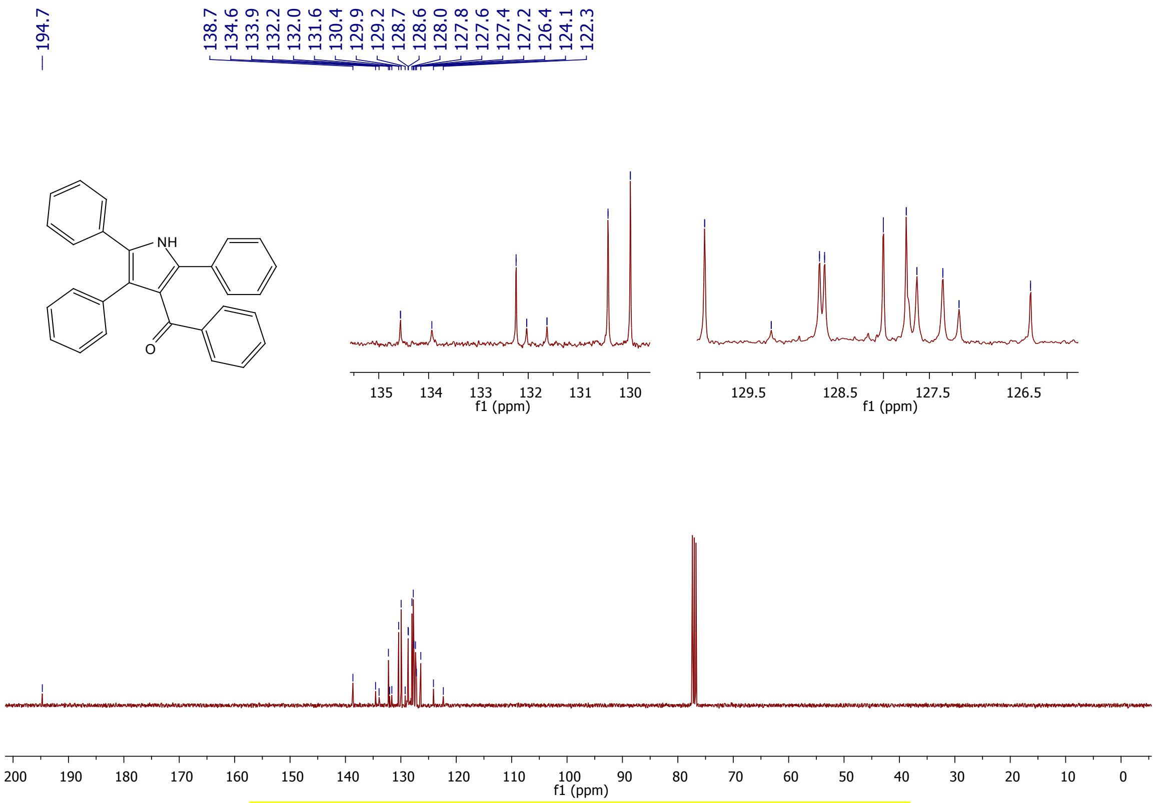

Figure S112. **$^{13}C\{^1H\}$ NMR** (100 MHz, $CDCl_3$) of compound **5a.**

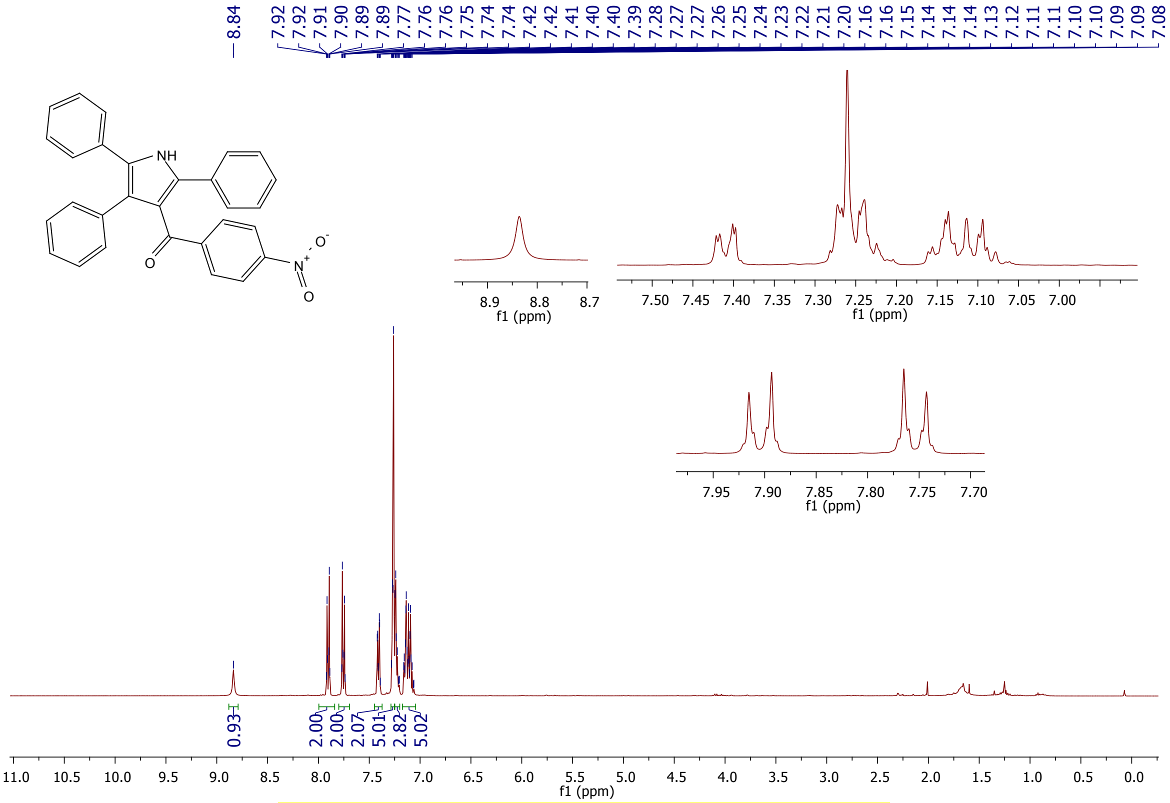

Figure S113. **^1H NMR** (400 MHz, $CDCl_3$) of compound **5b.**

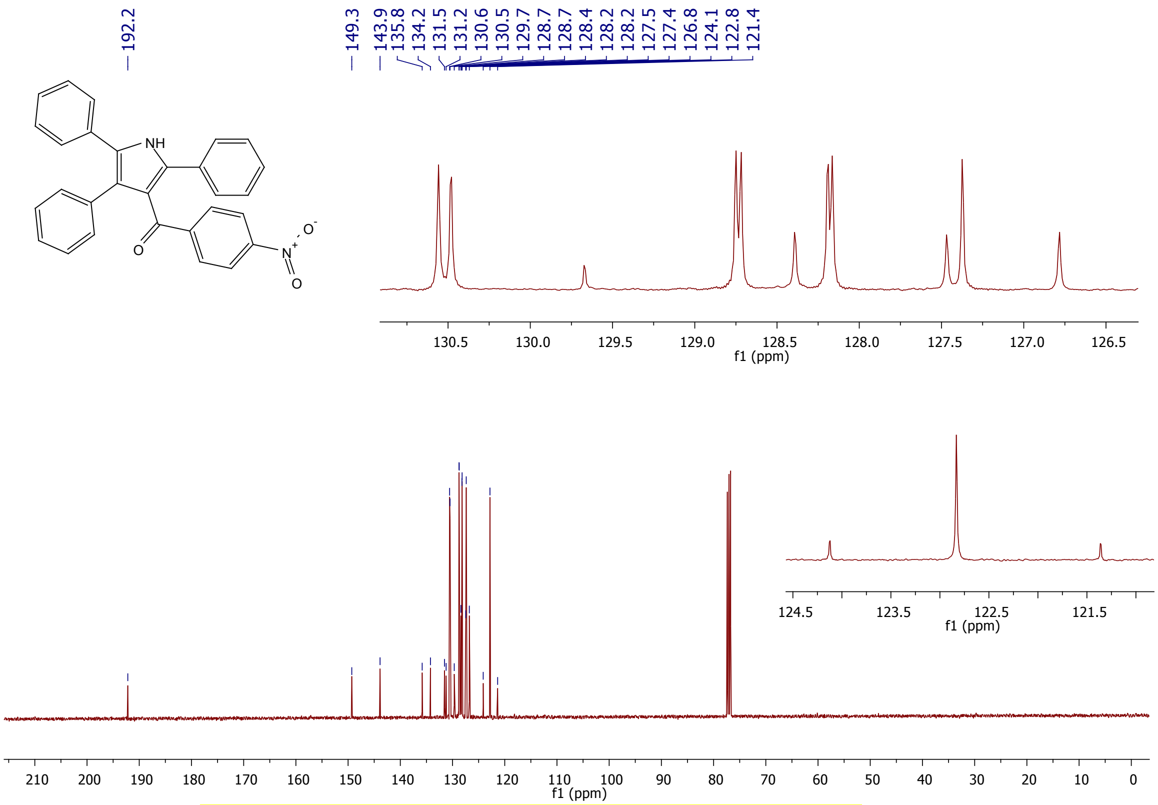

Figure S114. ^{13}C{^{1}H} NMR (100 MHz, $CDCl_3$) of compound **5b.**

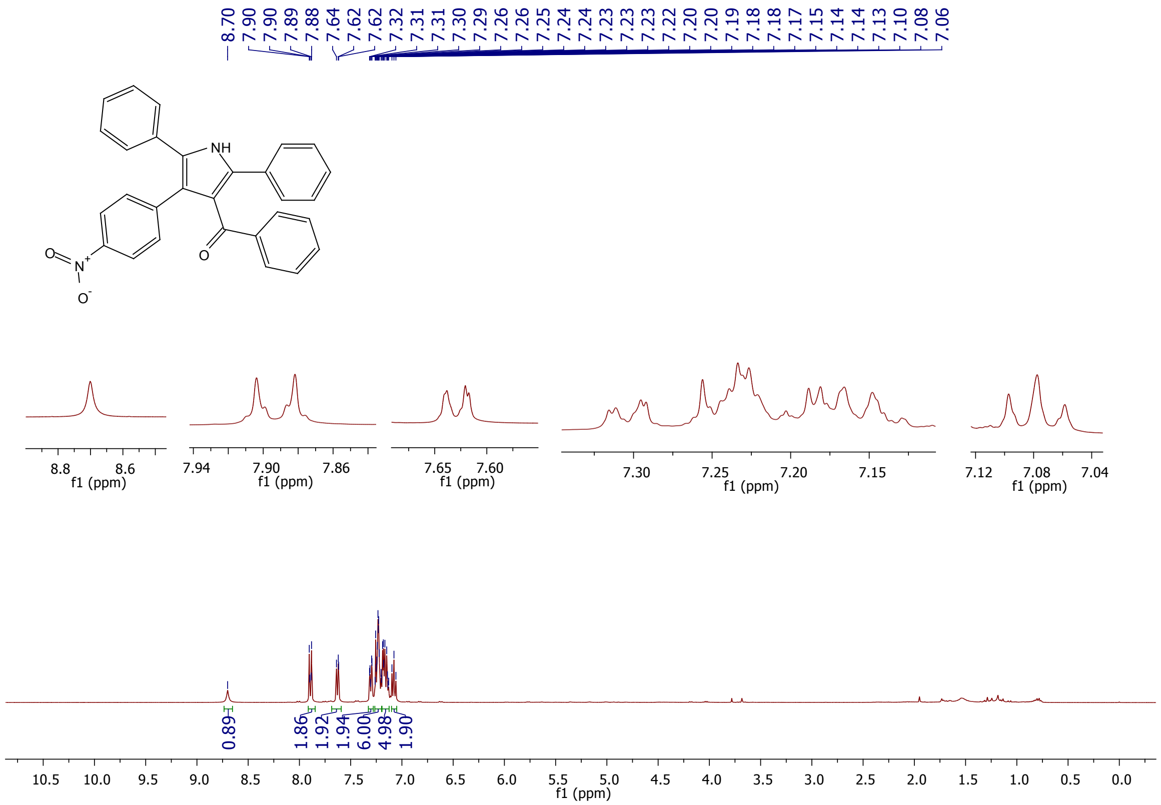

Figure S115. ^{1}H NMR (400 MHz, $CDCl_3$) of compound **5c.**

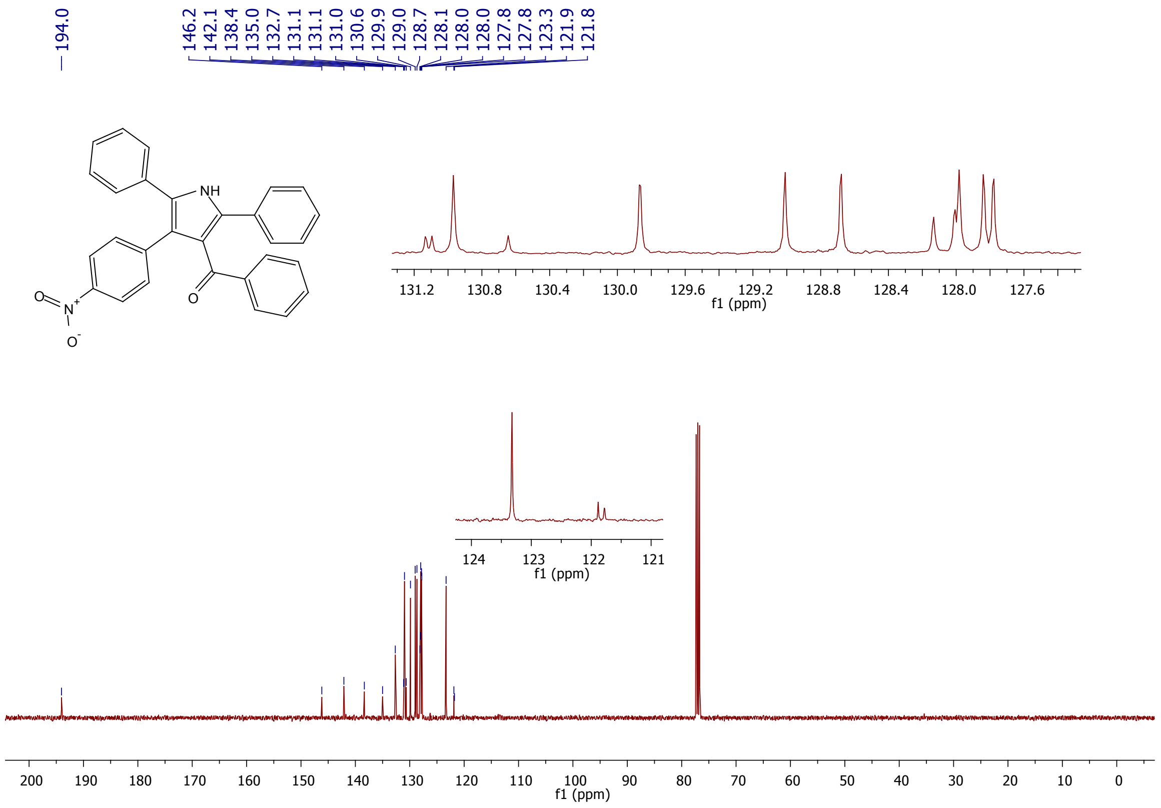

Figure S116. $^{13}C\{^1H\}$ NMR (100 MHz, $CDCl_3$) of compound **5c.**

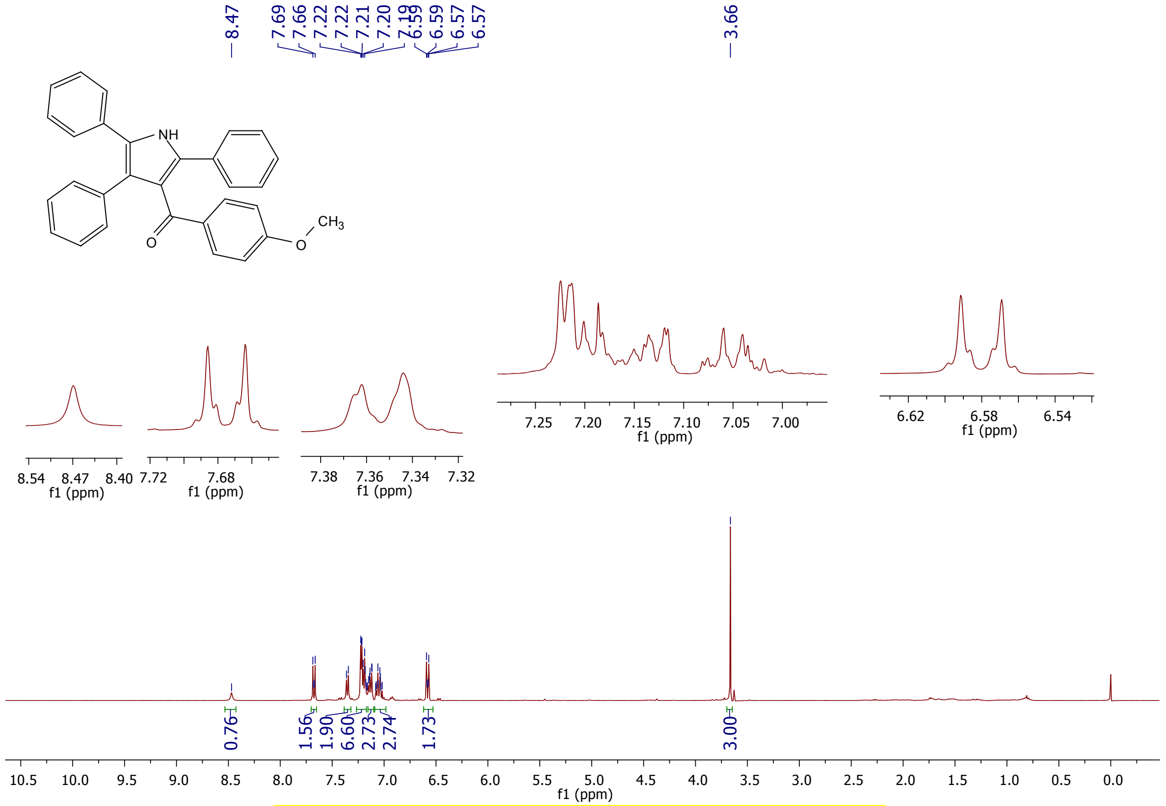

Figure S117. ^{1}H NMR (400 MHz, $CDCl_3$) of compound **5d.**

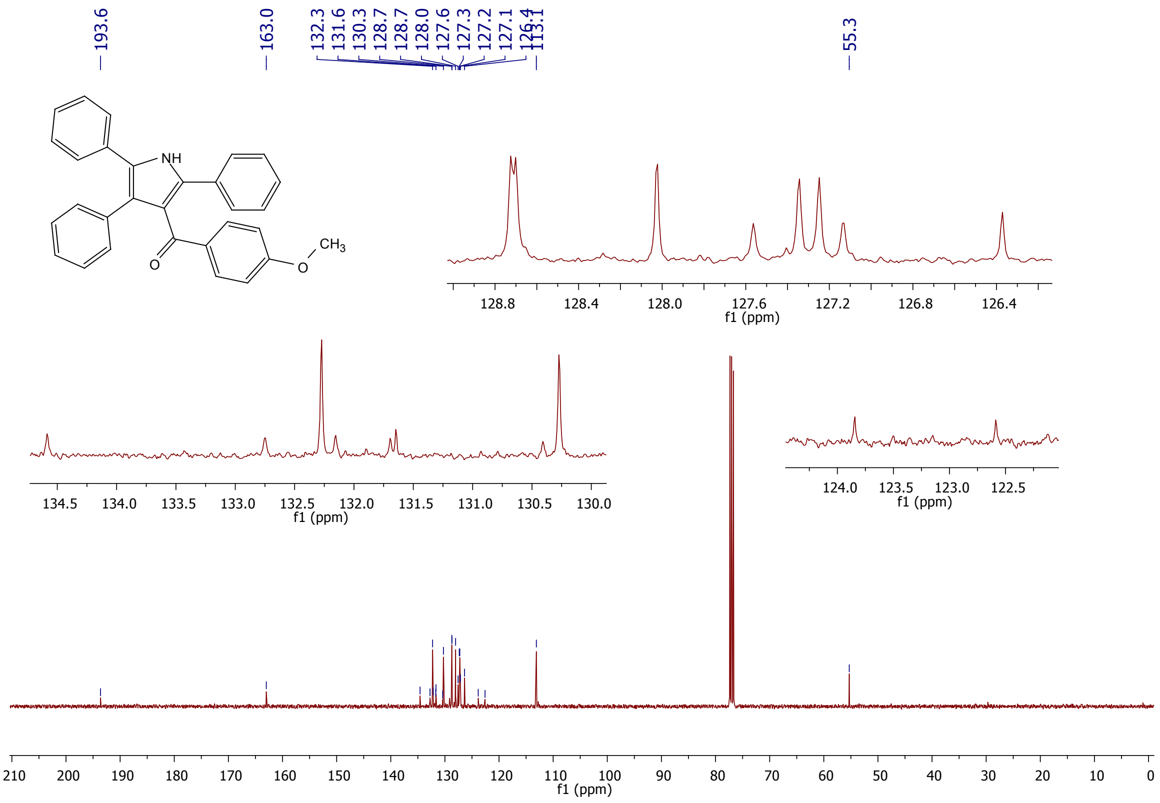

Figure S118. $^{13}C\{^1H\}$ **NMR** (100 MHz, $CDCl_3$) of compound **5d.**

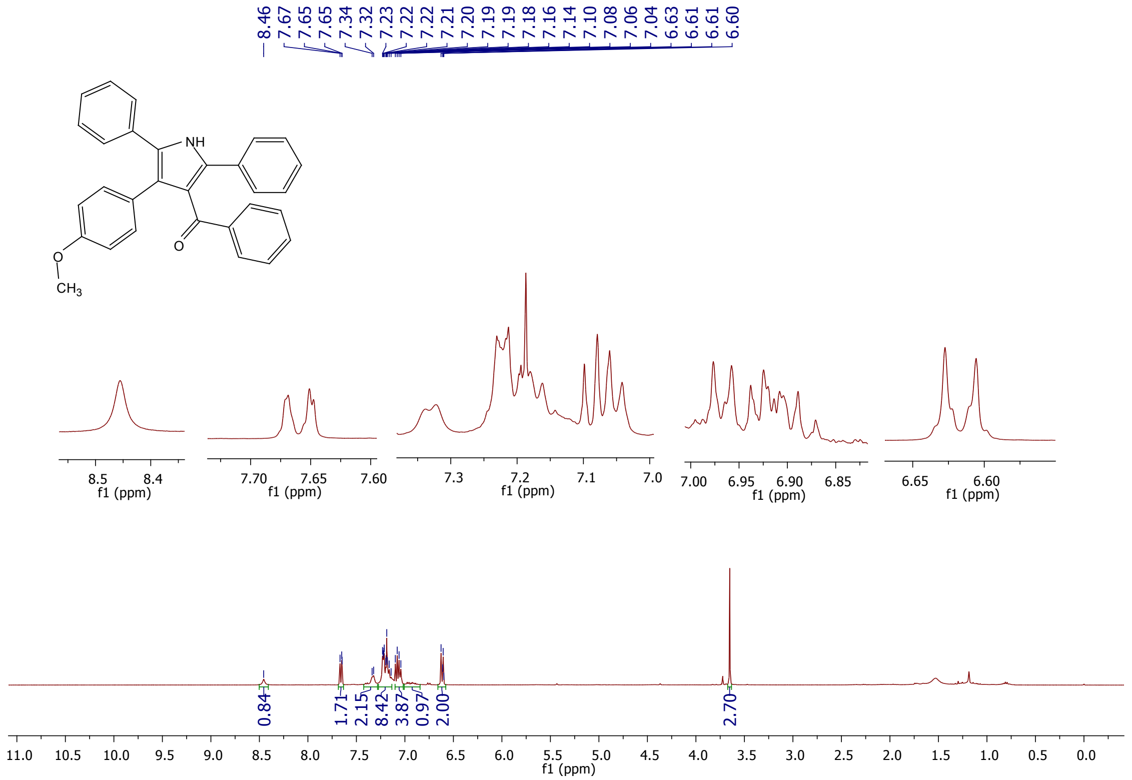

Figure S119. ^1H **NMR** (400 MHz, $CDCl_3$) of compound **5e.**

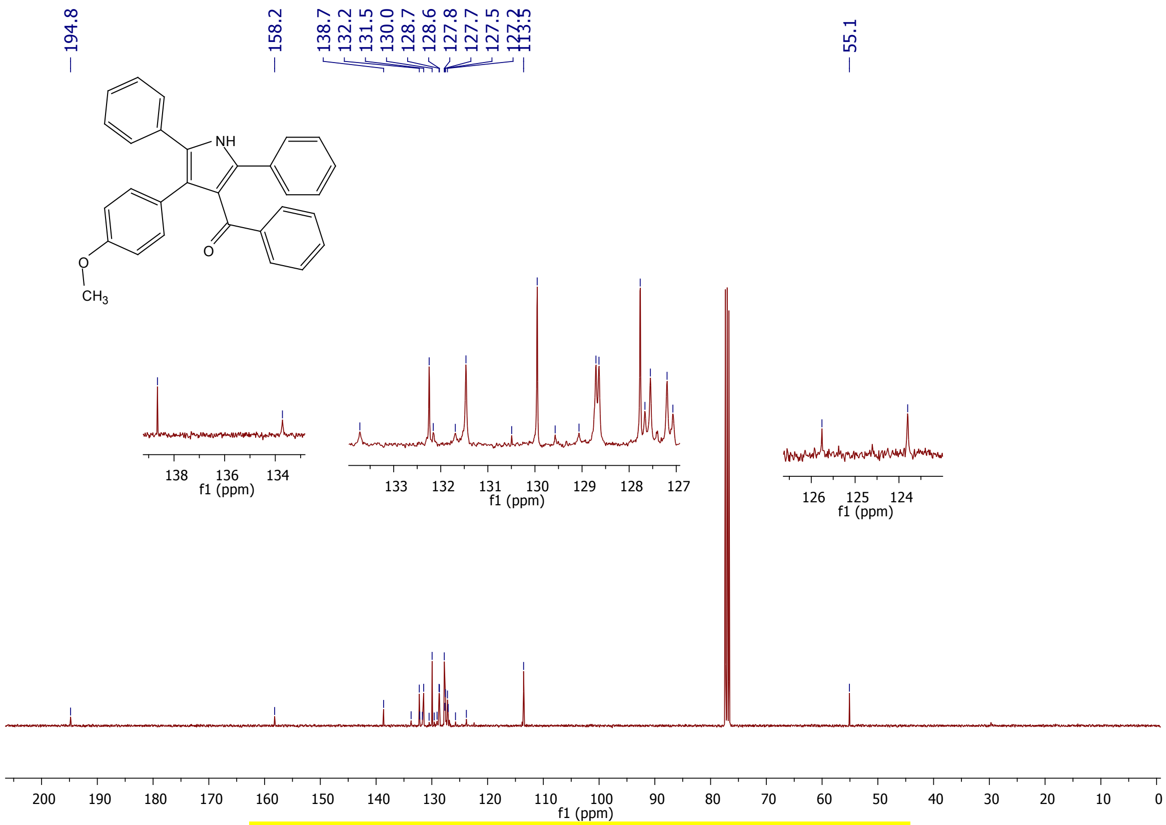

Figure S120. **$^{13}C\{^{1}H\}$ NMR** (100 MHz, $CDCl_3$) of compound **5e.**

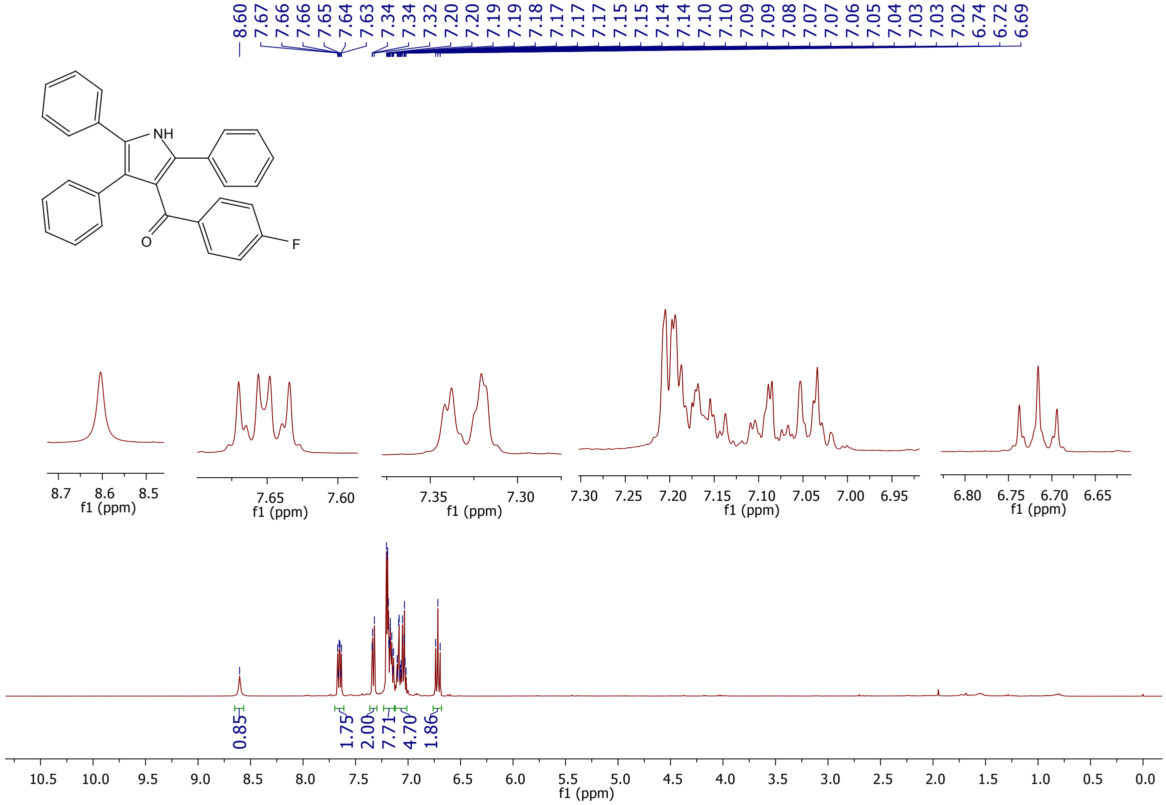

Figure S121. **^{1}H NMR** (400 MHz, $CDCl_3$) of compound **5f.**

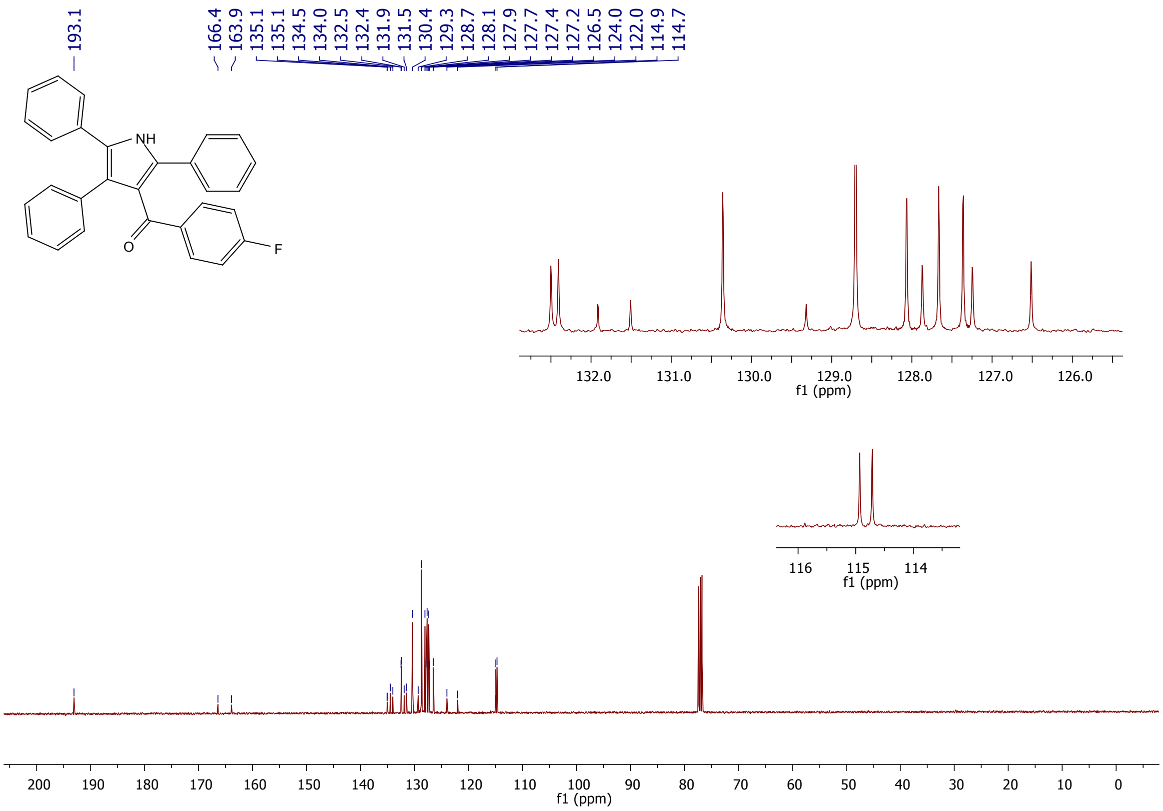

Figure S122. **$^{13}C\{^1H\}$ NMR** (100 MHz, $CDCl_3$) of compound **5f.**

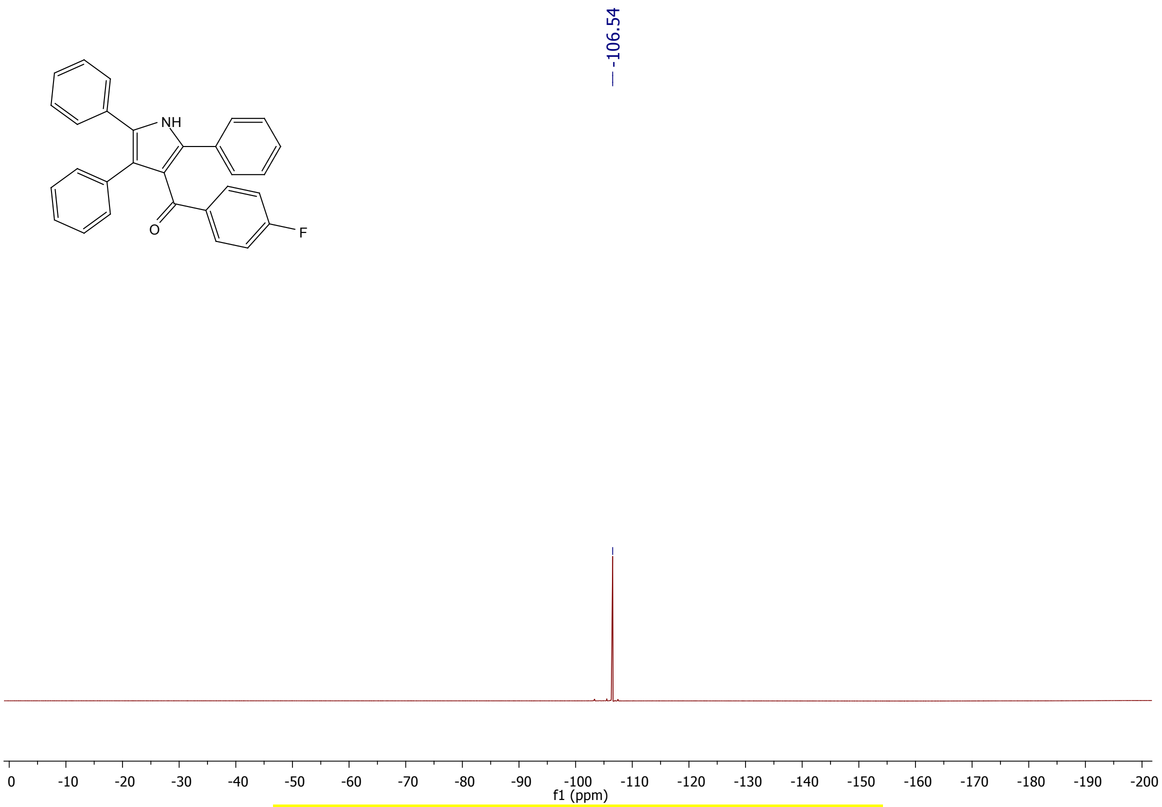

Figure S123. **^{19}F NMR** (376 MHz, $CDCl_3$) of compound **5f.**

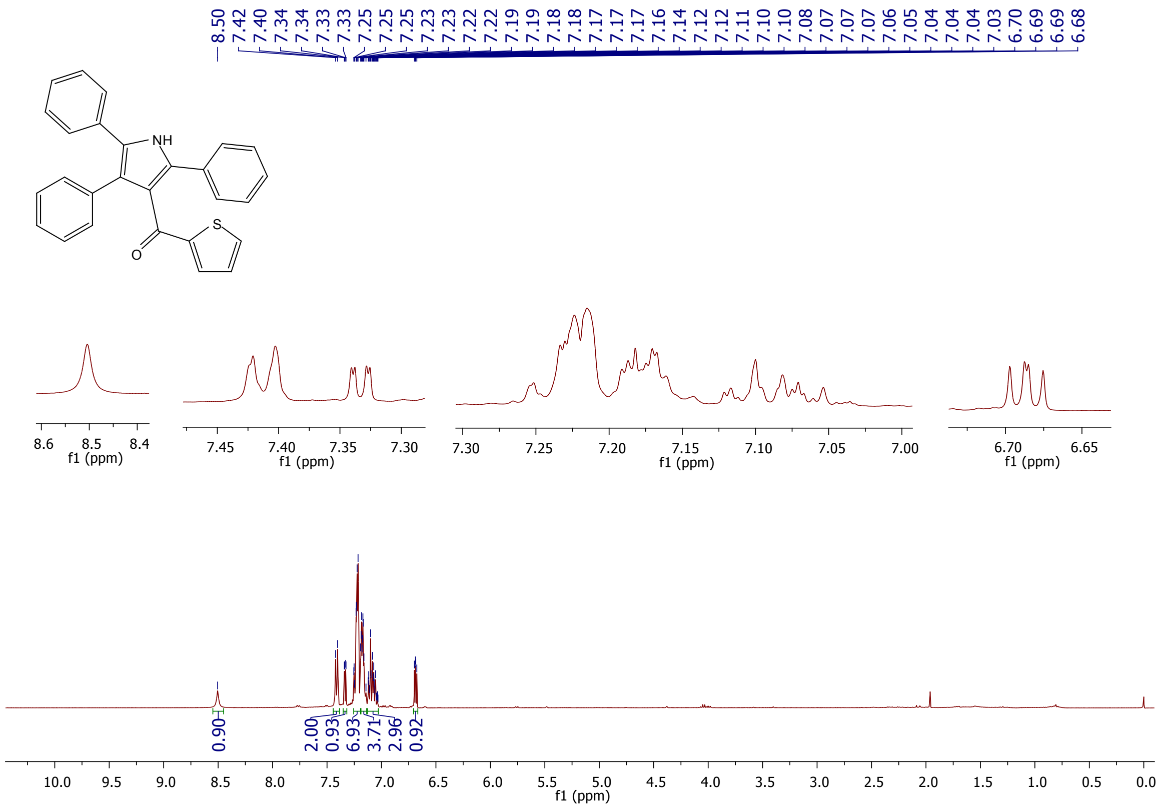

Figure S124. ^{1}**H NMR** (400 MHz, $CDCl_3$) of compound **5g.**

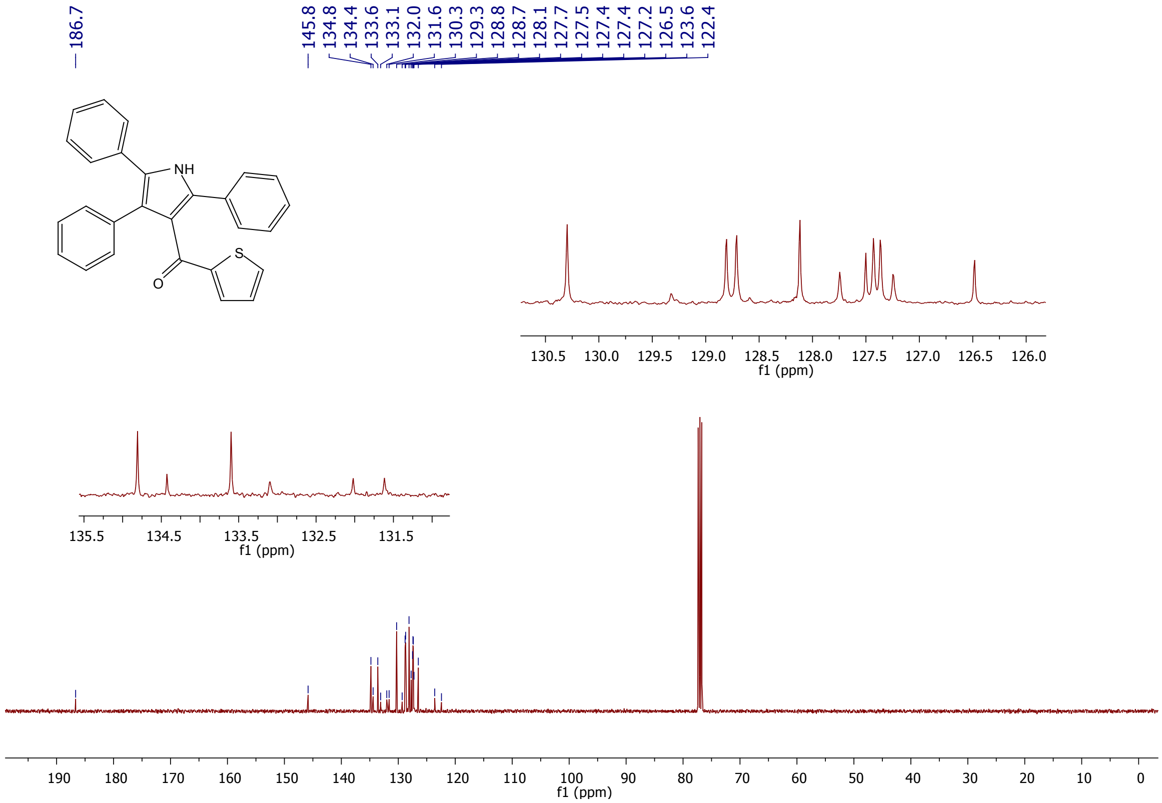

Figure S125. ^{13}**C{**^{1}**H} NMR** (100 MHz, $CDCl_3$) of compound **5g.**
